# Forward Genetics Approach Reveals a Mutation in bHLH Transcription Factor-Encoding Gene as the Best Candidate for the Root Hairless Phenotype in Barley

**DOI:** 10.3389/fpls.2018.01229

**Published:** 2018-09-03

**Authors:** Patrycja Gajewska, Agnieszka Janiak, Miroslaw Kwasniewski, Piotr Kędziorski, Iwona Szarejko

**Affiliations:** ^1^Department of Genetics, University of Silesia in Katowice, Katowice, Poland; ^2^Centre for Bioinformatics and Data Analysis, Medical University of Bialystok, Bialystok, Poland

**Keywords:** barley, gene mapping, mutants, root hairs, rhizodermis pattern

## Abstract

Root hairs are the part of root architecture contributing significantly to the root surface area. Their role is particularly substantial in maintaining plant growth under stress conditions, however, knowledge on mechanism of root hair differentiation is still limited for majority of crop species, including barley. Here, we report the results of a map-based identification of a candidate gene responsible for the lack of root epidermal cell differentiation, which results in the lack of root hairs in barley. The analysis was based on the root hairless barley mutant *rhl1.b*, obtained after chemical mutagenesis of spring cultivar ‘Karat’. The *rhl1* gene was located in chromosome 7HS in our previous studies. Fine mapping allowed to narrow the interval encompassing *rhl1* gene to 3.7 cM, which on physical barley map spans a region of 577 kb. Five high confidence genes are located within this region and their sequencing resulted in the identification of A>T mutation in one candidate, HORVU7Hr1G030250 (MLOC_38567), differing the mutant from its parent variety. The mutation, located in the 3′ splice-junction site, caused the retention of the last intron, 98 bp long, in mRNA of *rhl1.b* allele. This resulted in the frameshift, the synthesis of 71 abnormal amino acids and introduction of premature STOP codon in mRNA. The mutation was present in the recombinants from the mapping population (F_2_ *rhl1.b* × ‘Morex’) that lacked root hairs. The candidate gene encodes a bHLH transcription factor with LRL domain and may be involved in early stages of root hair cell development. We discuss the possible involvement of HORVU7Hr1G030250 in this process, as the best candidate responsible for early stages of rhizodermis differentiation in barley.

## Introduction

Root hairs are highly polarized long tubular outgrowths of the specialized root epidermal (rhizodermal) cells. They play a role in the absorption of water and mineral nutrients, such as nitrogen, phosphorous, potassium, sulfur, and iron (Mimura et al., 1990; Lauter et al., 1996; Hartje et al., 2000; Takahashi et al., 2000; Vert et al., 2002), which is related to the presence of nutrient transporters within the root hair cells. They are also important for anchoring the plant in the soil (Cutter, 1978; Peterson and Farquhar, 1996) and the establishment of plant–microbe interactions (Hofer, 1991; Oldroyd, 2001; Oldroyd and Dixon, 2014). The study of barley and wheat showed that root hairs considerably increase the root surface area (RSA) in comparison to RSA without root hairs, although the magnitude of their effect was genotype specific. It has been calculated for various barley cultivars that root hairs may increase RSA by 112–245% and in wheat cultivars they rise RSA by 95–341% (Gahoonia et al., 1997). Such significant increase may have a huge impact on water and nutrient uptake (Richardson et al., 2011; Haling et al., 2014). Although the existence of root hairs seems to be inessential for the plant yield under optimal water and nutrient conditions (Wen and Schnable, 1994; Chmielewska et al., 2014), their lack may negatively influence plant performance under the stress. The studies of phosphorous uptake in barley showed that in plants grown under low P availability, the length and density of root hairs increased, compared to plants cultivated in the optimal P content (Gahoonia et al., 1997; Brown et al., 2012; Haling et al., 2013; Zhang et al., 2018). Interestingly, there are not many experimental studies describing the role of root hairs in water uptake. Some evidences of their importance in this process come from the study of a root hairless mutant *brb* (*bald root barley*) and its parent variety ‘Pallas’, which demonstrated that the wild-type roots exhibit 55% higher efficiency of water uptake in the comparison to the hairless mutant (Segal et al., 2008). Brown et al. (2012) demonstrated that the lack of root hairs in barley has an adverse effect on the grain yield and plant growth, when a combination of extreme phosphorus deficiency and water stress was applied. Recently, the hypothesis of the possible role of root hairs as environmental sensors was also proposed (Kwasniewski et al., 2016). It emerged from the transcriptome analysis of a *root hairless 1.a* (*rhl1.a*) barley mutant and its parent variety ‘Karat’ subjected to drought stress. It has been found that in the roots of ‘Karat’ variety, a specific activation of genes related to water-stress signaling and protection against stress occurred under drought, which was not found in the roots of the mutant. Thus, it is likely that the presence of root hairs facilitate the activation of drought tolerance mechanisms via appropriate sensing of adverse environmental conditions (Kwasniewski et al., 2016). All these studies indicate the potential of root hairs to improve crop yield and stress tolerance, especially for low-quality soil cultivation (Meister et al., 2014). However, to take advantage of this potential in breeding programs, it is necessary to understand the genetic and molecular mechanisms underlying root hair development.

The most fundamental step of root hair morphogenesis is related to the establishment of the rhizodermis pattern and the formation of two types of cells: trichoblasts, which will further develop the root hair outgrowths, and atrichoblasts, which are not capable of producing a root hair. Three main types of trichoblast/atrichoblast patterns have been distinguished within the plant kingdom (Leavitt, 1904; Dolan and Costa, 2001; Marzec et al., 2014). In plants characterized by the first type of rhizodermis pattern, trichoblasts and atrichoblasts are undistinguishable and any rhizodermal cell is capable of producing a root hair. Nevertheless, the development of a root hair by each rhizodermal cell is not always accomplished and this pattern is characterized by a random distribution of root hairs along the root. The second type of patterning results from the formation of a shorter trichoblast and a longer atrichoblast cell. These two cell types are usually formed by an asymmetric division of a mother rhizodermal cell, reported for, e.g., in rice (Kim and Dolan, 2011), or by a symmetric division followed by asymmetric expansion of daughter cells, described in barley (Marzec et al., 2013). In the third type of rhizodermis patterning, the trichoblasts are arranged in files along the root and are separated by one or more stripes of root hairless cells (Dolan, 1996; Kwak and Schiefelbein, 2007; Horn et al., 2009). The developmental fate of a rhizodermal cell depends on its position in regards to the underlying cortex cells. Trichoblasts are located over the anticlinal cortical cell walls, whereas cells directed toward atrichoblast formation lay over periclinal cortical cell walls (Dolan and Costa, 2001). The molecular background of this type of patterning is well described in the model species *Arabidopsis thaliana*.

The central gene responsible for rhizodermis patterning in Arabidopsis encodes a homeodomain protein GLABRA2 (GL2) which blocks the expression of genes required for root hair development and promotes atrichoblast formation. To form a non-root-hair cell, *GL2* is positively regulated by the protein complex composed of the MYB-type transcription factor WEREWOLF (WER), the basic helix-loop-helix (bHLH) protein GLABRA3 (GL3), the ENHANCER OF GLABRA3 (EGL3) and the WD repeats protein TRANSPARENT TESTA GLABRA1 (TTG1; Schiefelbein et al., 2009). In trichoblasts, the expression of WER is reduced by a signal from the cortex cells that is generated by the JACKDAW (JKD) protein (Hassan et al., 2010) and perceived by the SCRAMBLED (SCM) kinase in the rhizodermal cell (Kwak et al., 2005). The expression of *WER* is also negatively affected by TORNADO1 protein (Kwak et al., 2015). When WER protein is less abundant in the rhizodermal cell, it is substituted in the WER-GL3/EGL3-TTG1 complex by other MYB-type proteins: CAPRICE (CPC), TRYPTICHON (TRY) and ENHANCER OF TRY AND CPC 1 (ETC1), which results in the formation of another transcriptional complex: CPC/TRY/ETC1-GL3/EGL3-TTG1 (Schiefelbein et al., 2014). Then, GL2 expression is not supported anymore, resulting in the activation of genes promoting root hair development. Several other feedback mechanisms are also involved in the cell fate programming in rhizodermis, including mutual transport of CPC and GL3/EGL3 proteins to neighboring tricho- and atrichoblasts, respectively (reviewed by Schiefelbein et al., 2014; Salazar-Henao et al., 2016).

Taking into consideration that the rhizodermis pattern in monocots is different from the Arabidopsis pattern, one may suppose that also molecular mechanisms that control tricho- and atrichoblast formation may, at least partially, require a different regulatory network. Nevertheless, neither rhizodermis patterning nor other stages of root hair formation were studied deeply in monocot species, mostly due to the limited number of mutants of different root hair phenotypes. Up to date, only some genes involved in different stages of root hair formation were described at the molecular level using mutants and/or overexpression lines in *Zea mays* (Wen et al., 2005; Hochholdinger et al., 2008; Nestler et al., 2014), *Oryza sativa* (Kim et al., 2007; Ding et al., 2009; Yuo et al., 2009, 2011; Huang et al., 2013a,b, Wang et al., 2014; Han et al., 2015) and *Brachypodium distachyon* (Kim and Dolan, 2016).

The availability of mutants with the alteration of specific phenotypic traits gives the opportunity to study the genetic basis of these phenotypes using a forward genetic approach, which includes linkage mapping and map-based isolation of a gene of interest. Additionally, recent development of next generation sequencing technologies opened the way to accelerate gene cloning (Mascher et al., 2013; Pankin et al., 2014). The number of studies dedicated to gene isolation via forward genetic approach proves that it is an important and universal strategy that enables the identification of genetic components of many important agrobotanical traits.

Here, we present an example of forward genetics approach to identify a gene sequence underlying the phenotypic change in a mutant. We report the results of a map-based identification of the best candidate gene which may be responsible for rhizodermis patterning in barley (*Hordeum vulgare* L.). The candidate gene encodes a bHLH transcription factor that shows the highest similarity to the bHLH subfamily XI (Heim et al., 2003). This subfamily possesses a conserved LRL (LOTUS JAPONICUS ROOTHAIRLESS1-LIKE) domain between the bHLH domain and the C terminus of the protein (Tam et al., 2015). We identified an A to T mutation in *rhl1.b* allele that resulted in the retention of the last intron of the *bHLH* mRNA sequence, a frameshift and the loss of functional LRL domain. Such a significant truncation of bHLH protein most probably confines its ability to regulate the expression of downstream targets and results in the lack of root hairs in the *rhl1.b* mutant.

## Materials and Methods

### Plant Material

The barley root hairless mutant *rhl1.b* used in the study was developed at the Department of Genetics, University of Silesia in Katowice, Poland, after a double treatment of spring cultivar ‘Karat’ with *N*-methyl-*N*-nitroso urea (MNU) and a 6 h inter-incubation period between treatments. The mutant is characterized by a complete lack of root hairs and a non-differentiated rhizodermis pattern, without distinguishable tricho- and atrichoblasts (Marzec et al., 2013; Chmielewska et al., 2014). A single recessive gene is responsible for the mutant phenotype (Szarejko et al., 2005). Despite the significant differences in the morphology of root hair zone in *rhl1.b* mutant and its parent cv. ‘Karat’, no other morphological differences were found in plant architecture between them. Also, the grain yield of the mutant was similar to the parent variety, when the plants were grown under optimal nutrient content and irrigation (Chmielewska et al., 2014).

Additionally, a spontaneous root hairless mutant *brb* (*bald root barley*) from cv. ‘Pallas’ (Gahoonia et al., 2001), which was kindly provided by the Department of Agricultural Sciences, The Royal Veterinary and Agricultural University, Denmark, was included in the analysis. Our previous studies showed that the root hair phenotype and rhizodermis pattern of *brb* mutant were identical to those of the *rhl1.b* (Marzec et al., 2013) and that both mutants were allelic (Chmielewska et al., 2014).

For the purpose of this study, a F_2_ population of 4,959 individuals derived from the cross of *rhl1.b* mutant × cultivar ‘Morex’ was developed and used in genetic mapping. ‘Morex’ is a barley variety whose genome represents the barley reference genome sequence (Mayer et al., 2012). A new, enriched version of ‘Morex’ genomic sequence is also available (Mascher et al., 2017).

### Growth Conditions and Phenotyping

In order to phenotype the mapping population, F_2_ grains from the cross *rhl1.b* × ‘Morex’ were surface-sterilized in 20% ACE detergent, washed three times in sterile water and transferred to Petri dishes filled with 1% solid agar. Petri dishes were protected from light and incubated at 4°C for 24 h followed by 98 h at 18°C. Phenotyping of root hair zone of each F_2_ individual was performed using light stereoscope microscopy. The phenotypes were classified into two categories: with normal wild type (WT) root hairs and completely root hairless. The 3:1 segregation was examined using the χ^2^ test. After phenotyping, plants were transferred to pots filled with soil and vermiculite mixture and grown in a greenhouse under 16/8 h photoperiod at 20/17°C (day/night) and light intensity of 420 μEm^-2^s^-1^. They were grown several weeks in order to develop enough leaf material for efficient DNA extraction. After the tissue for DNA extraction was collected, plants with mutant phenotype were grown until maturity.

In order to extract RNA from roots, grains of *rhl1.b* and *brb* mutants together with their respective parent varieties ‘Karat’ and ‘Pallas’ were sterilized in the same way as mentioned above and transferred on a wet cotton wool plug placed in the top of a sterile glass tube, one grain per tube. Two glass tubes were sealed together with a parafilm to form closed container for aeroponic growth. Bottom part of the tubes was covered by aluminum foil, to protect it from the access of light. Glass tubes were placed in a growth chamber under 16/8 h photoperiod at 18/18°C (day/night) and light intensity of 180 μEm^-2^s^-1^ for 6 days.

### DNA Extraction

Leaves of individual F_2_ plants, the mutants and respective varieties were collected from 5-week-old plants, placed in a plastic zipper bag filled with silica gel and left to dry for 2 weeks. Dried material was grinded using Retsch MM200 mill (Retsch, Germany) and subjected to DNA extraction using a modified micro-CTAB method (Doyle and Doyle, 1987).

### RNA Extraction

RNA was isolated from 6-day-old roots which were divided into 1 mm long segments representing subsequent root zones: (1) root apical zone, (2) elongation and differentiation zone, and (3) root hair zone (**Supplementary Figure S1**). All steps of RNA isolation were carried out under RNAse free conditions. Each sample was homogenized in 1 ml TRIzol^®^ reagent for 5 min (Invitrogen GmbH, Germany). Afterward, homogenized samples were transferred to RNase free collection tubes and subjected to centrifugation (14,000 × *g* for 3 min at 4 °C) in order to sediment the cell debris at the bottom of the tube. The clean supernatant was then transferred to a new RNase-free tube and subjected to RNA extraction using RNAqueous Kit (AMBION Thermo Scientific, Waltham, MA, United States) according to the manufacturer’s instructions.

### Markers Development

Several resources of molecular markers were used in the presented analysis. Three SSR loci: GBM1464, scssr07970 and EBmac0016 described in our previous first-pass mapping (Chmielewska et al., 2014) were used as the starting points for linkage analysis in the extended mapping population. These markers, however, were mapped on genetic maps only and their physical location in barley chromosome 7HS was unknown. For this reason, the information on barley genetic maps from several mapping populations and the virtually ordered gene map of barley, Genome Zipper (Mayer et al., 2011) were used to approximately align our first-pass map of *rhl1* gene region with the maps based on SNP markers with known physical position. The comparison of our map with the others was based on several selected genetic intervals, which included fragments of 7HS from the map published by Rostoks et al. (2005), Stein et al. (2007), Muñoz-Amatriaín et al. (2011), the maps of SM POPA123 and MB POPA123 of Close et al. (2009), and Hordeum-PilotOPA1-7H map available in GrainGenes Database. Common markers from these maps were compared to the Genome Zipper resource and subsequently, this last resource was used to select additional sequences that allowed to create anchoring points between *rhl1* gene region and the sequences with known physical location in 7HS chromosome. Additional markers were developed using the information from Ensembl Plants database, genome assembly version 082214v1. The candidate genes were selected using the new barley genome assembly version Hv_IBSC_PGSB_v2^1^ (**Supplementary Table S1**).

Primers for the PCR amplification of all markers and candidate gene sequences were designed using the online software Primer3^2^ (Untergasser et al., 2007; **Supplementary Tables S2, S3**). A BLAST search against barley genome was used to check the specificity of primers to their target sequences. After amplification, the identity of PCR fragments was verified by their alignment to the sequences from databases using CodonCode Aligner software version 3.0 (CodonCode Corporation, Dedham, MA, United States). This software was also used to assemble the whole gene sequences when their amplification was carried out in shorter fragments, to identify the polymorphic sites between parents of the mapping population and to compare the analyzed sequences of *rhl1.b* and *brb* mutants with their respective parent varieties.

Different genotyping procedures were used depending on the type of polymorphism detected between parents of mapping population: direct agarose gel electrophoresis, Cleaved Amplified Polymorphic Sequences (CAPS), High Resolution Melting (HRM), and EcoTILLING. In the case of two markers, the region with target SNP was subjected to Sanger sequencing in a small subset of recombinants (**Supplementary Table S1**).

### Genotyping Methods

#### InDel Markers

In several instances InDel polymorphisms of more than four nucleotides were identified between parents of the mapping population, *rhl1.b* mutant and ‘Morex’ variety. In such cases, the specific marker sequences were amplified using PCR reaction and then directly analyzed using 2–3% agarose gel in 0.5 × TBE buffer.

#### CAPS Markers

Each SNP and small InDel polymorphism that was identified between *rhl1.b* mutant and ‘Morex’ variety was subjected to the analysis in NEBcutter software (Vincze et al., 2003) to check, whether it is possible to convert the polymorphism into CAPS marker. If the appropriate restriction enzymes were identified, the following procedure of genotyping was applied. The PCR reaction was performed in a 20 μl volume containing 50–100 ng of template DNA, 1 × PCR buffer (20 mM Tris-HCl, pH 8.0 at 22°C, 1.5 mM MgCl_2_, 100 mM KCl and 50% glycelor), 5 pmol of each primer, 250 mM of each of dNTPs and 1U of TaqDNA polymerase (Color Taq DNA Polymerase, EURx, Poland). The annealing temperatures and number of PCR cycles in the PCR profiles depended on the amplified fragment. The PCR steps that were common for each amplification were as follows: initial denaturation of 5 min at 94°C, denaturation repeated in cycles: 45 s at 94°C, primers annealing lasting for 45 s at specific temperature, elongation for 1 min at 72°C, and final extension at 72°C for 5 min.

After the amplification, 2.5 μl of the products was subjected to restriction reaction in 7.5 μl total volume containing 1 × concentrated buffer for appropriate restriction enzyme and 1 unit of the enzyme (New England Biolabs, United Kingdom). The restriction reaction was carried out for 3 h in a water bath at the temperature optimal for each of enzyme used. The visualization of CAPS markers was performed on a 1% agarose gel in 0.5 × TBE buffer.

#### High Resolution Melting (HRM) Method

One SNP marker could not be transformed into CAPS and it was genotyped using HRM method (**Supplementary Table S1**), followed by the protocol described by Szurman-Zubrzycka et al. (2017), which allows to differentiate homozygotes and heterozygotes. The reaction was prepared in 20 μl volume with a ready-made High Resolution Melting Master Kit version 06 (Roche Diagnostics, Germany) according to manufacturer’s protocol, with 0.2 μM of each primer, 0.75 mM MgCl_2_ and 20 ng of template DNA.

The reaction was carried out using a LightCycler^®^ 480 instrument (Roche Diagnostics, Germany) with the following conditions: pre-incubation step at 95°C for 10 min, 42 cycles of amplification (denaturation at 95°C for 10 s, annealing at 56°C for 15 s and elongation at 72°C for 20 s with single fluorescence acquisition), formation of duplexes step consisting of 95°C for 1 min, 40°C for 1 min and melting interval of 65°–95°C, with continuous fluorescence acquisition. The data analysis was performed using the LightCycler^®^ 480 Gene Scanning Software Version 1.5.0 SP4 (Roche Diagnostics, Germany) following the software user guide.

#### EcoTILLING Method

The EcoTILLING method (Comai et al., 2004) was used to analyze the segregation of a mutation identified in a candidate HORVU7Hr1G030250 (MLOC_38567) gene in the F_2_ mapping population *rhl1.b* × ‘Morex’ (**Supplementary Table S1**). Before PCR reaction, two DNA samples were prepared for each F_2_ individual. The first one included DNA of a particular F_2_ individual only. The second one was a mixture of the first sample (DNA of an individual F_2_ plant) and reference DNA (DNA of WT ‘Morex’). The EcoTILLING of both samples allowed to determine whether a particular F_2_ plant is homo- or heterozygous in regards to the analyzed SNP. The PCR reaction mixture of 20 μl included 100 ng of template DNA, 1 × PCR buffer (20 mM Tris-HCl, pH 8.0 at 22°C, 1.5 mM MgCl_2_, 100 mM KCl and 50% glycelor), 5 pmol of each primer, 250 mM of each of dNTPs and 1U of TaqDNA polymerase (Color Taq DNA Polymerase, EURx, Poland) and 10 pmol of primers mixture. This mixture was composed of forward and reverse unlabelled primers together with forward and reverse primers labeled by IRDye-700 and IRDye-800 fluorescence dyes, respectively. The proportion of labeled to unlabelled primers was 3:2 for forward and 4:1 for reverse primers. The PCR cycling profile was composed of initial denaturation for 5 min at 94°C, 33 cycles of denaturation for 45 s at 94°C, primers annealing for 45 s at 58°C, elongation for 1 min at 72°C, followed by final extension at 72°C for 5 min.

After the PCR reaction, the products were subjected to heteroduplex formation and digestion by CelI enzyme, followed by the purification of digestion products and polyacrylamide gel electrophoresis, according to the protocol described by Szurman-Zubrzycka et al. (2017).

### Linkage Map Construction

The linkage analysis was performed using JoinMap 3.0 program (Van Ooijen and Voorrips, 2001). The marker genotypes of the F_2_ individuals from mapping population were encoded as ‘a’ in case of homozygote identical to the *rhl1.b* mutant, ‘b’ in case of homozygote identical to the ‘Morex’ variety and ‘h’ for heterozygote. The root hair phenotype was scored as a dominant trait and plants were encoded as ‘a’ for root hairless homozygotes identical to the *rhl1.b* mutant and ‘c’ in the case of plants with normal root hairs. The distances between markers were calculated according to the Kosambi (1944) function and their grouping was estimated using the LOD score of 3.0.

### Gene Expression Analysis

RNA extracted from root segments of *rhl1.b* and *brb* mutants and their respective parent varieties ‘Karat’ and ‘Pallas’ was subjected to DNase treatment and then used for the cDNA synthesis using a Maxima cDNA Synthesis kit according to manufacturer’s instructions (Thermo Scientific, United States). Prior to qPCR reaction, the cDNAs were diluted five times in ddH_2_O. The reactions were performed using a LightCycler^®^ 480 SYBR^TM^ Green I Master kit and LightCycler^®^ 480 instrument (Roche Diagnostics, Germany). Reaction mixture of total volume of 10 μl included: 2 μl of diluted cDNA, 10 pmol of each PCR primer and 5 μl of SYBR^TM^ Green I Master Mix. The following reaction conditions were used: initial denaturation for 5 min at 95°C, 42 cycles of denaturation for 10 s at 95°C, primer annealing for 15 s at 56°C and elongation for 15 s at 72°C with single fluorescence acquisition, followed by melting curve generation lasting 5 s at 95°C, 1 min at 65°C, and 98°C with ramp of 0.11°C/s with continuous fluorescence measurement.

The gene expression analysis was performed using three biological replicates for each genotype, where one replicate consisted of relevant root segments collected from five plants. For each biological replication two technical replicates of qPCR reactions were performed. The relative expression level of each gene was calculated using 2^(-ΔΔCt)^ method and a gene encoding ADP ribosylation factor 1-like protein (AJ508228.2; Rapacz et al., 2012) was used as a reference gene. The fold change of gene expression for each root zone of the specific genotype (*rhl1.b, brb, ‘Karat’, ‘Pallas’*) was normalized to the gene expression level characteristic to ‘Karat’ root apical zone, which was considered as the value of 1. The statistical significance of gene expression differences between samples was computed using the STATISTICA package [STATISTICA (data analysis software system), version 12. StatSoft, Inc.^3^] for One-Factor Variance Analysis (ANOVA) and LSD test (*P* ≤ 0.05) test.

### *In silico* Sequences Analysis

The physical interval encompassing *rhl1* gene was subjected to *in silico* characterization using several tools for sequence annotation. The analysis of interspersed repeats and low complexity DNA sequences was performed using RepeatMasker Open version 4.0^4^. RepeatMasker PERL script was configured using: RMBlast ver. 2.2.8^5^, TRF (Tandem Repeat Finder) version 4.0.9^6^ and RepBase repeat sequences database version 20170127^7^.

The information on coding sequences within the interval was retrieved directly from EnsemblPlants database and used as queries for BLAST tools from the NCBI database^8^ to compare their nucleotide and protein sequences and to identify homologous genes and proteins in other species. Additionally, the Lotus base^9^ was used to compare sequences from barley to *L. japonicus* genome.

The prediction for gene structure was conducted using Genscan program^10^ (Burge and Karlin, 1997) and Fgenesh program^11^ (Solovyev et al., 2006), using maize and barley as model organisms, respectively. Those analysis were supported by scrutinized mRNA sequences from known homologs.

The identification of conserved protein motif structures was performed using InterProScan^12^ (Jones et al., 2014) with standard settings. Structural alignment of conserved protein sequence was prepared with T-cofffe tool^13^ (Tree based Consistency Objective Function For AlignmEnt Evaluation, Notredame et al., 2000) supported by graphical visualization in SeaView software version 4.0^14^(Gouy et al., 2010). The identification of promotor motif L1 box-like was performed with Blast-2-Seq tool using as a query the 5′-TAAATGT-3′ motif and as a subject the sequence of the candidate gene together with 3 kb region upstream to the start codon.

The maximum likelihood phylogenetic tree of bHLH protein family was constructed using W-IQ-TREE tool (Nguyen et al., 2015; Trifinopoulos et al., 2016). The sequence of a protein encoded by HORVU7Hr1G030250 (MLOC_38567) candidate gene was compared to other bHLH proteins from barley and rice and the several sequences with the highest similarity were used in the analysis together with the *Lotus japonicus* Ljrhl1 (Karas et al., 2009) and rice Osrhl1 (Ding et al., 2009) proteins. The protein sequences from Arabidopsis bHLH family, previously described by Heim et al. (2003) and Toledo-Ortiz et al. (2003), were used as a background for the phylogenetic tree construction (**Supplementary Table S4**). All protein sequences were aligned using Clustal Omega from EMBL-EBI service^15^). Alignment file was used as an input to W-IQ-TREE. The ModelFinder algorithm (Kalyaanamoorthy et al., 2017) was used to identify the most appropriate substitution model and ultrafast bootstrap (Hoang et al., 2017) together with SH-aLRT tests (Guindon et al., 2010) were used to evaluate the statistical support of tree branches.

## Results

### Fine Mapping of *rhl1* Gene Region

The fine mapping of *rhl1* gene was based on a large mapping population, which consisted of 4,952 F_2_ individuals from the *rhl1.b* × ‘Morex’ cross. This population showed the expected 3:1 segregation for the plants with normal root hairs to the root hairless individuals, confirmed by the value of 0.0097 of χ^2^_3:1_ test. All 1,238 root hairless F_2_ individuals were genotyped for three SSR markers: scssr07970, GBM1464, and EBmatc0016, which were found in our previous first-pass mapping as the closest points flanking *rhl1* gene at the distance of 1.7, 1.73, and 4.6 cM, respectively (Chmielewska et al., 2014). This analysis allowed to select 228 root hairless recombinants between SSR markers and *rhl1* gene. As the physical location of any of the markers from the first-pass genetic map was not known, all 228 F_2_ recombinants served as the material for subsequent screening for additional markers that might allow to anchor the *rhl1* gene region on barley physical map. To meet this aim, four loci retrieved from the virtually ordered gene map of barley Genome Zipper (Mayer et al., 2011) were analyzed among 228 F_2_ root-hairless recombinants. In the case of one marker (3_0530) the initial analysis of 96 F_2_ recombinants showed that 71 of them were heterozygous, indicating a large distance between this locus and *rhl1* gene. For this reason this marker was excluded from further analysis. For three other markers, 2_1491, 3_0752 and 1_0772, the 17, 6, and 4 recombination events were found, respectively (**Table 1**).

**Table 1:** The segregation of three SSR markers and four loci derived from Genome Zipper database among 228 F_2_ root-hairless recombinants from *rhl1.b* × ‘Morex’ mapping population.

Locus	Genotypic classes			
	a	h	c	X
*rhl1*	228	0	0	0
Scssr07970	181	47	0	0
GBM1464	186	42	0	0
EBmac0016	49	179	0	0
3_0530	96	71	0	132
2_1491	211	17	0	0
1_0772	224	4	0	0
3_0752	222	6	0	0

*a, homozygote as *rhl1.b* mutant; h, heterozygote; c, homozygote as ‘Morex’, x, missing data.*

**FIGURE 1:**
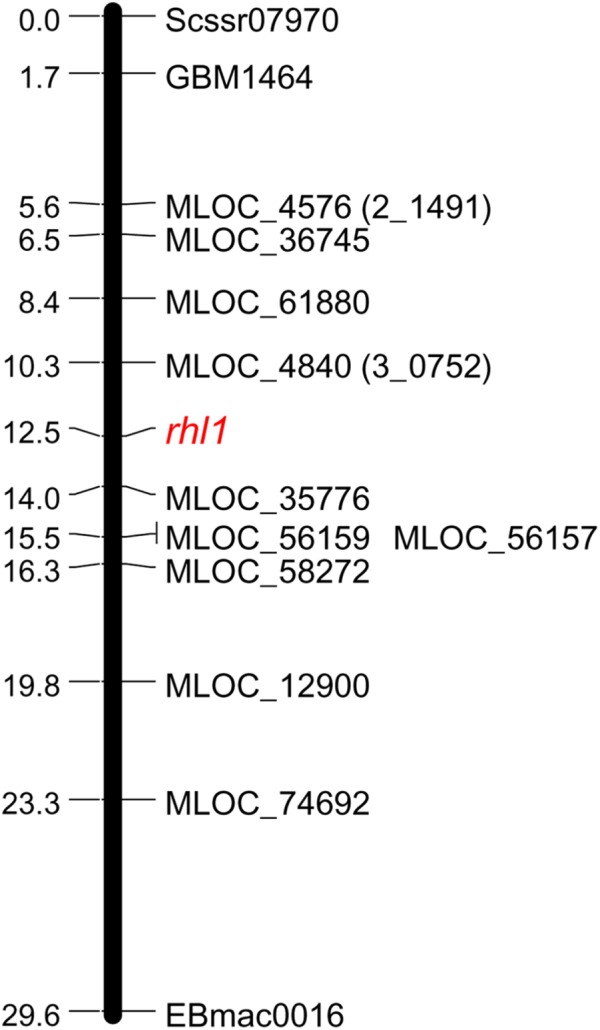
Linkage group spanning the region of *rhl1* gene in chromosome 7HS, based on the F_2_ *rhl1.b* × ‘Morex’ mapping population.

The following work focused on the genetic mapping of 2_1491 and 3_0752 markers in the region of *rhl1* gene, in order to find their genetic position in reference to our locus of interest. The third marker retrieved from Genome Zipper database, locus 1_0772, was not subjected to mapping, because of too high costs of restriction enzyme for CAPS or any other available genotyping method for the marker. Also, to make this and the subsequent analysis cost-effective and laboratory feasible, a sub-population was created based on 1,472 F_2_ plants from *rhl1.b* × ‘Morex’ mapping population. This sub-population consisted of 1,079 F_2_ plants with normal root hairs and 393 root hairless plants, which were randomly chosen from the available large (4,952 plants) F_2_ population. Phenotypic classes of the selected sub-set of F_2_ individuals retained the 3:1 segregation ratio, which was the only criterion for this selection. Using this sub-population, both Genome Zipper markers (2_1491 and 3_0752) were mapped on same site of *rhl1* locus, between the gene and Scssr07970 and GBM1464 SSR markers (**Figure 1**). Despite the lack of marker that would flank our target gene on the other site, these two markers were further used to physically anchor the region of interest in barley genome using the data from Ensembl Plants database (barley genome version 082214v1).

In order to find the second locus flanking the *rhl1* locus, nine arbitrarily selected barley genes located downstream from 2_1491 marker (corresponding to MLOC_4579 gene on barley physical map), were used (**Table 2**). The fine mapping was performed in the same sub-population of 1,472 F_2_ plants from the cross of *rhl1.b* and ‘Morex’. The aim of this analysis was to find the second locus flanking our gene of interest (*rhl1*) and to narrow down the interval around the gene. Altogether, the segregation of 11 markers was analyzed in the sub-population (**Table 2**) and 10 of them were used to create a linkage map of the *rhl1* gene region (**Figure 1**). One marker, MLOC_61391, was excluded from this analysis, because of its distorted segregation that resulted in erroneous positioning of several other loci on the genetic map. The final linkage group showed that the position of each marker was consistent with its physical location in barley chromosome 7HS. The map spans 29.6 cM and contains three SSR markers from our first-pass mapping, two markers retrieved from Genome Zipper database and eight loci representing barley genes from the Ensembl Plants database, assembly version 082214v1 (**Supplementary Tables S1, S2**). Two markers: MLOC_4840 (corresponding to the 3_0752 locus from Genome Zipper) and MLOC_35776 were mapped in the closest proximity to the *rhl1* gene, at the distance of 2.2 cM and 1.5 cM, respectively. These markers were used as the flanking borders of the physical interval for a subsequent analysis.

**Table 2:** Segregation of loci used to fine map the *rhl1* gene region.

Locus	Source of the marker	Genotypic classes					
		a	h	b	c	x	χ^2^
Scssr07970	SSR^∗^	366	728	378	0	0	0.37
GBM1464	SSR^∗^	364	736	372	0	0	0.09
MLOC_4579 (2_1491)	GZ	367	722	368	0	15	0.12
MLOC_36745	EP	368	728	376	0	0	0.26
MLOC_61880	EP	382	702	387	0	1	3.09
MLOC_4840 (3_0752)	GZ	376	719	376	0	1	0.74
*rhl1*		393	0	0	1,079	0	0.99
MLOC_35776	EP	387	699	385	0	1	3.63
MLOC_61391	EP	380	764	327	0	1	6.03
MLOC_56157	EP	385	705	382	0	0	2.62
MLOC_56159	EP	385	705	382	0	0	2.62
MLOC_58272	EP	378	716	376	0	2	0.99
MLOC_12900	EP	353	737	368	0	14	0.48
MLOC_74692	EP	335	741	372	0	24	2.69
EBmac0016	SSR^∗^	338	741	372	0	21	2.26

*^∗^SSR markers from the first-pass mapping analysis (Chmielewska et al., 2014); a, homozygote as *rhl1.b* mutant; b, homozygote as cv. ‘Morex’; c, genotype other than “a”; h, heterozygote; x, missing data; GZ, Genome Zipper; EP, Ensembl Plants.*

### The Characterization of Physical Interval Spanning *rhl1* Locus

The characterization of the identified interval was based on the new release of barley genome (version Hv_IBSC_PGSB_v2), where gene IDs were changed from MLOCs to HORVU identifiers. Thus, the corresponding new identifiers of the flanking MLOCs were found and further used in our analysis. The interval between MLOC_4840 (HORVU7Hr1G030210) and MLOC_35776 (HORVU7Hr1G030300) sequences encompasses a 577,263 bp distance. The annotation of this region showed that the total of 238,047 bp consisted of interspersed sequences and 41.3% of them belong to transposable elements (**Table 3**). Vast majority of these elements represent LTR retrotransposon classes, mostly from *Tyl/Copia* and *Gypsy/DIRS1* sub-types (18.2% and 19.5% of the total sequence, respectively). Much smaller fraction of the interval, accounting for 741 bp, consisted of LINE class retroelements. Nearly 3.5% of the sequence was classified as DNA transposons and 0.7% as different repeats including simple repeats, low complexity repeats and satellite DNA. A substantial region of the interval is composed of the unknown sequence. It was further used as a query for BLAST search against the genomic sequences of barley, rice, wheat, *Aegilops tauschii, Setaria italica*, and *Zea mays* in NCBI database and no significant hits to known genes were found.

**Table 3:** Annotation summary of the 577,263 bp physical interval encompassing the *rhl1* locus.

Sequence class	Total length (bp)	Percentage of sequence (%)
Retrotransposons	217,999	37.8
DNA transposons	20,048	3.5
Simple repeats	3,513	0.6
Low complexity repeats	347	0.1
Satellites DNA	158	0.03
High confidence gene sequences	13,352	2.3
Unclassified region	321,846	55.7
**Total**	**577,263**	**100**

Only five high confidence (HC) gene sequences were found within the target interval and their total length represented 2.3% of that region (**Table 3**). Each of the genes was characterized based on its ontology and sequence comparison to putative orthologs from *A. thaliana, O. sativa*, and *B. distachyon* (**Table 4**).

**Table 4:** The putative function and the orthologous sequences of five HC genes present in the physical interval encompassing the *rhl1* locus.

MLOC ID	HORVU ID	Putative gene product^∗^	Putative orthologs^∗^		
			*Arabidopsis thaliana*	*Oryza sativa*	*Brachypodium distachyon*
MLOC_39064	HORVU7Hr1G030220	RTF2 RING-finger protein involved in mitotic DNA replication termination	AT5G58020	OS06G0183900	BRADI1G47360
MLOC_38567	HORVU7Hr1G030250	Transcription factor bHLH with protein dimerization activity	AT2G24260	OS06G0184000	BRADI1G47350
MLOC_17531	HORVU7Hr1G030270	Transcription factor belonging to Krüppel-like family	AT5G16950	OS01G0258600	BRADI1G47340
MLOC_36656	HORVU7Hr1G030280	28S ribosomal S34 protein	AT5G52370	OS02G0481000	BRADI3G43520
MLOC_75365	HORVU7Hr1G030290	Protein involved in polar localization during asymmetric cell division and redistribution	No orthologs	Os02g0795200	BRADI1G47330

*^∗^Predictions were based on the gene annotation of the most similar homologs from other species. Blastp methods was used to compare the relevant protein sequences. MLOC ID, gene ID according to barley genome assembly version 082214v1; HORVU ID, gene ID according to barley genome assembly version Hv_IBSC_PGSB_v2.*

**FIGURE 2:**
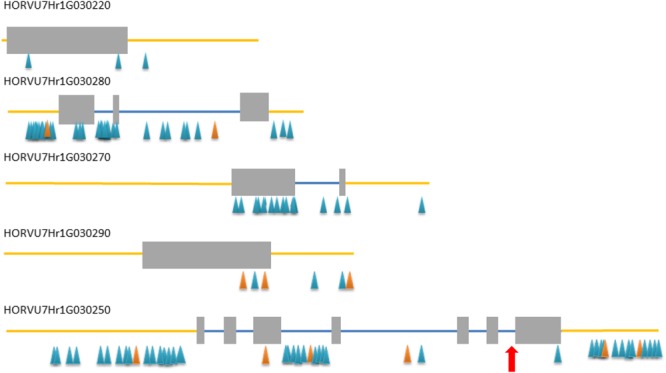
Exon–intron structure of five high confidence (HC) genes from the target interval. Gray rectangles – exons, blue lines – introns, yellow line – upstream and downstream region, blue/orange triangles – SNP/InDel polymorphisms detected between *rhl1* mutant and cv. ‘Morex’. Red arrow indicates the position of a mutation in HORVU7Hr1G030250 differing *rhl1.b* mutant and cv. ‘Karat’.

All five HC genes were sequenced in *rhl1.b* mutant and its parent cultivar ‘Karat’, in order to find a mutation that may be responsible for the changed rhizodermis pattern resulting in the lack of root hairs in the mutant (**Supplementary Figures S2, S3**). The primers for the amplification of candidate genes were designed in such a way as to cover the whole exon–intron structure of each gene (**Supplementary Table S3**). Additionally, selected fragments of these genes were also analyzed in cv. ‘Morex’ in order to discover the polymorphic sites between the mutant and ‘Morex’ (**Figure 2**) that were used for linkage analysis in the mapping population.

**FIGURE 3:**
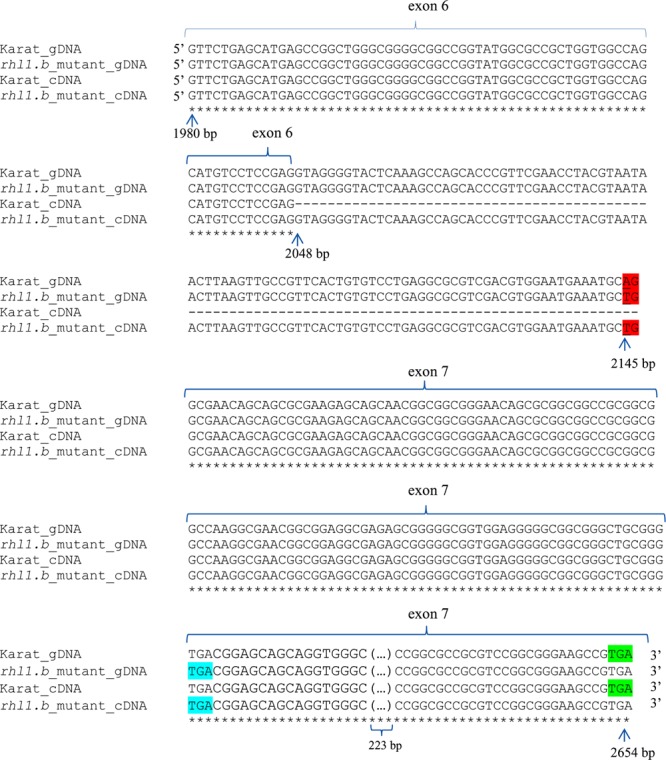
The alignment of a part of the HORVU7Hr1G030250 genomic and cDNA gene sequences representing exon 6th, intron 6th, and exon 7th of *rhl1.b* mutant and its parent variety ‘Karat’. The mutant allele harbors a single base-pair change A>T located in acceptor splice sites and is highlighted by a red color. Blue color represent the premature STOP codon in the gene sequence in the mutant. Green color indicates the correct STOP codon in the gene of cv. ‘Karat’. gDNA, genomic sequence; cDNA, transcript sequence. All nucleotides positions are related to the start codon in HORVU7Hr1G030250 sequence. The part of exon 7th (223 bp long and identical between both genotypes) is not shown.

**FIGURE 4:**
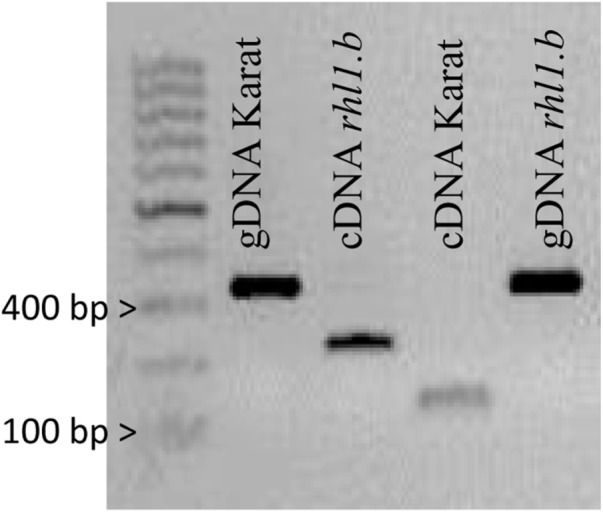
PCR products spanning the part of 5th and 7th exon of HORVU7Hr1G030250 sequence that were amplified using genomic DNA (gDNA) and cDNA as templates in ‘Karat’ and *rhl1.b* mutant. The expected PCR product sizes are 328 bp for gDNA of Karat and *rhl1.b* mutant, 126 bp for cDNA of ‘Karat’ and 223 bp for cDNA of *rhl1.*b mutant, in which the retention of the 6th intron occurs.

**FIGURE 5:**
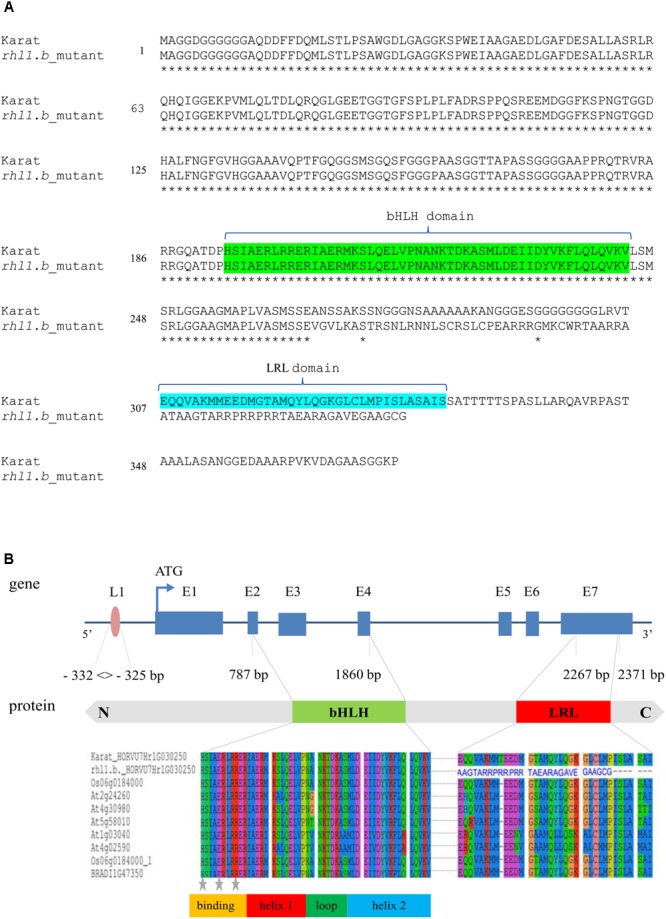
**(A)** The alignments of a putative protein sequences encoded by HORVU7Hr1G030250 (*HvRhl1*) gene for *rhl1.b* mutant and its parent variety ‘Karat’. Identical amino acids are marked by asterisks. bHLH and LRL domains are marked in green and blue, respectively. **(B)** The structure of HORVU7Hr1G030250 gene and the scheme of HORVU7Hr1G030250 protein domains. Dashed line represents the spliced intron in the wild type ‘Karat’. Blue boxes and lines represent exons and introns, respectively. Parts of exons 2nd and 4th and the whole exon 3rd encode the bHLH domain. Part of exon 7th encodes the LRL domain. L1, motif of L1 box-like sequence 5′-TAAATGT-3′ in the promoter region of the gene. N, C, N and C protein terminus, respectively. Below the alignment of the bHLH and LRL protein domains of HORVU7Hr1G030250 sequence from ‘Karat’ and *rhl1.b* mutant together with several *A. thaliana, O. sativa*, and *B. distachyon* homologous proteins are provided. The predicted protein sequence of *rhl1.b* mutant has an incorrect amino acid sequence (blue letters) in the region which corresponds to LRL domain in the wild type protein. Gray stars – conserved amino acid residues which are important for the contact with a nucleotides in DNA.

Only in one gene, HORVU7Hr1G030250, encoding a putative bHLH transcription factor, we found a mutation in *rhl1.b* that distinguished the mutant and parent variety sequences. It was a substitution of A to T nucleotide, located at the 2,145 bp position from the start codon in HORVU7Hr1G030250 sequence (**Figure 3**). *In silico* analysis showed that the mutation is located in the splice-junction acceptor site between the sixth intron and seventh exon and it should result in the retention of the last intron in the bHLH mRNA sequence of the root hairless mutant (**Figure 3** and **Supplementary Figure S3**). In order to prove this prediction, the region spanning a part of fifth and seventh exon of bHLH gene was amplified in *rhl1.b* and cv. ‘Karat’ using genomic DNA and root cDNA as templates. A longer PCR product generated from the cDNA template was observed in the *rhl1.b* mutant compared to the WT ‘Karat’ (**Figure 4**), indicating that the candidate mutation indeed leads to the retention of the last intron and changes the bHLH mRNA structure. As a consequence of intron retention, a frameshift mutation occurs leading to the aberrant synthesis of 71 amino acids and the formation of incorrect STOP codon at the 116 bp position from the beginning of the seventh exon. As a result, the predicted mutated protein is shorter of 58 amino acids and, importantly, lacks the correct sequence of LRL domain, which is present in the wild type protein (**Figure 5**). The LRL loss may impair bHLH activity and preclude the formation of a correct rhizodermis pattern, leading to the lack of root hairs in the mutant.

Next, we performed a co-segregation analysis to verify whether the bHLH encoding gene may be responsible for the root hairless phenotype of the mutant. Here, two approaches were used. The first was based on EcoTILLING procedure targeted directly to the A>T mutation site in the candidate gene and was performed for 96 root hairless F_2_ individuals from *rhl1.b* × ‘Morex’ population. A full co-segregation of the mutant phenotype and the homozygous state of the mutation was found. In the second approach, an In/Del polymorphism detected between *bHLH* sequence of *rhl1.b* mutant and ‘Morex’ variety was used. This polymorphic site is located in the fourth intron, 728 bp upstream from the *rhl1.b* mutation. The segregation of this polymorphism was analyzed in the 1,472 F_2_ plants serving as a sup-population in our linkage analysis. All individuals with mutant phenotype showed the same homozygous genotype for the In/Del marker site, as *rhl1.b* mutant, whereas the individuals with normal root hairs were either heterozygous or homozygous as cv. ‘Morex’. An expected 1:2:1 segregation ratio was found for this In/Del, confirmed by the value of 2.571 of χ^2^_1:2:1_ test (**Table 5**).

**Table 5:** The segregation analysis of an In/Del marker for our candidate HORVU7Hr1G030250 gene within 1,472 F_2_ plants serving as a sup-population in the linkage analysis.

Locus	Genotypic classes					χ^2^
	a	h	b	c	x	
*rhl1*	393	0	0	1,079	0	0.99
HORVU7Hr1G030250_In/Del	393	710	364	0	0	2.57

*a, homozygote as *rhl1.b* mutant; b, homozygote as ‘Morex’ variety; c, genotype other than “a”; h, heterozygote; x, missing data.*

**FIGURE 6:**
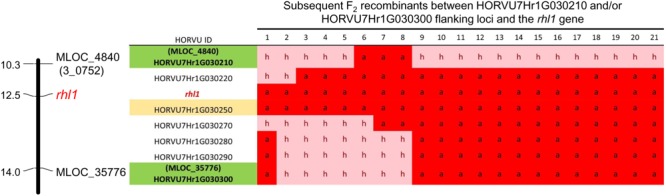
The results of genotyping the F_2_ double and single recombinants identified in the *rhl1* gene interval flanked by MLOC_4840 and MLOC_35776 markers (highlighted in green). The genotyping was performed for all HC genes located in the physical interval between flanking markers. H, heterozygote; a, homozygote as *rhl1.b* mutant. The candidate gene HORVU7Hr1G030250 is highlighted in yellow.

In addition to the above analysis, the information on the polymorphisms between *rhl1.b* mutant and ‘Morex’ variety was used to analyze four other candidate genes from the target physical interval. For this purpose all root hairless F_2_ individuals, which simultaneously showed at least one recombination event between *rhl1* gene and the MLOC_35776 or MLOC_4840 markers flanking the interval of interest (altogether 21 plants), were genotyped for selected SNPs or In/Dels in the candidate genes. The most important finding was the identification of two F_2_ individuals characterized by double recombination events between *rhl1* locus and the genes located on its both sides, in the closest proximity on the physical interval: the HORVU7Hr1G030270 gene encoding a TF from Krüppel-like family and HORVU7Hr1G030220 encoding a RING-finger protein (**Figure 6**). Additionally, four other plants were single recombinants between HORVU7Hr1G030270 and *rhl1* genes. In the case of two other loci: HORVU7Hr1G030280, which encodes 28S ribosomal S34 protein and HORVU7Hr1G030290, presumably responsible for polar localization of proteins during asymmetric cell division, the same pattern of segregation as for the flanking marker MLOC_35776 (HORVU7Hr1G030300) was noticed for all 21 individuals (**Figure 6**). This observation demonstrates that all these loci are located outside of the critical recombination points, in both, the distal and proximal positions from *rhl1* gene. Taking into consideration all the above analysis, the HORVU7Hr1G030250 gene, encoding a putative bHLH transcription factor is the best and the strongest candidate responsible for the lack of root hairs in the *rhl1.b* mutant.

### Structural and Functional Characterization of the Candidate HORVU7Hr1G030250 (*HvRhl1*) Gene

The HORVU7Hr1G030250 (*HvRhl1*) gene is 2,654 bp long and contains seven protein coding exons (363, 75, 159, 66, 65, 69, and 390 bp, respectively), which account for the 1,187 bp of the whole sequence. According to the bioinformatics analysis, it encodes a putative protein of 396 amino acids comprising basic-helix-loop-helix motif characteristic for bHLH transcription factors. The bHLH domain has approximately 50–60 amino acids of a highly conserved amino acid motif, including two amphipathic α-helices separated by a loop. Many plant bHLH proteins have characteristic configuration of His-Glu-Arg amino acid at the positions 5, 9, and 13, which are predicted to constitute for the interface of the contact with DNA molecule (Heim et al., 2003). These conserved positions were also found in the protein encoded by *HvRhl1* (HORVU7Hr1G030250) gene (**Figure 5B**). Moreover, a search for other protein motifs within HvRhl1 sequence allowed to distinguish a highly conserved region marked as a LRL protein domain (**Figure 5B**). Additionally, the phylogenetic analysis of HvRhl1 protein sequence showed that it probably belongs to the bHLH proteins from subfamily XI according to Heim et al. (2003) or a subfamily 17, based on the work of Toledo-Ortiz et al. (2003), as it forms a sub-cluster of a high similarity to Arabidopsis proteins from this group (**Supplementary Figures S4, S5**). Additionally, the analysis of the promoter sequence of *HvRhl1* gene allowed to find a motif of the L1 box-like sequence 5′-TAAATGT-3′ located at the region of 332 bp before exon 1 (**Figure 5B**), which is known to be recognized by the GLABRA2 (GL2) transcription factor (Lin et al., 2015).

Because our candidate gene showed significant similarity to the rice sequence *Osrhl1* (Ding et al., 2009) and *Lotus japonicus Ljrhl1* gene (Karas et al., 2009), both encoding proteins from bHLH subfamily XI, we had analyzed the conservation between genomic region around *HvRhl1* gene and the corresponding regions of its orthologs in *O. sativa* and *L. japonicus*. The order of five out of seven most similar homologs from the *HvRhl1* and *Osrhl1* region was the same (**Table 6** and **Supplementary Figure S6**) indicating high conservation and consequently microsynteny between these regions in barley and rice. No similar syntenic relationship could be found between *H. vulgare* and *L. japonicus*. Nevertheless, the sequence similarity, phylogenetic tree, and microsynteny of the barley and rice regions indicate that *HvRhl1* (HORVU7Hr1G030250) may be the true ortholog of *OsRhl1* gene.

**Table 6:** The indication of similarity between barley genes from the *HvRhl1* gene region and their homologs from rice syntenic region.

MLOC ID	HORVU ID	*Oryza sativa* Japonica Group ID	Sequence similarity	E-value
MLOC_4840	HORVU7Hr1G030210	*n/a – The most similar homolog out of syntenic region*	n/a	n/a
MLOC_39064	HORVU7Hr1G030220	LOC4340324	0.90%	7E-143
*HvRhl1* (MLOC_38567)	HORVU7Hr1G030250	*Osrhl1* (LOC107275733)	0.82%	4E-102
MLOC_17531	HORVU7Hr1G030270	LOC4340325	0.89%	2E-60
MLOC_36656	HORVU7Hr1G030280	*n/a – The most similar homolog out of syntenic region*	n/a	n/a
MLOC_75365	HORVU7Hr1G030290	Os06g0184200	0.69%	4E-31
MLOC_35776	HORVU7Hr1G030300	LOC4340327	0.96%	0.0

*The genes are ordered according to their position in the map interval of *HvRhl1* locus.*

**FIGURE 7:**
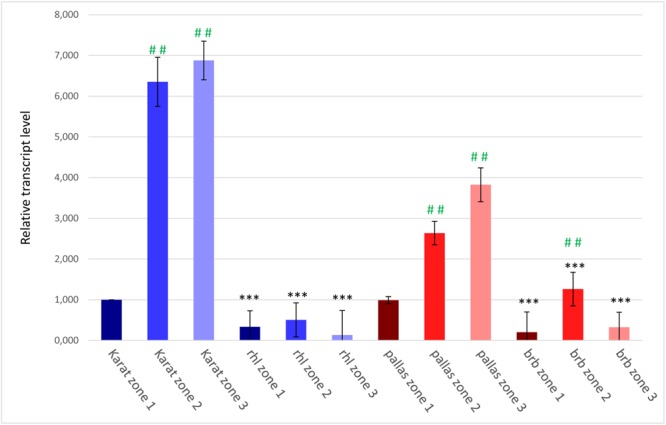
The expression levels of the candidate *HvRhl1* gene in three zones of the roots of ‘Karat’ and ‘Pallas’ varieties and *rhl1.b* and *brb* mutants. The fold change of gene expression for each root zone of the specific genotype (‘Pallas’, ‘Karat’, *rhl1.b, brb*) was normalized to the gene expression level characteristic for ‘Karat’ root apical zone (zone 1), which was considered as the value of 1. Black asterisks indicate the significant differences between the mutants and their parents regarding the corresponding root zones. Green hashes indicate the significant differences between zone 1 and either zones 2 or 3 within the single genotype. ^∗∗∗^*P* ≤ 0.001; ^∗∗^*P* ≤ 0.01; ^∗^*P* > 0.05. ^###^*P* ≤ 0.001; ^##^*P* ≤ 0.01; ^#^*P* > 0.05. The analysis was performed using three biological replicates.

To confirm the involvement of the candidate *HvRhl1* gene in root hair formation, its expression was analyzed in three 1 mm root segments of WT cv. ‘Karat’ and *rhl1.b* mutant: (1) root apical zone – a zone with meristematic activity, (2) elongation and differentiation zone – where newly formed cells undergo growth and the emerging root hairs initials are visible and (3) root hair zone – where tip growing root hairs are present (**Supplementary Figure S1**). In the cv. ‘Karat’ the lowest expression level of *HvRhl1* gene was observed in the root apical zone. It increased significantly (*P* ≤ 0.001) in the elongation and differentiation zone and remained on similarly high level in the root hair zone (**Figure 7**). This observation indicates that there is a relationship between the expression of the candidate gene and the formation of root hairs in the wild type cultivar. In contrast, the expression level of *HvRhl1* gene in *rhl1.b* mutant was 3 times, 12 times, and 50 times lower in the zones 1, 2, and 3, respectively, compared to the corresponding zones of cv. ‘Karat’. Moreover, when the expression of the candidate gene was compared solely between the three root zones of *rhl1.b* mutant, no statistical differences were noticed between them (*P* > 0.05; **Figure 7**).

### The Analysis of the Candidate HORVU7Hr1G030250 Gene in the Root Hairless *brb* Mutant

Our previous tests showed that *rhl1.b* mutant was allelic to the spontaneous root hairless mutant *brb* (*bald root barley*) found in cv. ‘Pallas’ (Gahoonia et al., 2001; Chmielewska et al., 2014). Therefore, we analyzed the genomic sequence of HORVU7Hr1G030250 gene also in these two genotypes. No mutations were found, however, between *brb* mutant and cv. ‘Pallas’ in any of the exons or introns of the gene. Thus, we extended the examined sequence to 4,474 bp upstream and 1,882 bp downstream from the CDS region of the gene, but again their analysis did not show any differences between *brb* and ‘Pallas’ (**Supplementary Figure S7**).

The expression level of the candidate HORVU7Hr1G030250 gene was also analyzed in the subsequent root zones of both genotypes. In cv. ‘Pallas’, a similar expression pattern as in cv. ‘Karat’ was observed, although the level of expression was generally lower in ‘Pallas’ (**Figure 7**). In the *brb* mutant, the expression of the candidate gene was much lower than in its WT parent, just as it was in *rhl1.b* compared to ‘Karat’. The expression of HORVU7Hr1G030250 in the root apical zone and the root hair zone was at the same level in *brb* and *rhl1.b* mutants. In the elongation zone of *brb* roots, however, a statistically significant (*P* ≤ 0.01) increase of the gene expression was observed, when compared to the root apical and differentiation zones (**Figure 7**).

As no mutations were identified in HORVU7Hr1G030250 gene in the *brb* mutant, four other HC genes from the target interval were sequenced in this genotype and compared to ‘Pallas’. Similarly to the HORVU7Hr1G030250 gene, the sequences of all of the remaining HC genes were identical between *brb* mutant and cv. ‘Pallas’ (**Supplementary Figure S7**). Such result suggests that some other gene or molecular mechanism is responsible for the lack of root hairs in the *brb* mutant.

Additionally, we selected six genes that were differentially expressed between *rhl1.a* mutant (a sister line of *rhl1.b*) and its WT parent in our previous microarray analyses (Kwasniewski et al., 2010, 2016) and we examined their expression profiles in the subsequent root zones of the *brb* mutant and ‘Pallas’ variety, together with *rhl1.b* and ‘Karat’. The genes encode cell wall-related proteins (**Supplementary Table S5**) that may probably act downstream of the *HvRhl1* gene. We found that their expression level was much lower in the mutants, compared to their respective parent varieties, and the expression profiles of all these genes in *brb* were very similar to the profiles of *rhl1.b* mutant (**Supplementary Figure S8**). It suggests that similar molecular pathways may be impaired in both mutants, which results in the changed rhizodermis patterning and the root hairless phenotype.

## Discussion

In our study, a barley mutant *rhl1.b* characterized by a lack of root hairs and undeveloped rhizodermal patterning was used to study the genetic basis of the first step of root hair development, i.e., formation of distinguishable tricho- and atrichoblasts. For this purpose a map-based cloning strategy was used to select a potential candidate gene responsible for the root hairless phenotype. We took advantage of the existence of barley genome sequence to omit the time-consuming process of sequencing the BAC clones. We also used this resource to develop markers for fine mapping of the region around the *rhl1* gene. We created F_2_ mapping population, which allowed to select an interval of 3.7 cM around *rhl1* gene, which translated to 577,263 bp of physical length. Only five genes were present within this region and the unique A>T mutation was identified in a gene sequence HORVU7Hr1G030250 encoding bHLH type transcription factor. The mutation resulted in the intron retention and a frameshift leading to the aberrant synthesis of 71 amino acids. Within the corresponding part of the wild type protein, the LRL (LOTUS JAPONICUS ROOTHAIRLESS1-LIKE) domain is located. Thus, the mutation in *rhl1.b* allele leads to the lack of the correct LRL domain, which may have a detrimental impact on the protein function. This finding, together with a lack of any additional mutation in the other coding sequences from this physical interval, makes the HORVU7Hr1G030250 the most probable candidate responsible for the lack of appropriate rhizodermis patterning, resulting in the root hairless phenotype of the mutant.

The possible involvement of this gene in root hair development may also be deduced from the studies of *Ljrhl1* (Karas et al., 2009) and *Osrhl1* root hair mutants (Ding et al., 2009) in *Lotus japonicus* and rice, respectively. In both cases, the mutant phenotypes were attributed to the mutations in genes encoding bHLH proteins, with a high sequence similarity to our barley candidate. It should be noted that only *Ljrhl1* mutant completely lacked root hairs, whereas rice *rhl1* mutant developed very short root hairs. What is important, however, the rhizodermis pattern in *Osrhl1* was disturbed, without clear formation of shorter and longer cells characteristic to the wild type rice (Ding et al., 2009). Additionally, our analysis showed the existence of the microsynteny encompassing the part of genomic region of barley *HvRhl1* candidate gene and a corresponding region on chromosome 6 of rice with *Osrhl1* locus. Thus, based on sequence similarity, phylogenetic tree, and synteny of the regions, *HvRhl1* is likely the true orthologous gene of *OsRhl1.* No synteny was found between analyzed region and *L. japonicus* genome, however such result may be a consequence of the too far evolutionary distance between *L. japonicus* and grasses.

The analysis of sequence structure of the protein encoded by HORVU7Hr1G030250 (*HvRhl1*) gene showed that it contains two conserved domains: a basic-helix-loop-helix (bHLH) and LRL domain. bHLH domain is involved in the contact with DNA, whereas other non-bHLH motifs, which are present in many members of bHLH family, are responsible for the specificity of the transcription factor toward the target genes (Heim et al., 2003). The function of the LRL domain was attributed to the transcriptional control of genes involved in root hair development (Karas et al., 2009; Bruex et al., 2012). The bHLH protein encoded by *HvRhl1* gene is the most similar to the bHLH subfamily XI of Arabidopsis, according to the classification of Heim et al. (2003), which corresponds to the subfamily 17 from the classification of Toledo-Ortiz et al. (2003). It is grouped on the phylogenetic tree in the same cluster as five Arabidopsis LRL proteins: AtLRL1, AtLRL2, AtLRL3, AtLRL4, and AtLRL5 (**Supplementary Figures S4, S5**) which are known to act as two antagonistic groups of regulators of root hairs growth. Our candidate falls into the sub-cluster with the first three of the above mentioned proteins (AtLRL1, AtLRL2, AtLRL3), which are the positive regulators of the root hair development in Arabidopsis. The Arabidopsis single mutants in LRL protein-encoding genes showed disorders of root hair formation (e.g., root hair shortening or branching), not related, however, to the rhizodermis patterning (Bruex et al., 2012; Breuninger et al., 2016). All single mutants developed root hairs of different length or morphology, while the double mutants *atlrl1-2, atlrl3-1* were only capable to initiate root hairs (Bruex et al., 2012; Breuninger et al., 2016). It is postulated that *AtLRL1, AtLRL2*, and *AtLRL3* genes have a partially redundant function in Arabidopsis and single mutations do not generate strong root hair phenotype (Karas et al., 2009). Interestingly, double mutants of the *Physcomitrella* homologs of *AtLRL1* and *AtLRL2* were completely rhizoidless, suggesting that LRL proteins evolved in the common ancestor of the mosses and angiosperms and control the development of tip-growing cells with rooting functions (Tam et al., 2015).

Taking into consideration the phenotypes of Arabidopsis and barley mutants, we may hypothesize that in Arabidopsis the proteins with LRL domain function in the processes of root hair tip growth and they prevent root hair branching, whereas in barley (and probably in rice and *L. japonicus*) they control the rhizodermis cells differentiation. Nevertheless, they may show some overlap in the control of similar downstream pathways in both species. Such a possibility is suggested by the transcriptome studies of Arabidopsis *atlrl1* (Bruex et al., 2012) and barley *rhl1.a*, a sibling *of rhl1.b* (Kwasniewski et al., 2010, 2016) mutants. The transcriptome analysis of root epidermal tissue of *atlrl1* mutant identified 10 differentially expressed genes in the mutant compared to the wild type. They included downregulated genes encoding cell wall related proteins, e.g., xyloglucan endotransglucosylase, and proteins with peroxidase activity. A similar set of genes was found to be downregulated in the roots of barley *rhl1.a* mutant, including cell wall-related genes, such as extensins, arabinogalactan proteins (AGPs), xyloglucan endotransglucosylases/hydrolases (XTHs) and pectinesterases, together with a probable barley homologs of Arabidopsis *RHD4* (*ROOT HAIR DEFECTIVE4*), *COW1* (*CAN OF WORMS1*), or *SHV3* (*SHAVEN 3*) genes (Kwasniewski et al., 2010, 2016). Additionally, a lack of the expression of *HvEXPB1* gene, encoding β-expansin 1, a cell wall-loosening protein, was noticed in the *rhl1.a* mutant (Kwasniewski and Szarejko, 2006). Accordingly, Bruex et al. (2012) postulated that *LRL* genes may be involved in the regulation of *COW1* and *EXP7* (*EXPANSIN A7*) expression in Arabidopsis.

The analysis of the promoter sequence of HORVU7Hr1G030250 gene showed that it contains a motif of L1 box-like sequence, 5′-TAAATGT-3′, localized 332 bp upstream of the initiation codon (**Figure 5B**). At present it is not known whether any barley homolog of GL2 acts upstream of *HvRhl1* gene nor if there is any other factor that may regulate the transcription of our HORVU7Hr1G030250 candidate. We have shown that in the wild type a significant increase of the candidate gene expression in the elongation and differentiation root zone occurs, i.e., in the zone, where the root hair development is initiated, comparing to the meristematic zone of the root. We have also observed that the expression of the candidate gene in the roots of root hairless mutant is very low compared to the wild type parent. Such finding suggests, that some regulatory mechanisms are involved in the expression reduction in *rhl1.b* mutant. It is possible that the mutated transcript, which contains a premature translation-termination codon is recognized by a nonsense-mediated decay mechanism targeting abnormal transcript to rapid degradation (Hug et al., 2016).

More light on the complexity of the mechanisms leading to the formation of rhizodermis pattern in barley may be shed by the analysis of another barley root hairless mutant, *brb*, which in our previous studies turned out to be allelic to the *rhl1.b*. Surprisingly, we did not find any changes between *brb* and its parent variety ‘Pallas’ in the sequence of *HvRhl1* candidate gene, both in the gene body and in a substantial upstream and downstream region. Similarly, no mutations were found in any other gene located in the target mapping interval. At present, we may only formulate several hypotheses explaining such a result. One of them is the possibility of an inactivation of *HvRhl1* gene in *brb* mutant due to a phenomenon of position-effect variegation (PEV), which was described and studied to the largest extent in *Drosophila melanogaster* (Elgin and Reuter, 2013). It is usually related to the situation where a gene, due to a chromosome rearrangement, is placed in a context of heterochromatin, which prevents its expression. Such an effect may also be observed when a transposable element is inserted in the close proximity of the gene in question, which again may have an inhibitory effect caused by epigenetic regulation. An example of such a phenomenon was found in a study of a fruit fly (*Drosophila melanogaster*) *ald* gene, responsible for defects in meiosis and mitosis (Gilliland et al., 2005). Two genotypes that seemed to be allelic to other *ald* mutants did not exhibit any mutation in *ald* gene sequence. Instead, a *Doc* transposable element was found to be inserted downstream of *ald* locus, at a distance of around 2,000 bp. This transposable element suppressed the function of nearby *ald* locus in germline cells (Gilliland et al., 2005). It is possible that in the spontaneous barley *brb* mutant a similar event of a transposition has taken place, considerably reducing the expression of *HvRhl1* gene to the significantly suboptimal level, which is too low to trigger the transcription of its downstream genes and, despite the lack of any changes in the gene body and regulatory sequences, it results in the root hairless phenotype of the mutant. It is known that transposon movement is silenced by the epigenetic modification of chromatin and such modifications may span a large part of the chromosome, beyond the transposon itself, what may create the context of heterochromatin around neighboring genes (Zhang et al., 2008). This hypothesis may be supported by the results of expression analysis of six genes examined in *brb, rhl1.b* and their respective parents in our studies. All these genes, which may be considered as putative direct or indirect targets of the candidate HORVU7Hr1G030250 (*HvRhl1*), showed the much lower expression level in both mutants, compared to the wild-type parents. Moreover, their expression profiles in the subsequent root zones of *brb* were very similar to the profiles of *rhl1.b* mutant. Thus, the hypothesis of the silencing of our candidate gene by heterochromatin formation in *brb* mutant, that leads to the mimicry of the mutation in *rhl1.b* genotype may be plausible. It would also indicate that two different molecular mechanisms may be responsible for the same downstream effect in these genotypes.

In some cases, the recessive mutations in two genes may also create a mutant phenotype in a double heterozygote, which results in a false outcome of a complementation test. Such phenomenon is known as a second-site non-complementation (SSNC; reviewed in Hawley and Gilliland, 2006). It may include several molecular mechanisms, but the most frequent one is attributed to the combined haplo-insufficiency, where the simultaneous reduction of a dosage of both gene products creates a mutated phenotype (Hawley and Gilliland, 2006). Taking into consideration that *HvRhl1* gene encodes a transcription factor which may regulate a large number of target genes and, at the same time, it itself is regulated by other TFs, it is possible that the combined haplo-insufficiency may be the answer for the false result of *rhl1.b* and *brb* complementation test. We have to assume, however, that the second hypothetical locus should be located at a very close proximity to our candidate gene, preventing recombination, because we did not found any recombinants in the F_2_ generation of *rhl1.b* × *brb* cross, consisting of 200 individuals.

The last hypothesis, although of the lowest probability, is based on two assumptions: (1) that *rhl1.b* mutant, which is a product of chemical mutagenesis, has not one, but two mutations, which may be located in the close proximity to each other, and (2) the yet not discovered mutation is located in a gene allelic to the *brb* locus. In such a scenario we assume that the *brb* gene acts upstream of *HvRhl1*, it carries a loss-of-function mutation and the distance between these two loci is short enough to prevent recombination events. The last assumption is due to the fact that in a cross of *rhl1.b* with the wild type parent, a 3:1 segregation was observed, indicating the single locus mutation in *rhl1.b* genotype. In the case of a strong recombination cold-spot around our candidate gene and the second hypothetical locus, we might not notice any recombinants due to the linkage drag of both loci. In the interval of our study, about 322 kb out of 577 kb sequence in the HORVU7Hr1G030250 gene region was of unknown function. Thus, we cannot rule out that within this region more genes are located and one of them is affected by a mutation in both *brb* and *rhl1.b* mutants.

## Conclusion

To conclude, our analysis proves the usefulness of a map-based cloning approach to identify the mutation underlying a phenotypic change in the mutant. We analyzed a barley mutant with changed root epidermis pattern and, consequently, a lack of root hairs – the important structure of root architecture that influences the plant performance under limited availability of water and nutrients in the soil. Our study shows that HORVU7Hr1G030250 gene encoding a bHLH transcription factor with LRL domain is the best putative candidate responsible for rhizodermis patterning and initiation of root hairs in barley. The splice-junction mutation identified in the *rhl1.b* mutant results in the retention of the last intron, leading to the frameshift mutation and aberrant synthesis of 71 amino acids in the mutated protein. As a result, the protein lacks the correct sequence of LRL domain involved in the transcriptional regulation of root hair related genes, which is present in the wild type protein. Based on our genetic analysis we presume that this mutation may be responsible for the lack of root hairs in *rhl1.b* mutant, although the functional complementation of the mutant will be necessary to directly link the mutation to the mutant phenotype. In the other root-hairless *brb* form, however, no changes were detected in the candidate HORVU7Hr1G030250 sequence. Therefore, it seems that the molecular mechanism of rhizodermis differentiation in barley may be more complex. Further studies of *brb* and other root hair mutants with changed rhizodermis pattern should reveal other possible components or mechanisms of the regulatory network involved in determination of rhizodermis cell fate.

## Author Contributions

IS and AJ conceived the study and designed the experiments. PG, PK, and AJ developed the mapping population. PG selected new markers, conducted fine mapping, analyzed the sequence content of physical interval, sequenced candidate loci, identified the mutated gene, and analyzed its expression. MK contributed to the analysis of barely, rice, and *L. japonicus* bHLH proteins. AJ performed the phylogenetic analysis of bHLH proteins. PG, IS, and AJ interpreted the data. PG and AJ wrote the manuscript. IS contributed to development of rhl1 mutants and participated in writing the manuscript. All authors discussed the results and commented on the manuscript.

## Conflict of Interest Statement

The authors declare that the research was conducted in the absence of any commercial or financial relationships that could be construed as a potential conflict of interest.

## Footnotes

1 http://plants.ensembl.org

2 http://bioinfo.ut.ee/primer3-0.4.0

3 www.statsoft.de

4 http://www.repeatmasker.org

5 http://www.repeatmasker.org/RMBlast.html

6 http://tandem.bu.edu/trf/trf.html

7 http://www.girinst.org/repbase/

8 http://blast.ncbi.nlm.nih.gov/Blast.cgi

9 https://lotus.au.dk/

10 http://genes.mit.edu/GENSCAN.html

11 www.softberry.com

12 http://www.ebi.ac.uk/InterProScan/

13 http://tcoffee.crg.cat/apps/tcoffee/index.html

14 http://doua.prabi.fr/software/seaview

15 https://www.ebi.ac.uk/Tools/msa/clustalo

## Supplementary Material

FIGURE S1 Root zones that were used for RNA extraction and the subsequent expression analysis of the HORVU7Hr1G030250 candidate gene.

FIGURE S2 The alignments of four HC gene sequences (HORVU7Hr1G030220, HORVU7Hr1G030270, HORVU7Hr1G030280, and HORVU7Hr1G030290) between *rhl1.b* mutant and its parent variety ‘Karat’.

FIGURE S3 The alignments of genomic and protein sequences of HORVU7Hr1G030250 candidate gene between *rhl1.b* mutant and its parent variety ‘Karat’.

FIGURE S4 The maximum likelihood phylogenetic tree of Arabidopsis, barley, rice, and *L. japonicus* bHLH protein family, including the HvRhl1 candidate protein. Support values written on the branches: SH-aLRT support (%)/ultrafast bootstrap support (%). The highly reliable clades are characterized by SH-aLRT ≥ 80% and UFboot ≥ 95% values. The numbers located to the right from the protein accessions indicate the membership of particular Arabidopsis protein to a specific subfamily according to Heim et al. (2003) (green) or Toledo-Ortiz et al. (2003) (red). At, *Arabidopsis thaliana*; Os, *Oryza sativa*; Hv, *Hordeum vulgare*; Lt, *Lotus japonicus*.

FIGURE S5 The close-up view of the phylogenetic tree showing HvRhl1 protein and the most closely related proteins from other specie.

FIGURE S6 The fragment of the *Oryza sativa* Japonica Group chromosome 6 with five genes homologous to genes from the *HvRhl1* region in barley. The order of corresponding homologs indicates the microsyntheny within *HvRhl1* region in barley and the region of its ortholog *Osrhl1* in rice.

FIGURE S7 The alignments of five HC gene sequences (HORVU7Hr1G030220, HORVU7Hr1G030250, HORVU7Hr1G030270, HORVU7Hr1G030280, and HORVU7Hr1G030290) between *brb* mutant and its parent variety ‘Pallas’.

FIGURE S8 The expression profiles of selected genes characterized by differential expression between *rhl1.a* mutant and ‘Karat’ variety, as previously shown by Kwasniewski et al. (2010, 2016), analyzed in the subsequent root zones of ‘Pallas’, ‘Karat’, *rhl1.b*, and *brb* genotypes. The relative transcript level for each root zone of the specific genotype was normalized to ‘Karat’ root apical zone, which was considered as the value of 1. Black asterisks indicate the significant differences between the mutants and their parents regarding the corresponding root zones. Green hashes indicate the significant differences between zone 1 and either zones 2 or 3 within the single genotype. ^∗∗∗^*P* ≤ 0.001; ^∗∗^*P* ≤ 0.01; ^∗^*P* > 0.05. ^###^*P* ≤ 0.001; ^##^*P* ≤ 0.01; ^#^*P* > 0.05. The analysis was performed using three biological replicates.

TABLE S1 The list of markers that were used in the fine mapping of *rhl1* gene, their genetic and physical position and the genotyping methods.

TABLE S2 Primer sequences that were used to amplify fragments of marker sequences subjected to the fine mapping of *rhl1* gene region and to amplify the fragment of the candidate gene encompassing the mutation between *rhl1.b* mutant and its parent variety ‘Karat’.

TABLE S3 Primer sequences that were used to amplify the whole exon-intron structure and the regions of up- and downstream parts of candidate genes from the physical interval around *rhl1* locus.

TABLE S4 The list of accession numbers for the Arabidopsis bHLH family proteins, together with their classification into subfamilies according to Heim et al. (2003) and Toledo-Ortiz et al. (2003) and the accessions of barley, rice, and *Lotus japonicus* bHLH proteins that were used to construct the phylogenetic tree.

TABLE S5 The list of genes subjected to qPCR analysis together with primer sequences used for their amplification.
