# Supplementary figures and images for: Forward Genetics Approach Reveals a Mutation in bHLH Transcription Factor-Encoding Gene as the Best Candidate for the Root Hairless Phenotype in Barley
Source: Front Plant Sci. 2018 Sep 3;9:1229. doi: 10.3389/fpls.2018.01229 (PMC6129617; doi:10.3389/fpls.2018.01229)

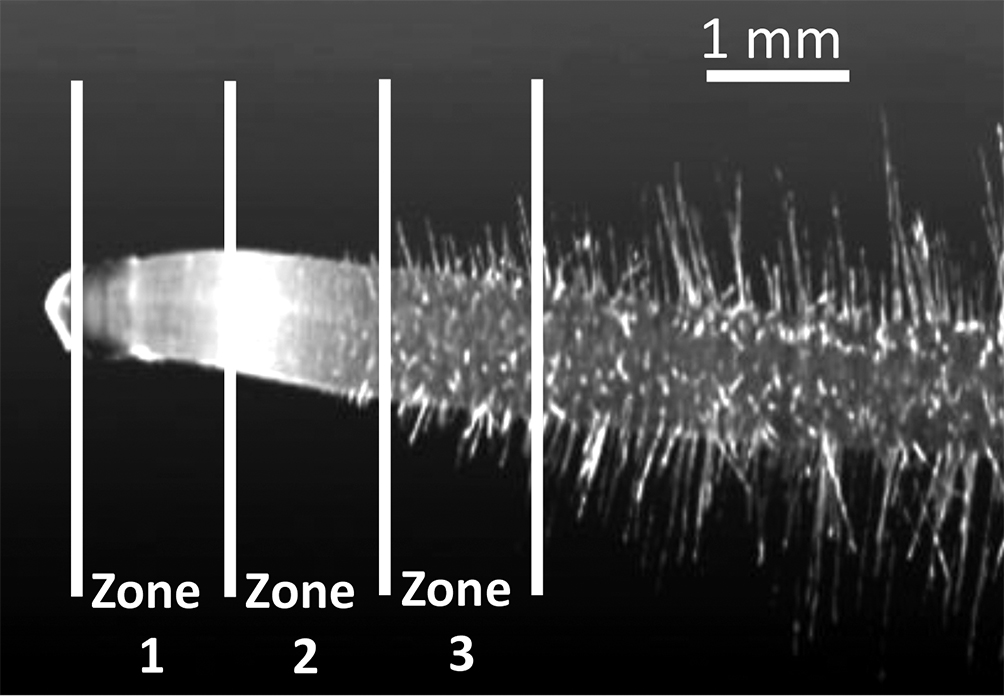

Supplement: FIGURE S1 — Root zones that were used for RNA extraction and the subsequent expression analysis of the HORVU7Hr1G030250 candidate gene. [file Image_1.JPEG]

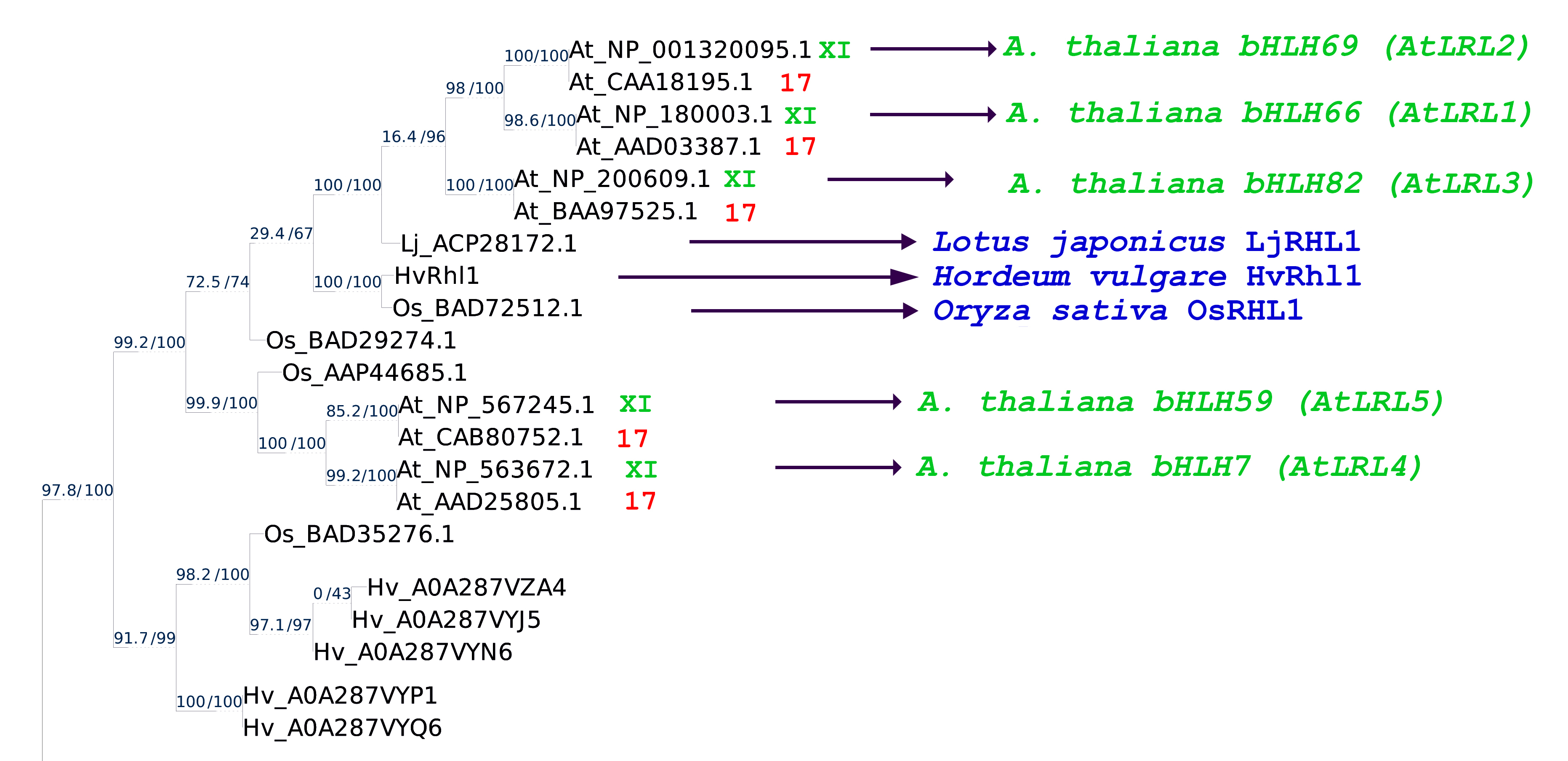

Supplement: FIGURE S5 — The close-up view of the phylogenetic tree showing HvRhl1 protein and the most closely related proteins from other specie. [file Image_5.JPEG]

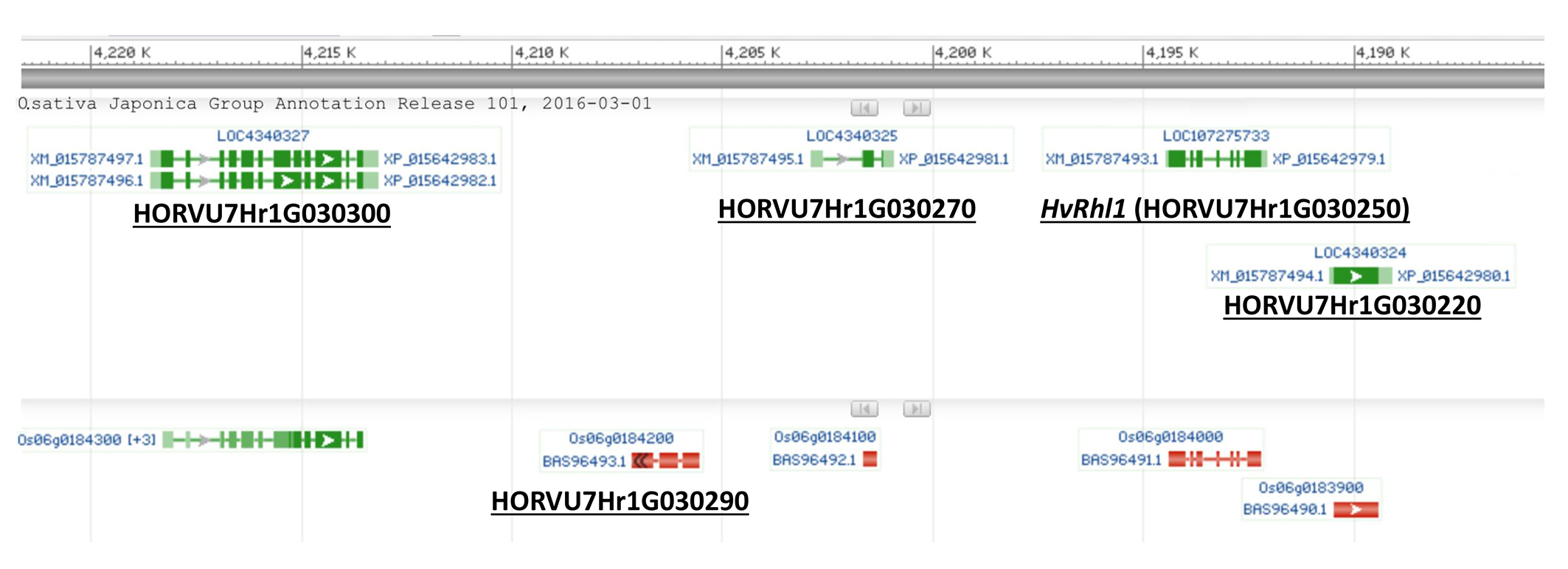

Supplement: FIGURE S6 — The fragment of the Oryza sativa Japonica Group chromosome 6 with five genes homologous to genes from the HvRhl1 region in barley. The order of corresponding homologs indicates the microsyntheny within HvRhl1 region in barley and the region of its ortholog Osrhl1 in rice. [file Image_6.JPEG]

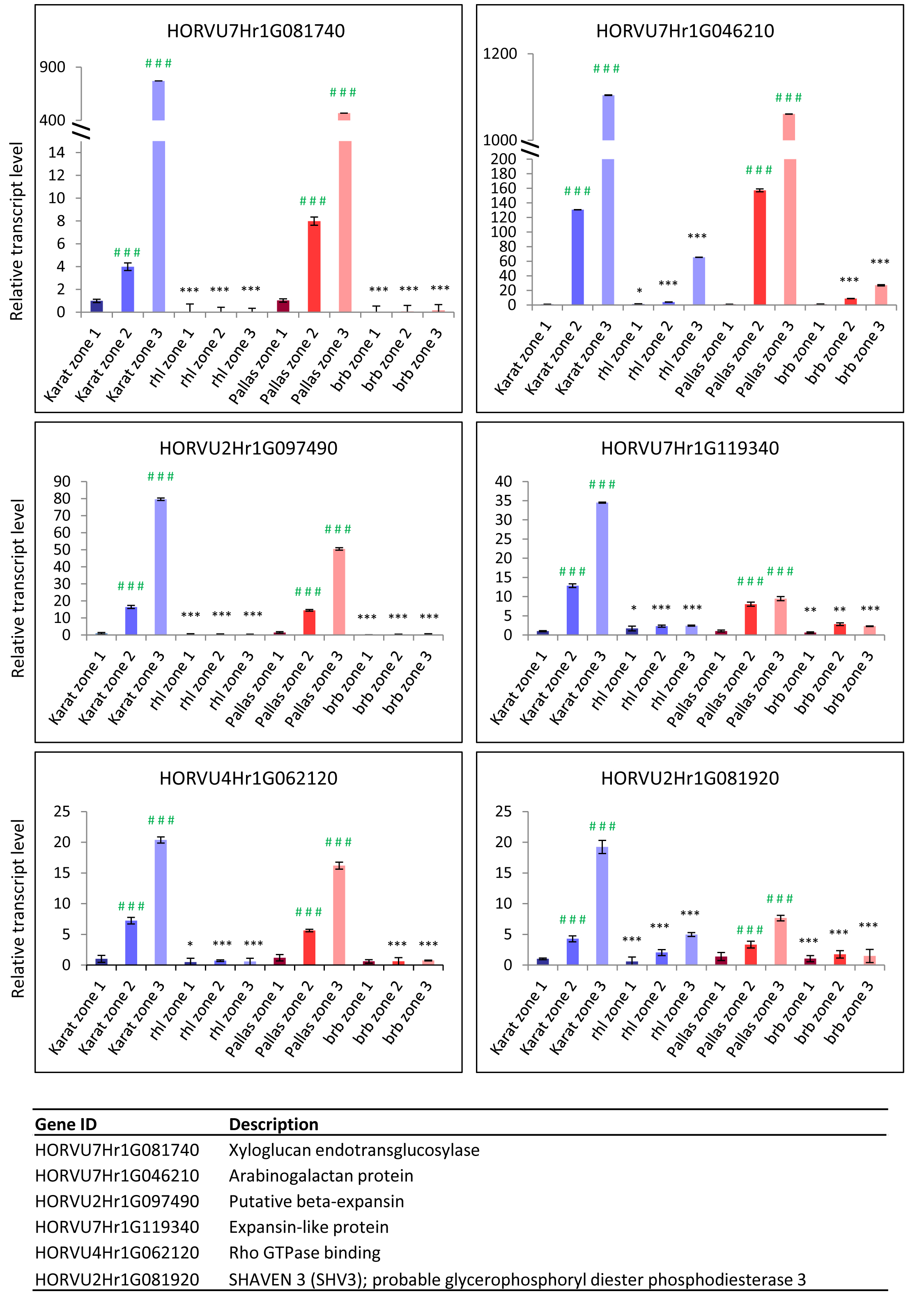

Supplement: FIGURE S8 — The expression profiles of selected genes characterized by differential expression between rhl1.a mutant and ‘Karat’ variety, as previously shown by Kwasniewski et al. (2010, 2016), analyzed in the subsequent root zones of ‘Pallas’, ‘Karat’, rhl1.b, and brb genotypes. The relative transcript level for each root zone of the specific genotype was normalized to ‘Karat’ root apical zone, which was considered as the value of 1. Black asterisks indicate the significant differences between the mutants and their parents regarding the corresponding root zones. Green hashes indicate the significant differences between zone 1 and either zones 2 or 3 within the single genotype. ∗∗∗P ≤ 0.001; ∗∗P ≤ 0.01; ∗P > 0.05. ###P ≤ 0.001; ##P ≤ 0.01; #P > 0.05. The analysis was performed using three biological replicates. [file Image_8.jpeg]
