# Supplementary material for: Forward Genetics Approach Reveals a Mutation in bHLH Transcription Factor-Encoding Gene as the Best Candidate for the Root Hairless Phenotype in Barley
Source: Front Plant Sci. 2018 Sep 3;9:1229. doi: 10.3389/fpls.2018.01229 (PMC6129617; doi:10.3389/fpls.2018.01229)
Supplement: FIGURE S2 — The alignments of four HC gene sequences (HORVU7Hr1G030220, HORVU7Hr1G030270, HORVU7Hr1G030280, and HORVU7Hr1G030290) between rhl1.b mutant and its parent variety ‘Karat’. [file Image_2.PDF]

Alignment_HORVU7Hr1G030220

```
Karat_F     CGACTGCTACCTCTCCATGTACCTCGCGAAGAAGCCCGACAAGGCCGACCCCAACGAGGC
Karat_R     CGACTGCTACCTCTCCATGTACCTCGCGAAGAAGCCCGACAAGGCCGACCCCAACGAGGC
Rhl_F       CGACTGCTACCTCTCCATGTACCTCGCGAAGAAGCCCGACAAGGCCGACCCCAACGAGGC
Rhl_R       CGACTGCTACCTCTCCATGTACCTCGCGAAGAAGCCCGACAAGGCCGACCCCAACGAGGC
            ************************************************************

Karat_F     CCGCCTCTCCCGCTTCACCTGCTGCGCGCTCTCGGGGGAGCCAATTGCCCCGCCCGCCGT
Karat_R     CCGCCTCTCCCGCTTCACCTGCTGCGCGCTCTCGGGGGAGCCAATTGCCCCGCCCGCCGT
Rhl_F       CCGCCTCTCCCGCTTCACCTGCTGCGCGCTCTCGGGGGAGCCAATTGCCCCGCCCGCCGT
Rhl_R       CCGCCTCTCCCGCTTCACCTGCTGCGCGCTCTCGGGGGAGCCAATTGCCCCGCCCGCCGT
            ************************************************************

Karat_F     CGTGGATCGGCTGGGCAACCTGTTCAACAAGGAGGCCCTCGTCGAGGCGCTCATCCACAA
Karat_R     CGTGGATCGGCTGGGCAACCTGTTCAACAAGGAGGCCCTCGTCGAGGCGCTCATCCACAA
Rhl_F       CGTGGATCGGCTGGGCAACCTGTTCAACAAGGAGGCCCTCGTCGAGGCGCTCATCCACAA
Rhl_R       CGTGGATCGGCTGGGCAACCTGTTCAACAAGGAGGCCCTCGTCGAGGCGCTCATCCACAA
            ************************************************************

Karat_F     GCGCCTGCCCAAGGCGCTCTCGCACATCCGCGGGCTCAAGGACATGATCCCCATCCACCT
Karat_R     GCGCCTGCCCAAGGCGCTCTCGCACATCCGCGGGCTCAAGGACATGATCCCCATCCACCT
Rhl_F       GCGCCTGCCCAAGGCGCTCTCGCACATCCGCGGGCTCAAGGACATGATCCCCATCCACCT
Rhl_R       GCGCCTGCCCAAGGCGCTCTCGCACATCCGCGGGCTCAAGGACATGATCCCCATCCACCT
            ************************************************************

Karat_F     GCACCCCAAGCCCAACGCGGCGGACCAGGAGGTCCGTTTCCAGTGCCCGGTCACCGGGTT
Karat_R     GCACCCCAAGCCCAACGCGGCGGACCAGGAGGTCCGTTTCCAGTGCCCGGTCACCGGGTT
Rhl_F       GCACCCCAAGCCCAACGCGGCGGACCAGGAGGTCCGTTTCCAGTGCCCGGTCACCGGGTT
Rhl_R       GCACCCCAAGCCCAACGCGGCGGACCAGGAGGTCCGTTTCCAGTGCCCGGTCACCGGGTT
            ************************************************************

Karat_F     TGAGTTCAACGGCAAGTCCCAGTTCCTTGCCCTCCGTGGATGCGGCCACGTGCTGAGCGT
Karat_R     TGAGTTCAACGGCAAGTCCCAGTTCCTTGCCCTCCGTGGATGCGGCCACGTGCTGAGCGT
Rhl_F       TGAGTTCAACGGCAAGTCCCAGTTCCTTGCCCTCCGTGGATGCGGCCACGTGCTGAGCGT
Rhl_R       TGAGTTCAACGGCAAGTCCCAGTTCCTTGCCCTCCGTGGATGCGGCCACGTGCTGAGCGT
            ************************************************************

Karat_F     GAAGGCTCTCAAGGAGGTGAAGTCGTCCTCGTGTTTGGTCTGCCATAAGGAGTTCGTTGA
Karat_R     GAAGGCTCTCAAGGAGGTGAAGTCGTCCTCGTGTTTGGTCTGCCATAAGGAGTTCGTTGA
Rhl_F       GAAGGCTCTCAAGGAGGTGAAGTCGTCCTCGTGTTTGGTCTGCCATAAGGAGTTCGTTGA
Rhl_R       GAAGGCTCTCAAGGAGGTGAAGTCGTCCTCGTGTTTGGTCTGCCATAAGGAGTTCGTTGA
            ************************************************************

Karat_F     GGTGGACAAGATGCCCATCAATGGGACCGAGGAGGAGGTGGAAGTGCTGAGGCAGAGGAT
Karat_R     GGTGGACAAGATGCCCATCAATGGGACCGAGGAGGAGGTGGAAGTGCTGAGGCAGAGGAT
Rhl_F       GGTGGACAAGATGCCCATCAATGGGACCGAGGAGGAGGTGGAAGTGCTGAGGCAGAGGAT
Rhl_R       GGTGGACAAGATGCCCATCAATGGGACCGAGGAGGAGGTGGAAGTGCTGAGGCAGAGGAT
            ************************************************************

Karat_F     GGAGGAGGAGAGAGGGAAGCTGAAGGAGAAGAAGGATAAGAAGCTGTCGAATGGGCTCAG
Karat_R     GGAGGAGGAGAGAGGGAAGCTGAAGGAGAAGAAGGATAAGAAGCTGTCGAATGGGCTCAG
Rhl_F       GGAGGAGGAGAGAGGGAAGCTGAAGGAGAAGAAGGATAAGAAGCTGTCGAATGGGCTCAG
Rhl_R       GGAGGAGGAGAGAGGGAAGCTGAAGGAGAAGAAGGATAAGAAGCTGTCGAATGGGCTCAG
            ************************************************************

Karat_F     TGGGAGTAAGCATGCTGCTGCTGCTTCTGCGGTTGCAGACGCTGAGAAGTTGGAGAATGG
Karat_R     TGGGAGTAAGCATGCTGCTGCTGCTTCTGCGGTTGCAGACGCTGAGAAGTTGGAGAATGG
Rhl_F       TGGGAGTAAGCATGCTGCTGCTGCTTCTGCGGTTGCAGACGCTGAGAAGTTGGAGAATGG
Rhl_R       TGGGAGTAAGCATGCTGCTGCTGCTTCTGCGGTTGCAGACGCTGAGAAGTTGGAGAATGG
            ************************************************************

Karat_F     GAAGAAAGGGGAGGCTGCCCCAGCAAAGCGGTTTAAGGCTGCAGATCATGCACCGGCTCA
Karat_R     GAAGAAAGGGGAGGCTGCCCCAGCAAAGCGGTTTAAGGCTGCAGATCATGCACCGGCTCA
Rhl_F       GAAGAAAGGGGAGGCTGCCCCAGCAAAGCGGTTTAAGGCTGCAGATCATGCACCGGCTCA
Rhl_R       GAAGAAAGGGGAGGCTGCCCCAGCAAAGCGGTTTAAGGCTGCAGATCATGCACCGGCTCA
            ************************************************************
```

```
Karat_F         TGCAAACAAGAAAGTGTATGCATCAATTTTCACTTCCTCCAACAAGTCTGATTTCAGGGA
Karat_R         TGCAAACAAGAAAGTGTATGCATCAATTTTCACTTCCTCCAACAAGTCTGATTTCAGGGA
Rhl_F           TGCAAACAAGAAAGTGTATGCATCAATTTTCACTTCCTCCAACAAGTCTGATTTCAGGGA
Rhl_R           TGCAAACAAGAAAGTGTATGCATCAATTTTCACTTCCTCCAACAAGTCTGATTTCAGGGA
                ************************************************************

Karat_F         AACATACTCATGCCGGTCACTCCCCCTGGGAAGGAATTAAATCGATGTTGGGGAGATTCC
Karat_R         AACATACTCATGCCGGTCACTCCCCCTGGGAAGGAATTAAATCGATGTTGGGGAGATTCC
Rhl_F           AACATACTCATGCCGGTCACTCCCCCTGGGAAGGAATTAAATCGATGTTGGGGAGATTCC
Rhl_R           AACATACTCATGCCGGTCACTCCCCCTGGGAAGGAATTAAATCGATGTTGGGGAGATTCC
                ************************************************************

Karat_F         AATGGCATGGAGGTGAACCTCCTGTACTGATTCAGCGATTAGCTTCATACCATGGCTTGC
Karat_R         AATGGCATGGAGGTGAACCTCCTGTACTGATTCAGCGATTAGCTTCATACCATGGCTTGC
Rhl_F           AATGGCATGGAGGTGAACCTCCTGTACTGATTCAGCGATTAGCTTCATACCATGGCTTGC
Rhl_R           AATGGCATGGAGGTGAACCTCCTGTACTGATTCAGCGATTAGCTTCATACCATGGCTTGC
                ************************************************************

Karat_F         CCATAAGTTCCAGTATTCTTGTTTTAGTTGTTGGTGTTATCTATGCTTGTTCACTCACAG
Karat_R         CCATAAGTTCCAGTATTCTTGTTTTAGTTGTTGGTGTTATCTATGCTTGTTCACTCACAG
Rhl_F           CCATAAGTTCCAGTATTCTTGTTTTAGTTGTTGGTGTTATCTATGCTTGTTCACTCACAG
Rhl_R           CCATAAGTTCCAGTATTCTTGTTTTAGTTGTTGGTGTTATCTATGCTTGTTCACTCACAG
                ************************************************************

Karat_F         AGAAGCTATATGTTTTTATAAATGATTTGTGAAATATGTAATGGTTGAAACATTTGGCAC
Karat_R         AGAAGCTATATGTTTTTATAAATGATTTGTGAAATATGTAATGGTTGAAACATTTGGCAC
Rhl_F           AGAAGCTATATGTTTTTATAAATGATTTGTGAAATATGTAATGGTTGAAACATTTGGCAC
Rhl_R           AGAAGCTATATGTTTTTATAAATGATTTGTGAAATATGTAATGGTTGAAACATTTGGCAC
                ************************************************************

Karat_F         ATATCATTTGGTCCAGTCTTCTCTAGCAATTTGTCTGAGTAATGCTGTAGTTTTCACTCA
Karat_R         ATATCATTTGGTCCAGTCTTCTCTAGCAATTTGTCTGAGTAATGCTGTAGTTTTCACTCA
Rhl_F           ATATCATTTGGTCCAGTCTTCTCTAGCAATTTGTCTGAGTAATGCTGTAGTTTTCACTCA
Rhl_R           ATATCATTTGGTCCAGTCTTCTCTAGCAATTTGTCTGAGTAATGCTGTAGTTTTCACTCA
                ************************************************************

Karat_F         AAGAGAAGCAATATGTTTTTATAAATGCTTTGTGAAACATGTAATGGTTGAAACAGTTAG
Karat_R         AAGAGAAGCAATATGTTTTTATAAATGCTTTGTGAAACATGTAATGGTTGAAACAGTTAG
Rhl_F           AAGAGAAGCAATATGTTTTTATAAATGCTTTGTGAAACATGTAATGGTTGAAACAGTTAG
Rhl_R           AAGAGAAGCAATATGTTTTTATAAATGCTTTGTGAAACATGTAATGGTTGAAACAGTTAG
                ************************************************************

Karat_F         CGCTTATTGTTGGGTCCAGTCTTCTCTAGCAATTTGTCTGAGTAATGCTGTAATTTTCCA
Karat_R         CGCTTATTGTTGGGTCCAGTCTTCTCTAGCAATTTGTCTGAGTAATGCTGTAATTTTCCA
Rhl_F           CGCTTATTGTTGGGTCCAGTCTTCTCTAGCAATTTGTCTGAGTAATGCTGTAATTTTCCA
Rhl_R           CGCTTATTGTTGGGTCCAGTCTTCTCTAGCAATTTGTCTGAGTAATGCTGTAATTTTCCA
                ************************************************************

Karat_F         CTTTGTTTCAAATTTACTCACATCTTAGGTCTCGTCAGGCCAATCCTTTGACCAACAATT
Karat_R         CTTTGTTTCAAATTTACTCACATCTTAGGTCTCGTCAGGCCAATCCTTTGACCAACAATT
Rhl_F           CTTTGTTTCAAATTTACTCACATCTTAGGTCTCGTCAGGCCAATCCTTTGACCAACAATT
Rhl_R           CTTTGTTTCAAATTTACTCACATCTTAGGTCTCGTCAGGCCAATCCTTTGACCAACAATT
                ************************************************************

Karat_F         ATTCTATTAGTAGACCTAAGGGTTCTTAGTGTTCATTGTTGCAACTTCCACTGTATCTTG
Karat_R         ATTCTATTAGTAGACCTAAGGGTTCTTAGTGTTCATTGTTGCAACTTCCACTGTATCTTG
Rhl_F           ATTCTATTAGTAGACCTAAGGGTTCTTAGTGTTCATTGTTGCAACTTCCACTGTATCTTG
Rhl_R           ATTCTATTAGTAGACCTAAGGGTTCTTAGTGTTCATTGTTGCAACTTCCACTGTATCTTG
                ************************************************************

Karat_F         GTCATGCAATCCTACATACAATTTGTTTGAATAAGACTGATTTATGTGCACATGCAAGTG
Karat_R         GTCATGCAATCCTACATACAATTTGTTTGAATAAGACTGATTTATGTGCACATGCAAGTG
Rhl_F           GTCATGCAATCCTACATACAATTTGTTTGAATAAGACTGATTTATGTGCACATGCAAGTG
Rhl_R           GTCATGCAATCCTACATACAATTTGTTTGAATAAGACTGATTTATGTGCACATGCAAGTG
                ************************************************************

Karat_F         GACTCTCAAGATAAATCAAACAGTTTCTATGATTTTCAATGTTGTAATCTAGACTGTGCC
Karat_R         GACTCTCAAGATAAATCAAACAGTTTCTATGATTTTCAATGTTGTAATCTAGACTGTGCC
Rhl_F           GACTCTCAAGATAAATCAAACAGTTTCTATGATTTTCAATGTTGTAATCTAGACTGTGCC
```

```
Rhl_R           GACTCTCAAGATAAATCAAACAGTTTCTATGATTTTCAATGTTGTAATCTAGACTGTGCC
                ************************************************************

Karat_F         ACTTTACTGAACAGATAAATATGCATTGCGCTTGTTTTGATGTATAGTTGTCCATGAAGT
Karat_R         ACTTTACTGAACAGATAAATATGCATTGCGCTTGTTTTGATGTATAGTTGTCCATGAAGT
Rhl_F           ACTTTACTGAACAGATAAATATGCATTGCGCTTGTTTTGATGTATAGTTGTCCATGAAGT
Rhl_R           ACTTTACTGAACAGATAAATATGCATTGCGCTTGTTTTGATGTATAGTTGTCCATGAAGT
                ************************************************************

Karat_F         ACGGAATGCGCGTGAAGACAGTCAGCGGTAGAACATTTGGCGCCACATTAATACTAGCAT
Karat_R         ACGGAATGCGCGTGAAGACAGTCAGCGGTAGAACATTTGGCGCCACATTAATACTAGCAT
Rhl_F           ACGGAATGCGCGTGAAGACAGTCAGCGGTAGAACATTTGGCGCCACATTAATACTAGCAT
Rhl_R           ACGGAATGCGCGTGAAGACAGTCAGCGGTAGAACATTTGGCGCCACATTAATACTAGCAT
                ************************************************************

Karat_F         GGTAGTTGCTCTTTACATGCTTCTTTAGATATGCTGTCAAGCTAACCTTGGACATGGTGA
Karat_R         GGTAGTTGCTCTTTACATGCTTCTTTAGATATGCTGTCAAGCTAACCTTGGACATGGTGA
Rhl_F           GGTAGTTGCTCTTTACATGCTTCTTTAGATATGCTGTCAAGCTAACCTTGGACATGGTGA
Rhl_R           GGTAGTTGCTCTTTACATGCTTCTTTAGATATGCTGTCAAGCTAACCTTGGACATGGTGA
                ************************************************************

Karat_F         AAGTTTGCCTATTTTCTTTCAAACCTTCTCCTTTGGGCCTTTATTTCCATAACATGTTTA
Karat_R         AAGTTTGCCTATTTTCTTTCAAACCTTCTCCTTTGGGCCTTTATTTCCATAACATGTTTA
Rhl_F           AAGTTTGCCTATTTTCTTTCAAACCTTCTCCTTTGGGCCTTTATTTCCATAACATGTTTA
Rhl_R           AAGTTTGCCTATTTTCTTTCAAACCTTCTCCTTTGGGCCTTTATTTCCATAACATGTTTA
                ************************************************************

Karat_F         TAATGTACACATGTTATATTTCTCGTTATACCTTTGTCATTTGGGCTATACTATGAATTG
Karat_R         TAATGTACACATGTTATATTTCTCGTTATACCTTTGTCATTTGGGCTATACTATGAATTG
Rhl_F           TAATGTACACATGTTATATTTCTCGTTATACCTTTGTCATTTGGGCTATACTATGAATTG
Rhl_R           TAATGTACACATGTTATATTTCTCGTTATACCTTTGTCATTTGGGCTATACTATGAATTG
                ************************************************************

Karat_F         TAACGTACATACTTATGTAGCTTTTGCTGAATTTACATATTTGCTAATTGTGGATTTTAT
Karat_R         TAACGTACATACTTATGTAGCTTTTGCTGAATTTACATATTTGCTAATTGTGGATTTTAT
Rhl_F           TAACGTACATACTTATGTAGCTTTTGCTGAATTTACATATTTGCTAATTGTGGATTTTAT
Rhl_R           TAACGTACATACTTATGTAGCTTTTGCTGAATTTACATATTTGCTAATTGTGGATTTTAT
                ************************************************************

Karat_F         AAAAAGATCCAAAATAGCTATAATCAATCTACTTCTGCCCTGTATGCACATTGCTGAATA
Karat_R         AAAAAGATCCAAAATAGCTATAATCAATCTACTTCTGCCCTGTATGCACATTGCTGAATA
Rhl_F           AAAAAGATCCAAAATAGCTATAATCAATCTACTTCTGCCCTGTATGCACATTGCTGAATA
Rhl_R           AAAAAGATCCAAAATAGCTATAATCAATCTACTTCTGCCCTGTATGCACATTGCTGAATA
                ************************************************************

Karat_F         AGATTCTGCAAAGATTCATAGGATGCCATGAACTTACTGAGATATCAAGACTAGAGTTGA
Karat_R         AGATTCTGCAAAGATTCATAGGATGCCATGAACTTACTGAGATATCAAGACTAGAGTTGA
Rhl_F           AGATTCTGCAAAGATTCATAGGATGCCATGAACTTACTGAGATATCAAGACTAGAGTTGA
Rhl_R           AGATTCTGCAAAGATTCATAGGATGCCATGAACTTACTGAGATATCAAGACTAGAGTTGA
                ************************************************************

Karat_F         TTTGGACACACAGGAATTTGACACCTCAGTTGTCTAACTCTGTTCAAATTTAGAAGGAAT
Karat_R         TTTGGACACACAGGAATTTGACACCTCAGTTGTCTAACTCTGTTCAAATTTAGAAGGAAT
Rhl_F           TTTGGACACACAGGAATTTGACACCTCAGTTGTCTAACTCTGTTCAAATTTAGAAGGAAT
Rhl_R           TTTGGACACACAGGAATTTGACACCTCAGTTGTCTAACTCTGTTCAAATTTAGAAGGAAT
                ************************************************************

Karat_F         GGTGAAAATAGCTCCTATCAAGAAGACATCCAGATTTATTTTGCGTTAACATGCGGTACC
Karat_R         GGTGAAAATAGCTCCTATCAAGAAGACATCCAGATTTATTTTGCGTTAACATGCGGTACC
Rhl_F           GGTGAAAATAGCTCCTATCAAGAAGACATCCAGATTTATTTTGCGTTAACATGCGGTACC
Rhl_R           GGTGAAAATAGCTCCTATCAAGAAGACATCCAGATTTATTTTGCGTTAACATGCGGTACC
                ************************************************************

Karat_F         TCCCTCAGACTATTTGTAGTTGGACACACATTAGTTTGTTTTTCTTATTCCATACTTGCT
Karat_R         TCCCTCAGACTATTTGTAGTTGGACACACATTAGTTTGTTTTTCTTATTCCATACTTGCT
Rhl_F           TCCCTCAGACTATTTGTAGTTGGACACACATTAGTTTGTTTTTCTTATTCCATACTTGCT
Rhl_R           TCCCTCAGACTATTTGTAGTTGGACACACATTAGTTTGTTTTTCTTATTCCATACTTGCT
                ************************************************************
```

```
Karat_F      TTGAAGGGGAGCCGTGGCGCAGCGGCAAAGCTGTTGCCTTGTGACCATTA
Karat_R      TTGAAGGGGAGCCGTGGCGCAGCGGCAAAGCTGTTGCCTTGTGACCATTA
Rhl_F        TTGAAGGGGAGCCGTGGCGCAGCGGCAAAGCTGTTGCCTTGTGACCATTA
Rhl_R        TTGAAGGGGAGCCGTGGCGCAGCGGCAAAGCTGTTGCCTTGTGACCATTA
             **************************************************
```

Alignment_HORVU7Hr1G030270

```
Karat_F     CATTGTGGGTAGACCCAAATAAGTGTCTGACAATGCCTCAGTAACAATGTCAAGTTCTCT
Karat_R     CATTGTGGGTAGACCCAAATAAGTGTCTGACAATGCCTCAGTAACAATGTCAAGTTCTCT
Rhl_F       CATTGTGGGTAGACCCAAATAAGTGTCTGACAATGCCTCAGTAACAATGTCAAGTTCTCT
Rhl_R       CATTGTGGGTAGACCCAAATAAGTGTCTGACAATGCCTCAGTAACAATGTCAAGTTCTCT
            ************************************************************

Karat_F     GCAGATATTCTCTCTCACACAGACCCCGGTATTCGGGCTGAACAATATACTAGATTTTGC
Karat_R     GCAGATATTCTCTCTCACACAGACCCCGGTATTCGGGCTGAACAATATACTAGATTTTGC
Rhl_F       GCAGATATTCTCTCTCACACAGACCCCGGTATTCGGGCTGAACAATATACTAGATTTTGC
Rhl_R       GCAGATATTCTCTCTCACACAGACCCCGGTATTCGGGCTGAACAATATACTAGATTTTGC
            ************************************************************

Karat_F     AGTACTTACAACTTGCCCTGAGCTGGTACAATAAAGATCCAACACCTTCTTAAGATTGCT
Karat_R     AGTACTTACAACTTGCCCTGAGCTGGTACAATAAAGATCCAACACCTTCTTAAGATTGCT
Rhl_F       AGTACTTACAACTTGCCCTGAGCTGGTACAATAAAGATCCAACACCTTCTTAAGATTGCT
Rhl_R       AGTACTTACAACTTGCCCTGAGCTGGTACAATAAAGATCCAACACCTTCTTAAGATTGCT
            ************************************************************

Karat_F     TGCATTCTGGGCATTGGCTTTCATCAACACCAACGAGTCATCTGCAAATAAAAGATGTGA
Karat_R     TGCATTCTGGGCATTGGCTTTCATCAACACCAACGAGTCATCTGCAAATAAAAGATGTGA
Rhl_F       TGCATTCTGGGCATTGGCTTTCATCAACACCAACGAGTCATCTGCAAATAAAAGATGTGA
Rhl_R       TGCATTCTGGGCATTGGCTTTCATCAACACCAACGAGTCATCTGCAAATAAAAGATGTGA
            ************************************************************

Karat_F     TACAGCGGGTGCACTCCTACACACCTTAACACACTCCAATCCTCGAGATTCCTCCTCGTG
Karat_R     TACAGCGGGTGCACTCCTACACACCTTAACACACTCCAATCCTCGAGATTCCTCCTCGTG
Rhl_F       TACAGCGGGTGCACTCCTACACACCTTAACACACTCCAATCCTCGAGATTCCTCCTCGTG
Rhl_R       TACAGCGGGTGCACTCCTACACACCTTAACACACTCCAATCCTCGAGATTCCTCCTCGTG
            ************************************************************

Karat_F     AGCTAATAAGCTTTTTTTTAAGAAGTGAGCTAATAAGCTTGATAGACCTTCAGAGCATAT
Karat_R     AGCTAATAAGCTTTTTTTTAAGAAGTGAGCTAATAAGCTTGATAGACCTTCAGAGCATAT
Rhl_F       AGCTAATAAGCTTTTTTTTAAGAAGTGAGCTAATAAGCTTGATAGACCTTCAGAGCATAT
Rhl_R       AGCTAATAAGCTTTTTTTTAAGAAGTGAGCTAATAAGCTTGATAGACCTTCAGAGCATAT
            ************************************************************

Karat_F     TAAGAACAAATATGGGATAACGGATCCCCCTATCTCAGGCCTCTACCGGGCACAAATTCG
Karat_R     TAAGAACAAATATGGGATAACGGATCCCCCTATCTCAGGCCTCTACCGGGCACAAATTCG
Rhl_F       TAAGAACAAATATGGGATAACGGATCCCCCTATCTCAGGCCTCTACCGGGCACAAATTCG
Rhl_R       TAAGAACAAATATGGGATAACGGATCCCCCTATCTCAGGCCTCTACCGGGCACAAATTCG
            ************************************************************

Karat_F     TTCGTCTCCGTATTATTAGAAACGCACTCTGTACGTGACCGAGGAAACACACTCCATATT
Karat_R     TTCGTCTCCGTATTATTAGAAACGCACTCTGTACGTGACCGAGGAAACACACTCCATATT
Rhl_F       TTCGTCTCCGTATTATTAGAAACGCACTCTGTACGTGACCGAGGAAACACACTCCATATT
Rhl_R       TTCGTCTCCGTATTATTAGAAACGCACTCTGTACGTGACCGAGGAAACACACTCCATATT
            ************************************************************

Karat_F     CAACACCTCTTCAATTCTTCCTCTAGCCCAATAACCAACCCGTCACTACGTTCTCCCTCG
Karat_R     CAACACCTCTTCAATTCTTCCTCTAGCCCAATAACCAACCCGTCACTACGTTCTCCCTCG
Rhl_F       CAACACCTCTTCAATTCTTCCTCTAGCCCAATAACCAACCCGTCACTACGTTCTCCCTCG
Rhl_R       CAACACCTCTTCAATTCTTCCTCTAGCCCAATAACCAACCCGTCACTACGTTCTCCCTCG
            ************************************************************

Karat_F     AACAGAGTCGCCCCGGTTTGCAGCGCCGCACCACGGGACCTCTCCGGCCGGCGACGACAA
Karat_R     AACAGAGTCGCCCCGGTTTGCAGCGCCGCACCACGGGACCTCTCCGGCCGGCGACGACAA
Rhl_F       AACAGAGTCGCCCCGGTTTGCAGCGCCGCACCACGGGACCTCTCCGGCCGGCGACGACAA
Rhl_R       AACAGAGTCGCCCCGGTTTGCAGCGCCGCACCACGGGACCTCTCCGGCCGGCGACGACAA
            ************************************************************

Karat_F     CCGGCCTCCGCCGGCAAACGGCGCACGCAGCGCAGGTAAGCTTCTTTCCCTCCATCCCCG
Karat_R     CCGGCCTCCGCCGGCAAACGGCGCACGCAGCGCAGGTAAGCTTCTTTCCCTCCATCCCCG
Rhl_F       CCGGCCTCCGCCGGCAAACGGCGCACGCAGCGCAGGTAAGCTTCTTTCCCTCCATCCCCG
Rhl_R       CCGGCCTCCGCCGGCAAACGGCGCACGCAGCGCAGGTAAGCTTCTTTCCCTCCATCCCCG
            ************************************************************
```

```
Karat_F     CCCCCAACCCGCTAAATCCACTCGCCCGCTCGGAAAAACAAAATGAAACGTCTCGTCGGC
Karat_R     CCCCCAACCCGCTAAATCCACTCGCCCGCTCGGAAAAACAAAATGAAACGTCTCGTCGGC
Rhl_F       CCCCCAACCCGCTAAATCCACTCGCCCGCTCGGAAAAACAAAATGAAACGTCTCGTCGGC
Rhl_R       CCCCCAACCCGCTAAATCCACTCGCCCGCTCGGAAAAACAAAATGAAACGTCTCGTCGGC
            ************************************************************

Karat_F     GCCGCGTCCGCCGCCTCTCACGCGAGCGAGCCAGCGGAGGCGCTTTCTCAGGCGACCCGT
Karat_R     GCCGCGTCCGCCGCCTCTCACGCGAGCGAGCCAGCGGAGGCGCTTTCTCAGGCGACCCGT
Rhl_F       GCCGCGTCCGCCGCCTCTCACGCGAGCGAGCCAGCGGAGGCGCTTTCTCAGGCGACCCGT
Rhl_R       GCCGCGTCCGCCGCCTCTCACGCGAGCGAGCCAGCGGAGGCGCTTTCTCAGGCGACCCGT
            ************************************************************

Karat_F     CCGCCGCCTCCTCCTCCTGTGCGGGTCTTCGTCTCTGTGCGGGTCTTCGTCTCCGGCGAC
Karat_R     CCGCCGCCTCCTCCTCCTGTGCGGGTCTTCGTCTCTGTGCGGGTCTTCGTCTCCGGCGAC
Rhl_F       CCGCCGCCTCCTCCTCCTGTGCGGGTCTTCGTCTCTGTGCGGGTCTTCGTCTCCGGCGAC
Rhl_R       CCGCCGCCTCCTCCTCCTGTGCGGGTCTTCGTCTCTGTGCGGGTCTTCGTCTCCGGCGAC
            ************************************************************

Karat_F     GCCTCCTCCTGCGGCGTCGCCCGCGAGGCTAGCCTGCTTCCTCTTCCTTCTCGCACGACG
Karat_R     GCCTCCTCCTGCGGCGTCGCCCGCGAGGCTAGCCTGCTTCCTCTTCCTTCTCGCACGACG
Rhl_F       GCCTCCTCCTGCGGCGTCGCCCGCGAGGCTAGCCTGCTTCCTCTTCCTTCTCGCACGACG
Rhl_R       GCCTCCTCCTGCGGCGTCGCCCGCGAGGCTAGCCTGCTTCCTCTTCCTTCTCGCACGACG
            ************************************************************

Karat_F     ACATGTTTTTTTTTTTAGGTTTAGGCGATTTCGGCCATGGCAGAGGGATAATTTTTTGTT
Karat_R     ACATGTTTTTTTTTTTAGGTTTAGGCGATTTCGGCCATGGCAGAGGGATAATTTTTTGTT
Rhl_F       ACATGTTTTTTTTTTTAGGTTTAGGCGATTTCGGCCATGGCAGAGGGATAATTTTTTGTT
Rhl_R       ACATGTTTTTTTTTTTAGGTTTAGGCGATTTCGGCCATGGCAGAGGGATAATTTTTTGTT
            ************************************************************

Karat_F     CGGCGAACTCAAGGAGAAATGAAATTAACCTCAGAGTGTGCTGTGTGGAAGTCCTATGCT
Karat_R     CGGCGAACTCAAGGAGAAATGAAATTAACCTCAGAGTGTGCTGTGTGGAAGTCCTATGCT
Rhl_F       CGGCGAACTCAAGGAGAAATGAAATTAACCTCAGAGTGTGCTGTGTGGAAGTCCTATGCT
Rhl_R       CGGCGAACTCAAGGAGAAATGAAATTAACCTCAGAGTGTGCTGTGTGGAAGTCCTATGCT
            ************************************************************

Karat_F     ATGCAGTCACTAACATTAGGGGGAAGTTGTCAGGCATGCCCAACTGCCCATCATTATTTT
Karat_R     ATGCAGTCACTAACATTAGGGGGAAGTTGTCAGGCATGCCCAACTGCCCATCATTATTTT
Rhl_F       ATGCAGTCACTAACATTAGGGGGAAGTTGTCAGGCATGCCCAACTGCCCATCATTATTTT
Rhl_R       ATGCAGTCACTAACATTAGGGGGAAGTTGTCAGGCATGCCCAACTGCCCATCATTATTTT
            ************************************************************

Karat_F     CTGGATCAAGCATATCATCAGCCACATCACTGCCTTCAGATTCAGTTTGTTGAGGCCACT
Karat_R     CTGGATCAAGCATATCATCAGCCACATCACTGCCTTCAGATTCAGTTTGTTGAGGCCACT
Rhl_F       CTGGATCAAGCATATCATCAGCCACATCACTGCCTTCAGATTCAGTTTGTTGAGGCCACT
Rhl_R       CTGGATCAAGCATATCATCAGCCACATCACTGCCTTCAGATTCAGTTTGTTGAGGCCACT
            ************************************************************

Karat_F     AGCTGCTTGCTGCTTCTGGTACTTTTTTTTATTGAGAACAAATAACAGTCCTTCTGTTCA
Karat_R     AGCTGCTTGCTGCTTCTGGTACTTTTTTTTATTGAGAACAAATAACAGTCCTTCTGTTCA
Rhl_F       AGCTGCTTGCTGCTTCTGGTACTTTTTTTTATTGAGAACAAATAACAGTCCTTCTGTTCA
Rhl_R       AGCTGCTTGCTGCTTCTGGTACTTTTTTTTATTGAGAACAAATAACAGTCCTTCTGTTCA
            ************************************************************

Karat_F     TGCATTCAGGAAGAAGGAATTGTCTTCAGTTTTTTCTGAAACTGAAGCTTTGTAGCCGCT
Karat_R     TGCATTCAGGAAGAAGGAATTGTCTTCAGTTTTTTCTGAAACTGAAGCTTTGTAGCCGCT
Rhl_F       TGCATTCAGGAAGAAGGAATTGTCTTCAGTTTTTTCTGAAACTGAAGCTTTGTAGCCGCT
Rhl_R       TGCATTCAGGAAGAAGGAATTGTCTTCAGTTTTTTCTGAAACTGAAGCTTTGTAGCCGCT
            ************************************************************

Karat_F     AGAATAGCAGTCCTGTAGTGCTGTTCTCGTAGAATAGCAGCCCTGTAGTGCTGTTCTTGT
Karat_R     AGAATAGCAGTCCTGTAGTGCTGTTCTCGTAGAATAGCAGCCCTGTAGTGCTGTTCTTGT
Rhl_F       AGAATAGCAGTCCTGTAGTGCTGTTCTCGTAGAATAGCAGCCCTGTAGTGCTGTTCTTGT
Rhl_R       AGAATAGCAGTCCTGTAGTGCTGTTCTCGTAGAATAGCAGCCCTGTAGTGCTGTTCTTGT
            ************************************************************

Karat_F     ATTTCCATTTTCACCTGTGCAGAGATTAAAGTTAAGTAGGTGAAAATTAATCTTTGTGAT
Karat_R     ATTTCCATTTTCACCTGTGCAGAGATTAAAGTTAAGTAGGTGAAAATTAATCTTTGTGAT
Rhl_F       ATTTCCATTTTCACCTGTGCAGAGATTAAAGTTAAGTAGGTGAAAATTAATCTTTGTGAT
```

```
Rhl_R       ATTTCCATTTTCACCTGTGCAGAGATTAAAGTTAAGTAGGTGAAAATTAATCTTTGTGAT
            ************************************************************

Karat_F     GATGATTACTTGATAAAACTACCAAGATTAAATTACTGAACTATGTCTGTTATTATCTAT
Karat_R     GATGATTACTTGATAAAACTACCAAGATTAAATTACTGAACTATGTCTGTTATTATCTAT
Rhl_F       GATGATTACTTGATAAAACTACCAAGATTAAATTACTGAACTATGTCTGTTATTATCTAT
Rhl_R       GATGATTACTTGATAAAACTACCAAGATTAAATTACTGAACTATGTCTGTTATTATCTAT
            ************************************************************

Karat_F     GTTGGGCGTCATCCGTCTATGTTGGGCGTCATCCGCCACAAGGACCATTTGATGAAAATC
Karat_R     GTTGGGCGTCATCCGTCTATGTTGGGCGTCATCCGCCACAAGGACCATTTGATGAAAATC
Rhl_F       GTTGGGCGTCATCCGTCTATGTTGGGCGTCATCCGCCACAAGGACCATTTGATGAAAATC
Rhl_R       GTTGGGCGTCATCCGTCTATGTTGGGCGTCATCCGCCACAAGGACCATTTGATGAAAATC
            ************************************************************

Karat_F     TTGCTTGTAGAGTTTAGAGAGTAGACTCTTTGCTTTTAGTTTCTCCATTCTGTTGCCATC
Karat_R     TTGCTTGTAGAGTTTAGAGAGTAGACTCTTTGCTTTTAGTTTCTCCATTCTGTTGCCATC
Rhl_F       TTGCTTGTAGAGTTTAGAGAGTAGACTCTTTGCTTTTAGTTTCTCCATTCTGTTGCCATC
Rhl_R       TTGCTTGTAGAGTTTAGAGAGTAGACTCTTTGCTTTTAGTTTCTCCATTCTGTTGCCATC
            ************************************************************

Karat_F     TGACCACAGGCTTAAAAATAAATGAGCAGGGAATTCTTGTTGTTGGTATGAACTCTAAAC
Karat_R     TGACCACAGGCTTAAAAATAAATGAGCAGGGAATTCTTGTTGTTGGTATGAACTCTAAAC
Rhl_F       TGACCACAGGCTTAAAAATAAATGAGCAGGGAATTCTTGTTGTTGGTATGAACTCTAAAC
Rhl_R       TGACCACAGGCTTAAAAATAAATGAGCAGGGAATTCTTGTTGTTGGTATGAACTCTAAAC
            ************************************************************

Karat_F     ATGTGAGTGTCCAGCATCATGACTCTTGATACTTGCCCTATATTTTATTTTCCTAAATCT
Karat_R     ATGTGAGTGTCCAGCATCATGACTCTTGATACTTGCCCTATATTTTATTTTCCTAAATCT
Rhl_F       ATGTGAGTGTCCAGCATCATGACTCTTGATACTTGCCCTATATTTTATTTTCCTAAATCT
Rhl_R       ATGTGAGTGTCCAGCATCATGACTCTTGATACTTGCCCTATATTTTATTTTCCTAAATCT
            ************************************************************

Karat_F     AACAGTATGTTTTGTATAATGTGCATAAGATTTGTAAGGACGAGAACTTGTTCACATTTT
Karat_R     AACAGTATGTTTTGTATAATGTGCATAAGATTTGTAAGGACGAGAACTTGTTCACATTTT
Rhl_F       AACAGTATGTTTTGTATAATGTGCATAAGATTTGTAAGGACGAGAACTTGTTCACATTTT
Rhl_R       AACAGTATGTTTTGTATAATGTGCATAAGATTTGTAAGGACGAGAACTTGTTCACATTTT
            ************************************************************

Karat_F     TTCCCTTCTGGAAAGAATCTGATTGGAGTGCAGCTGTGCATATTTAATTATTTATAGCAT
Karat_R     TTCCCTTCTGGAAAGAATCTGATTGGAGTGCAGCTGTGCATATTTAATTATTTATAGCAT
Rhl_F       TTCCCTTCTGGAAAGAATCTGATTGGAGTGCAGCTGTGCATATTTAATTATTTATAGCAT
Rhl_R       TTCCCTTCTGGAAAGAATCTGATTGGAGTGCAGCTGTGCATATTTAATTATTTATAGCAT
            ************************************************************

Karat_F     ATATTCAGTAAAACACAAGGTATCTGAGTAGCCTAATGGTTCATTAATCCTTGGTAGGAT
Karat_R     ATATTCAGTAAAACACAAGGTATCTGAGTAGCCTAATGGTTCATTAATCCTTGGTAGGAT
Rhl_F       ATATTCAGTAAAACACAAGGTATCTGAGTAGCCTAATGGTTCATTAATCCTTGGTAGGAT
Rhl_R       ATATTCAGTAAAACACAAGGTATCTGAGTAGCCTAATGGTTCATTAATCCTTGGTAGGAT
            ************************************************************

Karat_F     GCTACATGGATGAATCTAATCCTGTAACTCATTGGATAAGTTAATAAGATGGTGTGGCTG
Karat_R     GCTACATGGATGAATCTAATCCTGTAACTCATTGGATAAGTTAATAAGATGGTGTGGCTG
Rhl_F       GCTACATGGATGAATCTAATCCTGTAACTCATTGGATAAGTTAATAAGATGGTGTGGCTG
Rhl_R       GCTACATGGATGAATCTAATCCTGTAACTCATTGGATAAGTTAATAAGATGGTGTGGCTG
            ************************************************************

Karat_F     CTGTGTCCTGTGGAGTACTATGTTCTATGTTGCTTTCTGGATTTTCTTACCGCTCAAACT
Karat_R     CTGTGTCCTGTGGAGTACTATGTTCTATGTTGCTTTCTGGATTTTCTTACCGCTCAAACT
Rhl_F       CTGTGTCCTGTGGAGTACTATGTTCTATGTTGCTTTCTGGATTTTCTTACCGCTCAAACT
Rhl_R       CTGTGTCCTGTGGAGTACTATGTTCTATGTTGCTTTCTGGATTTTCTTACCGCTCAAACT
            ************************************************************

Karat_F     TTGCAATCCTTTTACCCTGCTACCAGTTTCCCTTATCCATAACTTGGACTGTGTTTTGAT
Karat_R     TTGCAATCCTTTTACCCTGCTACCAGTTTCCCTTATCCATAACTTGGACTGTGTTTTGAT
Rhl_F       TTGCAATCCTTTTACCCTGCTACCAGTTTCCCTTATCCATAACTTGGACTGTGTTTTGAT
Rhl_R       TTGCAATCCTTTTACCCTGCTACCAGTTTCCCTTATCCATAACTTGGACTGTGTTTTGAT
            ************************************************************
```

```
Karat_F      GTTCTTGTTGTTGTATCCATGTGAGAACGTTCGTGCTTTTTCATAACTTGTGAAGCTGCT
Karat_R      GTTCTTGTTGTTGTATCCATGTGAGAACGTTCGTGCTTTTTCATAACTTGTGAAGCTGCT
Rhl_F        GTTCTTGTTGTTGTATCCATGTGAGAACGTTCGTGCTTTTTCATAACTTGTGAAGCTGCT
Rhl_R        GTTCTTGTTGTTGTATCCATGTGAGAACGTTCGTGCTTTTTCATAACTTGTGAAGCTGCT
             ************************************************************

Karat_F      ACTATTCAAATTTTGCACTATGCAGTAATGGTGAAGCTCTGTTTGCTGTAAAGTTACCAA
Karat_R      ACTATTCAAATTTTGCACTATGCAGTAATGGTGAAGCTCTGTTTGCTGTAAAGTTACCAA
Rhl_F        ACTATTCAAATTTTGCACTATGCAGTAATGGTGAAGCTCTGTTTGCTGTAAAGTTACCAA
Rhl_R        ACTATTCAAATTTTGCACTATGCAGTAATGGTGAAGCTCTGTTTGCTGTAAAGTTACCAA
             ************************************************************

Karat_F      TCCTATTTCTAAGGGATCAGAAACCATTTTTTCTGTTGCATATATGGGACAATTCACTTA
Karat_R      TCCTATTTCTAAGGGATCAGAAACCATTTTTTCTGTTGCATATATGGGACAATTCACTTA
Rhl_F        TCCTATTTCTAAGGGATCAGAAACCATTTTTTCTGTTGCATATATGGGACAATTCACTTA
Rhl_R        TCCTATTTCTAAGGGATCAGAAACCATTTTTTCTGTTGCATATATGGGACAATTCACTTA
             ************************************************************

Karat_F      TCTGGGTCATAGAAATGGATGGGAGCAATCAATTTTAGCTTGAAACGGTTCTGTGAAAAA
Karat_R      TCTGGGTCATAGAAATGGATGGGAGCAATCAATTTTAGCTTGAAACGGTTCTGTGAAAAA
Rhl_F        TCTGGGTCATAGAAATGGATGGGAGCAATCAATTTTAGCTTGAAACGGTTCTGTGAAAAA
Rhl_R        TCTGGGTCATAGAAATGGATGGGAGCAATCAATTTTAGCTTGAAACGGTTCTGTGAAAAA
             ************************************************************

Karat_F      TAACCGAGATATTTTCTTTCCAAGGGAGTATGGGACTTTAATTGGGGCATACTAACAATT
Karat_R      TAACCGAGATATTTTCTTTCCAAGGGAGTATGGGACTTTAATTGGGGCATACTAACAATT
Rhl_F        TAACCGAGATATTTTCTTTCCAAGGGAGTATGGGACTTTAATTGGGGCATACTAACAATT
Rhl_R        TAACCGAGATATTTTCTTTCCAAGGGAGTATGGGACTTTAATTGGGGCATACTAACAATT
             ************************************************************

Karat_F      CATCCAAGACCTAAAGTAATGTTAGTTCATTCTTTAGTACACTGTGATGATTTTGTTGTA
Karat_R      CATCCAAGACCTAAAGTAATGTTAGTTCATTCTTTAGTACACTGTGATGATTTTGTTGTA
Rhl_F        CATCCAAGACCTAAAGTAATGTTAGTTCATTCTTTAGTACACTGTGATGATTTTGTTGTA
Rhl_R        CATCCAAGACCTAAAGTAATGTTAGTTCATTCTTTAGTACACTGTGATGATTTTGTTGTA
             ************************************************************

Karat_F      TCTGTATGTCCAGTGCAACTGGCAATGGCTAATCCAACTCTAACCTGCCACTGTTCCAAC
Karat_R      TCTGTATGTCCAGTGCAACTGGCAATGGCTAATCCAACTCTAACCTGCCACTGTTCCAAC
Rhl_F        TCTGTATGTCCAGTGCAACTGGCAATGGCTAATCCAACTCTAACCTGCCACTGTTCCAAC
Rhl_R        TCTGTATGTCCAGTGCAACTGGCAATGGCTAATCCAACTCTAACCTGCCACTGTTCCAAC
             ************************************************************

Karat_F      TCTAACCTGCCACTGTACTTGTGTAAGCTGACAGAGCTTTTGCCCTATTGTGTATATTCT
Karat_R      TCTAACCTGCCACTGTACTTGTGTAAGCTGACAGAGCTTTTGCCCTATTGTGTATATTCT
Rhl_F        TCTAACCTGCCACTGTACTTGTGTAAGCTGACAGAGCTTTTGCCCTATTGTGTATATTCT
Rhl_R        TCTAACCTGCCACTGTACTTGTGTAAGCTGACAGAGCTTTTGCCCTATTGTGTATATTCT
             ************************************************************

Karat_F      TCACAAATCTAAATGTAGCAGCAAGGTATTTGAATTATAATTGCTATTATTTGAGCATCT
Karat_R      TCACAAATCTAAATGTAGCAGCAAGGTATTTGAATTATAATTGCTATTATTTGAGCATCT
Rhl_F        TCACAAATCTAAATGTAGCAGCAAGGTATTTGAATTATAATTGCTATTATTTGAGCATCT
Rhl_R        TCACAAATCTAAATGTAGCAGCAAGGTATTTGAATTATAATTGCTATTATTTGAGCATCT
             ************************************************************

Karat_F      TTCATATGCTGCATGGAGATCGATTCTGATTAACATTTACGTATAGGCAGTTTCAGGATG
Karat_R      TTCATATGCTGCATGGAGATCGATTCTGATTAACATTTACGTATAGGCAGTTTCAGGATG
Rhl_F        TTCATATGCTGCATGGAGATCGATTCTGATTAACATTTACGTATAGGCAGTTTCAGGATG
Rhl_R        TTCATATGCTGCATGGAGATCGATTCTGATTAACATTTACGTATAGGCAGTTTCAGGATG
             ************************************************************

Karat_F      GGTACTGAAGACTCTAAAGATATGCTGAAGAATGCGGACTGGAAGACAGTGAGTGGTCCC
Karat_R      GGTACTGAAGACTCTAAAGATATGCTGAAGAATGCGGACTGGAAGACAGTGAGTGGTCCC
Rhl_F        GGTACTGAAGACTCTAAAGATATGCTGAAGAATGCGGACTGGAAGACAGTGAGTGGTCCC
Rhl_R        GGTACTGAAGACTCTAAAGATATGCTGAAGAATGCGGACTGGAAGACAGTGAGTGGTCCC
             ************************************************************

Karat_F      GTTATTACCGAGTCAAGCCAGCCGGTTGTCAAGAAGCGTCTTCCGAAGAAAATCAGACAA
Karat_R      GTTATTACCGAGTCAAGCCAGCCGGTTGTCAAGAAGCGTCTTCCGAAGAAAATCAGACAA
Rhl_F        GTTATTACCGAGTCAAGCCAGCCGGTTGTCAAGAAGCGTCTTCCGAAGAAAATCAGACAA
```

```
Rhl_R       GTTATTACCGAGTCAAGCCAGCCGGTTGTCAAGAAGCGTCTTCCGAAGAAAATCAGACAA
            ************************************************************

Karat_F     GTCCCTGAGTGTTACTTTCTGCCTCGACGATCTTTGCCGTCTGCATTGGCAATCTATGGT
Karat_R     GTCCCTGAGTGTTACTTTCTGCCTCGACGATCTTTGCCGTCTGCATTGGCAATCTATGGT
Rhl_F       GTCCCTGAGTGTTACTTTCTGCCTCGACGATCTTTGCCGTCTGCATTGGCAATCTATGGT
Rhl_R       GTCCCTGAGTGTTACTTTCTGCCTCGACGATCTTTGCCGTCTGCATTGGCAATCTATGGT
            ************************************************************

Karat_F     GCTGTTTGTGCTGCTGGAGTTGGTGCAGGGATGTTGCTTGAGGTTTGGATAAACAAAAAG
Karat_R     GCTGTTTGTGCTGCTGGAGTTGGTGCAGGGATGTTGCTTGAGGTTTGGATAAACAAAAAG
Rhl_F       GCTGTTTGTGCTGCTGGAGTTGGTGCAGGGATGTTGCTTGAGGTTTGGATAAACAAAAAG
Rhl_R       GCTGTTTGTGCTGCTGGAGTTGGTGCAGGGATGTTGCTTGAGGTTTGGATAAACAAAAAG
            ************************************************************

Karat_F     ATCAAAGGTATGATCCTGCATTTCATGGCCGAACCCTCTGGATGAACCACTTATCATAAG
Karat_R     ATCAAAGGTATGATCCTGCATTTCATGGCCGAACCCTCTGGATGAACCACTTATCATAAG
Rhl_F       ATCAAAGGTATGATCCTGCATTTCATGGCCGAACCCTCTGGATGAACCACTTATCATAAG
Rhl_R       ATCAAAGGTATGATCCTGCATTTCATGGCCGAACCCTCTGGATGAACCACTTATCATAAG
            ************************************************************

Karat_F     AAAACTACCCGACCTGGAAATTTGTGATCTCGTACTGTACCATGGAAATCGTTGCATATC
Karat_R     AAAACTACCCGACCTGGAAATTTGTGATCTCGTACTGTACCATGGAAATCGTTGCATATC
Rhl_F       AAAACTACCCGACCTGGAAATTTGTGATCTCGTACTGTACCATGGAAATCGTTGCATATC
Rhl_R       AAAACTACCCGACCTGGAAATTTGTGATCTCGTACTGTACCATGGAAATCGTTGCATATC
            ************************************************************

Karat_F     TTTTTCATCCTCATCGATTGTGTTTTTCCACACCCTCTTACTTTTTGTCGATTGCTGCAT
Karat_R     TTTTTCATCCTCATCGATTGTGTTTTTCCACACCCTCTTACTTTTTGTCGATTGCTGCAT
Rhl_F       TTTTTCATCCTCATCGATTGTGTTTTTCCACACCCTCTTACTTTTTGTCGATTGCTGCAT
Rhl_R       TTTTTCATCCTCATCGATTGTGTTTTTCCACACCCTCTTACTTTTTGTCGATTGCTGCAT
            ************************************************************

Karat_F     GCAGAGGATGGAGGCGTTGTCTGGGAGATGGGCAAATGATGTTGATGCACTTTGAGTTGG
Karat_R     GCAGAGGATGGAGGCGTTGTCTGGGAGATGGGCAAATGATGTTGATGCACTTTGAGTTGG
Rhl_F       GCAGAGGATGGAGGCGTTGTCTGGGAGATGGGCAAATGATGTTGATGCACTTTGAGTTGG
Rhl_R       GCAGAGGATGGAGGCGTTGTCTGGGAGATGGGCAAATGATGTTGATGCACTTTGAGTTGG
            ************************************************************

Karat_F     TAACAATCTGGTTTGGCAGACTCACTTGGCTTGCAGACTCACTTGGCTTCACTTATGCTG
Karat_R     TAACAATCTGGTTTGGCAGACTCACTTGGCTTGCAGACTCACTTGGCTTCACTTATGCTG
Rhl_F       TAACAATCTGGTTTGGCAGACTCACTTGGCTTGCAGACTCACTTGGCTTCACTTATGCTG
Rhl_R       TAACAATCTGGTTTGGCAGACTCACTTGGCTTGCAGACTCACTTGGCTTCACTTATGCTG
            ************************************************************

Karat_F     CTTAAGCTGTATCACGGAAATAAGCGGAGATTTGTTGTTTCAGACTATCTGTTGAGACTG
Karat_R     CTTAAGCTGTATCACGGAAATAAGCGGAGATTTGTTGTTTCAGACTATCTGTTGAGACTG
Rhl_F       CTTAAGCTGTATCACGGAAATAAGCGGAGATTTGTTGTTTCAGACTATCTGTTGAGACTG
Rhl_R       CTTAAGCTGTATCACGGAAATAAGCGGAGATTTGTTGTTTCAGACTATCTGTTGAGACTG
            ************************************************************

Karat_F     ACAAACGCAGACAATGTTTTCCGGTGTAATTTACACAACCTTGTCCCTGTGTTTCTAGTT
Karat_R     ACAAACGCAGACAATGTTTTCCGGTGTAATTTACACAACCTTGTCCCTGTGTTTCTAGTT
Rhl_F       ACAAACGCAGACAATGTTTTCCGGTGTAATTTACACAACCTTGTCCCTGTGTTTCTAGTT
Rhl_R       ACAAACGCAGACAATGTTTTCCGGTGTAATTTACACAACCTTGTCCCTGTGTTTCTAGTT
            ************************************************************

Karat_F     TGGTTGTGCAAGCTGTGTGATGGTTCAAACTTTATATTCGGCAAGCAAGCTGAAAACACC
Karat_R     TGGTTGTGCAAGCTGTGTGATGGTTCAAACTTTATATTCGGCAAGCAAGCTGAAAACACC
Rhl_F       TGGTTGTGCAAGCTGTGTGATGGTTCAAACTTTATATTCGGCAAGCAAGCTGAAAACACC
Rhl_R       TGGTTGTGCAAGCTGTGTGATGGTTCAAACTTTATATTCGGCAAGCAAGCTGAAAACACC
            ************************************************************

Karat_F     AGTCATCAACGCATCTGTAGCTGTCATTTGTAGGCATCATTAGTACTGCACAGGTAAGAA
Karat_R     AGTCATCAACGCATCTGTAGCTGTCATTTGTAGGCATCATTAGTACTGCACAGGTAAGAA
Rhl_F       AGTCATCAACGCATCTGTAGCTGTCATTTGTAGGCATCATTAGTACTGCACAGGTAAGAA
Rhl_R       AGTCATCAACGCATCTGTAGCTGTCATTTGTAGGCATCATTAGTACTGCACAGGTAAGAA
            ************************************************************
```

```
Karat_F        CAATCTGAAACTTTGTCTGCAGGTAGTTGCACGTCGTGATCAAAATAGTGTACCATGATT
Karat_R        CAATCTGAAACTTTGTCTGCAGGTAGTTGCACGTCGTGATCAAAATAGTGTACCATGATT
Rhl_F          CAATCTGAAACTTTGTCTGCAGGTAGTTGCACGTCGTGATCAAAATAGTGTACCATGATT
Rhl_R          CAATCTGAAACTTTGTCTGCAGGTAGTTGCACGTCGTGATCAAAATAGTGTACCATGATT
               ************************************************************

Karat_F        GATCTATTTTGTTTTGTTTATTATACCATGAAGTCTGCAGAAATCGTTACCATGATTAAT
Karat_R        GATCTATTTTGTTTTGTTTATTATACCATGAAGTCTGCAGAAATCGTTACCATGATTAAT
Rhl_F          GATCTATTTTGTTTTGTTTATTATACCATGAAGTCTGCAGAAATCGTTACCATGATTAAT
Rhl_R          GATCTATTTTGTTTTGTTTATTATACCATGAAGTCTGCAGAAATCGTTACCATGATTAAT
               ************************************************************

Karat_F        CTATTTTGTTTTGTTTATTATACCATGAAGTCTGGCAGAAATCGTCGTGCCCTCTGTATA
Karat_R        CTATTTTGTTTTGTTTATTATACCATGAAGTCTGGCAGAAATCGTCGTGCCCTCTGTATA
Rhl_F          CTATTTTGTTTTGTTTATTATACCATGAAGTCTGGCAGAAATCGTCGTGCCCTCTGTATA
Rhl_R          CTATTTTGTTTTGTTTATTATACCATGAAGTCTGGCAGAAATCGTCGTGCCCTCTGTATA
               ************************************************************

Karat_F        ATATTGGCAGCAGAAGTTGTTTGGTGATCAATAAATGCAAAAATAATAAATATCGGAAAA
Karat_R        ATATTGGCAGCAGAAGTTGTTTGGTGATCAATAAATGCAAAAATAATAAATATCGGAAAA
Rhl_F          ATATTGGCAGCAGAAGTTGTTTGGTGATCAATAAATGCAAAAATAATAAATATCGGAAAA
Rhl_R          ATATTGGCAGCAGAAGTTGTTTGGTGATCAATAAATGCAAAAATAATAAATATCGGAAAA
               ************************************************************

Karat_F        ATGTTTGAAGTAGTGCTATGCCATATACAGTTGGTTGGCCTCTGCTGTACTGGAGACAGA
Karat_R        ATGTTTGAAGTAGTGCTATGCCATATACAGTTGGTTGGCCTCTGCTGTACTGGAGACAGA
Rhl_F          ATGTTTGAAGTAGTGCTATGCCATATACAGTTGGTTGGCCTCTGCTGTACTGGAGACAGA
Rhl_R          ATGTTTGAAGTAGTGCTATGCCATATACAGTTGGTTGGCCTCTGCTGTACTGGAGACAGA
               ************************************************************

Karat_F        TATGATAGAAATTTGCATGCCCAAGAAGACAGATGCTTACCATATGCCTCATTTGTCTCC
Karat_R        TATGATAGAAATTTGCATGCCCAAGAAGACAGATGCTTACCATATGCCTCATTTGTCTCC
Rhl_F          TATGATAGAAATTTGCATGCCCAAGAAGACAGATGCTTACCATATGCCTCATTTGTCTCC
Rhl_R          TATGATAGAAATTTGCATGCCCAAGAAGACAGATGCTTACCATATGCCTCATTTGTCTCC
               ************************************************************

Karat_F        TTCATCTGCTGTGTTGGCACTTCCATCTCCCCATTATGCAAATGCAACCTTTCTGGCAAA
Karat_R        TTCATCTGCTGTGTTGGCACTTCCATCTCCCCATTATGCAAATGCAACCTTTCTGGCAAA
Rhl_F          TTCATCTGCTGTGTTGGCACTTCCATCTCCCCATTATGCAAATGCAACCTTTCTGGCAAA
Rhl_R          TTCATCTGCTGTGTTGGCACTTCCATCTCCCCATTATGCAAATGCAACCTTTCTGGCAAA
               ************************************************************

Karat_F        CGGAGCGTCTGAATTCATTATCATCGCTTGGAAAGCATTCGAGAAACTTGTGACCAGTTT
Karat_R        CGGAGCGTCTGAATTCATTATCATCGCTTGGAAAGCATTCGAGAAACTTGTGACCAGTTT
Rhl_F          CGGAGCGTCTGAATTCATTATCATCGCTTGGAAAGCATTCGAGAAACTTGTGACCAGTTT
Rhl_R          CGGAGCGTCTGAATTCATTATCATCGCTTGGAAAGCATTCGAGAAACTTGTGACCAGTTT
               ************************************************************

Karat_F        GTTTCATTATATTATTTGTTTCCCGGACTTCATTTCTCTGAAGCGCCTGATTTATTCCTG
Karat_R        GTTTCATTATATTATTTGTTTCCCGGACTTCATTTCTCTGAAGCGCCTGATTTATTCCTG
Rhl_F          GTTTCATTATATTATTTGTTTCCCGGACTTCATTTCTCTGAAGCGCCTGATTTATTCCTG
Rhl_R          GTTTCATTATATTATTTGTTTCCCGGACTTCATTTCTCTGAAGCGCCTGATTTATTCCTG
               ************************************************************

Karat_F        CTGAATGATGATGCGCCCGCTATCTTTTCTCAGACAAAGTCAAAGTCTGGCCTTTCTCTA
Karat_R        CTGAATGATGATGCGCCCGCTATCTTTTCTCAGACAAAGTCAAAGTCTGGCCTTTCTCTA
Rhl_F          CTGAATGATGATGCGCCCGCTATCTTTTCTCAGACAAAGTCAAAGTCTGGCCTTTCTCTA
Rhl_R          CTGAATGATGATGCGCCCGCTATCTTTTCTCAGACAAAGTCAAAGTCTGGCCTTTCTCTA
               ************************************************************

Karat_F        TGCCATGGGCGCACTTGCAAAAGAATAGAGATCCAGCGTCCTAGCCTTTCTCATCCAAGA
Karat_R        TGCCATGGGCGCACTTGCAAAAGAATAGAGATCCAGCGTCCTAGCCTTTCTCATCCAAGA
Rhl_F          TGCCATGGGCGCACTTGCAAAAGAATAGAGATCCAGCGTCCTAGCCTTTCTCATCCAAGA
Rhl_R          TGCCATGGGCGCACTTGCAAAAGAATAGAGATCCAGCGTCCTAGCCTTTCTCATCCAAGA
               ************************************************************

Karat_F        AGGGACACCACATTT
Karat_R        AGGGACACCACATTT
Rhl_F          AGGGACACCACATTT
```

```
Rhl_R         AGGGACACCACATTT
              ***************
```

Alignment_HORVU7Hr1G030280

```
Karat_F      GCGAGGGAGTTGCATGATCTCATGGTCTTAGGAACAAATACTTGACATAAAGAAAGCAAT
Karat_R      GCGAGGGAGTTGCATGATCTCATGGTCTTAGGAACAAATACTTGACATAAAGAAAGCAAT
Rhl_F        GCGAGGGAGTTGCATGATCTCATGGTCTTAGGAACAAATACTTGACATAAAGAAAGCAAT
Rhl_R        GCGAGGGAGTTGCATGATCTCATGGTCTTAGGAACAAATACTTGACATAAAGAAAGCAAT
             ************************************************************

Karat_F      AGCAATAAACTTAATGATACGTTCAGATGGTATGCTTACGGTTGGATCTTGTCCATCACA
Karat_R      AGCAATAAACTTAATGATACGTTCAGATGGTATGCTTACGGTTGGATCTTGTCCATCACA
Rhl_F        AGCAATAAACTTAATGATACGTTCAGATGGTATGCTTACGGTTGGATCTTGTCCATCACA
Rhl_R        AGCAATAAACTTAATGATACGTTCAGATGGTATGCTTACGGTTGGATCTTGTCCATCACA
             ************************************************************

Karat_F      TCATTCTCCTAATGATGTGGTCTCGTTATCAAATGACAACTCATGTCTATGACTAGAAAA
Karat_R      TCATTCTCCTAATGATGTGGTCTCGTTATCAAATGACAACTCATGTCTATGACTAGAAAA
Rhl_F        TCATTCTCCTAATGATGTGGTCTCGTTATCAAATGACAACTCATGTCTATGACTAGAAAA
Rhl_R        TCATTCTCCTAATGATGTGGTCTCGTTATCAAATGACAACTCATGTCTATGACTAGAAAA
             ************************************************************

Karat_F      CCTTAACCATCTTTGATCAATGAGCTAGTCTAGTAAAGACTTAATAGGGACTTTGTGTTA
Karat_R      CCTTAACCATCTTTGATCAATGAGCTAGTCTAGTAAAGACTTAATAGGGACTTTGTGTTA
Rhl_F        CCTTAACCATCTTTGATCAATGAGCTAGTCTAGTAAAGACTTAATAGGGACTTTGTGTTA
Rhl_R        CCTTAACCATCTTTGATCAATGAGCTAGTCTAGTAAAGACTTAATAGGGACTTTGTGTTA
             ************************************************************

Karat_F      CGGAAATGCAATTCGGCTCACATTACGAGATGATGTTTTATTGTCATGGAAAATTTCAAA
Karat_R      CGGAAATGCAATTCGGCTCACATTACGAGATGATGTTTTATTGTCATGGAAAATTTCAAA
Rhl_F        CGGAAATGCAATTCGGCTCACATTACGAGATGATGTTTTATTGTCATGGAAAATTTCAAA
Rhl_R        CGGAAATGCAATTCGGCTCACATTACGAGATGATGTTTTATTGTCATGGAAAATTTCAAA
             ************************************************************

Karat_F      TTGAGACACGTTACAAGATGTGAGCTACGAAAAAGACAAAATCAGATTTGAATACTTTTC
Karat_R      TTGAGACACGTTACAAGATGTGAGCTACGAAAAAGACAAAATCAGATTTGAATACTTTTC
Rhl_F        TTGAGACACGTTACAAGATGTGAGCTACGAAAAAGACAAAATCAGATTTGAATACTTTTC
Rhl_R        TTGAGACACGTTACAAGATGTGAGCTACGAAAAAGACAAAATCAGATTTGAATACTTTTC
             ************************************************************

Karat_F      AAATACTAATTGAAATTTGTCTTTTTCGTAGTTCACATCTTGTAATGCGTTTTAACTTGA
Karat_R      AAATACTAATTGAAATTTGTCTTTTTCGTAGTTCACATCTTGTAATGCGTTTTAACTTGA
Rhl_F        AAATACTAATTGAAATTTGTCTTTTTCGTAGTTCACATCTTGTAATGCGTTTTAACTTGA
Rhl_R        AAATACTAATTGAAATTTGTCTTTTTCGTAGTTCACATCTTGTAATGCGTTTTAACTTGA
             ************************************************************

Karat_F      AATTTTGTGTGACAATAAGACATCATCTTCTTAACATTCTATTTTTTTTAGATTCTTGGA
Karat_R      AATTTTGTGTGACAATAAGACATCATCTTCTTAACATTCTATTTTTTTTAGATTCTTGGA
Rhl_F        AATTTTGTGTGACAATAAGACATCATCTTCTTAACATTCTATTTTTTTTAGATTCTTGGA
Rhl_R        AATTTTGTGTGACAATAAGACATCATCTTCTTAACATTCTATTTTTTTTAGATTCTTGGA
             ************************************************************

Karat_F      AAACTTTAAAATATGATTTTCATGATGTTTTTATTAAATGTTGGTTTCCGTATGATACAA
Karat_R      AAACTTTAAAATATGATTTTCATGATGTTTTTATTAAATGTTGGTTTCCGTATGATACAA
Rhl_F        AAACTTTAAAATATGATTTTCATGATGTTTTTATTAAATGTTGGTTTCCGTATGATACAA
Rhl_R        AAACTTTAAAATATGATTTTCATGATGTTTTTATTAAATGTTGGTTTCCGTATGATACAA
             ************************************************************

Karat_F      GTGCACCCCGTACTGTTGTTTGCAAAGTATTCCAGCGTAGTGAGCGGCCCAGATCAGCCC
Karat_R      GTGCACCCCGTACTGTTGTTTGCAAAGTATTCCAGCGTAGTGAGCGGCCCAGATCAGCCC
Rhl_F        GTGCACCCCGTACTGTTGTTTGCAAAGTATTCCAGCGTAGTGAGCGGCCCAGATCAGCCC
Rhl_R        GTGCACCCCGTACTGTTGTTTGCAAAGTATTCCAGCGTAGTGAGCGGCCCAGATCAGCCC
             ************************************************************

Karat_F      GAAGCCAGTCGGCAAAGCTCCAGGCGAAACCTAGTCACTCGACTACTCCCAACCGGCCGC
Karat_R      GAAGCCAGTCGGCAAAGCTCCAGGCGAAACCTAGTCACTCGACTACTCCCAACCGGCCGC
Rhl_F        GAAGCCAGTCGGCAAAGCTCCAGGCGAAACCTAGTCACTCGACTACTCCCAACCGGCCGC
Rhl_R        GAAGCCAGTCGGCAAAGCTCCAGGCGAAACCTAGTCACTCGACTACTCCCAACCGGCCGC
             ************************************************************
```

```
Karat_F      CGCCGTCTCCCGTCCACGTTCCGGCCGCCGGAGCTCGCCTCCTCCTCCACCCGCTACCTC
Karat_R      CGCCGTCTCCCGTCCACGTTCCGGCCGCCGGAGCTCGCCTCCTCCTCCACCCGCTACCTC
Rhl_F        CGCCGTCTCCCGTCCACGTTCCGGCCGCCGGAGCTCGCCTCCTCCTCCACCCGCTACCTC
Rhl_R        CGCCGTCTCCCGTCCACGTTCCGGCCGCCGGAGCTCGCCTCCTCCTCCACCCGCTACCTC
             ************************************************************

Karat_F      TCGAGGCCACTCCGGACGGCCATGGCCCAGAGGGCCGCGGGCGGCCTTCTCCGACGGTCC
Karat_R      TCGAGGCCACTCCGGACGGCCATGGCCCAGAGGGCCGCGGGCGGCCTTCTCCGACGGTCC
Rhl_F        TCGAGGCCACTCCGGACGGCCATGGCCCAGAGGGCCGCGGGCGGCCTTCTCCGACGGTCC
Rhl_R        TCGAGGCCACTCCGGACGGCCATGGCCCAGAGGGCCGCGGGCGGCCTTCTCCGACGGTCC
             ************************************************************

Karat_F      CTCGGGCTCGCGCCGCCGACAACCCCGAGGGCCCTGAGCACCAGCGCCACCACGCCGGCG
Karat_R      CTCGGGCTCGCGCCGCCGACAACCCCGAGGGCCCTGAGCACCAGCGCCACCACGCCGGCG
Rhl_F        CTCGGGCTCGCGCCGCCGACAACCCCGAGGGCCCTGAGCACCAGCGCCACCACGCCGGCG
Rhl_R        CTCGGGCTCGCGCCGCCGACAACCCCGAGGGCCCTGAGCACCAGCGCCACCACGCCGGCG
             ************************************************************

Karat_F      GAGGGAGAGGCGGCGGCCAAGGCGAGGAGGAACAAGAAGAAGAACCTGTTCGACGTGGCG
Karat_R      GAGGGAGAGGCGGCGGCCAAGGCGAGGAGGAACAAGAAGAAGAACCTGTTCGACGTGGCG
Rhl_F        GAGGGAGAGGCGGCGGCCAAGGCGAGGAGGAACAAGAAGAAGAACCTGTTCGACGTGGCG
Rhl_R        GAGGGAGAGGCGGCGGCCAAGGCGAGGAGGAACAAGAAGAAGAACCTGTTCGACGTGGCG
             ************************************************************

Karat_F      CAATTCCTGCCGGACTGGGGCGTCGGCCACAGGGTCGCCAAGACCACCTGGCGCGACGTC
Karat_R      CAATTCCTGCCGGACTGGGGCGTCGGCCACAGGGTCGCCAAGACCACCTGGCGCGACGTC
Rhl_F        CAATTCCTGCCGGACTGGGGCGTCGGCCACAGGGTCGCCAAGACCACCTGGCGCGACGTC
Rhl_R        CAATTCCTGCCGGACTGGGGCGTCGGCCACAGGGTCGCCAAGACCACCTGGCGCGACGTC
             ************************************************************

Karat_F      TCCTACCAGATCACCAAGATCAACCTCTATAAGGTCGCCATCGCCACCATCCATAACAAT
Karat_R      TCCTACCAGATCACCAAGATCAACCTCTATAAGGTCGCCATCGCCACCATCCATAACAAT
Rhl_F        TCCTACCAGATCACCAAGATCAACCTCTATAAGGTCGCCATCGCCACCATCCATAACAAT
Rhl_R        TCCTACCAGATCACCAAGATCAACCTCTATAAGGTCGCCATCGCCACCATCCATAACAAT
             ************************************************************

Karat_F      CTCTGTGGAGGAGAAAATTGATCTCTTGTGTTCATTTCCGTGACTTAACTGCGGTTTCCT
Karat_R      CTCTGTGGAGGAGAAAATTGATCTCTTGTGTTCATTTCCGTGACTTAACTGCGGTTTCCT
Rhl_F        CTCTGTGGAGGAGAAAATTGATCTCTTGTGTTCATTTCCGTGACTTAACTGCGGTTTCCT
Rhl_R        CTCTGTGGAGGAGAAAATTGATCTCTTGTGTTCATTTCCGTGACTTAACTGCGGTTTCCT
             ************************************************************

Karat_F      GATCTCTGTATGTACTGATGCAGGATGGCCGCCACGGGAAGGCGTGGGGAATTCGGCACA
Karat_R      GATCTCTGTATGTACTGATGCAGGATGGCCGCCACGGGAAGGCGTGGGGAATTCGGCACA
Rhl_F        GATCTCTGTATGTACTGATGCAGGATGGCCGCCACGGGAAGGCGTGGGGAATTCGGCACA
Rhl_R        GATCTCTGTATGTACTGATGCAGGATGGCCGCCACGGGAAGGCGTGGGGAATTCGGCACA
             ************************************************************

Karat_F      AGGCCGGTGAGTCCTCCTGAATTACCGTTAATTTTGGTGGATTGGTGGAGCGTAAAATAA
Karat_R      AGGCCGGTGAGTCCTCCTGAATTACCGTTAATTTTGGTGGATTGGTGGAGCGTAAAATAA
Rhl_F        AGGCCGGTGAGTCCTCCTGAATTACCGTTAATTTTGGTGGATTGGTGGAGCGTAAAATAA
Rhl_R        AGGCCGGTGAGTCCTCCTGAATTACCGTTAATTTTGGTGGATTGGTGGAGCGTAAAATAA
             ************************************************************

Karat_F      ATGACAGGGTGTGTGTCGAATGGTAGAACATGAATGCCCCCTGGATGGAGGACTACTTCT
Karat_R      ATGACAGGGTGTGTGTCGAATGGTAGAACATGAATGCCCCCTGGATGGAGGACTACTTCT
Rhl_F        ATGACAGGGTGTGTGTCGAATGGTAGAACATGAATGCCCCCTGGATGGAGGACTACTTCT
Rhl_R        ATGACAGGGTGTGTGTCGAATGGTAGAACATGAATGCCCCCTGGATGGAGGACTACTTCT
             ************************************************************

Karat_F      GCTTGGCATCATCCTATGCAGTTGGGTGCACCAGGCGATGTTTCCGTTTTGGTTGCAGAT
Karat_R      GCTTGGCATCATCCTATGCAGTTGGGTGCACCAGGCGATGTTTCCGTTTTGGTTGCAGAT
Rhl_F        GCTTGGCATCATCCTATGCAGTTGGGTGCACCAGGCGATGTTTCCGTTTTGGTTGCAGAT
Rhl_R        GCTTGGCATCATCCTATGCAGTTGGGTGCACCAGGCGATGTTTCCGTTTTGGTTGCAGAT
             ************************************************************

Karat_F      TGTTGCTCTAGGATTAATTTGGTTGTCCTAGAATCTGTCTCATCTCGATAACATTGGGGT
Karat_R      TGTTGCTCTAGGATTAATTTGGTTGTCCTAGAATCTGTCTCATCTCGATAACATTGGGGT
Rhl_F        TGTTGCTCTAGGATTAATTTGGTTGTCCTAGAATCTGTCTCATCTCGATAACATTGGGGT
```

```
Rhl_R           TGTTGCTCTAGGATTAATTTGGTTGTCCTAGAATCTGTCTCATCTCGATAACATTGGGGT
                ************************************************************

Karat_F         TTAGGGTAAGGTGTAACAGCTCAAATACTTCATGTGGACATGCGGTGTAACCAAAAAAAT
Karat_R         TTAGGGTAAGGTGTAACAGCTCAAATACTTCATGTGGACATGCGGTGTAACCAAAAAAAT
Rhl_F           TTAGGGTAAGGTGTAACAGCTCAAATACTTCATGTGGACATGCGGTGTAACCAAAAAAAT
Rhl_R           TTAGGGTAAGGTGTAACAGCTCAAATACTTCATGTGGACATGCGGTGTAACCAAAAAAAT
                ************************************************************

Karat_F         TATAGGACATTGCGCTGTCTTTATCCCAGAAAGAAAAGATAAAAGAAGCACATCACACGT
Karat_R         TATAGGACATTGCGCTGTCTTTATCCCAGAAAGAAAAGATAAAAGAAGCACATCACACGT
Rhl_F           TATAGGACATTGCGCTGTCTTTATCCCAGAAAGAAAAGATAAAAGAAGCACATCACACGT
Rhl_R           TATAGGACATTGCGCTGTCTTTATCCCAGAAAGAAAAGATAAAAGAAGCACATCACACGT
                ************************************************************

Karat_F         AACAGTTATTTACCAGGTTGTGAAGGATGTATCCATTGCATCACTAGGCCACACTAGAAT
Karat_R         AACAGTTATTTACCAGGTTGTGAAGGATGTATCCATTGCATCACTAGGCCACACTAGAAT
Rhl_F           AACAGTTATTTACCAGGTTGTGAAGGATGTATCCATTGCATCACTAGGCCACACTAGAAT
Rhl_R           AACAGTTATTTACCAGGTTGTGAAGGATGTATCCATTGCATCACTAGGCCACACTAGAAT
                ************************************************************

Karat_F         CATTCAGTTGTTTGCTTCCCAGTTACCCCAAGTTATTTTTTTTTTTTTTTATTTTTATGT
Karat_R         CATTCAGTTGTTTGCTTCCCAGTTACCCCAAGTTATTTTTTTTTTTTTTTATTTTTATGT
Rhl_F           CATTCAGTTGTTTGCTTCCCAGTTACCCCAAGTTATTTTTTTTTTTTTTTATTTTTATGT
Rhl_R           CATTCAGTTGTTTGCTTCCCAGTTACCCCAAGTTATTTTTTTTTTTTTTTATTTTTATGT
                ************************************************************

Karat_F         CTGGACCAAGATAGATCATATTTTCCTTGCAAGGTAGATCATGTTTTCCCTGCCGTTTGC
Karat_R         CTGGACCAAGATAGATCATATTTTCCTTGCAAGGTAGATCATGTTTTCCCTGCCGTTTGC
Rhl_F           CTGGACCAAGATAGATCATATTTTCCTTGCAAGGTAGATCATGTTTTCCCTGCCGTTTGC
Rhl_R           CTGGACCAAGATAGATCATATTTTCCTTGCAAGGTAGATCATGTTTTCCCTGCCGTTTGC
                ************************************************************

Karat_F         AAAAGTCATGGCATGGTTACTGTTGTTGGTCTTCAACAAAACCGTTAGGACGCTTAATGT
Karat_R         AAAAGTCATGGCATGGTTACTGTTGTTGGTCTTCAACAAAACCGTTAGGACGCTTAATGT
Rhl_F           AAAAGTCATGGCATGGTTACTGTTGTTGGTCTTCAACAAAACCGTTAGGACGCTTAATGT
Rhl_R           AAAAGTCATGGCATGGTTACTGTTGTTGGTCTTCAACAAAACCGTTAGGACGCTTAATGT
                ************************************************************

Karat_F         AGAAGAAACTTGGCACAACCAAGAATTACTGTGTCAGAAGGCAATACTCTGTTCTCAAAT
Karat_R         AGAAGAAACTTGGCACAACCAAGAATTACTGTGTCAGAAGGCAATACTCTGTTCTCAAAT
Rhl_F           AGAAGAAACTTGGCACAACCAAGAATTACTGTGTCAGAAGGCAATACTCTGTTCTCAAAT
Rhl_R           AGAAGAAACTTGGCACAACCAAGAATTACTGTGTCAGAAGGCAATACTCTGTTCTCAAAT
                ************************************************************

Karat_F         ACTCTACCTGAAGTAGAACATGAAATACCTGAAGTAGGTGACGGGCTTCAGGATTATGCT
Karat_R         ACTCTACCTGAAGTAGAACATGAAATACCTGAAGTAGGTGACGGGCTTCAGGATTATGCT
Rhl_F           ACTCTACCTGAAGTAGAACATGAAATACCTGAAGTAGGTGACGGGCTTCAGGATTATGCT
Rhl_R           ACTCTACCTGAAGTAGAACATGAAATACCTGAAGTAGGTGACGGGCTTCAGGATTATGCT
                ************************************************************

Karat_F         CGCCGATTCCTCATTTTGGTTGATCTAACTTTATGTTTCTCTTGCAGGCGTGCCAGCAGC
Karat_R         CGCCGATTCCTCATTTTGGTTGATCTAACTTTATGTTTCTCTTGCAGGCGTGCCAGCAGC
Rhl_F           CGCCGATTCCTCATTTTGGTTGATCTAACTTTATGTTTCTCTTGCAGGCGTGCCAGCAGC
Rhl_R           CGCCGATTCCTCATTTTGGTTGATCTAACTTTATGTTTCTCTTGCAGGCGTGCCAGCAGC
                ************************************************************

Karat_F         AGATGCTCCGATAAGAATCAGCGGGGTTAACAAACGTGGTTGGAAGTACATAAAGGCGTC
Karat_R         AGATGCTCCGATAAGAATCAGCGGGGTTAACAAACGTGGTTGGAAGTACATAAAGGCGTC
Rhl_F           AGATGCTCCGATAAGAATCAGCGGGGTTAACAAACGTGGTTGGAAGTACATAAAGGCGTC
Rhl_R           AGATGCTCCGATAAGAATCAGCGGGGTTAACAAACGTGGTTGGAAGTACATAAAGGCGTC
                ************************************************************

Karat_F         TTTGCAAGATATCCCTGGAGCAGAGCCGCCAGCCGTCTCCGCTGCTTAACCAGTTACTGG
Karat_R         TTTGCAAGATATCCCTGGAGCAGAGCCGCCAGCCGTCTCCGCTGCTTAACCAGTTACTGG
Rhl_F           TTTGCAAGATATCCCTGGAGCAGAGCCGCCAGCCGTCTCCGCTGCTTAACCAGTTACTGG
Rhl_R           TTTGCAAGATATCCCTGGAGCAGAGCCGCCAGCCGTCTCCGCTGCTTAACCAGTTACTGG
                ************************************************************
```

```
Karat_F     CTAGATATTTCGTAGGTGATAAAACTGGTCCCAGTAGAAGCGTAGTGCTGAACTATTCAC
Karat_R     CTAGATATTTCGTAGGTGATAAAACTGGTCCCAGTAGAAGCGTAGTGCTGAACTATTCAC
Rhl_F       CTAGATATTTCGTAGGTGATAAAACTGGTCCCAGTAGAAGCGTAGTGCTGAACTATTCAC
Rhl_R       CTAGATATTTCGTAGGTGATAAAACTGGTCCCAGTAGAAGCGTAGTGCTGAACTATTCAC
            ************************************************************

Karat_F     AAGACTGGTTTGATCTGCCAGCTCCACGTGTTTCATAAGTCTCATGTCTCCACAAGGGGT
Karat_R     AAGACTGGTTTGATCTGCCAGCTCCACGTGTTTCATAAGTCTCATGTCTCCACAAGGGGT
Rhl_F       AAGACTGGTTTGATCTGCCAGCTCCACGTGTTTCATAAGTCTCATGTCTCCACAAGGGGT
Rhl_R       AAGACTGGTTTGATCTGCCAGCTCCACGTGTTTCATAAGTCTCATGTCTCCACAAGGGGT
            ************************************************************

Karat_F     TTAAACATGCTTGAACGTGTTTCGGCGTGAGCTTGTCATGGCCTCCACGCTTGTTGTTGA
Karat_R     TTAAACATGCTTGAACGTGTTTCGGCGTGAGCTTGTCATGGCCTCCACGCTTGTTGTTGA
Rhl_F       TTAAACATGCTTGAACGTGTTTCGGCGTGAGCTTGTCATGGCCTCCACGCTTGTTGTTGA
Rhl_R       TTAAACATGCTTGAACGTGTTTCGGCGTGAGCTTGTCATGGCCTCCACGCTTGTTGTTGA
            ************************************************************

Karat_F     AGCTAAAGATGATGTAGTGTTAATTTTGTTTTGTTTTAGATTTTATTTTTTGCAATGCCT
Karat_R     AGCTAAAGATGATGTAGTGTTAATTTTGTTTTGTTTTAGATTTTATTTTTTGCAATGCCT
Rhl_F       AGCTAAAGATGATGTAGTGTTAATTTTGTTTTGTTTTAGATTTTATTTTTTGCAATGCCT
Rhl_R       AGCTAAAGATGATGTAGTGTTAATTTTGTTTTGTTTTAGATTTTATTTTTTGCAATGCCT
            ************************************************************

Karat_F     CATTTCCATCAGAAGCAAGTGCTTCACCAACAATGACGAATCTCCATCGACGTCGAGGAC
Karat_R     CATTTCCATCAGAAGCAAGTGCTTCACCAACAATGACGAATCTCCATCGACGTCGAGGAC
Rhl_F       CATTTCCATCAGAAGCAAGTGCTTCACCAACAATGACGAATCTCCATCGACGTCGAGGAC
Rhl_R       CATTTCCATCAGAAGCAAGTGCTTCACCAACAATGACGAATCTCCATCGACGTCGAGGAC
            ************************************************************

Karat_F     ATGGCATTCCTGCAATGCCGCAGCTTGGCTCGGAGGCGATCGAGTGTCAAGTATGACAAT
Karat_R     ATGGCATTCCTGCAATGCCGCAGCTTGGCTCGGAGGCGATCGAGTGTCAAGTATGACAAT
Rhl_F       ATGGCATTCCTGCAATGCCGCAGCTTGGCTCGGAGGCGATCGAGTGTCAAGTATGACAAT
Rhl_R       ATGGCATTCCTGCAATGCCGCAGCTTGGCTCGGAGGCGATCGAGTGTCAAGTATGACAAT
            ************************************************************

Karat_F     TAGGTTAGACTCTAGCAAAGCAAGTTATGGTTTCCATTGTCTTTTTTCTTTTATCACTGA
Karat_R     TAGGTTAGACTCTAGCAAAGCAAGTTATGGTTTCCATTGTCTTTTTTCTTTTATCACTGA
Rhl_F       TAGGTTAGACTCTAGCAAAGCAAGTTATGGTTTCCATTGTCTTTTTTCTTTTATCACTGA
Rhl_R       TAGGTTAGACTCTAGCAAAGCAAGTTATGGTTTCCATTGTCTTTTTTCTTTTATCACTGA
            ************************************************************

Karat_F     CAGTTCATGTGAGTTCTGGCAAGGAATTCATCGCTCTGGAAAACGTTGTTATAACTCTGA
Karat_R     CAGTTCATGTGAGTTCTGGCAAGGAATTCATCGCTCTGGAAAACGTTGTTATAACTCTGA
Rhl_F       CAGTTCATGTGAGTTCTGGCAAGGAATTCATCGCTCTGGAAAACGTTGTTATAACTCTGA
Rhl_R       CAGTTCATGTGAGTTCTGGCAAGGAATTCATCGCTCTGGAAAACGTTGTTATAACTCTGA
            ************************************************************

Karat_F     GAATTCAGTCAAGGCCTGAACCTGTGAACCATCTATAACTCTGAACCTCGCTTGGTATAA
Karat_R     GAATTCAGTCAAGGCCTGAACCTGTGAACCATCTATAACTCTGAACCTCGCTTGGTATAA
Rhl_F       GAATTCAGTCAAGGCCTGAACCTGTGAACCATCTATAACTCTGAACCTCGCTTGGTATAA
Rhl_R       GAATTCAGTCAAGGCCTGAACCTGTGAACCATCTATAACTCTGAACCTCGCTTGGTATAA
            ************************************************************

Karat_F     ACAACGTCGATGACCAGCGAATGGTGAACGCCAGCACAAATCACAATGCGGCATAGATTA
Karat_R     ACAACGTCGATGACCAGCGAATGGTGAACGCCAGCACAAATCACAATGCGGCATAGATTA
Rhl_F       ACAACGTCGATGACCAGCGAATGGTGAACGCCAGCACAAATCACAATGCGGCATAGATTA
Rhl_R       ACAACGTCGATGACCAGCGAATGGTGAACGCCAGCACAAATCACAATGCGGCATAGATTA
            ************************************************************

Karat_F     ACCGATCAAGAGAAGACGCTGGTCCTGTCCGTTTTCCATTGCGAGGGGTTACTTATACAT
Karat_R     ACCGATCAAGAGAAGACGCTGGTCCTGTCCGTTTTCCATTGCGAGGGGTTACTTATACAT
Rhl_F       ACCGATCAAGAGAAGACGCTGGTCCTGTCCGTTTTCCATTGCGAGGGGTTACTTATACAT
Rhl_R       ACCGATCAAGAGAAGACGCTGGTCCTGTCCGTTTTCCATTGCGAGGGGTTACTTATACAT
            ************************************************************

Karat_F     TCTGAAGCCCATTTCCGAGCACGTGAAATCTGCCTGTGTTCGGACACGCTCTGCCTGCCT
Karat_R     TCTGAAGCCCATTTCCGAGCACGTGAAATCTGCCTGTGTTCGGACACGCTCTGCCTGCCT
Rhl_F       TCTGAAGCCCATTTCCGAGCACGTGAAATCTGCCTGTGTTCGGACACGCTCTGCCTGCCT
```

```
Rhl_R       TCTGAAGCCCATTTCCGAGCACGTGAAATCTGCCTGTGTTCGGACACGCTCTGCCTGCCT
            ************************************************************

Karat_F     GCCTACCGCGGCACCGGACTGGGAGCAGGGCAGAGCGGCTACAGCTTCCCTTCTATTCTT
Karat_R     GCCTACCGCGGCACCGGACTGGGAGCAGGGCAGAGCGGCTACAGCTTCCCTTCTATTCTT
Rhl_F       GCCTACCGCGGCACCGGACTGGGAGCAGGGCAGAGCGGCTACAGCTTCCCTTCTATTCTT
Rhl_R       GCCTACCGCGGCACCGGACTGGGAGCAGGGCAGAGCGGCTACAGCTTCCCTTCTATTCTT
            ************************************************************

Karat_F     TCTTCCACCTCTGTCACTCTGTCTGTTTCTTTTAACGGCCCGTCGATCTGCTCTTTTGCT
Karat_R     TCTTCCACCTCTGTCACTCTGTCTGTTTCTTTTAACGGCCCGTCGATCTGCTCTTTTGCT
Rhl_F       TCTTCCACCTCTGTCACTCTGTCTGTTTCTTTTAACGGCCCGTCGATCTGCTCTTTTGCT
Rhl_R       TCTTCCACCTCTGTCACTCTGTCTGTTTCTTTTAACGGCCCGTCGATCTGCTCTTTTGCT
            ************************************************************

Karat_F     GCCCATGCAAACACAGGGACATGCCACTGTAGCACGGCGTGTTTGCTGCCAATCTGCTGC
Karat_R     GCCCATGCAAACACAGGGACATGCCACTGTAGCACGGCGTGTTTGCTGCCAATCTGCTGC
Rhl_F       GCCCATGCAAACACAGGGACATGCCACTGTAGCACGGCGTGTTTGCTGCCAATCTGCTGC
Rhl_R       GCCCATGCAAACACAGGGACATGCCACTGTAGCACGGCGTGTTTGCTGCCAATCTGCTGC
            ************************************************************

Karat_F     GCCGCAGTCCCCCCAGTAA
Karat_R     GCCGCAGTCCCCCCAGTAA
Rhl_F       GCCGCAGTCCCCCCAGTAA
Rhl_R       GCCGCAGTCCCCCCAGTAA
            *******************
```

Alignment_HORVU7Hr1G030290

```
Karat_F      GGTGCCGATCTGGTCCGTCCGATCCCCGATCCGACGGCTGAGGAGACATAGTCCCAGGCC
Karat_R      GGTGCCGATCTGGTCCGTCCGATCCCCGATCCGACGGCTGAGGAGACATAGTCCCAGGCC
Rhl_F        GGTGCCGATCTGGTCCGTCCGATCCCCGATCCGACGGCTGAGGAGACATAGTCCCAGGCC
Rhl_R        GGTGCCGATCTGGTCCGTCCGATCCCCGATCCGACGGCTGAGGAGACATAGTCCCAGGCC
             ************************************************************

Karat_F      CCAAAAACGCTCCACCTATACATGCATCCCCGCCTGACACCTTCCATTTCCATTTCCAAG
Karat_R      CCAAAAACGCTCCACCTATACATGCATCCCCGCCTGACACCTTCCATTTCCATTTCCAAG
Rhl_F        CCAAAAACGCTCCACCTATACATGCATCCCCGCCTGACACCTTCCATTTCCATTTCCAAG
Rhl_R        CCAAAAACGCTCCACCTATACATGCATCCCCGCCTGACACCTTCCATTTCCATTTCCAAG
             ************************************************************

Karat_F      AAGCTGCGAGCCTGCAGAGCTGCACGCCATGGCAGCACTGCACTGCACTGCCCTGCCATG
Karat_R      AAGCTGCGAGCCTGCAGAGCTGCACGCCATGGCAGCACTGCACTGCACTGCCCTGCCATG
Rhl_F        AAGCTGCGAGCCTGCAGAGCTGCACGCCATGGCAGCACTGCACTGCACTGCCCTGCCATG
Rhl_R        AAGCTGCGAGCCTGCAGAGCTGCACGCCATGGCAGCACTGCACTGCACTGCCCTGCCATG
             ************************************************************

Karat_F      GTAGTACTCCCACTATTCCCTCCCGCAGAGCTGCGCCGAAAATTCCCTTCTTCCCATTCA
Karat_R      GTAGTACTCCCACTATTCCCTCCCGCAGAGCTGCGCCGAAAATTCCCTTCTTCCCATTCA
Rhl_F        GTAGTACTCCCACTATTCCCTCCCGCAGAGCTGCGCCGAAAATTCCCTTCTTCCCATTCA
Rhl_R        GTAGTACTCCCACTATTCCCTCCCGCAGAGCTGCGCCGAAAATTCCCTTCTTCCCATTCA
             ************************************************************

Karat_F      GCACAGGCGCAGGCGCAGCAGCTGTCACGCCGGCCTGCTCTGCGCGGCGCACACGCGCGC
Karat_R      GCACAGGCGCAGGCGCAGCAGCTGTCACGCCGGCCTGCTCTGCGCGGCGCACACGCGCGC
Rhl_F        GCACAGGCGCAGGCGCAGCAGCTGTCACGCCGGCCTGCTCTGCGCGGCGCACACGCGCGC
Rhl_R        GCACAGGCGCAGGCGCAGCAGCTGTCACGCCGGCCTGCTCTGCGCGGCGCACACGCGCGC
             ************************************************************

Karat_F      GCGCTCTGGCGCGAGCTGCACGGCGCGTCAGTAACTCGCAGAATCACAGCTTCCATTTCA
Karat_R      GCGCTCTGGCGCGAGCTGCACGGCGCGTCAGTAACTCGCAGAATCACAGCTTCCATTTCA
Rhl_F        GCGCTCTGGCGCGAGCTGCACGGCGCGTCAGTAACTCGCAGAATCACAGCTTCCATTTCA
Rhl_R        GCGCTCTGGCGCGAGCTGCACGGCGCGTCAGTAACTCGCAGAATCACAGCTTCCATTTCA
             ************************************************************

Karat_F      AAAACAAAAGGTAAAAATGGAAGCCCGCCACAGTCGCCCGGCGTCAGTGCCCCGCTCGTA
Karat_R      AAAACAAAAGGTAAAAATGGAAGCCCGCCACAGTCGCCCGGCGTCAGTGCCCCGCTCGTA
Rhl_F        AAAACAAAAGGTAAAAATGGAAGCCCGCCACAGTCGCCCGGCGTCAGTGCCCCGCTCGTA
Rhl_R        AAAACAAAAGGTAAAAATGGAAGCCCGCCACAGTCGCCCGGCGTCAGTGCCCCGCTCGTA
             ************************************************************

Karat_F      CCGCCTCCGCCGCCTTCACTCCGGCGCATGGGTTGCCCGCCCCCGCACTTAATTCCCGGC
Karat_R      CCGCCTCCGCCGCCTTCACTCCGGCGCATGGGTTGCCCGCCCCCGCACTTAATTCCCGGC
Rhl_F        CCGCCTCCGCCGCCTTCACTCCGGCGCATGGGTTGCCCGCCCCCGCACTTAATTCCCGGC
Rhl_R        CCGCCTCCGCCGCCTTCACTCCGGCGCATGGGTTGCCCGCCCCCGCACTTAATTCCCGGC
             ************************************************************

Karat_F      CGGCCTGCGCTTTCAGTTAAGGTACCTCCACGTACCGCAGTACGGGCCCGGGTCCATCGA
Karat_R      CGGCCTGCGCTTTCAGTTAAGGTACCTCCACGTACCGCAGTACGGGCCCGGGTCCATCGA
Rhl_F        CGGCCTGCGCTTTCAGTTAAGGTACCTCCACGTACCGCAGTACGGGCCCGGGTCCATCGA
Rhl_R        CGGCCTGCGCTTTCAGTTAAGGTACCTCCACGTACCGCAGTACGGGCCCGGGTCCATCGA
             ************************************************************

Karat_F      TCGCCATTAGCTTAGCTCCCCCGTCGCCATGATTAATGGCGCCCGGGCTAGCTTCCAAAA
Karat_R      TCGCCATTAGCTTAGCTCCCCCGTCGCCATGATTAATGGCGCCCGGGCTAGCTTCCAAAA
Rhl_F        TCGCCATTAGCTTAGCTCCCCCGTCGCCATGATTAATGGCGCCCGGGCTAGCTTCCAAAA
Rhl_R        TCGCCATTAGCTTAGCTCCCCCGTCGCCATGATTAATGGCGCCCGGGCTAGCTTCCAAAA
             ************************************************************

Karat_F      CCCGATCCATTGGCTCGATCCGAACGGAATGCTGCCGCATTTACTCGCCTGCTTTTTTGA
Karat_R      CCCGATCCATTGGCTCGATCCGAACGGAATGCTGCCGCATTTACTCGCCTGCTTTTTTGA
Rhl_F        CCCGATCCATTGGCTCGATCCGAACGGAATGCTGCCGCATTTACTCGCCTGCTTTTTTGA
Rhl_R        CCCGATCCATTGGCTCGATCCGAACGGAATGCTGCCGCATTTACTCGCCTGCTTTTTTGA
             ************************************************************
```

```
Karat_F      ATGCGTGCTGCGATTTCAGCTTGCCGCGCATGCAGCCGTGTCCAGCCGCCGGGTGTTCGG
Karat_R      ATGCGTGCTGCGATTTCAGCTTGCCGCGCATGCAGCCGTGTCCAGCCGCCGGGTGTTCGG
Rhl_F        ATGCGTGCTGCGATTTCAGCTTGCCGCGCATGCAGCCGTGTCCAGCCGCCGGGTGTTCGG
Rhl_R        ATGCGTGCTGCGATTTCAGCTTGCCGCGCATGCAGCCGTGTCCAGCCGCCGGGTGTTCGG
             ************************************************************

Karat_F      TGTTCGGATGGATGATGCTTCGCTCTGCTCCTAGTAGCAGCCTCCTATAAATACCGGGCG
Karat_R      TGTTCGGATGGATGATGCTTCGCTCTGCTCCTAGTAGCAGCCTCCTATAAATACCGGGCG
Rhl_F        TGTTCGGATGGATGATGCTTCGCTCTGCTCCTAGTAGCAGCCTCCTATAAATACCGGGCG
Rhl_R        TGTTCGGATGGATGATGCTTCGCTCTGCTCCTAGTAGCAGCCTCCTATAAATACCGGGCG
             ************************************************************

Karat_F      TCGTCGTGCATCGTGCATCGATCGATCGCCAACAATTCGAATTGTCCATCTCCCGCCACG
Karat_R      TCGTCGTGCATCGTGCATCGATCGATCGCCAACAATTCGAATTGTCCATCTCCCGCCACG
Rhl_F        TCGTCGTGCATCGTGCATCGATCGATCGCCAACAATTCGAATTGTCCATCTCCCGCCACG
Rhl_R        TCGTCGTGCATCGTGCATCGATCGATCGCCAACAATTCGAATTGTCCATCTCCCGCCACG
             ************************************************************

Karat_F      CGCACCCACCCACCTACGGGAATAAGCAAGCAAGCTGGTCACTGGGGTGACGAACTAACG
Karat_R      CGCACCCACCCACCTACGGGAATAAGCAAGCAAGCTGGTCACTGGGGTGACGAACTAACG
Rhl_F        CGCACCCACCCACCTACGGGAATAAGCAAGCAAGCTGGTCACTGGGGTGACGAACTAACG
Rhl_R        CGCACCCACCCACCTACGGGAATAAGCAAGCAAGCTGGTCACTGGGGTGACGAACTAACG
             ************************************************************

Karat_F      ATCGATGGCCCGTTTGGTTAGAGACGGCGGTCGTGGACGAGGGGTGGCGGCGGTGGAGGA
Karat_R      ATCGATGGCCCGTTTGGTTAGAGACGGCGGTCGTGGACGAGGGGTGGCGGCGGTGGAGGA
Rhl_F        ATCGATGGCCCGTTTGGTTAGAGACGGCGGTCGTGGACGAGGGGTGGCGGCGGTGGAGGA
Rhl_R        ATCGATGGCCCGTTTGGTTAGAGACGGCGGTCGTGGACGAGGGGTGGCGGCGGTGGAGGA
             ************************************************************

Karat_F      GATGGCGCCGCCACTGCCTCTGCCTCCGCCGCCGCGCCTCGCGTCGGCGGCGTTGTCTTC
Karat_R      GATGGCGCCGCCACTGCCTCTGCCTCCGCCGCCGCGCCTCGCGTCGGCGGCGTTGTCTTC
Rhl_F        GATGGCGCCGCCACTGCCTCTGCCTCCGCCGCCGCGCCTCGCGTCGGCGGCGTTGTCTTC
Rhl_R        GATGGCGCCGCCACTGCCTCTGCCTCCGCCGCCGCGCCTCGCGTCGGCGGCGTTGTCTTC
             ************************************************************

Karat_F      CTCGTCGCCGGCGTCCATCCGTGCGCTGCTGGCGAGGACCGGGGGCGGGGCGGACTGCCA
Karat_R      CTCGTCGCCGGCGTCCATCCGTGCGCTGCTGGCGAGGACCGGGGGCGGGGCGGACTGCCA
Rhl_F        CTCGTCGCCGGCGTCCATCCGTGCGCTGCTGGCGAGGACCGGGGGCGGGGCGGACTGCCA
Rhl_R        CTCGTCGCCGGCGTCCATCCGTGCGCTGCTGGCGAGGACCGGGGGCGGGGCGGACTGCCA
             ************************************************************

Karat_F      GCAGTCGCCGCGGTCGCTGCTGTCCCGCATCCTGCTGCGCGGCGGCGGCGATCATCACGG
Karat_R      GCAGTCGCCGCGGTCGCTGCTGTCCCGCATCCTGCTGCGCGGCGGCGGCGATCATCACGG
Rhl_F        GCAGTCGCCGCGGTCGCTGCTGTCCCGCATCCTGCTGCGCGGCGGCGGCGATCATCACGG
Rhl_R        GCAGTCGCCGCGGTCGCTGCTGTCCCGCATCCTGCTGCGCGGCGGCGGCGATCATCACGG
             ************************************************************

Karat_F      CGGGAACGGGGGAGGGTCGTTCGGGTGCCGGGTCAGGCTTCCGCGGCGGTACGGTAGTAG
Karat_R      CGGGAACGGGGGAGGGTCGTTCGGGTGCCGGGTCAGGCTTCCGCGGCGGTACGGTAGTAG
Rhl_F        CGGGAACGGGGGAGGGTCGTTCGGGTGCCGGGTCAGGCTTCCGCGGCGGTACGGTAGTAG
Rhl_R        CGGGAACGGGGGAGGGTCGTTCGGGTGCCGGGTCAGGCTTCCGCGGCGGTACGGTAGTAG
             ************************************************************

Karat_F      CAGCTCGTCCGTCGGCGACAGCATCAGGGAGGAGAGGAAGGACGACGGCGCTGCCTCCGA
Karat_R      CAGCTCGTCCGTCGGCGACAGCATCAGGGAGGAGAGGAAGGACGACGGCGCTGCCTCCGA
Rhl_F        CAGCTCGTCCGTCGGCGACAGCATCAGGGAGGAGAGGAAGGACGACGGCGCTGCCTCCGA
Rhl_R        CAGCTCGTCCGTCGGCGACAGCATCAGGGAGGAGAGGAAGGACGACGGCGCTGCCTCCGA
             ************************************************************

Karat_F      GCAGTCGGCGGACGACGTCGGCTCCGCCAGGGTCAAGGTTGTCGAGCGCTCGCCGGAGCT
Karat_R      GCAGTCGGCGGACGACGTCGGCTCCGCCAGGGTCAAGGTTGTCGAGCGCTCGCCGGAGCT
Rhl_F        GCAGTCGGCGGACGACGTCGGCTCCGCCAGGGTCAAGGTTGTCGAGCGCTCGCCGGAGCT
Rhl_R        GCAGTCGGCGGACGACGTCGGCTCCGCCAGGGTCAAGGTTGTCGAGCGCTCGCCGGAGCT
             ************************************************************

Karat_F      GCCCGTCGACACGCCCCGGAGCTCCCTAGGTAATTGCCATATAGATCGTCTACGTACAGT
Karat_R      GCCCGTCGACACGCCCCGGAGCTCCCTAGGTAATTGCCATATAGATCGTCTACGTACAGT
Rhl_F        GCCCGTCGACACGCCCCGGAGCTCCCTAGGTAATTGCCATATAGATCGTCTACGTACAGT
```

```
Rhl_R        GCCCGTCGACACGCCCCGGAGCTCCCTAGGTAATTGCCATATAGATCGTCTACGTACAGT
             ************************************************************

Karat_F      TCGTAGCGATTAGTAGATTAGATGATGTTGATCGGAACTTGTGGATGATCGATGCAGGCA
Karat_R      TCGTAGCGATTAGTAGATTAGATGATGTTGATCGGAACTTGTGGATGATCGATGCAGGCA
Rhl_F        TCGTAGCGATTAGTAGATTAGATGATGTTGATCGGAACTTGTGGATGATCGATGCAGGCA
Rhl_R        TCGTAGCGATTAGTAGATTAGATGATGTTGATCGGAACTTGTGGATGATCGATGCAGGCA
             ************************************************************

Karat_F      AGAAGAAGCCGGAGGAGGAGGTCATGTCGATGAGCCTTCGGCTGGGCGCGAGCCTGGTGC
Karat_R      AGAAGAAGCCGGAGGAGGAGGTCATGTCGATGAGCCTTCGGCTGGGCGCGAGCCTGGTGC
Rhl_F        AGAAGAAGCCGGAGGAGGAGGTCATGTCGATGAGCCTTCGGCTGGGCGCGAGCCTGGTGC
Rhl_R        AGAAGAAGCCGGAGGAGGAGGTCATGTCGATGAGCCTTCGGCTGGGCGCGAGCCTGGTGC
             ************************************************************

Karat_F      TGCTGCTCTCCAAGAGCGCGGTGGAGCTGAACAAGATGGTGGAGCTCCGCGCGCAGATGG
Karat_R      TGCTGCTCTCCAAGAGCGCGGTGGAGCTGAACAAGATGGTGGAGCTCCGCGCGCAGATGG
Rhl_F        TGCTGCTCTCCAAGAGCGCGGTGGAGCTGAACAAGATGGTGGAGCTCCGCGCGCAGATGG
Rhl_R        TGCTGCTCTCCAAGAGCGCGGTGGAGCTGAACAAGATGGTGGAGCTCCGCGCGCAGATGG
             ************************************************************

Karat_F      AGGCGCTCGTGTCGGAGATCAGGCACGAGACCATCGGGAAGGAGAAGCACGGCGGCTCTG
Karat_R      AGGCGCTCGTGTCGGAGATCAGGCACGAGACCATCGGGAAGGAGAAGCACGGCGGCTCTG
Rhl_F        AGGCGCTCGTGTCGGAGATCAGGCACGAGACCATCGGGAAGGAGAAGCACGGCGGCTCTG
Rhl_R        AGGCGCTCGTGTCGGAGATCAGGCACGAGACCATCGGGAAGGAGAAGCACGGCGGCTCTG
             ************************************************************

Karat_F      CTCCGGCCGCCTCGTCCTCCTCCTCCCAGGAGTCCACCGTGATCAAGGACCCCATCGCCC
Karat_R      CTCCGGCCGCCTCGTCCTCCTCCTCCCAGGAGTCCACCGTGATCAAGGACCCCATCGCCC
Rhl_F        CTCCGGCCGCCTCGTCCTCCTCCTCCCAGGAGTCCACCGTGATCAAGGACCCCATCGCCC
Rhl_R        CTCCGGCCGCCTCGTCCTCCTCCTCCCAGGAGTCCACCGTGATCAAGGACCCCATCGCCC
             ************************************************************

Karat_F      GCGCCGAGGACGCGCTGTCCGGCAACTGCTCCGGCGCCCGGACCGCTGATCGCCGCCAGC
Karat_R      GCGCCGAGGACGCGCTGTCCGGCAACTGCTCCGGCGCCCGGACCGCTGATCGCCGCCAGC
Rhl_F        GCGCCGAGGACGCGCTGTCCGGCAACTGCTCCGGCGCCCGGACCGCTGATCGCCGCCAGC
Rhl_R        GCGCCGAGGACGCGCTGTCCGGCAACTGCTCCGGCGCCCGGACCGCTGATCGCCGCCAGC
             ************************************************************

Karat_F      TTTCCGCCGCCGTCGTCGCGATGGACCATAACAAGATGGAGGCCGAGCTCCAGCTCGAGC
Karat_R      TTTCCGCCGCCGTCGTCGCGATGGACCATAACAAGATGGAGGCCGAGCTCCAGCTCGAGC
Rhl_F        TTTCCGCCGCCGTCGTCGCGATGGACCATAACAAGATGGAGGCCGAGCTCCAGCTCGAGC
Rhl_R        TTTCCGCCGCCGTCGTCGCGATGGACCATAACAAGATGGAGGCCGAGCTCCAGCTCGAGC
             ************************************************************

Karat_F      TGAGCCGCATGCAGGCGCAGCATCGCGCCATGCACGCGCCCATCAGAGGGCTCGAGGTAC
Karat_R      TGAGCCGCATGCAGGCGCAGCATCGCGCCATGCACGCGCCCATCAGAGGGCTCGAGGTAC
Rhl_F        TGAGCCGCATGCAGGCGCAGCATCGCGCCATGCACGCGCCCATCAGAGGGCTCGAGGTAC
Rhl_R        TGAGCCGCATGCAGGCGCAGCATCGCGCCATGCACGCGCCCATCAGAGGGCTCGAGGTAC
             ************************************************************

Karat_F      GTAGCGTACGTACGATCGAGTGTACCGCACGGCATATCTTGGTATCTTGAGTAGCACGTA
Karat_R      GTAGCGTACGTACGATCGAGTGTACCGCACGGCATATCTTGGTATCTTGAGTAGCACGTA
Rhl_F        GTAGCGTACGTACGATCGAGTGTACCGCACGGCATATCTTGGTATCTTGAGTAGCACGTA
Rhl_R        GTAGCGTACGTACGATCGAGTGTACCGCACGGCATATCTTGGTATCTTGAGTAGCACGTA
             ************************************************************

Karat_F      CTCCATGCATGCATGCATGCACAGCGAATTGCGTCCCGTTCCCGCGCTTGCTTGCTGATG
Karat_R      CTCCATGCATGCATGCATGCACAGCGAATTGCGTCCCGTTCCCGCGCTTGCTTGCTGATG
Rhl_F        CTCCATGCATGCATGCATGCACAGCGAATTGCGTCCCGTTCCCGCGCTTGCTTGCTGATG
Rhl_R        CTCCATGCATGCATGCATGCACAGCGAATTGCGTCCCGTTCCCGCGCTTGCTTGCTGATG
             ************************************************************

Karat_F      ATGCACTTACGCGTCTTCTCTTTTTGGCCAGCTGCCGCCGCTGCAGGTGAAGACGGCGAG
Karat_R      ATGCACTTACGCGTCTTCTCTTTTTGGCCAGCTGCCGCCGCTGCAGGTGAAGACGGCGAG
Rhl_F        ATGCACTTACGCGTCTTCTCTTTTTGGCCAGCTGCCGCCGCTGCAGGTGAAGACGGCGAG
Rhl_R        ATGCACTTACGCGTCTTCTCTTTTTGGCCAGCTGCCGCCGCTGCAGGTGAAGACGGCGAG
             ************************************************************
```

```
Karat_F      GAGCGCGCACGTGTCCGTCGACACGACGTCGAGGAGCTGCGTCGTCGACCACGCGACGCA
Karat_R      GAGCGCGCACGTGTCCGTCGACACGACGTCGAGGAGCTGCGTCGTCGACCACGCGACGCA
Rhl_F        GAGCGCGCACGTGTCCGTCGACACGACGTCGAGGAGCTGCGTCGTCGACCACGCGACGCA
Rhl_R        GAGCGCGCACGTGTCCGTCGACACGACGTCGAGGAGCTGCGTCGTCGACCACGCGACGCA
             ************************************************************

Karat_F      GGTGAACGCCGACGAGGAGGACGGCGAGGACGAAGAGGATCAGCGCGAGGAGGACTACGA
Karat_R      GGTGAACGCCGACGAGGAGGACGGCGAGGACGAAGAGGATCAGCGCGAGGAGGACTACGA
Rhl_F        GGTGAACGCCGACGAGGAGGACGGCGAGGACGAAGAGGATCAGCGCGAGGAGGACTACGA
Rhl_R        GGTGAACGCCGACGAGGAGGACGGCGAGGACGAAGAGGATCAGCGCGAGGAGGACTACGA
             ************************************************************

Karat_F      CGAGGAGGAGGAGGGCGACGACGACGACGACGGTGGCGAGGTGGTGGACCGCGACAGGAG
Karat_R      CGAGGAGGAGGAGGGCGACGACGACGACGACGGTGGCGAGGTGGTGGACCGCGACAGGAG
Rhl_F        CGAGGAGGAGGAGGGCGACGACGACGACGACGGTGGCGAGGTGGTGGACCGCGACAGGAG
Rhl_R        CGAGGAGGAGGAGGGCGACGACGACGACGACGGTGGCGAGGTGGTGGACCGCGACAGGAG
             ************************************************************

Karat_F      CCCCCCGCACGGCGGCGTGTCGGCGCGCGCGCTGGAACGGCGGCTGCACGAGCTGCTGCA
Karat_R      CCCCCCGCACGGCGGCGTGTCGGCGCGCGCGCTGGAACGGCGGCTGCACGAGCTGCTGCA
Rhl_F        CCCCCCGCACGGCGGCGTGTCGGCGCGCGCGCTGGAACGGCGGCTGCACGAGCTGCTGCA
Rhl_R        CCCCCCGCACGGCGGCGTGTCGGCGCGCGCGCTGGAACGGCGGCTGCACGAGCTGCTGCA
             ************************************************************

Karat_F      GCGGCGGCAGCAGGACCGCATCGTGGAGCTGGAGGCGGCGCTGGACGGCGCCCAGCGGCG
Karat_R      GCGGCGGCAGCAGGACCGCATCGTGGAGCTGGAGGCGGCGCTGGACGGCGCCCAGCGGCG
Rhl_F        GCGGCGGCAGCAGGACCGCATCGTGGAGCTGGAGGCGGCGCTGGACGGCGCCCAGCGGCG
Rhl_R        GCGGCGGCAGCAGGACCGCATCGTGGAGCTGGAGGCGGCGCTGGACGGCGCCCAGCGGCG
             ************************************************************

Karat_F      GCTCCAGGAGCGGGAGCGCGAGGTGGTGTGGTGGCGCGACGCCGCAAAGCTCGTGTCCCA
Karat_R      GCTCCAGGAGCGGGAGCGCGAGGTGGTGTGGTGGCGCGACGCCGCAAAGCTCGTGTCCCA
Rhl_F        GCTCCAGGAGCGGGAGCGCGAGGTGGTGTGGTGGCGCGACGCCGCAAAGCTCGTGTCCCA
Rhl_R        GCTCCAGGAGCGGGAGCGCGAGGTGGTGTGGTGGCGCGACGCCGCAAAGCTCGTGTCCCA
             ************************************************************

Karat_F      CCGCCGCGACGAGTCCCGCCGCCTCAGGTGCACCGCCCCCGAGCCGGTCCGATAAGCACA
Karat_R      CCGCCGCGACGAGTCCCGCCGCCTCAGGTGCACCGCCCCCGAGCCGGTCCGATAAGCACA
Rhl_F        CCGCCGCGACGAGTCCCGCCGCCTCAGGTGCACCGCCCCCGAGCCGGTCCGATAAGCACA
Rhl_R        CCGCCGCGACGAGTCCCGCCGCCTCAGGTGCACCGCCCCCGAGCCGGTCCGATAAGCACA
             ************************************************************

Karat_F      CCCACACGCACACGCACGCGCACACGCGCGTGTCAGCTCTCGGCTCACGTGTGCGTGGCA
Karat_R      CCCACACGCACACGCACGCGCACACGCGCGTGTCAGCTCTCGGCTCACGTGTGCGTGGCA
Rhl_F        CCCACACGCACACGCACGCGCACACGCGCGTGTCAGCTCTCGGCTCACGTGTGCGTGGCA
Rhl_R        CCCACACGCACACGCACGCGCACACGCGCGTGTCAGCTCTCGGCTCACGTGTGCGTGGCA
             ************************************************************

Karat_F      TGGCAGCTCTAGCTAGCGAGTAATGGCGGGGCAGCCGCCATGTCAGGGTGTGTGCAGTCG
Karat_R      TGGCAGCTCTAGCTAGCGAGTAATGGCGGGGCAGCCGCCATGTCAGGGTGTGTGCAGTCG
Rhl_F        TGGCAGCTCTAGCTAGCGAGTAATGGCGGGGCAGCCGCCATGTCAGGGTGTGTGCAGTCG
Rhl_R        TGGCAGCTCTAGCTAGCGAGTAATGGCGGGGCAGCCGCCATGTCAGGGTGTGTGCAGTCG
             ************************************************************

Karat_F      GGCTTGTGGAGGGAGAGGAGTGGAGGTCTCCTGGTCACGCTGCGCGGGTGAGGCGGCGTT
Karat_R      GGCTTGTGGAGGGAGAGGAGTGGAGGTCTCCTGGTCACGCTGCGCGGGTGAGGCGGCGTT
Rhl_F        GGCTTGTGGAGGGAGAGGAGTGGAGGTCTCCTGGTCACGCTGCGCGGGTGAGGCGGCGTT
Rhl_R        GGCTTGTGGAGGGAGAGGAGTGGAGGTCTCCTGGTCACGCTGCGCGGGTGAGGCGGCGTT
             ************************************************************

Karat_F      AATAACGGCCCCCGATCGGGCTCGGTTTGGCGCGCAACCGCGCGCGCGCAGGCGCTCGGA
Karat_R      AATAACGGCCCCCGATCGGGCTCGGTTTGGCGCGCAACCGCGCGCGCGCAGGCGCTCGGA
Rhl_F        AATAACGGCCCCCGATCGGGCTCGGTTTGGCGCGCAACCGCGCGCGCGCAGGCGCTCGGA
Rhl_R        AATAACGGCCCCCGATCGGGCTCGGTTTGGCGCGCAACCGCGCGCGCGCAGGCGCTCGGA
             ************************************************************

Karat_F      GATGGCGGGAAATGGGTGCGTGTGTGGGGTCTGCGCTGTAATAACAACCATTGTTCAGAG
Karat_R      GATGGCGGGAAATGGGTGCGTGTGTGGGGTCTGCGCTGTAATAACAACCATTGTTCAGAG
Rhl_F        GATGGCGGGAAATGGGTGCGTGTGTGGGGTCTGCGCTGTAATAACAACCATTGTTCAGAG
```

```
Rhl_R        GATGGCGGGAAATGGGTGCGTGTGTGGGGTCTGCGCTGTAATAACAACCATTGTTCAGAG
             ************************************************************

Karat_F      TTTTTGTTTCGGCTGTCCTGCTGCCCGATTGATTGCTTGCTAGCGTCGATGCGTCCTTCG
Karat_R      TTTTTGTTTCGGCTGTCCTGCTGCCCGATTGATTGCTTGCTAGCGTCGATGCGTCCTTCG
Rhl_F        TTTTTGTTTCGGCTGTCCTGCTGCCCGATTGATTGCTTGCTAGCGTCGATGCGTCCTTCG
Rhl_R        TTTTTGTTTCGGCTGTCCTGCTGCCCGATTGATTGCTTGCTAGCGTCGATGCGTCCTTCG
             ************************************************************

Karat_F      TTTTATTTATTATATTATATATGCTCCTACGTATGTTTCCAAATTCTGCGAGGGAAAGAC
Karat_R      TTTTATTTATTATATTATATATGCTCCTACGTATGTTTCCAAATTCTGCGAGGGAAAGAC
Rhl_F        TTTTATTTATTATATTATATATGCTCCTACGTATGTTTCCAAATTCTGCGAGGGAAAGAC
Rhl_R        TTTTATTTATTATATTATATATGCTCCTACGTATGTTTCCAAATTCTGCGAGGGAAAGAC
             ************************************************************

Karat_F      AGAAGAGCTGGGGCCTGGTTTCAGAAACCAGAATTTCGGTTTCTGAAGCTCTGCTCTGAT
Karat_R      AGAAGAGCTGGGGCCTGGTTTCAGAAACCAGAATTTCGGTTTCTGAAGCTCTGCTCTGAT
Rhl_F        AGAAGAGCTGGGGCCTGGTTTCAGAAACCAGAATTTCGGTTTCTGAAGCTCTGCTCTGAT
Rhl_R        AGAAGAGCTGGGGCCTGGTTTCAGAAACCAGAATTTCGGTTTCTGAAGCTCTGCTCTGAT
             ************************************************************

Karat_F      TTTTCCAAATTCTGCGAGGGGCAGCTTGTTTCCGAACCTGTTCTGAGCTCTGCTGCGTTC
Karat_R      TTTTCCAAATTCTGCGAGGGGCAGCTTGTTTCCGAACCTGTTCTGAGCTCTGCTGCGTTC
Rhl_F        TTTTCCAAATTCTGCGAGGGGCAGCTTGTTTCCGAACCTGTTCTGAGCTCTGCTGCGTTC
Rhl_R        TTTTCCAAATTCTGCGAGGGGCAGCTTGTTTCCGAACCTGTTCTGAGCTCTGCTGCGTTC
             ************************************************************

Karat_F      TGGTTCCAAATTCAGCGAGGGAAAAACGGAGCTGGGGCCTGGTTTCTGAATTTGGTTCTG
Karat_R      TGGTTCCAAATTCAGCGAGGGAAAAACGGAGCTGGGGCCTGGTTTCTGAATTTGGTTCTG
Rhl_F        TGGTTCCAAATTCAGCGAGGGAAAAACGGAGCTGGGGCCTGGTTTCTGAATTTGGTTCTG
Rhl_R        TGGTTCCAAATTCAGCGAGGGAAAAACGGAGCTGGGGCCTGGTTTCTGAATTTGGTTCTG
             ************************************************************

Karat_F      GGCTCTGCTCATGCTCCAAATTCTGGGAGGGAAAATGGGCAGGGACCAGGTATATACTAG
Karat_R      GGCTCTGCTCATGCTCCAAATTCTGGGAGGGAAAATGGGCAGGGACCAGGTATATACTAG
Rhl_F        GGCTCTGCTCATGCTCCAAATTCTGGGAGGGAAAATGGGCAGGGACCAGGTATATACTAG
Rhl_R        GGCTCTGCTCATGCTCCAAATTCTGGGAGGGAAAATGGGCAGGGACCAGGTATATACTAG
             ************************************************************

Karat_F      CGTAGTACATTATCTCGAGCAGGAACTGTCTGATTCTAGCCGAATTTACTCGGGTGCTGC
Karat_R      CGTAGTACATTATCTCGAGCAGGAACTGTCTGATTCTAGCCGAATTTACTCGGGTGCTGC
Rhl_F        CGTAGTACATTATCTCGAGCAGGAACTGTCTGATTCTAGCCGAATTTACTCGGGTGCTGC
Rhl_R        CGTAGTACATTATCTCGAGCAGGAACTGTCTGATTCTAGCCGAATTTACTCGGGTGCTGC
             ************************************************************

Karat_F      GGCTCAGCAGATTGGCTGCTGCTGCTGTTGCGGTGTGTCCTTGGGGCTGGCCGGCTGGGG
Karat_R      GGCTCAGCAGATTGGCTGCTGCTGCTGTTGCGGTGTGTCCTTGGGGCTGGCCGGCTGGGG
Rhl_F        GGCTCAGCAGATTGGCTGCTGCTGCTGTTGCGGTGTGTCCTTGGGGCTGGCCGGCTGGGG
Rhl_R        GGCTCAGCAGATTGGCTGCTGCTGCTGTTGCGGTGTGTCCTTGGGGCTGGCCGGCTGGGG
             ************************************************************

Karat_F      CATTCATTTGGGCGGCAAACGGGGCGTTAGTTAGGACAGGGAAACCGACAGCTGCATGCG
Karat_R      CATTCATTTGGGCGGCAAACGGGGCGTTAGTTAGGACAGGGAAACCGACAGCTGCATGCG
Rhl_F        CATTCATTTGGGCGGCAAACGGGGCGTTAGTTAGGACAGGGAAACCGACAGCTGCATGCG
Rhl_R        CATTCATTTGGGCGGCAAACGGGGCGTTAGTTAGGACAGGGAAACCGACAGCTGCATGCG
             ************************************************************

Karat_F      GCGCAGCAGCAGTAACAGACAGTGGTAAGAAATGGAAGGAGGAGAAGCTGCGCGCTCTGC
Karat_R      GCGCAGCAGCAGTAACAGACAGTGGTAAGAAATGGAAGGAGGAGAAGCTGCGCGCTCTGC
Rhl_F        GCGCAGCAGCAGTAACAGACAGTGGTAAGAAATGGAAGGAGGAGAAGCTGCGCGCTCTGC
Rhl_R        GCGCAGCAGCAGTAACAGACAGTGGTAAGAAATGGAAGGAGGAGAAGCTGCGCGCTCTGC
             ************************************************************

Karat_F      TCT
Karat_R      TCT
Rhl_F        TCT
Rhl_R        TCT
             ***
```
