# Supplementary material for: Forward Genetics Approach Reveals a Mutation in bHLH Transcription Factor-Encoding Gene as the Best Candidate for the Root Hairless Phenotype in Barley
Source: Front Plant Sci. 2018 Sep 3;9:1229. doi: 10.3389/fpls.2018.01229 (PMC6129617; doi:10.3389/fpls.2018.01229)
Supplement: FIGURE S3 — The alignments of genomic and protein sequences of HORVU7Hr1G030250 candidate gene between rhl1.b mutant and its parent variety ‘Karat’. [file Image_3.PDF]

gDNA_HORVU7Hr1G030250_Alignment

```
KARAT_F      TAATTGAATAAGGACAATAGAATAGTTTGATGGAATGAACTCATAAGTAGTGACACCCTA
KARAT_R      TAATTGAATAAGGACAATAGAATAGTTTGATGGAATGAACTCATAAGTAGTGACACCCTA
rhl_F        TAATTGAATAAGGACAATAGAATAGTTTGATGGAATGAACTCATAAGTAGTGACACCCTA
rhl_R        TAATTGAATAAGGACAATAGAATAGTTTGATGGAATGAACTCATAAGTAGTGACACCCTA
             ************************************************************

KARAT_F      CACAATGAGCTGGGTCAAACATTAACTCGTTGTGGGTGGCCTTCACGGACCTTTGAGGAT
KARAT_R      CACAATGAGCTGGGTCAAACATTAACTCGTTGTGGGTGGCCTTCACGGACCTTTGAGGAT
rhl_F        CACAATGAGCTGGGTCAAACATTAACTCGTTGTGGGTGGCCTTCACGGACCTTTGAGGAT
rhl_R        CACAATGAGCTGGGTCAAACATTAACTCGTTGTGGGTGGCCTTCACGGACCTTTGAGGAT
             ************************************************************

KARAT_F      ATACAGAAAGAAATTCTCAGGTTGCATGAAAGATGGATGAAGCAACACCATAAGAGTGAA
KARAT_R      ATACAGAAAGAAATTCTCAGGTTGCATGAAAGATGGATGAAGCAACACCATAAGAGTGAA
rhl_F        ATACAGAAAGAAATTCTCAGGTTGCATGAAAGATGGATGAAGCAACACCATAAGAGTGAA
rhl_R        ATACAGAAAGAAATTCTCAGGTTGCATGAAAGATGGATGAAGCAACACCATAAGAGTGAA
             ************************************************************

KARAT_F      TCATTCCTAAAGTATGCAACAGAACATAATTTTTTATGGAGTGCCGACAACGATGACGAA
KARAT_R      TCATTCCTAAAGTATGCAACAGAACATAATTTTTTATGGAGTGCCGACAACGATGACGAA
rhl_F        TCATTCCTAAAGTATGCAACAGAACATAATTTTTTATGGAGTGCCGACAACGATGACGAA
rhl_R        TCATTCCTAAAGTATGCAACAGAACATAATTTTTTATGGAGTGCCGACAACGATGACGAA
             ************************************************************

KARAT_F      GACGATATCTTCATGCCGAGCACCAAGGCCAAGACTACCACATCGTCGAAGGGCAAGAAT
KARAT_R      GACGATATCTTCATGCCGAGCACCAAGGCCAAGACTACCACATCGTCGAAGGGCAAGAAT
rhl_F        GACGATATCTTCATGCCGAGCACCAAGGCCAAGACTACCACATCGTCGAAGGGCAAGAAT
rhl_R        GACGATATCTTCATGCCGAGCACCAAGGCCAAGACTACCACATCGTCGAAGGGCAAGAAT
             ************************************************************

KARAT_F      GTTGCATCGACGGAGGATGCCGATGATGGCTTCATGTAGTTTTTAAGTTGTAGTTTTTAA
KARAT_R      GTTGCATCGACGGAGGATGCCGATGATGGCTTCATGTAGTTTTTAAGTTGTAGTTTTTAA
rhl_F        GTTGCATCGACGGAGGATGCCGATGATGGCTTCATGTAGTTTTTAAGTTGTAGTTTTTAA
rhl_R        GTTGCATCGACGGAGGATGCCGATGATGGCTTCATGTAGTTTTTAAGTTGTAGTTTTTAA
             ************************************************************

KARAT_F      GTTCAGTCCTACCGTTTAATTCAGTTGTACTTGTATCTTAATTATCTATTGTGGTTTCTT
KARAT_R      GTTCAGTCCTACCGTTTAATTCAGTTGTACTTGTATCTTAATTATCTATTGTGGTTTCTT
rhl_F        GTTCAGTCCTACCGTTTAATTCAGTTGTACTTGTATCTTAATTATCTATTGTGGTTTCTT
rhl_R        GTTCAGTCCTACCGTTTAATTCAGTTGTACTTGTATCTTAATTATCTATTGTGGTTTCTT
             ************************************************************

KARAT_F      TGAAAATGTGACTCGAATAATAATGAGGTTTCAATGGCCTCAACCACACAATACATTGGG
KARAT_R      TGAAAATGTGACTCGAATAATAATGAGGTTTCAATGGCCTCAACCACACAATACATTGGG
rhl_F        TGAAAATGTGACTCGAATAATAATGAGGTTTCAATGGCCTCAACCACACAATACATTGGG
rhl_R        TGAAAATGTGACTCGAATAATAATGAGGTTTCAATGGCCTCAACCACACAATACATTGGG
             ************************************************************

KARAT_F      AGATACAAGGATTAAAACTACCATCAAAATGAAAATCGGACATCTTCACGAGACTATACT
KARAT_R      AGATACAAGGATTAAAACTACCATCAAAATGAAAATCGGACATCTTCACGAGACTATACT
rhl_F        AGATACAAGGATTAAAACTACCATCAAAATGAAAATCGGACATCTTCACGAGACTATACT
rhl_R        AGATACAAGGATTAAAACTACCATCAAAATGAAAATCGGACATCTTCACGAGACTATACT
             ************************************************************

KARAT_F      GAACTACAATCAAAATGACGGAAAAGAATGGCATGAATAAGTGTACAGAAAAGGTAGCGG
KARAT_R      GAACTACAATCAAAATGACGGAAAAGAATGGCATGAATAAGTGTACAGAAAAGGTAGCGG
rhl_F        GAACTACAATCAAAATGACGGAAAAGAATGGCATGAATAAGTGTACAGAAAAGGTAGCGG
rhl_R        GAACTACAATCAAAATGACGGAAAAGAATGGCATGAATAAGTGTACAGAAAAGGTAGCGG
             ************************************************************

KARAT_F      GAAACTAGGAAGAGGATAGGGGAAAACTAGGGATAAAATTACGATGAAAAATATGTGGCA
KARAT_R      GAAACTAGGAAGAGGATAGGGGAAAACTAGGGATAAAATTACGATGAAAAATATGTGGCA
rhl_F        GAAACTAGGAAGAGGATAGGGGAAAACTAGGGATAAAATTACGATGAAAAATATGTGGCA
rhl_R        GAAACTAGGAAGAGGATAGGGGAAAACTAGGGATAAAATTACGATGAAAAATATGTGGCA
             ************************************************************
```

```
KARAT_F      TGCATTAATTGGTTTCGGCGAAGCTAATTAGTACTAATTAGCTTCATGGTATATGTTAGC
KARAT_R      TGCATTAATTGGTTTCGGCGAAGCTAATTAGTACTAATTAGCTTCATGGTATATGTTAGC
rhl_F        TGCATTAATTGGTTTCGGCGAAGCTAATTAGTACTAATTAGCTTCATGGTATATGTTAGC
rhl_R        TGCATTAATTGGTTTCGGCGAAGCTAATTAGTACTAATTAGCTTCATGGTATATGTTAGC
             ************************************************************

KARAT_F      TTCGCTGAAGCTGACTGGAATCTATCAGCTTCGTCGAAGCCGACTGGATTCTATTTTAAA
KARAT_R      TTCGCTGAAGCTGACTGGAATCTATCAGCTTCGTCGAAGCCGACTGGATTCTATTTTAAA
rhl_F        TTCGCTGAAGCTGACTGGAATCTATCAGCTTCGTCGAAGCCGACTGGATTCTATTTTAAA
rhl_R        TTCGCTGAAGCTGACTGGAATCTATCAGCTTCGTCGAAGCCGACTGGATTCTATTTTAAA
             ************************************************************

KARAT_F      AAACTTATAACTTGCGTATTACTTCCTCCATTTTAAAATTCTTGTCTTAAATTTATTTAG
KARAT_R      AAACTTATAACTTGCGTATTACTTCCTCCATTTTAAAATTCTTGTCTTAAATTTATTTAG
rhl_F        AAACTTATAACTTGCGTATTACTTCCTCCATTTTAAAATTCTTGTCTTAAATTTATTTAG
rhl_R        AAACTTATAACTTGCGTATTACTTCCTCCATTTTAAAATTCTTGTCTTAAATTTATTTAG
             ************************************************************

KARAT_F      ATGTGAATGTATCTAGTTAAATTTTAGTATTTTGATATATTTATTTCTAGACAAAGCTAA
KARAT_R      ATGTGAATGTATCTAGTTAAATTTTAGTATTTTGATATATTTATTTCTAGACAAAGCTAA
rhl_F        ATGTGAATGTATCTAGTTAAATTTTAGTATTTTGATATATTTATTTCTAGACAAAGCTAA
rhl_R        ATGTGAATGTATCTAGTTAAATTTTAGTATTTTGATATATTTATTTCTAGACAAAGCTAA
             ************************************************************

KARAT_F      GACAAGAATTTTAGGACGGAAGGAGTATTTATTAAAATCATAAAAATAAATGTATTATTT
KARAT_R      GACAAGAATTTTAGGACGGAAGGAGTATTTATTAAAATCATAAAAATAAATGTATTATTT
rhl_F        GACAAGAATTTTAGGACGGAAGGAGTATTTATTAAAATCATAAAAATAAATGTATTATTT
rhl_R        GACAAGAATTTTAGGACGGAAGGAGTATTTATTAAAATCATAAAAATAAATGTATTATTT
             ************************************************************

KARAT_F      TAAAAAATAGCAAAGAAATAGAGCACTGACAATCAAATTACTCAAATCCCATGTTTATAC
KARAT_R      TAAAAAATAGCAAAGAAATAGAGCACTGACAATCAAATTACTCAAATCCCATGTTTATAC
rhl_F        TAAAAAATAGCAAAGAAATAGAGCACTGACAATCAAATTACTCAAATCCCATGTTTATAC
rhl_R        TAAAAAATAGCAAAGAAATAGAGCACTGACAATCAAATTACTCAAATCCCATGTTTATAC
             ************************************************************

KARAT_F      CAGCTGTAATTTGCAGCTCTTAAGCAAAGTCAGGAAAAAAAAAGTAGACAGAAGGTCAGTA
KARAT_R      CAGCTGTAATTTGCAGCTCTTAAGCAAAGTCAGGAAAAAAAAAGTAGACAGAAGGTCAGTA
rhl_F        CAGCTGTAATTTGCAGCTCTTAAGCAAAGTCAGGAAAAAAAAAGTAGACAGAAGGTCAGTA
rhl_R        CAGCTGTAATTTGCAGCTCTTAAGCAAAGTCAGGAAAAAAAAAGTAGACAGAAGGTCAGTA
             ************************************************************

KARAT_F      TATAAACGCCAAAGAGTTGCACGAACCGAACTCAGTTAGTTTTCCATTTGAAAATTGGCA
KARAT_R      TATAAACGCCAAAGAGTTGCACGAACCGAACTCAGTTAGTTTTCCATTTGAAAATTGGCA
rhl_F        TATAAACGCCAAAGAGTTGCACGAACCGAACTCAGTTAGTTTTCCATTTGAAAATTGGCA
rhl_R        TATAAACGCCAAAGAGTTGCACGAACCGAACTCAGTTAGTTTTCCATTTGAAAATTGGCA
             ************************************************************

KARAT_F      CCTTAGCACGGGATTATAAAGGCGCGCACGTAGCGAAGCTAGTTCGCCTCGCCTCCTCCA
KARAT_R      CCTTAGCACGGGATTATAAAGGCGCGCACGTAGCGAAGCTAGTTCGCCTCGCCTCCTCCA
rhl_F        CCTTAGCACGGGATTATAAAGGCGCGCACGTAGCGAAGCTAGTTCGCCTCGCCTCCTCCA
rhl_R        CCTTAGCACGGGATTATAAAGGCGCGCACGTAGCGAAGCTAGTTCGCCTCGCCTCCTCCA
             ************************************************************

KARAT_F      TACTCTCCTCCTGCCTTCTCGCGACCGCATTTACCCGCCAGCTCGTAGCTGCACAGCCCG
KARAT_R      TACTCTCCTCCTGCCTTCTCGCGACCGCATTTACCCGCCAGCTCGTAGCTGCACAGCCCG
rhl_F        TACTCTCCTCCTGCCTTCTCGCGACCGCATTTACCCGCCAGCTCGTAGCTGCACAGCCCG
rhl_R        TACTCTCCTCCTGCCTTCTCGCGACCGCATTTACCCGCCAGCTCGTAGCTGCACAGCCCG
             ************************************************************

KARAT_F      GGGACGCAGCCCGCGCGAGAGACCGATGGCAGGCGGTGACGGCGGCGGGGGCGGCGGCGC
KARAT_R      GGGACGCAGCCCGCGCGAGAGACCGATGGCAGGCGGTGACGGCGGCGGGGGCGGCGGCGC
rhl_F        GGGACGCAGCCCGCGCGAGAGACCGATGGCAGGCGGTGACGGCGGCGGGGGCGGCGGCGC
rhl_R        GGGACGCAGCCCGCGCGAGAGACCGATGGCAGGCGGTGACGGCGGCGGGGGCGGCGGCGC
             ************************************************************
```

```
KARAT_F      GCAGGACGACTTCTTCGACCAGATGCTGTCGACGCTGCCCTCCGCCTGGGGCGACCTTGG
KARAT_R      GCAGGACGACTTCTTCGACCAGATGCTGTCGACGCTGCCCTCCGCCTGGGGCGACCTTGG
rhl_F        GCAGGACGACTTCTTCGACCAGATGCTGTCGACGCTGCCCTCCGCCTGGGGCGACCTTGG
rhl_R        GCAGGACGACTTCTTCGACCAGATGCTGTCGACGCTGCCCTCCGCCTGGGGCGACCTTGG
             ************************************************************

KARAT_F      CGCCGGCGGGAAGTCGCCCTGGGAGATCGCGGCCGGCGCCGAGGACCTCGGAGCCTTCGA
KARAT_R      CGCCGGCGGGAAGTCGCCCTGGGAGATCGCGGCCGGCGCCGAGGACCTCGGAGCCTTCGA
rhl_F        CGCCGGCGGGAAGTCGCCCTGGGAGATCGCGGCCGGCGCCGAGGACCTCGGAGCCTTCGA
rhl_R        CGCCGGCGGGAAGTCGCCCTGGGAGATCGCGGCCGGCGCCGAGGACCTCGGAGCCTTCGA
             ************************************************************

KARAT_F      CGAGTCGGCGCTGCTCGCGTCCAGGCTCCGGCAGCACCAGATCGGCGGAGAGAAGCCGGT
KARAT_R      CGAGTCGGCGCTGCTCGCGTCCAGGCTCCGGCAGCACCAGATCGGCGGAGAGAAGCCGGT
rhl_F        CGAGTCGGCGCTGCTCGCGTCCAGGCTCCGGCAGCACCAGATCGGCGGAGAGAAGCCGGT
rhl_R        CGAGTCGGCGCTGCTCGCGTCCAGGCTCCGGCAGCACCAGATCGGCGGAGAGAAGCCGGT
             ************************************************************

KARAT_F      GATGCTGCAGCTCACCGACCTCCAGCGGCAGGGCCTCGGCGAGGAGACCGGCGGCACGGG
KARAT_R      GATGCTGCAGCTCACCGACCTCCAGCGGCAGGGCCTCGGCGAGGAGACCGGCGGCACGGG
rhl_F        GATGCTGCAGCTCACCGACCTCCAGCGGCAGGGCCTCGGCGAGGAGACCGGCGGCACGGG
rhl_R        GATGCTGCAGCTCACCGACCTCCAGCGGCAGGGCCTCGGCGAGGAGACCGGCGGCACGGG
             ************************************************************

KARAT_F      GTTCTCGCCGCTGCCGCTGTTCGCGGACCGGTCGCCCCCGCAGTCGCGGGAGGAGATGGA
KARAT_R      GTTCTCGCCGCTGCCGCTGTTCGCGGACCGGTCGCCCCCGCAGTCGCGGGAGGAGATGGA
rhl_F        GTTCTCGCCGCTGCCGCTGTTCGCGGACCGGTCGCCCCCGCAGTCGCGGGAGGAGATGGA
rhl_R        GTTCTCGCCGCTGCCGCTGTTCGCGGACCGGTCGCCCCCGCAGTCGCGGGAGGAGATGGA
             ************************************************************

KARAT_F      CGGCGGCTTCAAGTCGCCCAATGGCACGGTATAGGATCCTTTTTCATCCTCGTTCTCTCT
KARAT_R      CGGCGGCTTCAAGTCGCCCAATGGCACGGTATAGGATCCTTTTTCATCCTCGTTCTCTCT
rhl_F        CGGCGGCTTCAAGTCGCCCAATGGCACGGTATAGGATCCTTTTTCATCCTCGTTCTCTCT
rhl_R        CGGCGGCTTCAAGTCGCCCAATGGCACGGTATAGGATCCTTTTTCATCCTCGTTCTCTCT
             ************************************************************

KARAT_F      CTGCCGTTCTTAATTTCTCTAGTCAAAATTTCCCTTGACGACATGGACTCGTTTTGTGCG
KARAT_R      CTGCCGTTCTTAATTTCTCTAGTCAAAATTTCCCTTGACGACATGGACTCGTTTTGTGCG
rhl_F        CTGCCGTTCTTAATTTCTCTAGTCAAAATTTCCCTTGACGACATGGACTCGTTTTGTGCG
rhl_R        CTGCCGTTCTTAATTTCTCTAGTCAAAATTTCCCTTGACGACATGGACTCGTTTTGTGCG
             ************************************************************

KARAT_F      GGCGCGTGCGTGCATGCATGCAGGGAGGTGACCACGCGCTGTTCAACGGATTTGGGGTGC
KARAT_R      GGCGCGTGCGTGCATGCATGCAGGGAGGTGACCACGCGCTGTTCAACGGATTTGGGGTGC
rhl_F        GGCGCGTGCGTGCATGCATGCAGGGAGGTGACCACGCGCTGTTCAACGGATTTGGGGTGC
rhl_R        GGCGCGTGCGTGCATGCATGCAGGGAGGTGACCACGCGCTGTTCAACGGATTTGGGGTGC
             ************************************************************

KARAT_F      ATGGCGGCGCCGCCGCGGTGCAGCCGACGTTCGGCCAGGTACAAACTTGCATGCACCTGT
KARAT_R      ATGGCGGCGCCGCCGCGGTGCAGCCGACGTTCGGCCAGGTACGAACTTGCATGCACCTGT
rhl_F        ATGGCGGCGCCGCCGCGGTGCAGCCGACGTTCGGCCAGGTACGAACTTGCATGCACCTGT
rhl_R        ATGGCGGCGCCGCCGCGGTGCAGCCGACGTTCGGCCAGGTACGAACTTGCATGCACCTGT
             ************************************************************

KARAT_F      ACTACGATCCACGCACGACCGTGTGAAGCAGCACCATATAATCGCATGTTCTTTGATCGC
KARAT_R      ACTACGATCCACGCACGACCGTGTGAAGCAGCACCATATAATCGCATGTTCTTTGATCGC
rhl_F        ACTACGATCCACGCACGACCGTGTGAAGCAGCACCATATAATCGCATGTTCTTTGATCGC
rhl_R        ACTACGATCCACGCACGACCGTGTGAAGCAGCACCATATAATCGCATGTTCTTTGATCGC
             ************************************************************

KARAT_F      TGGCAGGGAGGATCAATGTCGGGGCAGAGCTTCGGAGGAGGGCCGGCGGCGAGCGGAGGC
KARAT_R      TGGCAGGGAGGATCAATGTCGGGGCAGAGCTTCGGAGGAGGGCCGGCGGCGAGCGGAGGC
rhl_F        TGGCAGGGAGGATCAATGTCGGGGCAGAGCTTCGGAGGAGGGCCGGCGGCGAGCGGAGGC
rhl_R        TGGCAGGGAGGATCAATGTCGGGGCAGAGCTTCGGAGGAGGGCCGGCGGCGAGCGGAGGC
             ************************************************************

KARAT_F      ACGACAGCGCCCGCCTCCTCTGGCGGCGGCGGCGCGGCCCCGCCGCGGCAGACGCGCGTG
```

```
KARAT_R      ACGACAGCGCCCGCCTCCTCTGGCGGCGGCGGCGCGGCCCCGCCGCGGCAGACGCGCGTG
rhl_F        ACGACAGCGCCCGCCTCCTCTGGCGGCGGCGGCGCGGCCCCGCCGCGGCAGACGCGCGTG
rhl_R        ACGACAGCGCCCGCCTCCTCTGGCGGCGGCGGCGCGGCCCCGCCGCGGCAGACGCGCGTG
             ************************************************************

KARAT_F      CGGGCGAGGCGAGGGCAGGCCACCGACCCGCACAGCATCGCCGAACGTGTACGCATGCCA
KARAT_R      CGGGCGAGGCGAGGGCAGGCCACCGACCCGCACAGCATCGCCGAACGTGTACGCATGCCA
rhl_F        CGGGCGAGGCGAGGGCAGGCCACCGACCCGCACAGCATCGCCGAACGTGTACGCATGCCA
rhl_R        CGGGCGAGGCGAGGGCAGGCCACCGACCCGCACAGCATCGCCGAACGTGTACGCATGCCA
             ************************************************************

KARAT_F      CTCCCACACTCTCACCATATTCCCCCTTCCTCTGACGCAATTTTAAAAACAAAACCCCAA
KARAT_R      CTCCCACACTCTCACCATATTCCCCCTTCCTCTGACGCAATTTTAAAAACAAAACCCCAA
rhl_F        CTCCCACACTCTCACCATATTCCCCCTTCCTCTGACGCAATTTTAAAAACAAAACCCCAA
rhl_R        CTCCCACACTCTCACCATATTCCCCCTTCCTCTGACGCAATTTTAAAAACAAAACCCCAA
             ************************************************************

KARAT_F      CATGATGGTGCACATCAACAACAACCCAAAGTCTCCCGTCCTCCAACGTATATATTCTCT
KARAT_R      CATGATGGTGCACATCAACAACAACCCAAAGTCTCCCGTCCTCCAACGTATATATTCTCT
rhl_F        CATGATGGTGCACATCAACAACAACCCAAAGTCTCCCGTCCTCCAACGTATATATTCTCT
rhl_R        CATGATGGTGCACATCAACAACAACCCAAAGTCTCCCGTCCTCCAACGTATATATTCTCT
             ************************************************************

KARAT_F      GCAAATTTGATCGCTCCTCTTTTTTATAACAGAAGATGCAGGAACCGTTCTGTTCCTACG
KARAT_R      GCAAATTTGATCGCTCCTCTTTTTTATAACAGAAGATGCAGGAACCGTTCTGTTCCTACG
rhl_F        GCAAATTTGATCGCTCCTCTTTTTTATAACAGAAGATGCAGGAACCGTTCTGTTCCTACG
rhl_R        GCAAATTTGATCGCTCCTCTTTTTTATAACAGAAGATGCAGGAACCGTTCTGTTCCTACG
             ************************************************************

KARAT_F      CTACGCTATGCTATGCTATGATATGCTATGCTTGTAATCAAACTGAAATTTGAACGACTT
KARAT_R      CTACGCTATGCTATGCTATGATATGCTATGCTTGTAATCAAACTGAAATTTGAACGACTT
rhl_F        CTACGCTATGCTATGCTATGATATGCTATGCTTGTAATCAAACTGAAATTTGAACGACTT
rhl_R        CTACGCTATGCTATGCTATGATATGCTATGCTTGTAATCAAACTGAAATTTGAACGACTT
             ************************************************************

KARAT_F      TTTACCCTGAGCAACTCGCGTGCATGCAGCTCCGGCGGGAGAGGATCGCGGAGCGGATGA
KARAT_R      TTTACCCTGAGCAACTCGCGTGCATGCAGCTCCGGCGGGAGAGGATCGCGGAGCGGATGA
rhl_F        TTTACCCTGAGCAACTCGCGTGCATGCAGCTCCGGCGGGAGAGGATCGCGGAGCGGATGA
rhl_R        TTTACCCTGAGCAACTCGCGTGCATGCAGCTCCGGCGGGAGAGGATCGCGGAGCGGATGA
             ************************************************************

KARAT_F      AGTCGCTGCAGGAGCTGGTCCCCAACGCCAACAAGGTACGTAAAACAATCACGTCCCCGA
KARAT_R      AGTCGCTGCAGGAGCTGGTCCCCAACGCCAACAAGGTACGTAAAACAATCACGTCCCCGA
rhl_F        AGTCGCTGCAGGAGCTGGTCCCCAACGCCAACAAGGTACGTAAAACAATCACGTCCCCGA
rhl_R        AGTCGCTGCAGGAGCTGGTCCCCAACGCCAACAAGGTACGTAAAACAATCACGTCCCCGA
             ************************************************************

KARAT_F      CGAGAATCTCTCGGCGATCAAACCGTATCGTGTAGAAAATTCCTGCGTTTCCCATCGACC
KARAT_R      CGAGAATCTCTCGGCGATCAAACCGTATCGTGTAGAAAATTCCTGCGTTTCCCATCGACC
rhl_F        CGAGAATCTCTCGGCGATCAAACCGTATCGTGTAGAAAATTCCTGCGTTTCCCATCGACC
rhl_R        CGAGAATCTCTCGGCGATCAAACCGTATCGTGTAGAAAATTCCTGCGTTTCCCATCGACC
             ************************************************************

KARAT_F      TGAAACTGAAAGCCAGGAATCTCTCCCTGAAAGCCAGGAATCTCTCCCGGCGCGTGGGGG
KARAT_R      TGAAACTGAAAGCCAGGAATCTCTCCCTGAAAGCCAGGAATCTCTCCCGGCGCGTGGGGG
rhl_F        TGAAACTGAAAGCCAGGAATCTCTCCCTGAAAGCCAGGAATCTCTCCCGGCGCGTGGGGG
rhl_R        TGAAACTGAAAGCCAGGAATCTCTCCCTGAAAGCCAGGAATCTCTCCCGGCGCGTGGGGG
             ************************************************************

KARAT_F      TGGGGGCTCGCCTGTCTATGCTACTAGTGGGCTTGTATGGTAGCAGTGCAGAAAAGGGCA
KARAT_R      TGGGGGCTCGCCTGTCTATGCTACTAGTGGGCTTGTATGGTAGCAGTGCAGAAAAGGGCA
rhl_F        TGGGGGCTCGCCTGTCTATGCTACTAGTGGGCTTGTATGGTAGCAGTGCAGAAAAGGGCA
rhl_R        TGGGGGCTCGCCTGTCTATGCTACTAGTGGGCTTGTATGGTAGCAGTGCAGAAAAGGGCA
             ************************************************************

KARAT_F      CGCCGGCCGGCCGGTTGGTGAGCGAGGGTGCGGCGTCGCTCCTGTCTAGCTCACGGGTCA
KARAT_R      CGCCGGCCGGCCGGTTGGTGAGCGAGGGTGCGGCGTCGCTCCTGTCTAGCTCACGGGTCA
```

```
rhl_F        CGCCGGCCGGCCGGTTGGTGAGCGAGGGTGCGGCGTCGCTCCTGTCTAGCTCACGGGTCA
rhl_R        CGCCGGCCGGCCGGTTGGTGAGCGAGGGTGCGGCGTCGCTCCTGTCTAGCTCACGGGTCA
             ************************************************************

KARAT_F      CGCCATGATGTGATGTGATGTGACATGTGTGCCGTCGAAGCGTGCGCAGCACAGGAGACG
KARAT_R      CGCCATGATGTGATGTGATGTGACATGTGTGCCGTCGAAGCGTGCGCAGCACAGGAGACG
rhl_F        CGCCATGATGTGATGTGATGTGACATGTGTGCCGTCGAAGCGTGCGCAGCACAGGAGACG
rhl_R        CGCCATGATGTGATGTGATGTGACATGTGTGCCGTCGAAGCGTGCGCAGCACAGGAGACG
             ************************************************************

KARAT_F      CAGCACGTGTCGAGCTCGTGCTGGAAATGTCTTGGACCGGAGAAGGCCGCAGCCTTGGGT
KARAT_R      CAGCACGTGTCGAGCTCGTGCTGGAAATGTCTTGGACCGGAGAAGGCCGCAGCCTTGGGT
rhl_F        CAGCACGTGTCGAGCTCGTGCTGGAAATGTCTTGGACCGGAGAAGGCCGCAGCCTTGGGT
rhl_R        CAGCACGTGTCGAGCTCGTGCTGGAAATGTCTTGGACCGGAGAAGGCCGCAGCCTTGGGT
             ************************************************************

KARAT_F      GGCTCTTGGGCTAGCTGAAGAATTCAGCTGATGCCGGGATGGCAAATGAGGCGTGCAGTG
KARAT_R      GGCTCTTGGGCTAGCTGAAGAATTCAGCTGATGCCGGGATGGCAAATGAGGCGTGCAGTG
rhl_F        GGCTCTTGGGCTAGCTGAAGAATTCAGCTGATGCCGGGATGGCAAATGAGGCGTGCAGTG
rhl_R        GGCTCTTGGGCTAGCTGAAGAATTCAGCTGATGCCGGGATGGCAAATGAGGCGTGCAGTG
             ************************************************************

KARAT_F      AATGGGCACATGGGCGCATTGGATGCCCTGTTTCGCTCGATCCGCCATGCCCTTCTAGTC
KARAT_R      AATGGGCACATGGGCGCATTGGATGCCCTGTTTCGCTCGATCCGCCATGCCCTTCTAGTC
rhl_F        AATGGGCACATGGGCGCATTGGATGCCCTGTTTCGCTCGATCCGCCATGCCCTTCTAGTC
rhl_R        AATGGGCACATGGGCGCATTGGATGCCCTGTTTCGCTCGATCCGCCATGCCCTTCTAGTC
             ************************************************************

KARAT_F      CGGTAGTCATAATTACAGGCCATTTGTCAATGCCCGGATATGTGATCGCGCTGAAATCGA
KARAT_R      CGGTAGTCATAATTACAGGCCATTTGTCAATGCCCGGATATGTGATCGCGCTGAAATCGA
rhl_F        CGGTAGTCATAATTACAGGCCATTTGTCAATGCCCGGATATGTGATCGCGCTGAAATCGA
rhl_R        CGGTAGTCATAATTACAGGCCATTTGTCAATGCCCGGATATGTGATCGCGCTGAAATCGA
             ************************************************************

KARAT_F      GCCGTTTCGTGTAGTATGTAAAAATCTATGCGCCGTGGTGGTAGAATACTCGTTAGAAGG
KARAT_R      GCCGTTTCGTGTAGTATGTAAAAATCTATGCGCCGTGGTGGTAGAATACTCGTTAGAAGG
rhl_F        GCCGTTTCGTGTAGTATGTAAAAATCTATGCGCCGTGGTGGTAGAATACTCGTTAGAAGG
rhl_R        GCCGTTTCGTGTAGTATGTAAAAATCTATGCGCCGTGGTGGTAGAATACTCGTTAGAAGG
             ************************************************************

KARAT_F      GGCGGTTACTGATGAGATGGCCATGCCGTTGGATTGTTGATTCGCAGACTGACAAGGCGT
KARAT_R      GGCGGTTACTGATGAGATGGCCATGCCGTTGGATTGTTGATTCGCAGACTGACAAGGCGT
rhl_F        GGCGGTTACTGATGAGATGGCCATGCCGTTGGATTGTTGATTCGCAGACTGACAAGGCGT
rhl_R        GGCGGTTACTGATGAGATGGCCATGCCGTTGGATTGTTGATTCGCAGACTGACAAGGCGT
             ************************************************************

KARAT_F      CGATGCTGGACGAGATCATCGACTACGTCAAGTTCCTGCAGCTCCAAGTCAAGGTATATA
KARAT_R      CGATGCTGGACGAGATCATCGACTACGTCAAGTTCCTGCAGCTCCAAGTCAAGGTATATA
rhl_F        CGATGCTGGACGAGATCATCGACTACGTCAAGTTCCTGCAGCTCCAAGTCAAGGTATATA
rhl_R        CGATGCTGGACGAGATCATCGACTACGTCAAGTTCCTGCAGCTCCAAGTCAAGGTATATA
             ************************************************************

KARAT_F      TACAGAGGTGTTCGATGACAGAGGTTGCTCTGTGTTTCCCTGCTTCTGATTACGTAATAC
KARAT_R      TACAGAGGTGTTCGATGACAGAGGTTGCTCTGTGTTTCCCTGCTTCTGATTACGTAATAC
rhl_F        TACAGAGGTGTTCGATGACAGAGGTTGCTCTGTGTTTCCCTGCTTCTGATTACGTAATAC
rhl_R        TACAGAGGTGTTCGATGACAGAGGTTGCTCTGTGTTTCCCTGCTTCTGATTACGTAATAC
             ************************************************************

KARAT_F      GACCTGTGGAGGTGACACCACGCTTTTGTTCTGTCAGGTTCTGAGCATGAGCCGGCTGGG
KARAT_R      GACCTGTGGAGGTGACACCACGCTTTTGTTCTGTCAGGTTCTGAGCATGAGCCGGCTGGG
rhl_F        GACCTGTGGAGGTGACACCACGCTTTTGTTCTGTCAGGTTCTGAGCATGAGCCGGCTGGG
rhl_R        GACCTGTGGAGGTGACACCACGCTTTTGTTCTGTCAGGTTCTGAGCATGAGCCGGCTGGG
             ************************************************************

KARAT_F      CGGGGCGGCCGGTATGGCGCCGCTGGTGGCCAGCATGTCCTCCGAGGTAGGGCTACTCAA
KARAT_R      CGGGGCGGCCGGTATGGCGCCGCTGGTGGCCAGCATGTCCTCCGAGGTAGGGCTACTCAA
rhl_F        CGGGGCGGCCGGTATGGCGCCGCTGGTGGCCAGCATGTCCTCCGAGGTAGGGCTACTCAA
```

```
rhl_R          CGGGGCGGCCGGTATGGCGCCGCTGGTGGCCAGCATGTCCTCCGAGGTAGGGCTACTCAA
               ************************************************************

KARAT_F        AGCCAGCACCCGTTCGAACCTACGTAATAACTTAAGTTGCCGTTCACTGTGTCCTGAGGC
KARAT_R        AGCCAGCACCCGTTCGAACCTACGTAATAACTTAAGTTGCCGTTCACTGTGTCCTGAGGC
rhl_F          AGCCAGCACCCGTTCGAACCTACGTAATAACTTAAGTTGCCGTTCACTGTGTCCTGAGGC
rhl_R          AGCCAGCACCCGTTCGAACCTACGTAATAACTTAAGTTGCCGTTCACTGTGTCCTGAGGC
               ************************************************************

KARAT_F        GCGTCGACGTGGAATGAAATGCAGGCGAACAGCAGCGCGAAGAGCAGCAACGGCGGCGGG
KARAT_R        GCGTCGACGTGGAATGAAATGCAGGCGAACAGCAGCGCGAAGAGCAGCAACGGCGGCGGG
rhl_F          GCGTCGACGTGGAATGAAATGCTGGCGAACAGCAGCGCGAAGAGCAGCAACGGCGGCGGG
rhl_R          GCGTCGACGTGGAATGAAATGCTGGCGAACAGCAGCGCGAAGAGCAGCAACGGCGGCGGG
               ********************** *************************************

KARAT_F        AACAGCGCGGCGGCCGCGGCGGCCAAGGCGAACGGCGGAGGCGAGAGCGGGGGCGGTGGA
KARAT_R        AACAGCGCGGCGGCCGCGGCGGCCAAGGCGAACGGCGGAGGCGAGAGCGGGGGCGGTGGA
rhl_F          AACAGCGCGGCGGCCGCGGCGGCCAAGGCGAACGGCGGAGGCGAGAGCGGGGGCGGTGGA
rhl_R          AACAGCGCGGCGGCCGCGGCGGCCAAGGCGAACGGCGGAGGCGAGAGCGGGGGCGGTGGA
               ************************************************************

KARAT_F        GGGGGCGGCGGGCTGCGGGTGACGGAGCAGCAGGTGGCCAAGATGATGGAGGAGGACATG
KARAT_R        GGGGGCGGCGGGCTGCGGGTGACGGAGCAGCAGGTGGCCAAGATGATGGAGGAGGACATG
rhl_F          GGGGGCGGCGGGCTGCGGGTGACGGAGCAGCAGGTGGCCAAGATGATGGAGGAGGACATG
rhl_R          GGGGGCGGCGGGCTGCGGGTGACGGAGCAGCAGGTGGCCAAGATGATGGAGGAGGACATG
               ************************************************************

KARAT_F        GGCACGGCCATGCAGTACCTGCAGGGGAAGGGCCTCTGCCTCATGCCCATCTCCCTCGCC
KARAT_R        GGCACGGCCATGCAGTACCTGCAGGGGAAGGGCCTCTGCCTCATGCCCATCTCCCTCGCC
rhl_F          GGCACGGCCATGCAGTACCTGCAGGGGAAGGGCCTCTGCCTCATGCCCATCTCCCTCGCC
rhl_R          GGCACGGCCATGCAGTACCTGCAGGGGAAGGGCCTCTGCCTCATGCCCATCTCCCTCGCC
               ************************************************************

KARAT_F        TCCGCCATCTCCTCCGCCACCACCACCACCTCCCCGGCCTCGCTCCTCGCGAGGCAGGCC
KARAT_R        TCCGCCATCTCCTCCGCCACCACCACCACCTCCCCGGCCTCGCTCCTCGCGAGGCAGGCC
rhl_F          TCCGCCATCTCCTCCGCCACCACCACCACCTCCCCGGCCTCGCTCCTCGCGAGGCAGGCC
rhl_R          TCCGCCATCTCCTCCGCCACCACCACCACCTCCCCGGCCTCGCTCCTCGCGAGGCAGGCC
               ************************************************************

KARAT_F        GTCCGCCCCGCCTCCACCGCCGCCGCGCTGGCCTCCGCGAACGGCGGCGAAGACGCCGCT
KARAT_R        GTCCGCCCCGCCTCCACCGCCGCCGCGCTGGCCTCCGCGAACGGCGGCGAAGACGCCGCT
rhl_F          GTCCGCCCCGCCTCCACCGCCGCCGCGCTGGCCTCCGCGAACGGCGGCGAAGACGCCGCT
rhl_R          GTCCGCCCCGCCTCCACCGCCGCCGCGCTGGCCTCCGCGAACGGCGGCGAAGACGCCGCT
               ************************************************************

KARAT_F        GCCAGGCCCGTCAAGGTCGATGCCGGCGCCGCGTCCGGCGGGAAGCCGTGAAGCTGACCG
KARAT_R        GCCAGGCCCGTCAAGGTCGATGCCGGCGCCGCGTCCGGCGGGAAGCCGTGAAGCTGACCG
rhl_F          GCCAGGCCCGTCAAGGTCGATGCCGGCGCCGCGTCCGGCGGGAAGCCGTGAAGCTGACCG
rhl_R          GCCAGGCCCGTCAAGGTCGATGCCGGCGCCGCGTCCGGCGGGAAGCCGTGAAGCTGACCG
               ************************************************************

KARAT_F        GCCGGAACAAGGGTGCATGCATTATCTATGCTAATACTATTAGGAGAGCCATATGTATTT
KARAT_R        GCCGGAACAAGGGTGCATGCATTATCTATGCTAATACTATTAGGAGAGCCATATGTATTT
rhl_F          GCCGGAACAAGGGTGCATGCATTATCTATGCTAATACTATTAGGAGAGCCATATGTATTT
rhl_R          GCCGGAACAAGGGTGCATGCATTATCTATGCTAATACTATTAGGAGAGCCATATGTATTT
               ************************************************************

KARAT_F        CGCTCCTGTATACGCCTTCCCTTCCAGTTTTCTTCCTCTGAAAATCAGACTCGCCATGCA
KARAT_R        CGCTCCTGTATACGCCTTCCCTTCCAGTTTTCTTCCTCTGAAAATCAGACTCGCCATGCA
rhl_F          CGCTCCTGTATACGCCTTCCCTTCCAGTTTTCTTCCTCTGAAAATCAGACTCGCCATGCA
rhl_R          CGCTCCTGTATACGCCTTCCCTTCCAGTTTTCTTCCTCTGAAAATCAGACTCGCCATGCA
               ************************************************************

KARAT_F        CACGTACGTGCATGCGCATGATCGAGCGAGCTCCCATGAGAAGGCTTACCACGACTTCCT
KARAT_R        CACGTACGTGCATGCGCATGATCGAGCGAGCTCCCATGAGAAGGCTTACCACGACTTCCT
rhl_F          CACGTACGTGCATGCGCATGATCGAGCGAGCTCCCATGAGAAGGCTTACCACGACTTCCT
rhl_R          CACGTACGTGCATGCGCATGATCGAGCGAGCTCCCATGAGAAGGCTTACCACGACTTCCT
```

```
           ************************************************************

KARAT_F    CCATGAATTTGCATGAAGATATTCTCGTGTGCATGCGCAGCCACCTTTCGTGCGCTTCGA
KARAT_R    CCATGAATTTGCATGAAGATATTCTCGTGTGCATGCGCAGCCACCTTTCGTGCGCTTCGA
rhl_F      CCATGAATTTGCATGAAGATATTCTCGTGTGCATGCGCAGCCACCTTTCGTGCGCTTCGA
rhl_R      CCATGAATTTGCATGAAGATATTCTCGTGTGCATGCGCAGCCACCTTTCGTGCGCTTCGA
           ************************************************************

KARAT_F    CGTTGACAAAACACGGTGGTACTAATCTCACAAGGTCAGCCGTTGACACTGCTGCCTCGG
KARAT_R    CGTTGACAAAACACGGTGGTACTAATCTCACAAGGTCAGCCGTTGACACTGCTGCCTCGG
rhl_F      CGTTGACAAAACACGGTGGTACTAATCTCACAAGGTCAGCCGTTGACACTGCTGCCTCGG
rhl_R      CGTTGACAAAACACGGTGGTACTAATCTCACAAGGTCAGCCGTTGACACTGCTGCCTCGG
           ************************************************************

KARAT_F    CATATTCCTAATCGGATGCGAGCATTAATGATCCTGATGCTGCTGGTGATTAGTTGCGAG
KARAT_R    CATATTCCTAATCGGATGCGAGCATTAATGATCCTGATGCTGCTGGTGATTAGTTGCGAG
rhl_F      CATATTCCTAATCGGATGCGAGCATTAATGATCCTGATGCTGCTGGTGATTAGTTGCGAG
rhl_R      CATATTCCTAATCGGATGCGAGCATTAATGATCCTGATGCTGCTGGTGATTAGTTGCGAG
           ************************************************************

KARAT_F    TAAAATGGTGGCGAGATGTGCAGGCTACTAACGCCGCACGACGTTCGTCTATGGGCTGGC
KARAT_R    TAAAATGGTGGCGAGATGTGCAGGCTACTAACGCCGCACGACGTTCGTCTATGGGCTGGC
rhl_F      TAAAATGGTGGCGAGATGTGCAGGCTACTAACGCCGCACGACGTTCGTCTATGGGCTGGC
rhl_R      TAAAATGGTGGCGAGATGTGCAGGCTACTAACGCCGCACGACGTTCGTCTATGGGCTGGC
           ************************************************************

KARAT_F    TGAGGTAAACAAATGCGGCGGAATAATTAAGGGCGTGCATTGTTCGCACGACACGGAGCG
KARAT_R    TGAGGTAAACAAATGCGGCGGAATAATTAAGGGCGTGCATTGTTCGCACGACACGGAGCG
rhl_F      TGAGGTAAACAAATGCGGCGGAATAATTAAGGGCGTGCATTGTTCGCACGACACGGAGCG
rhl_R      TGAGGTAAACAAATGCGGCGGAATAATTAAGGGCGTGCATTGTTCGCACGACACGGAGCG
           ************************************************************

KARAT_F    AGACGAGGATTAAGGGGGTGGATTGAGAATCAATCCGGCGCGAGGTGCCCACGTTGGTGG
KARAT_R    AGACGAGGATTAAGGGGGTGGATTGAGAATCAATCCGGCGCGAGGTGCCCACGTTGGTGG
rhl_F      AGACGAGGATTAAGGGGGTGGATTGAGAATCAATCCGGCGCGAGGTGCCCACGTTGGTGG
rhl_R      AGACGAGGATTAAGGGGGTGGATTGAGAATCAATCCGGCGCGAGGTGCCCACGTTGGTGG
           ************************************************************

KARAT_F    TGAGGCGAACGAGTTCCTGCCAGCTGCGGTAGAGGATTTTTGGATCCAGTATACCACCAC
KARAT_R    TGAGGCGAACGAGTTCCTGCCAGCTGCGGTAGAGGATTTTTGGATCCAGTATACCACCAC
rhl_F      TGAGGCGAACGAGTTCCTGCCAGCTGCGGTAGAGGATTTTTGGATCCAGTATACCACCAC
rhl_R      TGAGGCGAACGAGTTCCTGCCAGCTGCGGTAGAGGATTTTTGGATCCAGTATACCACCAC
           ************************************************************

KARAT_F    CATCATCATCACCATCACCACCACCACCACCACCACCACCATCACCACCACCACAACAAC
KARAT_R    CATCATCATCACCATCACCACCACCACCACCACCACCACCATCACCACCACCACAACAAC
rhl_F      CATCATCATCACCATCACCACCACCACCACCACCACCACCATCACCACCACCACAACAAC
rhl_R      CATCATCATCACCATCACCACCACCACCACCACCACCACCATCACCACCACCACAACAAC
           ************************************************************

KARAT_F    AACAATAAAAGACCCATCCCAGGTTGTCAGTGTGACGGGAGGACGACCTAGGTCTTCAAT
KARAT_R    AACAATAAAAGACCCATCCCAGGTTGTCAGTGTGACGGGAGGACGACCTAGGTCTTCAAT
rhl_F      AACAATAAAAGACCCATCCCAGGTTGTCAGTGTGACGGGAGGACGACCTAGGTCTTCAAT
rhl_R      AACAATAAAAGACCCATCCCAGGTTGTCAGTGTGACGGGAGGACGACCTAGGTCTTCAAT
           ************************************************************

KARAT_F    GCCAGAGCCTCCCTTTTCTTTCCGCTTCGCCGACGCCTGATGAGGAAGCTCAATCGGCGT
KARAT_R    GCCAGAGCCTCCCTTTTCTTTCCGCTTCGCCGACGCCTGATGAGGAAGCTCAATCGGCGT
rhl_F      GCCAGAGCCTCCCTTTTCTTTCCGCTTCGCCGACGCCTGATGAGGAAGCTCAATCGGCGT
rhl_R      GCCAGAGCCTCCCTTTTCTTTCCGCTTCGCCGACGCCTGATGAGGAAGCTCAATCGGCGT
           ************************************************************

KARAT_F    CGGCAACAGCAGGGTCATTAAAATCTCTCGTGCAGCGGGAGCGGCAATGCGACGTCATGT
KARAT_R    CGGCAACAGCAGGGTCATTAAAATCTCTCGTGCAGCGGGAGCGGCAATGCGACGTCATGT
rhl_F      CGGCAACAGCAGGGTCATTAAAATCTCTCGTGCAGCGGGAGCGGCAATGCGACGTCATGT
rhl_R      CGGCAACAGCAGGGTCATTAAAATCTCTCGTGCAGCGGGAGCGGCAATGCGACGTCATGT
           ************************************************************
```

```
KARAT_F      GGTTGCAACGACGACGCCCGTTGTCCTACCTCCTCACGACGGCGATTGTACCTGGCCTAC
KARAT_R      GGTTGCAACGACGACGCCCGTTGTCCTACCTCCTCACGACGGCGATTGTACCTGGCCTAC
rhl_F        GGTTGCAACGACGACGCCCGTTGTCCTACCTCCTCACGACGGCGATTGTACCTGGCCTAC
rhl_R        GGTTGCAACGACGACGCCCGTTGTCCTACCTCCTCACGACGGCGATTGTACCTGGCCTAC
             ************************************************************

KARAT_F      GTTCGGTCAGTTTTGGGGCCGATGCGGGTTGTGGTGGTGGGGCGGGCTGGTGGTGGGGCG
KARAT_R      GTTCGGTCAGTTTTGGGGCCGATGCGGGTTGTGGTGGTGGGGCGGGCTGGTGGTGGGGCG
rhl_F        GTTCGGTCAGTTTTGGGGCCGATGCGGGTTGTGGTGGTGGGGCGGGCTGGTGGTGGGGCG
rhl_R        GTTCGGTCAGTTTTGGGGCCGATGCGGGTTGTGGTGGTGGGGCGGGCTGGTGGTGGGGCG
             ************************************************************

KARAT_F      ACCGTGGTCCAGCTGGCGGCATGATAATCCCTAAGTATAAGGAGAAGCACTCTCCTAGAA
KARAT_R      ACCGTGGTCCAGCTGGCGGCATGATAATCCCTAAGTATAAGGAGAAGCACTCTCCTAGAA
rhl_F        ACCGTGGTCCAGCTGGCGGCATGATAATCCCTAAGTATAAGGAGAAGCACTCTCCTAGAA
rhl_R        ACCGTGGTCCAGCTGGCGGCATGATAATCCCTAAGTATAAGGAGAAGCACTCTCCTAGAA
             ************************************************************

KARAT_F      GGATATGAAGGTATATGAAGTGTCAAACCAAAGGGAGCTAAAGATATTTTTCTTATTCTT
KARAT_R      GGATATGAAGGTATATGAAGTGTCAAACCAAAGGGAGCTAAAGATATTTTTCTTATTCTT
rhl_F        GGATATGAAGGTATATGAAGTGTCAAACCAAAGGGAGCTAAAGATATTTTTCTTATTCTT
rhl_R        GGATATGAAGGTATATGAAGTGTCAAACCAAAGGGAGCTAAAGATATTTTTCTTATTCTT
             ************************************************************

KARAT_F      TCAGGTTTCATATGCTACTTACGCAACTCTATATGTACTTAACATCTATCATACCTAAGC
KARAT_R      TCAGGTTTCATATGCTACTTACGCAACTCTATATGTACTTAACATCTATCATACCTAAGC
rhl_F        TCAGGTTTCATATGCTACTTACGCAACTCTATATGTACTTAACATCTATCATACCTAAGC
rhl_R        TCAGGTTTCATATGCTACTTACGCAACTCTATATGTACTTAACATCTATCATACCTAAGC
             ************************************************************

KARAT_F      TCTCTACAACTAGGATATTATCTGGGAGTAGAAACTAAGGTACTCGCTAAAAAGGTTAGT
KARAT_R      TCTCTACAACTAGGATATTATCTGGGAGTAGAAACTAAGGTACTCGCTAAAAAGGTTAGT
rhl_F        TCTCTACAACTAGGATATTATCTGGGAGTAGAAACTAAGGTACTCGCTAAAAAGGTTAGT
rhl_R        TCTCTACAACTAGGATATTATCTGGGAGTAGAAACTAAGGTACTCGCTAAAAAGGTTAGT
             ************************************************************

KARAT_F      GGATACAAAATAAAAATAAAAATGCTAGTGGCTACAACTAAAAGATTTACAATGATCCTT
KARAT_R      GGATACAAAATAAAAATAAAAATGCTAGTGGCTACAACTAAAAGATTTACAATGATCCTT
rhl_F        GGATACAAAATAAAAATAAAAATGCTAGTGGCTACAACTAAAAGATTTACAATGATCCTT
rhl_R        GGATACAAAATAAAAATAAAAATGCTAGTGGCTACAACTAAAAGATTTACAATGATCCTT
             ************************************************************

KARAT_F      CATACAGTCTGAAATTTATCTTATAAATTAAGTGATTTACTCTTCTCTCTCTCTCTCTCT
KARAT_R      CATACAGTCTGAAATTTATCTTATAAATTAAGTGATTTACTCTTCTCTCTCTCTCTCTCT
rhl_F        CATACAGTCTGAAATTTATCTTATAAATTAAGTGATTTACTCTTCTCTCTCTCTCTCTCT
rhl_R        CATACAGTCTGAAATTTATCTTATAAATTAAGTGATTTACTCTTCTCTCTCTCTCTCTCT
             ************************************************************

KARAT_F      CCCCACTCGCTCGCTCACTCACTCACTCACTCCCCCTCTCTCCCTCTCCTTCCCTCTCTC
KARAT_R      CCCCACTCGCTCGCTCACTCACTCACTCACTCCCCCTCTCTCCCTCTCCTTCCCTCTCTC
rhl_F        CCCCACTCGCTCGCTCACTCACTCACTCACTCCCCCTCTCTCCCTCTCCTTCCCTCTCTC
rhl_R        CCCCACTCGCTCGCTCACTCACTCACTCACTCCCCCTCTCTCCCTCTCCTTCCCTCTCTC
             ************************************************************

KARAT_F      CCTCTCTATCTCTTCCCTCCCTCCCTCCCTTTCTCTATCTCTCTCAAATTGTTCATTGAA
KARAT_R      CCTCTCTATCTCTTCCCTCCCTCCCTCCCTTTCTCTATCTCTCTCAAATTGTTCATTGAA
rhl_F        CCTCTCTATCTCTTCCCTCCCTCCCTCCCTTTCTCTATCTCTCTCAAATTGTTCATTGAA
rhl_R        CCTCTCTATCTCTTCCCTCCCTCCCTCCCTTTCTCTATCTCTCTCAAATTGTTCATTGAA
             ************************************************************

KARAT_F      GTCAATTGTATCTTTTGTGGTTTTATTTGTTGTATAAGGTTTGAGGTTTAGGGTTTAAAT
KARAT_R      GTCAATTGTATCTTTTGTGGTTTTATTTGTTGTATAAGGTTTGAGGTTTAGGGTTTAAAT
rhl_F        GTCAATTGTATCTTTTGTGGTTTTATTTGTTGTATAAGGTTTGAGGTTTAGGGTTTAAAT
rhl_R        GTCAATTGTATCTTTTGTGGTTTTATTTGTTGTATAAGGTTTGAGGTTTAGGGTTTAAAT
             ************************************************************
```

```
KARAT_F      AGATTTGCATGATCTCTAATCTATATATGTCTTCTTTGGCCAAGTTTGACGGCTTTTTCT
KARAT_R      AGATTTGCATGATCTCTAATCTATATATGTCTTCTTTGGCCAAGTTTGACGGCTTTTTCT
rhl_F        AGATTTGCATGATCTCTAATCTATATATGTCTTCTTTGGCCAAGTTTGACGGCTTTTTCT
rhl_R        AGATTTGCATGATCTCTAATCTATATATGTCTTCTTTGGCCAAGTTTGACGGCTTTTTCT
             ************************************************************

KARAT_F      TCGGGGGGGACTAGTGCTTTGCAGTTGGTTCGGTCTTGTGGTGATCCTAGTGACAA
KARAT_R      TCGGGGGGGACTAGTGCTTTGCAGTTGGTTCGGTCTTGTGGTGATCCTAGTGACAA
rhl_F        TCGGGGGGGACTAGTGCTTTGCAGTTGGTTCGGTCTTGTGGTGATCCTAGTGACAA
rhl_R        TCGGGGGGGACTAGTGCTTTGCAGTTGGTTCGGTCTTGTGGTGATCCTAGTGACAA
             ********************************************************
```

protein_HORVU7Hr1G030250_Alignment

```
Karat                MAGGDGGGGGGAQDDFFDQMLSTLPSAWGDLGAGGKSPWEIAAGAEDLGAFDESALLASRLR
rhl1.b_mutant   1    MAGGDGGGGGGAQDDFFDQMLSTLPSAWGDLGAGGKSPWEIAAGAEDLGAFDESALLASRLR
                     **************************************************************

Karat                QHQIGGEKPVMLQLTDLQRQGLGEETGGTGFSPLPLFADRSPPQSREEMDGGFKSPNGTGGD
rhl1.b_mutant   63   QHQIGGEKPVMLQLTDLQRQGLGEETGGTGFSPLPLFADRSPPQSREEMDGGFKSPNGTGGD
                     **************************************************************

Karat                HALFNGFGVHGGAAAVQPTFGQGGSMSGQSFGGGPAASGGTTAPASSGGGGAAPPRQTRVRA
rhl1.b_mutant   125  HALFNGFGVHGGAAAVQPTFGQGGSMSGQSFGGGPAASGGTTAPASSGGGGAAPPRQTRVRA
                     **************************************************************

                                            bHLH domain
Karat                RRGQATDPHSIAERLRRERIAERMKSLQELVPNANKTDKASMLDEIIDYVKFLQLQVKVLSM
rhl1.b_mutant   186  RRGQATDPHSIAERLRRERIAERMKSLQELVPNANKTDKASMLDEIIDYVKFLQLQVKVLSM
                     **************************************************************

Karat                SRLGGAAGMAPLVASMSSEANSSAKSSNGGGNSAAAAAAKANGGGESGGGGGGGGLRVT
rhl1.b_mutant   248  SRLGGAAGMAPLVASMSSEVGVLKASTRSNLRNNLSCRSLCPEARRRGMKCWRTAARRA
                     *******************      *                     *

                                 LRL domain
Karat                EQQVAKMMEEDMGTAMQYLQGKGLCLMPISLASAISSATTTTTSPASLLARQAVRPAST
rhl1.b_mutant   307  ATAAGTARRPRRPRRTAEARAGAVEGAAGCG

Karat                AAALASANGGEDAAARPVKVDAGAASGGKP
rhl1.b_mutant   348
```
