# Supplementary material for: Forward Genetics Approach Reveals a Mutation in bHLH Transcription Factor-Encoding Gene as the Best Candidate for the Root Hairless Phenotype in Barley
Source: Front Plant Sci. 2018 Sep 3;9:1229. doi: 10.3389/fpls.2018.01229 (PMC6129617; doi:10.3389/fpls.2018.01229)
Supplement: FIGURE S7 — The alignments of five HC gene sequences (HORVU7Hr1G030220, HORVU7Hr1G030250, HORVU7Hr1G030270, HORVU7Hr1G030280, and HORVU7Hr1G030290) between brb mutant and its parent variety ‘Pallas’. [file Image_7.pdf]

Alignment_HORVU7Hr1G030220

```
Pallas_F      CGACTGCTACCTCTCCATGTACCTCGCGAAGAAGCCCGACAAGGCCGACCCCAACGAGGC
Pallas_R      CGACTGCTACCTCTCCATGTACCTCGCGAAGAAGCCCGACAAGGCCGACCCCAACGAGGC
Brb_F         CGACTGCTACCTCTCCATGTACCTCGCGAAGAAGCCCGACAAGGCCGACCCCAACGAGGC
Brb_R         CGACTGCTACCTCTCCATGTACCTCGCGAAGAAGCCCGACAAGGCCGACCCCAACGAGGC
              ************************************************************

Pallas_F      CCGCCTCTCCCGCTTCACCTGCTGCGCGCTCTCGGGGGAGCCCCTTGCCCCGCCCGCCGT
Pallas_R      CCGCCTCTCCCGCTTCACCTGCTGCGCGCTCTCGGGGGAGCCCCTTGCCCCGCCCGCCGT
Brb_F         CCGCCTCTCCCGCTTCACCTGCTGCGCGCTCTCGGGGGAGCCCCTTGCCCCGCCCGCCGT
Brb_R         CCGCCTCTCCCGCTTCACCTGCTGCGCGCTCTCGGGGGAGCCCCTTGCCCCGCCCGCCGT
              ************************************************************

Pallas_F      CGTGGATCGGCTGGGCAACCTGTTCAACAAGGAGGCCCTCGTCGAGGCGCTCATCCACAA
Pallas_R      CGTGGATCGGCTGGGCAACCTGTTCAACAAGGAGGCCCTCGTCGAGGCGCTCATCCACAA
Brb_F         CGTGGATCGGCTGGGCAACCTGTTCAACAAGGAGGCCCTCGTCGAGGCGCTCATCCACAA
Brb_R         CGTGGATCGGCTGGGCAACCTGTTCAACAAGGAGGCCCTCGTCGAGGCGCTCATCCACAA
              ************************************************************

Pallas_F      GCGCCTGCCCAAGGCGCTCTCGCACATCCGCGGGCTCAAGGACATGATCCCCATCCACCT
Pallas_R      GCGCCTGCCCAAGGCGCTCTCGCACATCCGCGGGCTCAAGGACATGATCCCCATCCACCT
Brb_F         GCGCCTGCCCAAGGCGCTCTCGCACATCCGCGGGCTCAAGGACATGATCCCCATCCACCT
Brb_R         GCGCCTGCCCAAGGCGCTCTCGCACATCCGCGGGCTCAAGGACATGATCCCCATCCACCT
              ************************************************************

Pallas_F      GCACCCCAAGCCCAACGCGGCGGACCAGGAGGTCCGTTTCCAGTGCCCGGTCACCGGGTT
Pallas_R      GCACCCCAAGCCCAACGCGGCGGACCAGGAGGTCCGTTTCCAGTGCCCGGTCACCGGGTT
Brb_F         GCACCCCAAGCCCAACGCGGCGGACCAGGAGGTCCGTTTCCAGTGCCCGGTCACCGGGTT
Brb_R         GCACCCCAAGCCCAACGCGGCGGACCAGGAGGTCCGTTTCCAGTGCCCGGTCACCGGGTT
              ************************************************************

Pallas_F      TGAGTTCAACGGCAAGTCCCAGTTCCTTGCCCTCCGTGGATGCGGCCACGTGCTGAGCGT
Pallas_R      TGAGTTCAACGGCAAGTCCCAGTTCCTTGCCCTCCGTGGATGCGGCCACGTGCTGAGCGT
Brb_F         TGAGTTCAACGGCAAGTCCCAGTTCCTTGCCCTCCGTGGATGCGGCCACGTGCTGAGCGT
Brb_R         TGAGTTCAACGGCAAGTCCCAGTTCCTTGCCCTCCGTGGATGCGGCCACGTGCTGAGCGT
              ************************************************************

Pallas_F      GAAGGCTCTCAAGGAGGTGAAGTCGTCCTCGTGTTTGGTCTGCCATAAGGAGTTCGTTGA
Pallas_R      GAAGGCTCTCAAGGAGGTGAAGTCGTCCTCGTGTTTGGTCTGCCATAAGGAGTTCGTTGA
Brb_F         GAAGGCTCTCAAGGAGGTGAAGTCGTCCTCGTGTTTGGTCTGCCATAAGGAGTTCGTTGA
Brb_R         GAAGGCTCTCAAGGAGGTGAAGTCGTCCTCGTGTTTGGTCTGCCATAAGGAGTTCGTTGA
              ************************************************************

Pallas_F      GGTGGACAAGATGCCCATCAATGGGACCGAGGAGGAGGTGGAAGTGCTGAGGCAGAGGAT
Pallas_R      GGTGGACAAGATGCCCATCAATGGGACCGAGGAGGAGGTGGAAGTGCTGAGGCAGAGGAT
Brb_F         GGTGGACAAGATGCCCATCAATGGGACCGAGGAGGAGGTGGAAGTGCTGAGGCAGAGGAT
Brb_R         GGTGGACAAGATGCCCATCAATGGGACCGAGGAGGAGGTGGAAGTGCTGAGGCAGAGGAT
              ************************************************************

Pallas_F      GGAGGAGGAGAGAGGGAAGCTGAAGGAGAAGAAGGATAAGAAGCTGTCGAATGGGCTCAG
Pallas_R      GGAGGAGGAGAGAGGGAAGCTGAAGGAGAAGAAGGATAAGAAGCTGTCGAATGGGCTCAG
Brb_F         GGAGGAGGAGAGAGGGAAGCTGAAGGAGAAGAAGGATAAGAAGCTGTCGAATGGGCTCAG
Brb_R         GGAGGAGGAGAGAGGGAAGCTGAAGGAGAAGAAGGATAAGAAGCTGTCGAATGGGCTCAG
              ************************************************************

Pallas_F      TGGGAGTAAGCATGCTGCTGCTGCTTCTGCGGTTGCAGACGCTGAGAAGTTGGAGAATGG
Pallas_R      TGGGAGTAAGCATGCTGCTGCTGCTTCTGCGGTTGCAGACGCTGAGAAGTTGGAGAATGG
Brb_F         TGGGAGTAAGCATGCTGCTGCTGCTTCTGCGGTTGCAGACGCTGAGAAGTTGGAGAATGG
Brb_R         TGGGAGTAAGCATGCTGCTGCTGCTTCTGCGGTTGCAGACGCTGAGAAGTTGGAGAATGG
              ************************************************************

Pallas_F      GAAGAAAGGGGAGGCTGCCCCAGCAAAGCGGTTTAAGGCTGCAGATCATGCACCGGCTCA
Pallas_R      GAAGAAAGGGGAGGCTGCCCCAGCAAAGCGGTTTAAGGCTGCAGATCATGCACCGGCTCA
Brb_F         GAAGAAAGGGGAGGCTGCCCCAGCAAAGCGGTTTAAGGCTGCAGATCATGCACCGGCTCA
Brb_R         GAAGAAAGGGGAGGCTGCCCCAGCAAAGCGGTTTAAGGCTGCAGATCATGCACCGGCTCA
              ************************************************************
```

```
Pallas_F    TGCAAACAAGAAAGTGTATGCATCAATTTTCACTTCCTCCAACAAGTCTGATTTCAGGGA
Pallas_R    TGCAAACAAGAAAGTGTATGCATCAATTTTCACTTCCTCCAACAAGTCTGATTTCAGGGA
Brb_F       TGCAAACAAGAAAGTGTATGCATCAATTTTCACTTCCTCCAACAAGTCTGATTTCAGGGA
Brb_R       TGCAAACAAGAAAGTGTATGCATCAATTTTCACTTCCTCCAACAAGTCTGATTTCAGGGA
            ************************************************************

Pallas_F    AACATACTCATGCCGGTCACTCCCCCTGGGAAGGAATTAAATCGATGTTGGGGAGATTCC
Pallas_R    AACATACTCATGCCGGTCACTCCCCCTGGGAAGGAATTAAATCGATGTTGGGGAGATTCC
Brb_F       AACATACTCATGCCGGTCACTCCCCCTGGGAAGGAATTAAATCGATGTTGGGGAGATTCC
Brb_R       AACATACTCATGCCGGTCACTCCCCCTGGGAAGGAATTAAATCGATGTTGGGGAGATTCC
            ************************************************************

Pallas_F    AATGGCATGGAGGTGAACCTCCTGTACTGATTCAGCGATTAGCTTCATACCATGGCTTGC
Pallas_R    AATGGCATGGAGGTGAACCTCCTGTACTGATTCAGCGATTAGCTTCATACCATGGCTTGC
Brb_F       AATGGCATGGAGGTGAACCTCCTGTACTGATTCAGCGATTAGCTTCATACCATGGCTTGC
Brb_R       AATGGCATGGAGGTGAACCTCCTGTACTGATTCAGCGATTAGCTTCATACCATGGCTTGC
            ************************************************************

Pallas_F    CCATAAGTTCCAGTATTCTTGTTTTAGTTGTTGGTGTTATCTATGCTTGTTCACTCACAG
Pallas_R    CCATAAGTTCCAGTATTCTTGTTTTAGTTGTTGGTGTTATCTATGCTTGTTCACTCACAG
Brb_F       CCATAAGTTCCAGTATTCTTGTTTTAGTTGTTGGTGTTATCTATGCTTGTTCACTCACAG
Brb_R       CCATAAGTTCCAGTATTCTTGTTTTAGTTGTTGGTGTTATCTATGCTTGTTCACTCACAG
            ************************************************************

Pallas_F    AGAAGCTATATGTTTTTATAAATGATTTGTGAAATATGTAATGGTTGAAACATTTGGCAC
Pallas_R    AGAAGCTATATGTTTTTATAAATGATTTGTGAAATATGTAATGGTTGAAACATTTGGCAC
Brb_F       AGAAGCTATATGTTTTTATAAATGATTTGTGAAATATGTAATGGTTGAAACATTTGGCAC
Brb_R       AGAAGCTATATGTTTTTATAAATGATTTGTGAAATATGTAATGGTTGAAACATTTGGCAC
            ************************************************************

Pallas_F    ATATCATTTGGTCCAGTCTTCTCTAGCAATTTGTCTGAGTAATGCTGTAGTTTTCACTCA
Pallas_R    ATATCATTTGGTCCAGTCTTCTCTAGCAATTTGTCTGAGTAATGCTGTAGTTTTCACTCA
Brb_F       ATATCATTTGGTCCAGTCTTCTCTAGCAATTTGTCTGAGTAATGCTGTAGTTTTCACTCA
Brb_R       ATATCATTTGGTCCAGTCTTCTCTAGCAATTTGTCTGAGTAATGCTGTAGTTTTCACTCA
            ************************************************************

Pallas_F    AAGAGAAGCAATATGTTTTTATAAATGCTTTGTGAAACATGTAATGGTTGAAACAGTTAG
Pallas_R    AAGAGAAGCAATATGTTTTTATAAATGCTTTGTGAAACATGTAATGGTTGAAACAGTTAG
Brb_F       AAGAGAAGCAATATGTTTTTATAAATGCTTTGTGAAACATGTAATGGTTGAAACAGTTAG
Brb_R       AAGAGAAGCAATATGTTTTTATAAATGCTTTGTGAAACATGTAATGGTTGAAACAGTTAG
            ************************************************************

Pallas_F    CGCTTATTGTTGGGTCCAGTCTTCTCTAGCAATTTGTCTGAGTAATGCTGTAATTTTCCA
Pallas_R    CGCTTATTGTTGGGTCCAGTCTTCTCTAGCAATTTGTCTGAGTAATGCTGTAATTTTCCA
Brb_F       CGCTTATTGTTGGGTCCAGTCTTCTCTAGCAATTTGTCTGAGTAATGCTGTAATTTTCCA
Brb_R       CGCTTATTGTTGGGTCCAGTCTTCTCTAGCAATTTGTCTGAGTAATGCTGTAATTTTCCA
            ************************************************************

Pallas_F    CTTTGTTTCAAATTTACTCACATCTTAGGTCTCGTCAGGCCAATCCTTTGACCAACAATT
Pallas_R    CTTTGTTTCAAATTTACTCACATCTTAGGTCTCGTCAGGCCAATCCTTTGACCAACAATT
Brb_F       CTTTGTTTCAAATTTACTCACATCTTAGGTCTCGTCAGGCCAATCCTTTGACCAACAATT
Brb_R       CTTTGTTTCAAATTTACTCACATCTTAGGTCTCGTCAGGCCAATCCTTTGACCAACAATT
            ************************************************************

Pallas_F    ATTCTATTAGTAGACCTAAGGGTTCTTAGTGTTCATTGTTGCAACTTCCACTGTATCTTG
Pallas_R    ATTCTATTAGTAGACCTAAGGGTTCTTAGTGTTCATTGTTGCAACTTCCACTGTATCTTG
Brb_F       ATTCTATTAGTAGACCTAAGGGTTCTTAGTGTTCATTGTTGCAACTTCCACTGTATCTTG
Brb_R       ATTCTATTAGTAGACCTAAGGGTTCTTAGTGTTCATTGTTGCAACTTCCACTGTATCTTG
            ************************************************************

Pallas_F    GTCATGCAATCCTACATACAATTTGTTTGAATAAGACTGATTTATGTGCACATGCAAGTG
Pallas_R    GTCATGCAATCCTACATACAATTTGTTTGAATAAGACTGATTTATGTGCACATGCAAGTG
Brb_F       GTCATGCAATCCTACATACAATTTGTTTGAATAAGACTGATTTATGTGCACATGCAAGTG
Brb_R       GTCATGCAATCCTACATACAATTTGTTTGAATAAGACTGATTTATGTGCACATGCAAGTG
            ************************************************************

Pallas_F    GACTCTCAAGATAAATCAAACAGTTTCTATGATTTTCAATGTTGTAATCTAGACTGTGCC
Pallas_R    GACTCTCAAGATAAATCAAACAGTTTCTATGATTTTCAATGTTGTAATCTAGACTGTGCC
Brb_F       GACTCTCAAGATAAATCAAACAGTTTCTATGATTTTCAATGTTGTAATCTAGACTGTGCC
```

```
Brb_R         GACTCTCAAGATAAATCAAACAGTTTCTATGATTTTCAATGTTGTAATCTAGACTGTGCC
              ************************************************************

Pallas_F      ACTTTACTGAACAGATAAATATGCATTGTGCTTGTTTTGATGTATAGTTGTCCATGAAGT
Pallas_R      ACTTTACTGAACAGATAAATATGCATTGTGCTTGTTTTGATGTATAGTTGTCCATGAAGT
Brb_F         ACTTTACTGAACAGATAAATATGCATTGTGCTTGTTTTGATGTATAGTTGTCCATGAAGT
Brb_R         ACTTTACTGAACAGATAAATATGCATTGTGCTTGTTTTGATGTATAGTTGTCCATGAAGT
              ************************************************************

Pallas_F      ACGGAATGCGCGTGAAGACAGTCAGCGGTAGAACATTTGGCGCCACATTAATACTAGCAT
Pallas_R      ACGGAATGCGCGTGAAGACAGTCAGCGGTAGAACATTTGGCGCCACATTAATACTAGCAT
Brb_F         ACGGAATGCGCGTGAAGACAGTCAGCGGTAGAACATTTGGCGCCACATTAATACTAGCAT
Brb_R         ACGGAATGCGCGTGAAGACAGTCAGCGGTAGAACATTTGGCGCCACATTAATACTAGCAT
              ************************************************************

Pallas_F      GGTAGTTGCTCTTTACATGCTTCTTTAGATATGCTGTCAAGCTAACCTTGGACATGGTGA
Pallas_R      GGTAGTTGCTCTTTACATGCTTCTTTAGATATGCTGTCAAGCTAACCTTGGACATGGTGA
Brb_F         GGTAGTTGCTCTTTACATGCTTCTTTAGATATGCTGTCAAGCTAACCTTGGACATGGTGA
Brb_R         GGTAGTTGCTCTTTACATGCTTCTTTAGATATGCTGTCAAGCTAACCTTGGACATGGTGA
              ************************************************************

Pallas_F      AAGTTTGCCTATTTTCTTTCAAACCTTCTCCTTTGGGCCTTTATTTCCATAACATGTTTA
Pallas_R      AAGTTTGCCTATTTTCTTTCAAACCTTCTCCTTTGGGCCTTTATTTCCATAACATGTTTA
Brb_F         AAGTTTGCCTATTTTCTTTCAAACCTTCTCCTTTGGGCCTTTATTTCCATAACATGTTTA
Brb_R         AAGTTTGCCTATTTTCTTTCAAACCTTCTCCTTTGGGCCTTTATTTCCATAACATGTTTA
              ************************************************************

Pallas_F      TAATGTACACATGTTATATTTCTCGTTATACCTTTGTCATTTGGGCTATACTATGAATTG
Pallas_R      TAATGTACACATGTTATATTTCTCGTTATACCTTTGTCATTTGGGCTATACTATGAATTG
Brb_F         TAATGTACACATGTTATATTTCTCGTTATACCTTTGTCATTTGGGCTATACTATGAATTG
Brb_R         TAATGTACACATGTTATATTTCTCGTTATACCTTTGTCATTTGGGCTATACTATGAATTG
              ************************************************************

Pallas_F      TAACGTACATACTTATGTAGCTTTTGCTGAATTTACATATTTGCTAATTGTGGATTTTAT
Pallas_R      TAACGTACATACTTATGTAGCTTTTGCTGAATTTACATATTTGCTAATTGTGGATTTTAT
Brb_F         TAACGTACATACTTATGTAGCTTTTGCTGAATTTACATATTTGCTAATTGTGGATTTTAT
Brb_R         TAACGTACATACTTATGTAGCTTTTGCTGAATTTACATATTTGCTAATTGTGGATTTTAT
              ************************************************************

Pallas_F      AAAAAGATCCAAAATAGCTATAATCAATCTACTTCTGCCCTGTATGCACATTGCTGAATA
Pallas_R      AAAAAGATCCAAAATAGCTATAATCAATCTACTTCTGCCCTGTATGCACATTGCTGAATA
Brb_F         AAAAAGATCCAAAATAGCTATAATCAATCTACTTCTGCCCTGTATGCACATTGCTGAATA
Brb_R         AAAAAGATCCAAAATAGCTATAATCAATCTACTTCTGCCCTGTATGCACATTGCTGAATA
              ************************************************************

Pallas_F      AGATTCTGCAAAGATTCATAGGATGCCATGAACTTACTGAGATATCAAGACTAGAGTTGA
Pallas_R      AGATTCTGCAAAGATTCATAGGATGCCATGAACTTACTGAGATATCAAGACTAGAGTTGA
Brb_F         AGATTCTGCAAAGATTCATAGGATGCCATGAACTTACTGAGATATCAAGACTAGAGTTGA
Brb_R         AGATTCTGCAAAGATTCATAGGATGCCATGAACTTACTGAGATATCAAGACTAGAGTTGA
              ************************************************************

Pallas_F      TTTGGACACACAGGAATTTGACACCTCAGTTGTCTAACTCTGTTCAAATTTAGAAGGAAT
Pallas_R      TTTGGACACACAGGAATTTGACACCTCAGTTGTCTAACTCTGTTCAAATTTAGAAGGAAT
Brb_F         TTTGGACACACAGGAATTTGACACCTCAGTTGTCTAACTCTGTTCAAATTTAGAAGGAAT
Brb_R         TTTGGACACACAGGAATTTGACACCTCAGTTGTCTAACTCTGTTCAAATTTAGAAGGAAT
              ************************************************************

Pallas_F      GGTGAAAATAGCTCCTATCAAGAAGACATCCAAATTTATTTTGCGTTAACATGCGGTACC
Pallas_R      GGTGAAAATAGCTCCTATCAAGAAGACATCCAAATTTATTTTGCGTTAACATGCGGTACC
Brb_F         GGTGAAAATAGCTCCTATCAAGAAGACATCCAAATTTATTTTGCGTTAACATGCGGTACC
Brb_R         GGTGAAAATAGCTCCTATCAAGAAGACATCCAAATTTATTTTGCGTTAACATGCGGTACC
              ************************************************************

Pallas_F      TCCCTCAGACTATTTGTAGTTGGACACACATTAGTTTGTTTTTCTTATTCCATACTTGCT
Pallas_R      TCCCTCAGACTATTTGTAGTTGGACACACATTAGTTTGTTTTTCTTATTCCATACTTGCT
Brb_F         TCCCTCAGACTATTTGTAGTTGGACACACATTAGTTTGTTTTTCTTATTCCATACTTGCT
Brb_R         TCCCTCAGACTATTTGTAGTTGGACACACATTAGTTTGTTTTTCTTATTCCATACTTGCT
              ************************************************************
```

```
Pallas_F        TTGAAGGGGAGCCGTGGCGCAGCGGCAAAGCTGTTGCCTTGTGACCATTA
Pallas_R        TTGAAGGGGAGCCGTGGCGCAGCGGCAAAGCTGTTGCCTTGTGACCATTA
Brb_F           TTGAAGGGGAGCCGTGGCGCAGCGGCAAAGCTGTTGCCTTGTGACCATTA
Brb_R           TTGAAGGGGAGCCGTGGCGCAGCGGCAAAGCTGTTGCCTTGTGACCATTA
                **************************************************
```

Alignment_HORVU7Hr1G030250

```
PALLAS_F      TGTGGCCTATTTTACAGGGTCTTGTTTGTATGGTGGTGCTTGACACAAATTGTCCCCGGT
PALLAS_R      TGTGGCCTATTTTACAGGGTCTTGTTTGTATGGTGGTGCTTGACACAAATTGTCCCCGGT
BRB_F         TGTGGCCTATTTTACAGGGTCTTGTTTGTATGGTGGTGCTTGACACAAATTGTCCCCGGT
BRB_R         TGTGGCCTATTTTACAGGGTCTTGTTTGTATGGTGGTGCTTGACACAAATTGTCCCCGGT
              ************************************************************

PALLAS_F      GAATAACGTTGCTTCAAAGACTGTGGATTAGGGGCATGCGTTGATTAGGAGATGGTCGGC
PALLAS_R      GAATAACGTTGCTTCAAAGACTGTGGATTAGGGGCATGCGTTGATTAGGAGATGGTCGGC
BRB_F         GAATAACGTTGCTTCAAAGACTGTGGATTAGGGGCATGCGTTGATTAGGAGATGGTCGGC
BRB_R         GAATAACGTTGCTTCAAAGACTGTGGATTAGGGGCATGCGTTGATTAGGAGATGGTCGGC
              ************************************************************

PALLAS_F      TCTTTGGTTTAATTAATTATCATCATACTAACATTTTGTTATCTCAATGGTAACCTAAGT
PALLAS_R      TCTTTGGTTTAATTAATTATCATCATACTAACATTTTGTTATCTCAATGGTAACCTAAGT
BRB_F         TCTTTGGTTTAATTAATTATCATCATACTAACATTTTGTTATCTCAATGGTAACCTAAGT
BRB_R         TCTTTGGTTTAATTAATTATCATCATACTAACATTTTGTTATCTCAATGGTAACCTAAGT
              ************************************************************

PALLAS_F      CCATGGTACATGAAAATATGTGTGTGTTTGTGGGAACAAAAAAATGTGGGTACTCTAAAC
PALLAS_R      CCATGGTACATGAAAATATGTGTGTGTTTGTGGGAACAAAAAAATGTGGGTACTCTAAAC
BRB_F         CCATGGTACATGAAAATATGTGTGTGTTTGTGGGAACAAAAAAATGTGGGTACTCTAAAC
BRB_R         CCATGGTACATGAAAATATGTGTGTGTTTGTGGGAACAAAAAAATGTGGGTACTCTAAAC
              ************************************************************

PALLAS_F      AGAAGTGTCGCCATCGTCCGAACACCGCCCGTGTGAGGACTAAAAAGAAACTTAACCTAA
PALLAS_R      AGAAGTGTCGCCATCGTCCGAACACCGCCCGTGTGAGGACTAAAAAGAAACTTAACCTAA
BRB_F         AGAAGTGTCGCCATCGTCCGAACACCGCCCGTGTGAGGACTAAAAAGAAACTTAACCTAA
BRB_R         AGAAGTGTCGCCATCGTCCGAACACCGCCCGTGTGAGGACTAAAAAGAAACTTAACCTAA
              ************************************************************

PALLAS_F      ACTACAAGCTCGGGCCGGAGCCGAGGCACTCGAATTTTCCTCACCGCGAGGTGCGGAGGG
PALLAS_R      ACTACAAGCTCGGGCCGGAGCCGAGGCACTCGAATTTTCCTCACCGCGAGGTGCGGAGGG
BRB_F         ACTACAAGCTCGGGCCGGAGCCGAGGCACTCGAATTTTCCTCACCGCGAGGTGCGGAGGG
BRB_R         ACTACAAGCTCGGGCCGGAGCCGAGGCACTCGAATTTTCCTCACCGCGAGGTGCGGAGGG
              ************************************************************

PALLAS_F      AAGACGAATCTACGAGGTCATTGATGGTGCTCGGAGGGAGGTGTATCCCCTAAAGGAAAA
PALLAS_R      AAGACGAATCTACGAGGTCATTGATGGTGCTCGGAGGGAGGTGTATCCCCTAAAGGAAAA
BRB_F         AAGACGAATCTACGAGGTCATTGATGGTGCTCGGAGGGAGGTGTATCCCCTAAAGGAAAA
BRB_R         AAGACGAATCTACGAGGTCATTGATGGTGCTCGGAGGGAGGTGTATCCCCTAAAGGAAAA
              ************************************************************

PALLAS_F      AATAAATAGATATTTTTTAAATAATACATTTATTTTCATGATTTTGGAAAATAATATGCA
PALLAS_R      AATAAATAGATATTTTTTAAATAATACATTTATTTTCATGATTTTGGAAAATAATATGCA
BRB_F         AATAAATAGATATTTTTTAAATAATACATTTATTTTCATGATTTTGGAAAATAATATGCA
BRB_R         AATAAATAGATATTTTTTAAATAATACATTTATTTTCATGATTTTGGAAAATAATATGCA
              ************************************************************

PALLAS_F      TGTTGAAAGTTTTTCGAAATTAGAATTCAGAAGGCTTCGGCAAAGCCAGTAGGTTCCAGT
PALLAS_R      TGTTGAAAGTTTTTCGAAATTAGAATTCAGAAGGCTTCGGCAAAGCCAGTAGGTTCCAGT
BRB_F         TGTTGAAAGTTTTTCGAAATTAGAATTCAGAAGGCTTCGGCAAAGCCAGTAGGTTCCAGT
BRB_R         TGTTGAAAGTTTTTCGAAATTAGAATTCAGAAGGCTTCGGCAAAGCCAGTAGGTTCCAGT
              ************************************************************

PALLAS_F      CGGCTTCGACGAAGCTGACAAGTTCCAATCCGCTTCGGCGAAACCAATAGGTACGAGTCA
PALLAS_R      CGGCTTCGACGAAGCTGACAAGTTCCAATCCGCTTCGGCGAAACCAATAGGTACGAGTCA
BRB_F         CGGCTTCGACGAAGCTGACAAGTTCCAATCCGCTTCGGCGAAACCAATAGGTACGAGTCA
BRB_R         CGGCTTCGACGAAGCTGACAAGTTCCAATCCGCTTCGGCGAAACCAATAGGTACGAGTCA
              ************************************************************

PALLAS_F      GCTTTGGTGAAGCTAATAGGATTTCAGTGGGCTTCAACGAAGCCAATTAGTACCAATTGT
PALLAS_R      GCTTTGGTGAAGCTAATAGGATTTCAGTGGGCTTCAACGAAGCCAATTAGTACCAATTGT
BRB_F         GCTTTGGTGAAGCTAATAGGATTTCAGTGGGCTTCAACGAAGCCAATTAGTACCAATTGT
BRB_R         GCTTTGGTGAAGCTAATAGGATTTCAGTGGGCTTCAACGAAGCCAATTAGTACCAATTGT
              ************************************************************
```

```
PALLAS_F      ATTTGTCAAAATCAATTAGTGCATGCCACATATTTTGCATCGTAATTTTATCCCTAATTT
PALLAS_R      ATTTGTCAAAATCAATTAGTGCATGCCACATATTTTGCATCGTAATTTTATCCCTAATTT
BRB_F         ATTTGTCAAAATCAATTAGTGCATGCCACATATTTTGCATCGTAATTTTATCCCTAATTT
BRB_R         ATTTGTCAAAATCAATTAGTGCATGCCACATATTTTGCATCGTAATTTTATCCCTAATTT
              ************************************************************

PALLAS_F      TCCGCTACCCTGTCCTTAGTTTACTACCACCCTCTCCCTAGGTTCTCGCCACCTTTTTCC
PALLAS_R      TCCGCTACCCTGTCCTTAGTTTACTACCACCCTCTCCCTAGGTTCTCGCCACCTTTTTCC
BRB_F         TCCGCTACCCTGTCCTTAGTTTACTACCACCCTCTCCCTAGGTTCTCGCCACCTTTTTCC
BRB_R         TCCGCTACCCTGTCCTTAGTTTACTACCACCCTCTCCCTAGGTTCTCGCCACCTTTTTCC
              ************************************************************

PALLAS_F      TCCATTCTTTCCCGCCATCTTCTTTCACGTCATTTTCTTTCCCGCTATTCTTTCCCTAAT
PALLAS_R      TCCATTCTTTCCCGCCATCTTCTTTCACGTCATTTTCTTTCCCGCTATTCTTTCCCTAAT
BRB_F         TCCATTCTTTCCCGCCATCTTCTTTCACGTCATTTTCTTTCCCGCTATTCTTTCCCTAAT
BRB_R         TCCATTCTTTCCCGCCATCTTCTTTCACGTCATTTTCTTTCCCGCTATTCTTTCCCTAAT
              ************************************************************

PALLAS_F      TGAATAAGGACAATAGAATAGTTTGATGGAACGAACTCATAAGTAGTGACACCCTACACA
PALLAS_R      TGAATAAGGACAATAGAATAGTTTGATGGAACGAACTCATAAGTAGTGACACCCTACACA
BRB_F         TGAATAAGGACAATAGAATAGTTTGATGGAACGAACTCATAAGTAGTGACACCCTACACA
BRB_R         TGAATAAGGACAATAGAATAGTTTGATGGAACGAACTCATAAGTAGTGACACCCTACACA
              ************************************************************

PALLAS_F      ATGAGCTGGGTCAAACATTAACTCGTTGTGGGTGGCCTTCACGGACCTTTGAGGATATAC
PALLAS_R      ATGAGCTGGGTCAAACATTAACTCGTTGTGGGTGGCCTTCACGGACCTTTGAGGATATAC
BRB_F         ATGAGCTGGGTCAAACATTAACTCGTTGTGGGTGGCCTTCACGGACCTTTGAGGATATAC
BRB_R         ATGAGCTGGGTCAAACATTAACTCGTTGTGGGTGGCCTTCACGGACCTTTGAGGATATAC
              ************************************************************

PALLAS_F      GGAAAGAAATTCTCAGGTTGCATGAAAGATGGATGAAGCAACACCATAAGAGTGAATCAT
PALLAS_R      GGAAAGAAATTCTCAGGTTGCATGAAAGATGGATGAAGCAACACCATAAGAGTGAATCAT
BRB_F         GGAAAGAAATTCTCAGGTTGCATGAAAGATGGATGAAGCAACACCATAAGAGTGAATCAT
BRB_R         GGAAAGAAATTCTCAGGTTGCATGAAAGATGGATGAAGCAACACCATAAGAGTGAATCAT
              ************************************************************

PALLAS_F      TCCTAAAGTATGCAACAGAACATAATTTTTTATGGAGTGCCGACAACAATGACGAAGACG
PALLAS_R      TCCTAAAGTATGCAACAGAACATAATTTTTTATGGAGTGCCGACAACAATGACGAAGACG
BRB_F         TCCTAAAGTATGCAACAGAACATAATTTTTTATGGAGTGCCGACAACAATGACGAAGACG
BRB_R         TCCTAAAGTATGCAACAGAACATAATTTTTTATGGAGTGCCGACAACAATGACGAAGACG
              ************************************************************

PALLAS_F      ATATCTTCATGCCGAGCACCAAGGCCAAGACTACCACATCGTCGAAGGGCAAGAATGTTG
PALLAS_R      ATATCTTCATGCCGAGCACCAAGGCCAAGACTACCACATCGTCGAAGGGCAAGAATGTTG
BRB_F         ATATCTTCATGCCGAGCACCAAGGCCAAGACTACCACATCGTCGAAGGGCAAGAATGTTG
BRB_R         ATATCTTCATGCCGAGCACCAAGGCCAAGACTACCACATCGTCGAAGGGCAAGAATGTTG
              ************************************************************

PALLAS_F      CATCGACGGAGGATGCCGATGATGGCTTCATGTAGTTTTTAAGCTGTAGTTTTTAAGTTC
PALLAS_R      CATCGACGGAGGATGCCGATGATGGCTTCATGTAGTTTTTAAGCTGTAGTTTTTAAGTTC
BRB_F         CATCGACGGAGGATGCCGATGATGGCTTCATGTAGTTTTTAAGCTGTAGTTTTTAAGTTC
BRB_R         CATCGACGGAGGATGCCGATGATGGCTTCATGTAGTTTTTAAGCTGTAGTTTTTAAGTTC
              ************************************************************

PALLAS_F      AGTCCTACCGTTTAATTCAGTTGTACTTGTATCTTAATTATCTATTGTGGTTTCTTTGAA
PALLAS_R      AGTCCTACCGTTTAATTCAGTTGTACTTGTATCTTAATTATCTATTGTGGTTTCTTTGAA
BRB_F         AGTCCTACCGTTTAATTCAGTTGTACTTGTATCTTAATTATCTATTGTGGTTTCTTTGAA
BRB_R         AGTCCTACCGTTTAATTCAGTTGTACTTGTATCTTAATTATCTATTGTGGTTTCTTTGAA
              ************************************************************

PALLAS_F      AATGTGACTCGAATAATAATGAGGTTTCAATGGCCTCAACCACACAATACATTGGGAGAT
PALLAS_R      AATGTGACTCGAATAATAATGAGGTTTCAATGGCCTCAACCACACAATACATTGGGAGAT
BRB_F         AATGTGACTCGAATAATAATGAGGTTTCAATGGCCTCAACCACACAATACATTGGGAGAT
BRB_R         AATGTGACTCGAATAATAATGAGGTTTCAATGGCCTCAACCACACAATACATTGGGAGAT
              ************************************************************

PALLAS_F      ACAAGGATGAAAACTACCATCAAAATGAAAATCGGACATCTTCACGAGACTATACCGAAC
PALLAS_R      ACAAGGATGAAAACTACCATCAAAATGAAAATCGGACATCTTCACGAGACTATACCGAAC
BRB_F         ACAAGGATGAAAACTACCATCAAAATGAAAATCGGACATCTTCACGAGACTATACCGAAC
```

```
BRB_R           ACAAGGATGAAAACTACCATCAAAATGAAAATCGGACATCTTCACGAGACTATACCGAAC
                ************************************************************

PALLAS_F        TACGATCAAAATGACGGAAAAGAAGATGACGGAAAAGAATGGCATGAATAAGTGTACAGA
PALLAS_R        TACGATCAAAATGACGGAAAAGAAGATGACGGAAAAGAATGGCATGAATAAGTGTACAGA
BRB_F           TACGATCAAAATGACGGAAAAGAAGATGACGGAAAAGAATGGCATGAATAAGTGTACAGA
BRB_R           TACGATCAAAATGACGGAAAAGAAGATGACGGAAAAGAATGGCATGAATAAGTGTACAGA
                ************************************************************

PALLAS_F        AAAGGTAGCGGGAAACTAGGAAGAGGTAGGGGAAAACTAGGGATAAAATTACGATGAAAA
PALLAS_R        AAAGGTAGCGGGAAACTAGGAAGAGGTAGGGGAAAACTAGGGATAAAATTACGATGAAAA
BRB_F           AAAGGTAGCGGGAAACTAGGAAGAGGTAGGGGAAAACTAGGGATAAAATTACGATGAAAA
BRB_R           AAAGGTAGCGGGAAACTAGGAAGAGGTAGGGGAAAACTAGGGATAAAATTACGATGAAAA
                ************************************************************

PALLAS_F        ATATATGGCATGCATTAATTGGTTTCGGCGAAGCTAATTAGTACTAATTAGCTTCATGGT
PALLAS_R        ATATATGGCATGCATTAATTGGTTTCGGCGAAGCTAATTAGTACTAATTAGCTTCATGGT
BRB_F           ATATATGGCATGCATTAATTGGTTTCGGCGAAGCTAATTAGTACTAATTAGCTTCATGGT
BRB_R           ATATATGGCATGCATTAATTGGTTTCGGCGAAGCTAATTAGTACTAATTAGCTTCATGGT
                ************************************************************

PALLAS_F        ATATGTTAGCTTCGCTGAAGCTGACTAGAATCTATCAGCTTCGTCGAAGCCGACTGGATT
PALLAS_R        ATATGTTAGCTTCGCTGAAGCTGACTAGAATCTATCAGCTTCGTCGAAGCCGACTGGATT
BRB_F           ATATGTTAGCTTCGCTGAAGCTGACTAGAATCTATCAGCTTCGTCGAAGCCGACTGGATT
BRB_R           ATATGTTAGCTTCGCTGAAGCTGACTAGAATCTATCAGCTTCGTCGAAGCCGACTGGATT
                ************************************************************

PALLAS_F        CTATTTTAAAAAACTTATAACTTGCGTATTACTTCCTCCGTTTTAAAATTCTTGTCTTAA
PALLAS_R        CTATTTTAAAAAACTTATAACTTGCGTATTACTTCCTCCGTTTTAAAATTCTTGTCTTAA
BRB_F           CTATTTTAAAAAACTTATAACTTGCGTATTACTTCCTCCGTTTTAAAATTCTTGTCTTAA
BRB_R           CTATTTTAAAAAACTTATAACTTGCGTATTACTTCCTCCGTTTTAAAATTCTTGTCTTAA
                ************************************************************

PALLAS_F        ATTTATTTAGATGTGAATGTATCTAGTCAAATTTTAGTATTTTGATATATTTATTTCTAG
PALLAS_R        ATTTATTTAGATGTGAATGTATCTAGTCAAATTTTAGTATTTTGATATATTTATTTCTAG
BRB_F           ATTTATTTAGATGTGAATGTATCTAGTCAAATTTTAGTATTTTGATATATTTATTTCTAG
BRB_R           ATTTATTTAGATGTGAATGTATCTAGTCAAATTTTAGTATTTTGATATATTTATTTCTAG
                ************************************************************

PALLAS_F        GCAAAGCTAAGACAAGAATTTTAGGACGGAAGGAGTATTTATTAAAATCATAAAAATAAA
PALLAS_R        GCAAAGCTAAGACAAGAATTTTAGGACGGAAGGAGTATTTATTAAAATCATAAAAATAAA
BRB_F           GCAAAGCTAAGACAAGAATTTTAGGACGGAAGGAGTATTTATTAAAATCATAAAAATAAA
BRB_R           GCAAAGCTAAGACAAGAATTTTAGGACGGAAGGAGTATTTATTAAAATCATAAAAATAAA
                ************************************************************

PALLAS_F        TGTATTATTTTAAAAAATAGCAAAGAAATAGAGCACTGACAATCAAATTACTGAAATCCC
PALLAS_R        TGTATTATTTTAAAAAATAGCAAAGAAATAGAGCACTGACAATCAAATTACTGAAATCCC
BRB_F           TGTATTATTTTAAAAAATAGCAAAGAAATAGAGCACTGACAATCAAATTACTGAAATCCC
BRB_R           TGTATTATTTTAAAAAATAGCAAAGAAATAGAGCACTGACAATCAAATTACTGAAATCCC
                ************************************************************

PALLAS_F        ATGTTTATACCAGCTGTAATTTGCAGCTCTTAAGCAAAGTCAGAAAAAAAAAGTAGACAG
PALLAS_R        ATGTTTATACCAGCTGTAATTTGCAGCTCTTAAGCAAAGTCAGAAAAAAAAAGTAGACAG
BRB_F           ATGTTTATACCAGCTGTAATTTGCAGCTCTTAAGCAAAGTCAGAAAAAAAAAGTAGACAG
BRB_R           ATGTTTATACCAGCTGTAATTTGCAGCTCTTAAGCAAAGTCAGAAAAAAAAAGTAGACAG
                ************************************************************

PALLAS_F        AAGGTCAGTATATAAACGCCAAAGAGTTGCACGAACCGAACTCAGTTAGTTTTCCATTTG
PALLAS_R        AAGGTCAGTATATAAACGCCAAAGAGTTGCACGAACCGAACTCAGTTAGTTTTCCATTTG
BRB_F           AAGGTCAGTATATAAACGCCAAAGAGTTGCACGAACCGAACTCAGTTAGTTTTCCATTTG
BRB_R           AAGGTCAGTATATAAACGCCAAAGAGTTGCACGAACCGAACTCAGTTAGTTTTCCATTTG
                ************************************************************

PALLAS_F        AAAATTGGCACCTTAGCACGGGATTATAAAGGCGCGCACGTAGCGAAGCTAGTTCGCCTC
PALLAS_R        AAAATTGGCACCTTAGCACGGGATTATAAAGGCGCGCACGTAGCGAAGCTAGTTCGCCTC
BRB_F           AAAATTGGCACCTTAGCACGGGATTATAAAGGCGCGCACGTAGCGAAGCTAGTTCGCCTC
BRB_R           AAAATTGGCACCTTAGCACGGGATTATAAAGGCGCGCACGTAGCGAAGCTAGTTCGCCTC
                ************************************************************
```

```
PALLAS_F      GCCTCCTCCATACTCTCCTCCTGCCTTCTCGCGACCGCATTTACCCGCCAGCTCGTAGCT
PALLAS_R      GCCTCCTCCATACTCTCCTCCTGCCTTCTCGCGACCGCATTTACCCGCCAGCTCGTAGCT
BRB_F         GCCTCCTCCATACTCTCCTCCTGCCTTCTCGCGACCGCATTTACCCGCCAGCTCGTAGCT
BRB_R         GCCTCCTCCATACTCTCCTCCTGCCTTCTCGCGACCGCATTTACCCGCCAGCTCGTAGCT
              ************************************************************

PALLAS_F      GCACAGCCCGGGGACGCAGCCCGCGCGAGAGACCGATGGCAGGCGGTGACGGCGGCGGGG
PALLAS_R      GCACAGCCCGGGGACGCAGCCCGCGCGAGAGACCGATGGCAGGCGGTGACGGCGGCGGGG
BRB_F         GCACAGCCCGGGGACGCAGCCCGCGCGAGAGACCGATGGCAGGCGGTGACGGCGGCGGGG
BRB_R         GCACAGCCCGGGGACGCAGCCCGCGCGAGAGACCGATGGCAGGCGGTGACGGCGGCGGGG
              ************************************************************

PALLAS_F      GCGGCGGCGCGCAGGACGACTTCTTCGACCAGATGCTGTCGACGCTGCCCTCCGCCTGGG
PALLAS_R      GCGGCGGCGCGCAGGACGACTTCTTCGACCAGATGCTGTCGACGCTGCCCTCCGCCTGGG
BRB_F         GCGGCGGCGCGCAGGACGACTTCTTCGACCAGATGCTGTCGACGCTGCCCTCCGCCTGGG
BRB_R         GCGGCGGCGCGCAGGACGACTTCTTCGACCAGATGCTGTCGACGCTGCCCTCCGCCTGGG
              ************************************************************

PALLAS_F      GCGACCTTGGCGCCGGCGGGAAGTCGCCCTGGGAGATCGCGGCCGGCGCCGAGGACCTCG
PALLAS_R      GCGACCTTGGCGCCGGCGGGAAGTCGCCCTGGGAGATCGCGGCCGGCGCCGAGGACCTCG
BRB_F         GCGACCTTGGCGCCGGCGGGAAGTCGCCCTGGGAGATCGCGGCCGGCGCCGAGGACCTCG
BRB_R         GCGACCTTGGCGCCGGCGGGAAGTCGCCCTGGGAGATCGCGGCCGGCGCCGAGGACCTCG
              ************************************************************

PALLAS_F      GAGCCTTCGACGAGTCGGCGCTGCTCGCGTCCAGGCTCCGGCAGCACCAGATCGGCGGAG
PALLAS_R      GAGCCTTCGACGAGTCGGCGCTGCTCGCGTCCAGGCTCCGGCAGCACCAGATCGGCGGAG
BRB_F         GAGCCTTCGACGAGTCGGCGCTGCTCGCGTCCAGGCTCCGGCAGCACCAGATCGGCGGAG
BRB_R         GAGCCTTCGACGAGTCGGCGCTGCTCGCGTCCAGGCTCCGGCAGCACCAGATCGGCGGAG
              ************************************************************

PALLAS_F      AGAAGCCGGTGATGCTGCAGCTCACCGACCTCCAGCGGCAGGGCCTCGGCGAGGAGACCG
PALLAS_R      AGAAGCCGGTGATGCTGCAGCTCACCGACCTCCAGCGGCAGGGCCTCGGCGAGGAGACCG
BRB_F         AGAAGCCGGTGATGCTGCAGCTCACCGACCTCCAGCGGCAGGGCCTCGGCGAGGAGACCG
BRB_R         AGAAGCCGGTGATGCTGCAGCTCACCGACCTCCAGCGGCAGGGCCTCGGCGAGGAGACCG
              ************************************************************

PALLAS_F      GCGGCACGGGGTTCTCGCCGCTGCCGCTGTTCGCGGACCGGTCGCCCCCGCAGTCGCGGG
PALLAS_R      GCGGCACGGGGTTCTCGCCGCTGCCGCTGTTCGCGGACCGGTCGCCCCCGCAGTCGCGGG
BRB_F         GCGGCACGGGGTTCTCGCCGCTGCCGCTGTTCGCGGACCGGTCGCCCCCGCAGTCGCGGG
BRB_R         GCGGCACGGGGTTCTCGCCGCTGCCGCTGTTCGCGGACCGGTCGCCCCCGCAGTCGCGGG
              ************************************************************

PALLAS_F      AGGAGATGGACGGCGGCTTCAAGTCGCCCAATGGCACGGTATAGGATCCTTTTTCATCCT
PALLAS_R      AGGAGATGGACGGCGGCTTCAAGTCGCCCAATGGCACGGTATAGGATCCTTTTTCATCCT
BRB_F         AGGAGATGGACGGCGGCTTCAAGTCGCCCAATGGCACGGTATAGGATCCTTTTTCATCCT
BRB_R         AGGAGATGGACGGCGGCTTCAAGTCGCCCAATGGCACGGTATAGGATCCTTTTTCATCCT
              ************************************************************

PALLAS_F      CGTTCTCTCTCTGCCGTTCTTAATTTCTCTAGTCAAAATTTCCCTTGACGACATGGACTC
PALLAS_R      CGTTCTCTCTCTGCCGTTCTTAATTTCTCTAGTCAAAATTTCCCTTGACGACATGGACTC
BRB_F         CGTTCTCTCTCTGCCGTTCTTAATTTCTCTAGTCAAAATTTCCCTTGACGACATGGACTC
BRB_R         CGTTCTCTCTCTGCCGTTCTTAATTTCTCTAGTCAAAATTTCCCTTGACGACATGGACTC
              ************************************************************

PALLAS_F      GTTTTGTGCGGGCGCGTGCGTGCATGCATGCAGGGAGGTGACCACGCGCTGTTCAACGGA
PALLAS_R      GTTTTGTGCGGGCGCGTGCGTGCATGCATGCAGGGAGGTGACCACGCGCTGTTCAACGGA
BRB_F         GTTTTGTGCGGGCGCGTGCGTGCATGCATGCAGGGAGGTGACCACGCGCTGTTCAACGGA
BRB_R         GTTTTGTGCGGGCGCGTGCGTGCATGCATGCAGGGAGGTGACCACGCGCTGTTCAACGGA
              ************************************************************

PALLAS_F      TTTGGGGTGCATGGCGGCGCCGCCGCGGTGCAGCCGACGTTCGGCCAGGTACGAACTTGC
PALLAS_R      TTTGGGGTGCATGGCGGCGCCGCCGCGGTGCAGCCGACGTTCGGCCAGGTACGAACTTGC
BRB_F         TTTGGGGTGCATGGCGGCGCCGCCGCGGTGCAGCCGACGTTCGGCCAGGTACGAACTTGC
BRB_R         TTTGGGGTGCATGGCGGCGCCGCCGCGGTGCAGCCGACGTTCGGCCAGGTACGAACTTGC
              ************************************************************

PALLAS_F      ATGCACCTGTACTACGATCCACGCACGACCGTGTGAAGCAGCACCATATAATCGCATGTT
PALLAS_R      ATGCACCTGTACTACGATCCACGCACGACCGTGTGAAGCAGCACCATATAATCGCATGTT
BRB_F         ATGCACCTGTACTACGATCCACGCACGACCGTGTGAAGCAGCACCATATAATCGCATGTT
```

```
BRB_R           ATGCACCTGTACTACGATCCACGCACGACCGTGTGAAGCAGCACCATATAATCGCATGTT
                ************************************************************

PALLAS_F        CTTTGATCGCTGGCAGGGAGGATCAATGTCGGGGCAGAGCTTCGGAGGAGGGCCGGCGGC
PALLAS_R        CTTTGATCGCTGGCAGGGAGGATCAATGTCGGGGCAGAGCTTCGGAGGAGGGCCGGCGGC
BRB_F           CTTTGATCGCTGGCAGGGAGGATCAATGTCGGGGCAGAGCTTCGGAGGAGGGCCGGCGGC
BRB_R           CTTTGATCGCTGGCAGGGAGGATCAATGTCGGGGCAGAGCTTCGGAGGAGGGCCGGCGGC
                ************************************************************

PALLAS_F        GAGCGGAGGCACGACAGCGCCCGCCTCCTCTGGCGGCGGCGCGGCCCCGCCGCGGCAGAC
PALLAS_R        GAGCGGAGGCACGACAGCGCCCGCCTCCTCTGGCGGCGGCGCGGCCCCGCCGCGGCAGAC
BRB_F           GAGCGGAGGCACGACAGCGCCCGCCTCCTCTGGCGGCGGCGCGGCCCCGCCGCGGCAGAC
BRB_R           GAGCGGAGGCACGACAGCGCCCGCCTCCTCTGGCGGCGGCGCGGCCCCGCCGCGGCAGAC
                ************************************************************

PALLAS_F        GCGCGTGCGGGCGAGGCGAGGGCAGGCCACCGACCCGCACAGCATCGCCGAACGTGTACG
PALLAS_R        GCGCGTGCGGGCGAGGCGAGGGCAGGCCACCGACCCGCACAGCATCGCCGAACGTGTACG
BRB_F           GCGCGTGCGGGCGAGGCGAGGGCAGGCCACCGACCCGCACAGCATCGCCGAACGTGTACG
BRB_R           GCGCGTGCGGGCGAGGCGAGGGCAGGCCACCGACCCGCACAGCATCGCCGAACGTGTACG
                ************************************************************

PALLAS_F        CATGCCACTCCCACACTCTCACCAGTATTCCCCCTTCCCATGACCCAATTTTAAAAACAA
PALLAS_R        CATGCCACTCCCACACTCTCACCAGTATTCCCCCTTCCCATGACCCAATTTTAAAAACAA
BRB_F           CATGCCACTCCCACACTCTCACCAGTATTCCCCCTTCCCATGACCCAATTTTAAAAACAA
BRB_R           CATGCCACTCCCACACTCTCACCAGTATTCCCCCTTCCCATGACCCAATTTTAAAAACAA
                ************************************************************

PALLAS_F        AACCCCAACATGTTGGTGCACATCAACAACAACCCAAAGTCTTCCAATCCTCCAACGTAT
PALLAS_R        AACCCCAACATGTTGGTGCACATCAACAACAACCCAAAGTCTTCCAATCCTCCAACGTAT
BRB_F           AACCCCAACATGTTGGTGCACATCAACAACAACCCAAAGTCTTCCAATCCTCCAACGTAT
BRB_R           AACCCCAACATGTTGGTGCACATCAACAACAACCCAAAGTCTTCCAATCCTCCAACGTAT
                ************************************************************

PALLAS_F        ATATTCTCTGCAAATTTGATCGCTCCTCTTTTTTATAACAGTAGAAGCAGGAACCGTTCT
PALLAS_R        ATATTCTCTGCAAATTTGATCGCTCCTCTTTTTTATAACAGTAGAAGCAGGAACCGTTCT
BRB_F           ATATTCTCTGCAAATTTGATCGCTCCTCTTTTTTATAACAGTAGAAGCAGGAACCGTTCT
BRB_R           ATATTCTCTGCAAATTTGATCGCTCCTCTTTTTTATAACAGTAGAAGCAGGAACCGTTCT
                ************************************************************

PALLAS_F        GTACCTACGCTACGCTATGCTATGCTATGATATGCTATGCTTGTAATCAAACTGAAATTT
PALLAS_R        GTACCTACGCTACGCTATGCTATGCTATGATATGCTATGCTTGTAATCAAACTGAAATTT
BRB_F           GTACCTACGCTACGCTATGCTATGCTATGATATGCTATGCTTGTAATCAAACTGAAATTT
BRB_R           GTACCTACGCTACGCTATGCTATGCTATGATATGCTATGCTTGTAATCAAACTGAAATTT
                ************************************************************

PALLAS_F        GAACGACTTTTTACCCTGAGCAACTCGCGTGCATGCAGCTCCGGCGGGAGAGGATCGCGG
PALLAS_R        GAACGACTTTTTACCCTGAGCAACTCGCGTGCATGCAGCTCCGGCGGGAGAGGATCGCGG
BRB_F           GAACGACTTTTTACCCTGAGCAACTCGCGTGCATGCAGCTCCGGCGGGAGAGGATCGCGG
BRB_R           GAACGACTTTTTACCCTGAGCAACTCGCGTGCATGCAGCTCCGGCGGGAGAGGATCGCGG
                ************************************************************

PALLAS_F        AGCGGATGAAGTCGCTGCAGGAGCTGGTCCCCAACGCCAACAAGGTACGTAAAACAATCA
PALLAS_R        AGCGGATGAAGTCGCTGCAGGAGCTGGTCCCCAACGCCAACAAGGTACGTAAAACAATCA
BRB_F           AGCGGATGAAGTCGCTGCAGGAGCTGGTCCCCAACGCCAACAAGGTACGTAAAACAATCA
BRB_R           AGCGGATGAAGTCGCTGCAGGAGCTGGTCCCCAACGCCAACAAGGTACGTAAAACAATCA
                ************************************************************

PALLAS_F        CGTCCCCGACGAGAATCTCTCGGCGATCAAACCGTATCGTGTAGAAAATTCCTGCGTTTC
PALLAS_R        CGTCCCCGACGAGAATCTCTCGGCGATCAAACCGTATCGTGTAGAAAATTCCTGCGTTTC
BRB_F           CGTCCCCGACGAGAATCTCTCGGCGATCAAACCGTATCGTGTAGAAAATTCCTGCGTTTC
BRB_R           CGTCCCCGACGAGAATCTCTCGGCGATCAAACCGTATCGTGTAGAAAATTCCTGCGTTTC
                ************************************************************

PALLAS_F        CCATCGACCTGAAACTGAAAGCCAGGAATCTCTCCCTGAAAGCCAGGAATCTCTCCCGGC
PALLAS_R        CCATCGACCTGAAACTGAAAGCCAGGAATCTCTCCCTGAAAGCCAGGAATCTCTCCCGGC
BRB_F           CCATCGACCTGAAACTGAAAGCCAGGAATCTCTCCCTGAAAGCCAGGAATCTCTCCCGGC
BRB_R           CCATCGACCTGAAACTGAAAGCCAGGAATCTCTCCCTGAAAGCCAGGAATCTCTCCCGGC
                ************************************************************
```

PALLAS_F GCGTGGGGGTGGGGGCTCGCCTGTCTATGCTACTAGTGGGCTTGTATGGTAGCAGTGCAG
PALLAS_R GCGTGGGGGTGGGGGCTCGCCTGTCTATGCTACTAGTGGGCTTGTATGGTAGCAGTGCAG
BRB_F GCGTGGGGGTGGGGGCTCGCCTGTCTATGCTACTAGTGGGCTTGTATGGTAGCAGTGCAG
BRB_R GCGTGGGGGTGGGGGCTCGCCTGTCTATGCTACTAGTGGGCTTGTATGGTAGCAGTGCAG
****

PALLAS_F AAAAGGGCACGCCGGCCGGCCGGTTGGTGAGCGAGGGTGCGGCGTCGCTCCTGTCTAGCT
PALLAS_R AAAAGGGCACGCCGGCCGGCCGGTTGGTGAGCGAGGGTGCGGCGTCGCTCCTGTCTAGCT
BRB_F AAAAGGGCACGCCGGCCGGCCGGTTGGTGAGCGAGGGTGCGGCGTCGCTCCTGTCTAGCT
BRB_R AAAAGGGCACGCCGGCCGGCCGGTTGGTGAGCGAGGGTGCGGCGTCGCTCCTGTCTAGCT
****

PALLAS_F CACGGGTCACGCCATGATGTGATGTGATGTGATGTGACATGTGTGCCGTCGAAGCGTGCG
PALLAS_R CACGGGTCACGCCATGATGTGATGTGATGTGATGTGACATGTGTGCCGTCGAAGCGTGCG
BRB_F CACGGGTCACGCCATGATGTGATGTGATGTGATGTGACATGTGTGCCGTCGAAGCGTGCG
BRB_R CACGGGTCACGCCATGATGTGATGTGATGTGATGTGACATGTGTGCCGTCGAAGCGTGCG
****

PALLAS_F CAGCACAGGAGACGCAGCACGTGTCGAGCTCGTGCTGGAAATGGCTTGGACCGGAGAAGG
PALLAS_R CAGCACAGGAGACGCAGCACGTGTCGAGCTCGTGCTGGAAATGGCTTGGACCGGAGAAGG
BRB_F CAGCACAGGAGACGCAGCACGTGTCGAGCTCGTGCTGGAAATGGCTTGGACCGGAGAAGG
BRB_R CAGCACAGGAGACGCAGCACGTGTCGAGCTCGTGCTGGAAATGGCTTGGACCGGAGAAGG
****

PALLAS_F CCGCAGCCTTGGGTGGCTCTTGGGCTAGCTGAAGAATTCAGCTGATGCCGGGATGGCAAA
PALLAS_R CCGCAGCCTTGGGTGGCTCTTGGGCTAGCTGAAGAATTCAGCTGATGCCGGGATGGCAAA
BRB_F CCGCAGCCTTGGGTGGCTCTTGGGCTAGCTGAAGAATTCAGCTGATGCCGGGATGGCAAA
BRB_R CCGCAGCCTTGGGTGGCTCTTGGGCTAGCTGAAGAATTCAGCTGATGCCGGGATGGCAAA
****

PALLAS_F TGAGGCGTGCAGTGAATGGGCACATGGGCGCATTGGATGCCCTGTTTCGCTCGATCCGCC
PALLAS_R TGAGGCGTGCAGTGAATGGGCACATGGGCGCATTGGATGCCCTGTTTCGCTCGATCCGCC
BRB_F TGAGGCGTGCAGTGAATGGGCACATGGGCGCATTGGATGCCCTGTTTCGCTCGATCCGCC
BRB_R TGAGGCGTGCAGTGAATGGGCACATGGGCGCATTGGATGCCCTGTTTCGCTCGATCCGCC
****

PALLAS_F ATGCCCTTCTAGTCCGGTAGTCATAATTACAGGCCATTTGTCAATGCCCGGATATGTGAT
PALLAS_R ATGCCCTTCTAGTCCGGTAGTCATAATTACAGGCCATTTGTCAATGCCCGGATATGTGAT
BRB_F ATGCCCTTCTAGTCCGGTAGTCATAATTACAGGCCATTTGTCAATGCCCGGATATGTGAT
BRB_R ATGCCCTTCTAGTCCGGTAGTCATAATTACAGGCCATTTGTCAATGCCCGGATATGTGAT
****

PALLAS_F CGCGCTGAAATCGAGCCGTTTCGTGTAGTATGTAAAAATCTATGCGCCGTGGTGGTAGAA
PALLAS_R CGCGCTGAAATCGAGCCGTTTCGTGTAGTATGTAAAAATCTATGCGCCGTGGTGGTAGAA
BRB_F CGCGCTGAAATCGAGCCGTTTCGTGTAGTATGTAAAAATCTATGCGCCGTGGTGGTAGAA
BRB_R CGCGCTGAAATCGAGCCGTTTCGTGTAGTATGTAAAAATCTATGCGCCGTGGTGGTAGAA
****

PALLAS_F TACTCGTTAGAAGGGGCGGTTACTGATGAGATGGCCATGCCGTTGGATTGTTGATTCGCA
PALLAS_R TACTCGTTAGAAGGGGCGGTTACTGATGAGATGGCCATGCCGTTGGATTGTTGATTCGCA
BRB_F TACTCGTTAGAAGGGGCGGTTACTGATGAGATGGCCATGCCGTTGGATTGTTGATTCGCA
BRB_R TACTCGTTAGAAGGGGCGGTTACTGATGAGATGGCCATGCCGTTGGATTGTTGATTCGCA
****

PALLAS_F GACTGACAAGGCGTCGATGCTGGACGAGATCATCGACTACGTCAAGTTCCTGCAGCTCCA
PALLAS_R GACTGACAAGGCGTCGATGCTGGACGAGATCATCGACTACGTCAAGTTCCTGCAGCTCCA
BRB_F GACTGACAAGGCGTCGATGCTGGACGAGATCATCGACTACGTCAAGTTCCTGCAGCTCCA
BRB_R GACTGACAAGGCGTCGATGCTGGACGAGATCATCGACTACGTCAAGTTCCTGCAGCTCCA
****

PALLAS_F AGTCAAGGTATATATACAGAGGTGTTCGATGACAGAGGTTGCTCTGTGTTTCCCTGCTTC
PALLAS_R AGTCAAGGTATATATACAGAGGTGTTCGATGACAGAGGTTGCTCTGTGTTTCCCTGCTTC
BRB_F AGTCAAGGTATATATACAGAGGTGTTCGATGACAGAGGTTGCTCTGTGTTTCCCTGCTTC
BRB_R AGTCAAGGTATATATACAGAGGTGTTCGATGACAGAGGTTGCTCTGTGTTTCCCTGCTTC
****

PALLAS_F TGATTACGTAATACGACCTGTGGAGGTGACACCACGCTTTTGTTCTGTCAGGTTCTGAGC
PALLAS_R TGATTACGTAATACGACCTGTGGAGGTGACACCACGCTTTTGTTCTGTCAGGTTCTGAGC
BRB_F TGATTACGTAATACGACCTGTGGAGGTGACACCACGCTTTTGTTCTGTCAGGTTCTGAGC

```
BRB_R         TGATTACGTAATACGACCTGTGGAGGTGACACCACGCTTTTGTTCTGTCAGGTTCTGAGC
              ************************************************************

PALLAS_F      ATGAGCCGGCTGGGCGGGGCGGCCGGTATGGCGCCGCTGGTGGCCAGCATGTCCTCCGAG
PALLAS_R      ATGAGCCGGCTGGGCGGGGCGGCCGGTATGGCGCCGCTGGTGGCCAGCATGTCCTCCGAG
BRB_F         ATGAGCCGGCTGGGCGGGGCGGCCGGTATGGCGCCGCTGGTGGCCAGCATGTCCTCCGAG
BRB_R         ATGAGCCGGCTGGGCGGGGCGGCCGGTATGGCGCCGCTGGTGGCCAGCATGTCCTCCGAG
              ************************************************************

PALLAS_F      GTAGGGGTACTCAAAGCCAGCACCCGTTCGAACCTACGTAATAACTTAAGTTGCCGTTCA
PALLAS_R      GTAGGGGTACTCAAAGCCAGCACCCGTTCGAACCTACGTAATAACTTAAGTTGCCGTTCA
BRB_F         GTAGGGGTACTCAAAGCCAGCACCCGTTCGAACCTACGTAATAACTTAAGTTGCCGTTCA
BRB_R         GTAGGGGTACTCAAAGCCAGCACCCGTTCGAACCTACGTAATAACTTAAGTTGCCGTTCA
              ************************************************************

PALLAS_F      CTGTGTCCTGAGGCGCGTCGACGTGGAATGAAATGCAGGCGAACAGCAGCGCGAAGAGCA
PALLAS_R      CTGTGTCCTGAGGCGCGTCGACGTGGAATGAAATGCAGGCGAACAGCAGCGCGAAGAGCA
BRB_F         CTGTGTCCTGAGGCGCGTCGACGTGGAATGAAATGCAGGCGAACAGCAGCGCGAAGAGCA
BRB_R         CTGTGTCCTGAGGCGCGTCGACGTGGAATGAAATGCAGGCGAACAGCAGCGCGAAGAGCA
              ************************************************************

PALLAS_F      GCAACGGCGGCGGGAACAGCGCGGCGGCCGCGGCGGCCAAGGCGAACGGCGGAGGCGAGA
PALLAS_R      GCAACGGCGGCGGGAACAGCGCGGCGGCCGCGGCGGCCAAGGCGAACGGCGGAGGCGAGA
BRB_F         GCAACGGCGGCGGGAACAGCGCGGCGGCCGCGGCGGCCAAGGCGAACGGCGGAGGCGAGA
BRB_R         GCAACGGCGGCGGGAACAGCGCGGCGGCCGCGGCGGCCAAGGCGAACGGCGGAGGCGAGA
              ************************************************************

PALLAS_F      GCGGGGGCGGTGGAGGGGGCGGCGGGCTGCGGGTGACGGAGCAGCAGGTGGCCAAGATGA
PALLAS_R      GCGGGGGCGGTGGAGGGGGCGGCGGGCTGCGGGTGACGGAGCAGCAGGTGGCCAAGATGA
BRB_F         GCGGGGGCGGTGGAGGGGGCGGCGGGCTGCGGGTGACGGAGCAGCAGGTGGCCAAGATGA
BRB_R         GCGGGGGCGGTGGAGGGGGCGGCGGGCTGCGGGTGACGGAGCAGCAGGTGGCCAAGATGA
              ************************************************************

PALLAS_F      TGGAGGAGGACATGGGCACGGCCATGCAGTACCTGCAGGGGAAGGGCCTCTGCCTCATGC
PALLAS_R      TGGAGGAGGACATGGGCACGGCCATGCAGTACCTGCAGGGGAAGGGCCTCTGCCTCATGC
BRB_F         TGGAGGAGGACATGGGCACGGCCATGCAGTACCTGCAGGGGAAGGGCCTCTGCCTCATGC
BRB_R         TGGAGGAGGACATGGGCACGGCCATGCAGTACCTGCAGGGGAAGGGCCTCTGCCTCATGC
              ************************************************************

PALLAS_F      CCATCTCCCTCGCCTCCGCCATCTCCTCCGCCACCACCACCACCTCCCCGGCCTCGCTCC
PALLAS_R      CCATCTCCCTCGCCTCCGCCATCTCCTCCGCCACCACCACCACCTCCCCGGCCTCGCTCC
BRB_F         CCATCTCCCTCGCCTCCGCCATCTCCTCCGCCACCACCACCACCTCCCCGGCCTCGCTCC
BRB_R         CCATCTCCCTCGCCTCCGCCATCTCCTCCGCCACCACCACCACCTCCCCGGCCTCGCTCC
              ************************************************************

PALLAS_F      TCGCGAGGCAGGCCGTCCGCCCCGCCTCCACCGCCGCCGCGCTGGCCTCCGCGAACGGCG
PALLAS_R      TCGCGAGGCAGGCCGTCCGCCCCGCCTCCACCGCCGCCGCGCTGGCCTCCGCGAACGGCG
BRB_F         TCGCGAGGCAGGCCGTCCGCCCCGCCTCCACCGCCGCCGCGCTGGCCTCCGCGAACGGCG
BRB_R         TCGCGAGGCAGGCCGTCCGCCCCGCCTCCACCGCCGCCGCGCTGGCCTCCGCGAACGGCG
              ************************************************************

PALLAS_F      GCGAAGACGCCGCTGCCAGGCCCGTCAAGGTCGACGCCGGCGCCGCGTCCGGCGGGAAGC
PALLAS_R      GCGAAGACGCCGCTGCCAGGCCCGTCAAGGTCGACGCCGGCGCCGCGTCCGGCGGGAAGC
BRB_F         GCGAAGACGCCGCTGCCAGGCCCGTCAAGGTCGACGCCGGCGCCGCGTCCGGCGGGAAGC
BRB_R         GCGAAGACGCCGCTGCCAGGCCCGTCAAGGTCGACGCCGGCGCCGCGTCCGGCGGGAAGC
              ************************************************************

PALLAS_F      CGTGAAGCTGACCGGCCGGAACAAGGGTGCATGCATTATCTATGCTAATACTATTAGGAG
PALLAS_R      CGTGAAGCTGACCGGCCGGAACAAGGGTGCATGCATTATCTATGCTAATACTATTAGGAG
BRB_F         CGTGAAGCTGACCGGCCGGAACAAGGGTGCATGCATTATCTATGCTAATACTATTAGGAG
BRB_R         CGTGAAGCTGACCGGCCGGAACAAGGGTGCATGCATTATCTATGCTAATACTATTAGGAG
              ************************************************************

PALLAS_F      AGCCATATGTATTTCGCTCCTGTATACGCCTTCCCTTCCAGTTTTCTTCCTCTGAAAATC
PALLAS_R      AGCCATATGTATTTCGCTCCTGTATACGCCTTCCCTTCCAGTTTTCTTCCTCTGAAAATC
BRB_F         AGCCATATGTATTTCGCTCCTGTATACGCCTTCCCTTCCAGTTTTCTTCCTCTGAAAATC
BRB_R         AGCCATATGTATTTCGCTCCTGTATACGCCTTCCCTTCCAGTTTTCTTCCTCTGAAAATC
              ************************************************************
```

```
PALLAS_F      AGACTCGCCATGCACACGTACGTGCATGCGCATGATCGAGCGAGCTCCCATGAGAAGGCT
PALLAS_R      AGACTCGCCATGCACACGTACGTGCATGCGCATGATCGAGCGAGCTCCCATGAGAAGGCT
BRB_F         AGACTCGCCATGCACACGTACGTGCATGCGCATGATCGAGCGAGCTCCCATGAGAAGGCT
BRB_R         AGACTCGCCATGCACACGTACGTGCATGCGCATGATCGAGCGAGCTCCCATGAGAAGGCT
              ************************************************************

PALLAS_F      TACCACGACTTCCTCCATGAATTTGCATGAAGATATTCTCGTGTGCATGCGCAGCCACCT
PALLAS_R      TACCACGACTTCCTCCATGAATTTGCATGAAGATATTCTCGTGTGCATGCGCAGCCACCT
BRB_F         TACCACGACTTCCTCCATGAATTTGCATGAAGATATTCTCGTGTGCATGCGCAGCCACCT
BRB_R         TACCACGACTTCCTCCATGAATTTGCATGAAGATATTCTCGTGTGCATGCGCAGCCACCT
              ************************************************************

PALLAS_F      TTCGTGCGCTTCGACGTTGACAAAACACGGTGGTACTAATCTCACAAGGTCAGCCGTTGA
PALLAS_R      TTCGTGCGCTTCGACGTTGACAAAACACGGTGGTACTAATCTCACAAGGTCAGCCGTTGA
BRB_F         TTCGTGCGCTTCGACGTTGACAAAACACGGTGGTACTAATCTCACAAGGTCAGCCGTTGA
BRB_R         TTCGTGCGCTTCGACGTTGACAAAACACGGTGGTACTAATCTCACAAGGTCAGCCGTTGA
              ************************************************************

PALLAS_F      CACTGCTGCCTCGGCATATTCCTAATCGGATGCGAGCATTAATGATCCTGATGCTGCTGG
PALLAS_R      CACTGCTGCCTCGGCATATTCCTAATCGGATGCGAGCATTAATGATCCTGATGCTGCTGG
BRB_F         CACTGCTGCCTCGGCATATTCCTAATCGGATGCGAGCATTAATGATCCTGATGCTGCTGG
BRB_R         CACTGCTGCCTCGGCATATTCCTAATCGGATGCGAGCATTAATGATCCTGATGCTGCTGG
              ************************************************************

PALLAS_F      TGATTAGTTGCGAGTAAGATGGTGGCGAGATGTGCAGGCTACTAACGCCGCACGACGTTC
PALLAS_R      TGATTAGTTGCGAGTAAGATGGTGGCGAGATGTGCAGGCTACTAACGCCGCACGACGTTC
BRB_F         TGATTAGTTGCGAGTAAGATGGTGGCGAGATGTGCAGGCTACTAACGCCGCACGACGTTC
BRB_R         TGATTAGTTGCGAGTAAGATGGTGGCGAGATGTGCAGGCTACTAACGCCGCACGACGTTC
              ************************************************************

PALLAS_F      GTCTATGGGCTGGCTGAGGTAAACAAATGCGGCGGAATAATTAAGGGCGTGCATTGTTCG
PALLAS_R      GTCTATGGGCTGGCTGAGGTAAACAAATGCGGCGGAATAATTAAGGGCGTGCATTGTTCG
BRB_F         GTCTATGGGCTGGCTGAGGTAAACAAATGCGGCGGAATAATTAAGGGCGTGCATTGTTCG
BRB_R         GTCTATGGGCTGGCTGAGGTAAACAAATGCGGCGGAATAATTAAGGGCGTGCATTGTTCG
              ************************************************************

PALLAS_F      CACGACACGGAGCGAGACGAGGATTAAGGGGGTGGATTGAGAATCAATCCGGCGCGAGGT
PALLAS_R      CACGACACGGAGCGAGACGAGGATTAAGGGGGTGGATTGAGAATCAATCCGGCGCGAGGT
BRB_F         CACGACACGGAGCGAGACGAGGATTAAGGGGGTGGATTGAGAATCAATCCGGCGCGAGGT
BRB_R         CACGACACGGAGCGAGACGAGGATTAAGGGGGTGGATTGAGAATCAATCCGGCGCGAGGT
              ************************************************************

PALLAS_F      GCCCACGTTGGTGGTGAGGCGAACGAGTTTCTGCCAGCTGCGGTAGAGGATTTTTGGATC
PALLAS_R      GCCCACGTTGGTGGTGAGGCGAACGAGTTTCTGCCAGCTGCGGTAGAGGATTTTTGGATC
BRB_F         GCCCACGTTGGTGGTGAGGCGAACGAGTTTCTGCCAGCTGCGGTAGAGGATTTTTGGATC
BRB_R         GCCCACGTTGGTGGTGAGGCGAACGAGTTTCTGCCAGCTGCGGTAGAGGATTTTTGGATC
              ************************************************************

PALLAS_F      CAGTATACCACCACCATCACCACCACCGTCAGCACCACCACCACCACAACAACAACAATA
PALLAS_R      CAGTATACCACCACCATCACCACCACCGTCAGCACCACCACCACCACAACAACAACAATA
BRB_F         CAGTATACCACCACCATCACCACCACCGTCAGCACCACCACCACCACAACAACAACAATA
BRB_R         CAGTATACCACCACCATCACCACCACCGTCAGCACCACCACCACCACAACAACAACAATA
              ************************************************************

PALLAS_F      AAAGACCCATCCCAGGTTGTCAGTGTGACGGGAGGACGACCTAGGTCTTCATTGCGAAAG
PALLAS_R      AAAGACCCATCCCAGGTTGTCAGTGTGACGGGAGGACGACCTAGGTCTTCATTGCGAAAG
BRB_F         AAAGACCCATCCCAGGTTGTCAGTGTGACGGGAGGACGACCTAGGTCTTCATTGCGAAAG
BRB_R         AAAGACCCATCCCAGGTTGTCAGTGTGACGGGAGGACGACCTAGGTCTTCATTGCGAAAG
              ************************************************************

PALLAS_F      CCTCCCTTTTCTTTCTGCTTCGCCGACGCCTGATGAGGAAGCTCAATCGGCGTCGGCAAC
PALLAS_R      CCTCCCTTTTCTTTCTGCTTCGCCGACGCCTGATGAGGAAGCTCAATCGGCGTCGGCAAC
BRB_F         CCTCCCTTTTCTTTCTGCTTCGCCGACGCCTGATGAGGAAGCTCAATCGGCGTCGGCAAC
BRB_R         CCTCCCTTTTCTTTCTGCTTCGCCGACGCCTGATGAGGAAGCTCAATCGGCGTCGGCAAC
              ************************************************************

PALLAS_F      GGCAGGGTCATTAAAATCTCTCGTGCAGCAGGAGCGGCAGTGCGACGTCATGTGGTTGCA
PALLAS_R      GGCAGGGTCATTAAAATCTCTCGTGCAGCAGGAGCGGCAGTGCGACGTCATGTGGTTGCA
BRB_F         GGCAGGGTCATTAAAATCTCTCGTGCAGCAGGAGCGGCAGTGCGACGTCATGTGGTTGCA
```

```
BRB_R          GGCAGGGTCATTAAAATCTCTCGTGCAGCAGGAGCGGCAGTGCGACGTCATGTGGTTGCA
               ************************************************************

PALLAS_F       ACGACGACGCCCGTTGTCCTACCTCCTCACGACGGCGATTGTACCTGGCCTACGTTCGGT
PALLAS_R       ACGACGACGCCCGTTGTCCTACCTCCTCACGACGGCGATTGTACCTGGCCTACGTTCGGT
BRB_F          ACGACGACGCCCGTTGTCCTACCTCCTCACGACGGCGATTGTACCTGGCCTACGTTCGGT
BRB_R          ACGACGACGCCCGTTGTCCTACCTCCTCACGACGGCGATTGTACCTGGCCTACGTTCGGT
               ************************************************************

PALLAS_F       CAGTTTTGGGGCCGATGCGGGTTGTGGTGGTGGGGCGGGCTGGTGGTGGGGCGACCGTGG
PALLAS_R       CAGTTTTGGGGCCGATGCGGGTTGTGGTGGTGGGGCGGGCTGGTGGTGGGGCGACCGTGG
BRB_F          CAGTTTTGGGGCCGATGCGGGTTGTGGTGGTGGGGCGGGCTGGTGGTGGGGCGACCGTGG
BRB_R          CAGTTTTGGGGCCGATGCGGGTTGTGGTGGTGGGGCGGGCTGGTGGTGGGGCGACCGTGG
               ************************************************************

PALLAS_F       TCCAGCTGGCGGCATGATAATCCCTAAGTATAAGGAGAAGCACTCTCCTAGAAGGATATG
PALLAS_R       TCCAGCTGGCGGCATGATAATCCCTAAGTATAAGGAGAAGCACTCTCCTAGAAGGATATG
BRB_F          TCCAGCTGGCGGCATGATAATCCCTAAGTATAAGGAGAAGCACTCTCCTAGAAGGATATG
BRB_R          TCCAGCTGGCGGCATGATAATCCCTAAGTATAAGGAGAAGCACTCTCCTAGAAGGATATG
               ************************************************************

PALLAS_F       AAGGTATATGAAGTGTCAAACCAAAGGGAGCTAAAGATATTTTTCTTATTCTTTCAGGTT
PALLAS_R       AAGGTATATGAAGTGTCAAACCAAAGGGAGCTAAAGATATTTTTCTTATTCTTTCAGGTT
BRB_F          AAGGTATATGAAGTGTCAAACCAAAGGGAGCTAAAGATATTTTTCTTATTCTTTCAGGTT
BRB_R          AAGGTATATGAAGTGTCAAACCAAAGGGAGCTAAAGATATTTTTCTTATTCTTTCAGGTT
               ************************************************************

PALLAS_F       TCATATGCTACTTACGCAACTCTATATGTACTTAACATCTATCATACCTAAGCTCTCTAC
PALLAS_R       TCATATGCTACTTACGCAACTCTATATGTACTTAACATCTATCATACCTAAGCTCTCTAC
BRB_F          TCATATGCTACTTACGCAACTCTATATGTACTTAACATCTATCATACCTAAGCTCTCTAC
BRB_R          TCATATGCTACTTACGCAACTCTATATGTACTTAACATCTATCATACCTAAGCTCTCTAC
               ************************************************************

PALLAS_F       AACTAGGATATTATCTGGGAGTAGAAACTAAGGTACTCGCTAAAAAGGTTAGTGGATACA
PALLAS_R       AACTAGGATATTATCTGGGAGTAGAAACTAAGGTACTCGCTAAAAAGGTTAGTGGATACA
BRB_F          AACTAGGATATTATCTGGGAGTAGAAACTAAGGTACTCGCTAAAAAGGTTAGTGGATACA
BRB_R          AACTAGGATATTATCTGGGAGTAGAAACTAAGGTACTCGCTAAAAAGGTTAGTGGATACA
               ************************************************************

PALLAS_F       AAATAAAAAAAAAAATGCTAGTGGCTACAACTAAAAGATTTACAATGATCCTTCATACAG
PALLAS_R       AAATAAAAAAAAAAATGCTAGTGGCTACAACTAAAAGATTTACAATGATCCTTCATACAG
BRB_F          AAATAAAAAAAAAAATGCTAGTGGCTACAACTAAAAGATTTACAATGATCCTTCATACAG
BRB_R          AAATAAAAAAAAAAATGCTAGTGGCTACAACTAAAAGATTTACAATGATCCTTCATACAG
               ************************************************************

PALLAS_F       TCTGAAATTTATCTTATAAATTAAGTGATTTACTCTTCTCTCTCTCTCTCCCCACTCGCT
PALLAS_R       TCTGAAATTTATCTTATAAATTAAGTGATTTACTCTTCTCTCTCTCTCTCCCCACTCGCT
BRB_F          TCTGAAATTTATCTTATAAATTAAGTGATTTACTCTTCTCTCTCTCTCTCCCCACTCGCT
BRB_R          TCTGAAATTTATCTTATAAATTAAGTGATTTACTCTTCTCTCTCTCTCTCCCCACTCGCT
               ************************************************************

PALLAS_F       CGCTCACTCACTCACTCACTCCCCCCCTCTCCCTCTCCTTCCCTCTCTCCCTCTCTATCT
PALLAS_R       CGCTCACTCACTCACTCACTCCCCCCCTCTCCCTCTCCTTCCCTCTCTCCCTCTCTATCT
BRB_F          CGCTCACTCACTCACTCACTCCCCCCCTCTCCCTCTCCTTCCCTCTCTCCCTCTCTATCT
BRB_R          CGCTCACTCACTCACTCACTCCCCCCCTCTCCCTCTCCTTCCCTCTCTCCCTCTCTATCT
               ************************************************************

PALLAS_F       CTTCCCTCCCTCCCTCCCTTTCTCTATCTCTCTCAAATTGTTCATTGAAGTCAATTGTAT
PALLAS_R       CTTCCCTCCCTCCCTCCCTTTCTCTATCTCTCTCAAATTGTTCATTGAAGTCAATTGTAT
BRB_F          CTTCCCTCCCTCCCTCCCTTTCTCTATCTCTCTCAAATTGTTCATTGAAGTCAATTGTAT
BRB_R          CTTCCCTCCCTCCCTCCCTTTCTCTATCTCTCTCAAATTGTTCATTGAAGTCAATTGTAT
               ************************************************************

PALLAS_F       CTTTTGTGGTTTTATTTGTTGTATAAGGTTTGAGGTTTAGGGTTTAAATAGATTTGCATG
PALLAS_R       CTTTTGTGGTTTTATTTGTTGTATAAGGTTTGAGGTTTAGGGTTTAAATAGATTTGCATG
BRB_F          CTTTTGTGGTTTTATTTGTTGTATAAGGTTTGAGGTTTAGGGTTTAAATAGATTTGCATG
BRB_R          CTTTTGTGGTTTTATTTGTTGTATAAGGTTTGAGGTTTAGGGTTTAAATAGATTTGCATG
               ************************************************************
```

```
PALLAS_F        ATCTCCAATCTATATATGTCTTCTTTGGCCAAGTTTGATGGCTTTTTCTTCGGGGGGACT
PALLAS_R        ATCTCCAATCTATATATGTCTTCTTTGGCCAAGTTTGATGGCTTTTTCTTCGGGGGGACT
BRB_F           ATCTCCAATCTATATATGTCTTCTTTGGCCAAGTTTGATGGCTTTTTCTTCGGGGGGACT
BRB_R           ATCTCCAATCTATATATGTCTTCTTTGGCCAAGTTTGATGGCTTTTTCTTCGGGGGGACT
                ************************************************************

PALLAS_F        AGTGCTTTGCAGTTGGTTCGGTCTTGTGGTGATCCTAGTGACAA
PALLAS_R        AGTGCTTTGCAGTTGGTTCGGTCTTGTGGTGATCCTAGTGACAA
BRB_F           AGTGCTTTGCAGTTGGTTCGGTCTTGTGGTGATCCTAGTGACAA
BRB_R           AGTGCTTTGCAGTTGGTTCGGTCTTGTGGTGATCCTAGTGACAA
                ********************************************
```

Alignment_HORVU7Hr1G030270

```
Pallas_F      GCGAGGGAGTTGCATGATCTCATGGTCTTAGGAACAAATACTTGACATAAAGAAAGCAAT
Pallas_R      GCGAGGGAGTTGCATGATCTCATGGTCTTAGGAACAAATACTTGACATAAAGAAAGCAAT
Brb_F         GCGAGGGAGTTGCATGATCTCATGGTCTTAGGAACAAATACTTGACATAAAGAAAGCAAT
Brb_R         GCGAGGGAGTTGCATGATCTCATGGTCTTAGGAACAAATACTTGACATAAAGAAAGCAAT
              ************************************************************

Pallas_F      AGCAATAAACTTAATGATACGTTCAGATGGTATGCTTACGGTTGGATCTTGTCCATCACA
Pallas_R      AGCAATAAACTTAATGATACGTTCAGATGGTATGCTTACGGTTGGATCTTGTCCATCACA
Brb_F         AGCAATAAACTTAATGATACGTTCAGATGGTATGCTTACGGTTGGATCTTGTCCATCACA
Brb_R         AGCAATAAACTTAATGATACGTTCAGATGGTATGCTTACGGTTGGATCTTGTCCATCACA
              ************************************************************

Pallas_F      TCATTCTCCTAATGATGTGGTCTCGTTATCAAATGACAACTCATGTCTATGACTAGAAAA
Pallas_R      TCATTCTCCTAATGATGTGGTCTCGTTATCAAATGACAACTCATGTCTATGACTAGAAAA
Brb_F         TCATTCTCCTAATGATGTGGTCTCGTTATCAAATGACAACTCATGTCTATGACTAGAAAA
Brb_R         TCATTCTCCTAATGATGTGGTCTCGTTATCAAATGACAACTCATGTCTATGACTAGAAAA
              ************************************************************

Pallas_F      CCTTAACCATCTTTGATCAATGAGCTAGTCTAGTAAAGACTTAATAGGGACTTTGTGTTA
Pallas_R      CCTTAACCATCTTTGATCAATGAGCTAGTCTAGTAAAGACTTAATAGGGACTTTGTGTTA
Brb_F         CCTTAACCATCTTTGATCAATGAGCTAGTCTAGTAAAGACTTAATAGGGACTTTGTGTTA
Brb_R         CCTTAACCATCTTTGATCAATGAGCTAGTCTAGTAAAGACTTAATAGGGACTTTGTGTTA
              ************************************************************

Pallas_F      CGGAAATGCAATTCGGCTCACATTACGAGATGATGTTTTATTGTCATGGAAAATTTCAAA
Pallas_R      CGGAAATGCAATTCGGCTCACATTACGAGATGATGTTTTATTGTCATGGAAAATTTCAAA
Brb_F         CGGAAATGCAATTCGGCTCACATTACGAGATGATGTTTTATTGTCATGGAAAATTTCAAA
Brb_R         CGGAAATGCAATTCGGCTCACATTACGAGATGATGTTTTATTGTCATGGAAAATTTCAAA
              ************************************************************

Pallas_F      TTGAGACACATTACATGATGTGAGCTATGAAAAGAACAAAATCAGATTTGAATAGTTTTC
Pallas_R      TTGAGACACATTACATGATGTGAGCTATGAAAAGAACAAAATCAGATTTGAATAGTTTTC
Brb_F         TTGAGACACATTACATGATGTGAGCTATGAAAAGAACAAAATCAGATTTGAATAGTTTTC
Brb_R         TTGAGACACATTACATGATGTGAGCTATGAAAAGAACAAAATCAGATTTGAATAGTTTTC
              ************************************************************

Pallas_F      AAATACTAATGTCACTATCCAGGGTTGAAATTTGTCTTTTTCGTACTTCACATCATGTAA
Pallas_R      AAATACTAATGTCACTATCCAGGGTTGAAATTTGTCTTTTTCGTACTTCACATCATGTAA
Brb_F         AAATACTAATGTCACTATCCAGGGTTGAAATTTGTCTTTTTCGTACTTCACATCATGTAA
Brb_R         AAATACTAATGTCACTATCCAGGGTTGAAATTTGTCTTTTTCGTACTTCACATCATGTAA
              ************************************************************

Pallas_F      TGCGTTTTAACTTGAAATTTTATGTGGCAATATGATATCATCATCTCAACATTCTATTTT
Pallas_R      TGCGTTTTAACTTGAAATTTTATGTGGCAATATGATATCATCATCTCAACATTCTATTTT
Brb_F         TGCGTTTTAACTTGAAATTTTATGTGGCAATATGATATCATCATCTCAACATTCTATTTT
Brb_R         TGCGTTTTAACTTGAAATTTTATGTGGCAATATGATATCATCATCTCAACATTCTATTTT
              ************************************************************

Pallas_F      TTTCAGATTCTTGTAAAACTTTAAAATATAAGTTTTATGATGTTTTCATTGAATGTTGGT
Pallas_R      TTTCAGATTCTTGTAAAACTTTAAAATATAAGTTTTATGATGTTTTCATTGAATGTTGGT
Brb_F         TTTCAGATTCTTGTAAAACTTTAAAATATAAGTTTTATGATGTTTTCATTGAATGTTGGT
Brb_R         TTTCAGATTCTTGTAAAACTTTAAAATATAAGTTTTATGATGTTTTCATTGAATGTTGGT
              ************************************************************

Pallas_F      TTTCGTATGATACAAGTGCACCCCGTACTGTTGTTTGCAAAGTATTCCAGCGTAGTGAGC
Pallas_R      TTTCGTATGATACAAGTGCACCCCGTACTGTTGTTTGCAAAGTATTCCAGCGTAGTGAGC
Brb_F         TTTCGTATGATACAAGTGCACCCCGTACTGTTGTTTGCAAAGTATTCCAGCGTAGTGAGC
Brb_R         TTTCGTATGATACAAGTGCACCCCGTACTGTTGTTTGCAAAGTATTCCAGCGTAGTGAGC
              ************************************************************

Pallas_F      GGCCCAGATCAGCCCGAAGCCAGTCGCAAAGCTCCAGGCGAAACCTAGTCACTCGACTAC
Pallas_R      GGCCCAGATCAGCCCGAAGCCAGTCGCAAAGCTCCAGGCGAAACCTAGTCACTCGACTAC
Brb_F         GGCCCAGATCAGCCCGAAGCCAGTCGCAAAGCTCCAGGCGAAACCTAGTCACTCGACTAC
Brb_R         GGCCCAGATCAGCCCGAAGCCAGTCGCAAAGCTCCAGGCGAAACCTAGTCACTCGACTAC
              ************************************************************
```

```
Pallas_F      TCCCAACCGGCCGCCGCCGTCTCCCGTCCACGTTCCGACCGCCGGAGCTCGCCTCCTCCT
Pallas_R      TCCCAACCGGCCGCCGCCGTCTCCCGTCCACGTTCCGACCGCCGGAGCTCGCCTCCTCCT
Brb_F         TCCCAACCGGCCGCCGCCGTCTCCCGTCCACGTTCCGACCGCCGGAGCTCGCCTCCTCCT
Brb_R         TCCCAACCGGCCGCCGCCGTCTCCCGTCCACGTTCCGACCGCCGGAGCTCGCCTCCTCCT
              ************************************************************

Pallas_F      CCACCCGCTACCTCTCGAGGCCACTCCGGACGGCCATGGCCCAGAGGGCCGCGGGCGGCC
Pallas_R      CCACCCGCTACCTCTCGAGGCCACTCCGGACGGCCATGGCCCAGAGGGCCGCGGGCGGCC
Brb_F         CCACCCGCTACCTCTCGAGGCCACTCCGGACGGCCATGGCCCAGAGGGCCGCGGGCGGCC
Brb_R         CCACCCGCTACCTCTCGAGGCCACTCCGGACGGCCATGGCCCAGAGGGCCGCGGGCGGCC
              ************************************************************

Pallas_F      TTCTCCGACGGTCCCTCGGGCTCGCGCCGCCGACAACCCCGAGGGCCCTGAGCACCAGCG
Pallas_R      TTCTCCGACGGTCCCTCGGGCTCGCGCCGCCGACAACCCCGAGGGCCCTGAGCACCAGCG
Brb_F         TTCTCCGACGGTCCCTCGGGCTCGCGCCGCCGACAACCCCGAGGGCCCTGAGCACCAGCG
Brb_R         TTCTCCGACGGTCCCTCGGGCTCGCGCCGCCGACAACCCCGAGGGCCCTGAGCACCAGCG
              ************************************************************

Pallas_F      CCACCACGCCGGCGGAGGGAGAGGCGGCGGCCAAGGCGAGGAGGAGAAAGAAGAAGAACC
Pallas_R      CCACCACGCCGGCGGAGGGAGAGGCGGCGGCCAAGGCGAGGAGGAGAAAGAAGAAGAACC
Brb_F         CCACCACGCCGGCGGAGGGAGAGGCGGCGGCCAAGGCGAGGAGGAGAAAGAAGAAGAACC
Brb_R         CCACCACGCCGGCGGAGGGAGAGGCGGCGGCCAAGGCGAGGAGGAGAAAGAAGAAGAACC
              ************************************************************

Pallas_F      TGTTCGACGTGGCGCAGTTCCTGCCGGACTGGGGCGTCGGCCACAGGGTCGCCAAGACCA
Pallas_R      TGTTCGACGTGGCGCAGTTCCTGCCGGACTGGGGCGTCGGCCACAGGGTCGCCAAGACCA
Brb_F         TGTTCGACGTGGCGCAGTTCCTGCCGGACTGGGGCGTCGGCCACAGGGTCGCCAAGACCA
Brb_R         TGTTCGACGTGGCGCAGTTCCTGCCGGACTGGGGCGTCGGCCACAGGGTCGCCAAGACCA
              ************************************************************

Pallas_F      CCTGGCGCGACGTCTCCTACCAGATCACCAAGATCAACCTCTATAAGGTCGCCATCGCCA
Pallas_R      CCTGGCGCGACGTCTCCTACCAGATCACCAAGATCAACCTCTATAAGGTCGCCATCGCCA
Brb_F         CCTGGCGCGACGTCTCCTACCAGATCACCAAGATCAACCTCTATAAGGTCGCCATCGCCA
Brb_R         CCTGGCGCGACGTCTCCTACCAGATCACCAAGATCAACCTCTATAAGGTCGCCATCGCCA
              ************************************************************

Pallas_F      CCATCCATAACAATCTCTGCGGAGGAGAAAATTGATCTCTTGTGTTCAATTCCATGACTT
Pallas_R      CCATCCATAACAATCTCTGCGGAGGAGAAAATTGATCTCTTGTGTTCAATTCCATGACTT
Brb_F         CCATCCATAACAATCTCTGCGGAGGAGAAAATTGATCTCTTGTGTTCAATTCCATGACTT
Brb_R         CCATCCATAACAATCTCTGCGGAGGAGAAAATTGATCTCTTGTGTTCAATTCCATGACTT
              ************************************************************

Pallas_F      CACTGCGGTTTCCTGATCTCTGTATGTACTGATGCAGGATGGCCGCCACGGGAAGGCGTG
Pallas_R      CACTGCGGTTTCCTGATCTCTGTATGTACTGATGCAGGATGGCCGCCACGGGAAGGCGTG
Brb_F         CACTGCGGTTTCCTGATCTCTGTATGTACTGATGCAGGATGGCCGCCACGGGAAGGCGTG
Brb_R         CACTGCGGTTTCCTGATCTCTGTATGTACTGATGCAGGATGGCCGCCACGGGAAGGCGTG
              ************************************************************

Pallas_F      GGGAATTCGGCACAAGGCCGGTGAGTCCTCCTGAATTACCGTTAATTTTGGTGGATTGGT
Pallas_R      GGGAATTCGGCACAAGGCCGGTGAGTCCTCCTGAATTACCGTTAATTTTGGTGGATTGGT
Brb_F         GGGAATTCGGCACAAGGCCGGTGAGTCCTCCTGAATTACCGTTAATTTTGGTGGATTGGT
Brb_R         GGGAATTCGGCACAAGGCCGGTGAGTCCTCCTGAATTACCGTTAATTTTGGTGGATTGGT
              ************************************************************

Pallas_F      GGAGCGTAAAATAAATGACAGGGTGTGTGTCGAATGGTAGAACATGAATGCCCCCTGGAT
Pallas_R      GGAGCGTAAAATAAATGACAGGGTGTGTGTCGAATGGTAGAACATGAATGCCCCCTGGAT
Brb_F         GGAGCGTAAAATAAATGACAGGGTGTGTGTCGAATGGTAGAACATGAATGCCCCCTGGAT
Brb_R         GGAGCGTAAAATAAATGACAGGGTGTGTGTCGAATGGTAGAACATGAATGCCCCCTGGAT
              ************************************************************

Pallas_F      GGAGGACTACTTCTGCTTGACATCATCCTATGCAGTTGGGTGCACCAGGCGATGTTTCCG
Pallas_R      GGAGGACTACTTCTGCTTGACATCATCCTATGCAGTTGGGTGCACCAGGCGATGTTTCCG
Brb_F         GGAGGACTACTTCTGCTTGACATCATCCTATGCAGTTGGGTGCACCAGGCGATGTTTCCG
Brb_R         GGAGGACTACTTCTGCTTGACATCATCCTATGCAGTTGGGTGCACCAGGCGATGTTTCCG
              ************************************************************

Pallas_F      TTTTGGTTGCAGATTGTTGCTCTAGGATTAATTTGGTTGTCCTAGAATCTGTCTCATCTC
Pallas_R      TTTTGGTTGCAGATTGTTGCTCTAGGATTAATTTGGTTGTCCTAGAATCTGTCTCATCTC
Brb_F         TTTTGGTTGCAGATTGTTGCTCTAGGATTAATTTGGTTGTCCTAGAATCTGTCTCATCTC
```

```
Brb_R           TTTTGGTTGCAGATTGTTGCTCTAGGATTAATTTGGTTGTCCTAGAATCTGTCTCATCTC
                ************************************************************

Pallas_F        GATAACATTGGGGTTTAGGGTAAGGTGTAACAGCTCAAATACTTCATGTGGATATGCGGT
Pallas_R        GATAACATTGGGGTTTAGGGTAAGGTGTAACAGCTCAAATACTTCATGTGGATATGCGGT
Brb_F           GATAACATTGGGGTTTAGGGTAAGGTGTAACAGCTCAAATACTTCATGTGGATATGCGGT
Brb_R           GATAACATTGGGGTTTAGGGTAAGGTGTAACAGCTCAAATACTTCATGTGGATATGCGGT
                ************************************************************

Pallas_F        GTAACCAAAAAATTTCTAGGACATTGCGCTGTCTTTATCCCAGAAAGAAAAGATAAAAGA
Pallas_R        GTAACCAAAAAATTTCTAGGACATTGCGCTGTCTTTATCCCAGAAAGAAAAGATAAAAGA
Brb_F           GTAACCAAAAAATTTCTAGGACATTGCGCTGTCTTTATCCCAGAAAGAAAAGATAAAAGA
Brb_R           GTAACCAAAAAATTTCTAGGACATTGCGCTGTCTTTATCCCAGAAAGAAAAGATAAAAGA
                ************************************************************

Pallas_F        AGCACATCACACGTAACAGTTATTTACCAGGTTGTGAAGGATGTATCCATTGCATCACTA
Pallas_R        AGCACATCACACGTAACAGTTATTTACCAGGTTGTGAAGGATGTATCCATTGCATCACTA
Brb_F           AGCACATCACACGTAACAGTTATTTACCAGGTTGTGAAGGATGTATCCATTGCATCACTA
Brb_R           AGCACATCACACGTAACAGTTATTTACCAGGTTGTGAAGGATGTATCCATTGCATCACTA
                ************************************************************

Pallas_F        GGCCACACTAGAATCATTCAGTTGTTTGCTTCCCAATTACCCCAAGTTATTTTTATTTTA
Pallas_R        GGCCACACTAGAATCATTCAGTTGTTTGCTTCCCAATTACCCCAAGTTATTTTTATTTTA
Brb_F           GGCCACACTAGAATCATTCAGTTGTTTGCTTCCCAATTACCCCAAGTTATTTTTATTTTA
Brb_R           GGCCACACTAGAATCATTCAGTTGTTTGCTTCCCAATTACCCCAAGTTATTTTTATTTTA
                ************************************************************

Pallas_F        TTTTATTTTTATGTCTGGACCAAGATAGATCATATTTTCCTTGCAAGGTAGATCATGTTT
Pallas_R        TTTTATTTTTATGTCTGGACCAAGATAGATCATATTTTCCTTGCAAGGTAGATCATGTTT
Brb_F           TTTTATTTTTATGTCTGGACCAAGATAGATCATATTTTCCTTGCAAGGTAGATCATGTTT
Brb_R           TTTTATTTTTATGTCTGGACCAAGATAGATCATATTTTCCTTGCAAGGTAGATCATGTTT
                ************************************************************

Pallas_F        TCCCTGCCGTTTGCAAAAGTCATGGCATGGTTACTGTTGTTGGTCTTCAACAAAACCGTT
Pallas_R        TCCCTGCCGTTTGCAAAAGTCATGGCATGGTTACTGTTGTTGGTCTTCAACAAAACCGTT
Brb_F           TCCCTGCCGTTTGCAAAAGTCATGGCATGGTTACTGTTGTTGGTCTTCAACAAAACCGTT
Brb_R           TCCCTGCCGTTTGCAAAAGTCATGGCATGGTTACTGTTGTTGGTCTTCAACAAAACCGTT
                ************************************************************

Pallas_F        AGGACGCTTAATGTAGAAGAAACTTGGCACAACCAAGAATTACTGTGTCAGAAGGCAATA
Pallas_R        AGGACGCTTAATGTAGAAGAAACTTGGCACAACCAAGAATTACTGTGTCAGAAGGCAATA
Brb_F           AGGACGCTTAATGTAGAAGAAACTTGGCACAACCAAGAATTACTGTGTCAGAAGGCAATA
Brb_R           AGGACGCTTAATGTAGAAGAAACTTGGCACAACCAAGAATTACTGTGTCAGAAGGCAATA
                ************************************************************

Pallas_F        CTCTGTTCTCAAATACTCTACCTGAAGTAGAACATGAAATACCTGAAGTAGGTGACGGGC
Pallas_R        CTCTGTTCTCAAATACTCTACCTGAAGTAGAACATGAAATACCTGAAGTAGGTGACGGGC
Brb_F           CTCTGTTCTCAAATACTCTACCTGAAGTAGAACATGAAATACCTGAAGTAGGTGACGGGC
Brb_R           CTCTGTTCTCAAATACTCTACCTGAAGTAGAACATGAAATACCTGAAGTAGGTGACGGGC
                ************************************************************

Pallas_F        TTCAGGATTATGCTCGCCGATTCCTCATTTTGGTTGATCTAACTTTATGTTTCTCTTGCA
Pallas_R        TTCAGGATTATGCTCGCCGATTCCTCATTTTGGTTGATCTAACTTTATGTTTCTCTTGCA
Brb_F           TTCAGGATTATGCTCGCCGATTCCTCATTTTGGTTGATCTAACTTTATGTTTCTCTTGCA
Brb_R           TTCAGGATTATGCTCGCCGATTCCTCATTTTGGTTGATCTAACTTTATGTTTCTCTTGCA
                ************************************************************

Pallas_F        GGCGTGCCAGCAGCAGATGCTCCGATAAGAATCAGCGGGGTTAACAAACGTGGTTGGAAG
Pallas_R        GGCGTGCCAGCAGCAGATGCTCCGATAAGAATCAGCGGGGTTAACAAACGTGGTTGGAAG
Brb_F           GGCGTGCCAGCAGCAGATGCTCCGATAAGAATCAGCGGGGTTAACAAACGTGGTTGGAAG
Brb_R           GGCGTGCCAGCAGCAGATGCTCCGATAAGAATCAGCGGGGTTAACAAACGTGGTTGGAAG
                ************************************************************

Pallas_F        TACATAAAGGCGTCTTTGCAAGATATCCCTGGAGCAGAGCCGCCAGCCGTCTCCGCTGCT
Pallas_R        TACATAAAGGCGTCTTTGCAAGATATCCCTGGAGCAGAGCCGCCAGCCGTCTCCGCTGCT
Brb_F           TACATAAAGGCGTCTTTGCAAGATATCCCTGGAGCAGAGCCGCCAGCCGTCTCCGCTGCT
Brb_R           TACATAAAGGCGTCTTTGCAAGATATCCCTGGAGCAGAGCCGCCAGCCGTCTCCGCTGCT
                ************************************************************
```

```
Pallas_F      TAACCAGTTACTGGCTAGATATTTCGTAGGTGATAAAACTGGTCCCAATAGAAGCGTAGT
Pallas_R      TAACCAGTTACTGGCTAGATATTTCGTAGGTGATAAAACTGGTCCCAATAGAAGCGTAGT
Brb_F         TAACCAGTTACTGGCTAGATATTTCGTAGGTGATAAAACTGGTCCCAATAGAAGCGTAGT
Brb_R         TAACCAGTTACTGGCTAGATATTTCGTAGGTGATAAAACTGGTCCCAATAGAAGCGTAGT
              ************************************************************

Pallas_F      GCTGAACTATTCACAAGACTGGTTTGATCTGCCAGCTCCACGCGTTTCATAAGTCTCATG
Pallas_R      GCTGAACTATTCACAAGACTGGTTTGATCTGCCAGCTCCACGCGTTTCATAAGTCTCATG
Brb_F         GCTGAACTATTCACAAGACTGGTTTGATCTGCCAGCTCCACGCGTTTCATAAGTCTCATG
Brb_R         GCTGAACTATTCACAAGACTGGTTTGATCTGCCAGCTCCACGCGTTTCATAAGTCTCATG
              ************************************************************

Pallas_F      TCTCCACAAGGGGTTTAAACATGCTTGAACGTGTTTCGGCGTGAGCTTGTCATGGCCTCC
Pallas_R      TCTCCACAAGGGGTTTAAACATGCTTGAACGTGTTTCGGCGTGAGCTTGTCATGGCCTCC
Brb_F         TCTCCACAAGGGGTTTAAACATGCTTGAACGTGTTTCGGCGTGAGCTTGTCATGGCCTCC
Brb_R         TCTCCACAAGGGGTTTAAACATGCTTGAACGTGTTTCGGCGTGAGCTTGTCATGGCCTCC
              ************************************************************

Pallas_F      ACGCTTGTTGTTGAAGCTAAAGATGATGTAGTGTTAATTTTGTTTTGTTTTAGATTTTAT
Pallas_R      ACGCTTGTTGTTGAAGCTAAAGATGATGTAGTGTTAATTTTGTTTTGTTTTAGATTTTAT
Brb_F         ACGCTTGTTGTTGAAGCTAAAGATGATGTAGTGTTAATTTTGTTTTGTTTTAGATTTTAT
Brb_R         ACGCTTGTTGTTGAAGCTAAAGATGATGTAGTGTTAATTTTGTTTTGTTTTAGATTTTAT
              ************************************************************

Pallas_F      TTTTTGCAATGCCTCATTTCCATCAGAAGCAAGTGCTTCACCAACAATGACGAATCTCCA
Pallas_R      TTTTTGCAATGCCTCATTTCCATCAGAAGCAAGTGCTTCACCAACAATGACGAATCTCCA
Brb_F         TTTTTGCAATGCCTCATTTCCATCAGAAGCAAGTGCTTCACCAACAATGACGAATCTCCA
Brb_R         TTTTTGCAATGCCTCATTTCCATCAGAAGCAAGTGCTTCACCAACAATGACGAATCTCCA
              ************************************************************

Pallas_F      TCGACGTCGAGGACATGGCATTCCTGCAATGCCGCAGCTTGGCTCGGAGGCGATCGAGTG
Pallas_R      TCGACGTCGAGGACATGGCATTCCTGCAATGCCGCAGCTTGGCTCGGAGGCGATCGAGTG
Brb_F         TCGACGTCGAGGACATGGCATTCCTGCAATGCCGCAGCTTGGCTCGGAGGCGATCGAGTG
Brb_R         TCGACGTCGAGGACATGGCATTCCTGCAATGCCGCAGCTTGGCTCGGAGGCGATCGAGTG
              ************************************************************

Pallas_F      TCAAGTATGACAATTAGGTTAGACTCTAGCAAAGCAAGTTATGGTTTCCATTGTCTTTTT
Pallas_R      TCAAGTATGACAATTAGGTTAGACTCTAGCAAAGCAAGTTATGGTTTCCATTGTCTTTTT
Brb_F         TCAAGTATGACAATTAGGTTAGACTCTAGCAAAGCAAGTTATGGTTTCCATTGTCTTTTT
Brb_R         TCAAGTATGACAATTAGGTTAGACTCTAGCAAAGCAAGTTATGGTTTCCATTGTCTTTTT
              ************************************************************

Pallas_F      TCTTTTATCACTGACAGTTCATGTGAGTTCTGGCAAGGAATTCATCGCTCTGGAAAACGT
Pallas_R      TCTTTTATCACTGACAGTTCATGTGAGTTCTGGCAAGGAATTCATCGCTCTGGAAAACGT
Brb_F         TCTTTTATCACTGACAGTTCATGTGAGTTCTGGCAAGGAATTCATCGCTCTGGAAAACGT
Brb_R         TCTTTTATCACTGACAGTTCATGTGAGTTCTGGCAAGGAATTCATCGCTCTGGAAAACGT
              ************************************************************

Pallas_F      TGTTATAACTCTGAGAATTCAGTCAAGGCCTGAACCTGTGAACCATCTATAACTCTGAAC
Pallas_R      TGTTATAACTCTGAGAATTCAGTCAAGGCCTGAACCTGTGAACCATCTATAACTCTGAAC
Brb_F         TGTTATAACTCTGAGAATTCAGTCAAGGCCTGAACCTGTGAACCATCTATAACTCTGAAC
Brb_R         TGTTATAACTCTGAGAATTCAGTCAAGGCCTGAACCTGTGAACCATCTATAACTCTGAAC
              ************************************************************

Pallas_F      CTCGCTTGGTATAAACAACGTCGATGACCAGCGAATGGTGAACGCCAGCACAAATCACAA
Pallas_R      CTCGCTTGGTATAAACAACGTCGATGACCAGCGAATGGTGAACGCCAGCACAAATCACAA
Brb_F         CTCGCTTGGTATAAACAACGTCGATGACCAGCGAATGGTGAACGCCAGCACAAATCACAA
Brb_R         CTCGCTTGGTATAAACAACGTCGATGACCAGCGAATGGTGAACGCCAGCACAAATCACAA
              ************************************************************

Pallas_F      TGCGGCATAGATTAACCGATCAAGAGAAGACGCTGGTCCTGTCCGTTTTCCATTGCGAGG
Pallas_R      TGCGGCATAGATTAACCGATCAAGAGAAGACGCTGGTCCTGTCCGTTTTCCATTGCGAGG
Brb_F         TGCGGCATAGATTAACCGATCAAGAGAAGACGCTGGTCCTGTCCGTTTTCCATTGCGAGG
Brb_R         TGCGGCATAGATTAACCGATCAAGAGAAGACGCTGGTCCTGTCCGTTTTCCATTGCGAGG
              ************************************************************

Pallas_F      GGTTACTTATACATTCTGAAGCCCATTTCCGAGCACGTGAAATCTGCCTGTGTTCGGACA
Pallas_R      GGTTACTTATACATTCTGAAGCCCATTTCCGAGCACGTGAAATCTGCCTGTGTTCGGACA
Brb_F         GGTTACTTATACATTCTGAAGCCCATTTCCGAGCACGTGAAATCTGCCTGTGTTCGGACA
```

```
Brb_R        GGTTACTTATACATTCTGAAGCCCATTTCCGAGCACGTGAAATCTGCCTGTGTTCGGACA
             ************************************************************

Pallas_F     CGCTCTGCCTGCCTGCCTACCGCGGCACCGGACTGGGAGCAGGGCAGAGCGGCTACAGCT
Pallas_R     CGCTCTGCCTGCCTGCCTACCGCGGCACCGGACTGGGAGCAGGGCAGAGCGGCTACAGCT
Brb_F        CGCTCTGCCTGCCTGCCTACCGCGGCACCGGACTGGGAGCAGGGCAGAGCGGCTACAGCT
Brb_R        CGCTCTGCCTGCCTGCCTACCGCGGCACCGGACTGGGAGCAGGGCAGAGCGGCTACAGCT
             ************************************************************

Pallas_F     TCCCTTCTATTCTTTCTTTCACCTCTGCCACTCTGTCTGTTTCTTTTAACGGCCCGTCGA
Pallas_R     TCCCTTCTATTCTTTCTTTCACCTCTGCCACTCTGTCTGTTTCTTTTAACGGCCCGTCGA
Brb_F        TCCCTTCTATTCTTTCTTTCACCTCTGCCACTCTGTCTGTTTCTTTTAACGGCCCGTCGA
Brb_R        TCCCTTCTATTCTTTCTTTCACCTCTGCCACTCTGTCTGTTTCTTTTAACGGCCCGTCGA
             ************************************************************

Pallas_F     TCTGCTCTTTTGCTGCCCATGCAAACACAGGGACATGCCACTGTAGCACGGCGTGTTTGC
Pallas_R     TCTGCTCTTTTGCTGCCCATGCAAACACAGGGACATGCCACTGTAGCACGGCGTGTTTGC
Brb_F        TCTGCTCTTTTGCTGCCCATGCAAACACAGGGACATGCCACTGTAGCACGGCGTGTTTGC
Brb_R        TCTGCTCTTTTGCTGCCCATGCAAACACAGGGACATGCCACTGTAGCACGGCGTGTTTGC
             ************************************************************

Pallas_F     TGCCAATCTGCTGCGCCGCAGTCCCCCCAGTAA
Pallas_R     TGCCAATCTGCTGCGCCGCAGTCCCCCCAGTAA
Brb_F        TGCCAATCTGCTGCGCCGCAGTCCCCCCAGTAA
Brb_R        TGCCAATCTGCTGCGCCGCAGTCCCCCCAGTAA
             *********************************
```

Alignment_HORVU7Hr1G030290

```
Pallas_F      GGTGCCGATCTGGTCCGTCCGATCCCCGATCCGACGGCTGAGGAGACATAGTCCCAGGCC
Pallas_R      GGTGCCGATCTGGTCCGTCCGATCCCCGATCCGACGGCTGAGGAGACATAGTCCCAGGCC
Brb_F         GGTGCCGATCTGGTCCGTCCGATCCCCGATCCGACGGCTGAGGAGACATAGTCCCAGGCC
Brb_R         GGTGCCGATCTGGTCCGTCCGATCCCCGATCCGACGGCTGAGGAGACATAGTCCCAGGCC
              ************************************************************

Pallas_F      CCAAAAACGCTCCACCTATACATGCATCCCCGCCTGACACCTTCCATTTCCATTTCCAAG
Pallas_R      CCAAAAACGCTCCACCTATACATGCATCCCCGCCTGACACCTTCCATTTCCATTTCCAAG
Brb_F         CCAAAAACGCTCCACCTATACATGCATCCCCGCCTGACACCTTCCATTTCCATTTCCAAG
Brb_R         CCAAAAACGCTCCACCTATACATGCATCCCCGCCTGACACCTTCCATTTCCATTTCCAAG
              ************************************************************

Pallas_F      AAGCTGCGAGCCTGCAGAGCTGCACGCCATGGCAGCACTGCACTGCACTGCCCTGCCATG
Pallas_R      AAGCTGCGAGCCTGCAGAGCTGCACGCCATGGCAGCACTGCACTGCACTGCCCTGCCATG
Brb_F         AAGCTGCGAGCCTGCAGAGCTGCACGCCATGGCAGCACTGCACTGCACTGCCCTGCCATG
Brb_R         AAGCTGCGAGCCTGCAGAGCTGCACGCCATGGCAGCACTGCACTGCACTGCCCTGCCATG
              ************************************************************

Pallas_F      GTAGTACTCCCACTATTCCCTCCCGCAGAGCTGCGCCGAAAATTCCCTTCTTCCCATTCA
Pallas_R      GTAGTACTCCCACTATTCCCTCCCGCAGAGCTGCGCCGAAAATTCCCTTCTTCCCATTCA
Brb_F         GTAGTACTCCCACTATTCCCTCCCGCAGAGCTGCGCCGAAAATTCCCTTCTTCCCATTCA
Brb_R         GTAGTACTCCCACTATTCCCTCCCGCAGAGCTGCGCCGAAAATTCCCTTCTTCCCATTCA
              ************************************************************

Pallas_F      GCACAGGCGCAGGCGCAGCAGCTGTCACGCCGGCCTGCTCTGCGCGGCGCACACGCGCGC
Pallas_R      GCACAGGCGCAGGCGCAGCAGCTGTCACGCCGGCCTGCTCTGCGCGGCGCACACGCGCGC
Brb_F         GCACAGGCGCAGGCGCAGCAGCTGTCACGCCGGCCTGCTCTGCGCGGCGCACACGCGCGC
Brb_R         GCACAGGCGCAGGCGCAGCAGCTGTCACGCCGGCCTGCTCTGCGCGGCGCACACGCGCGC
              ************************************************************

Pallas_F      GCGCTCTGGCGCGAGCTGCACGGCGCGTCAGTAACTCGCAGAATCACAGCTTCCATTTCA
Pallas_R      GCGCTCTGGCGCGAGCTGCACGGCGCGTCAGTAACTCGCAGAATCACAGCTTCCATTTCA
Brb_F         GCGCTCTGGCGCGAGCTGCACGGCGCGTCAGTAACTCGCAGAATCACAGCTTCCATTTCA
Brb_R         GCGCTCTGGCGCGAGCTGCACGGCGCGTCAGTAACTCGCAGAATCACAGCTTCCATTTCA
              ************************************************************

Pallas_F      AAAACAAAAGGTAAAAATGGAAGCCCGCCACAGTCGCCCGGCGTCAGTGCCCCGCTCGTA
Pallas_R      AAAACAAAAGGTAAAAATGGAAGCCCGCCACAGTCGCCCGGCGTCAGTGCCCCGCTCGTA
Brb_F         AAAACAAAAGGTAAAAATGGAAGCCCGCCACAGTCGCCCGGCGTCAGTGCCCCGCTCGTA
Brb_R         AAAACAAAAGGTAAAAATGGAAGCCCGCCACAGTCGCCCGGCGTCAGTGCCCCGCTCGTA
              ************************************************************

Pallas_F      CCGCCTCCGCCGCCTTCACTCCGGCGCATGGGTTGCCCGCCCCCGCACTTAATTCCCGGC
Pallas_R      CCGCCTCCGCCGCCTTCACTCCGGCGCATGGGTTGCCCGCCCCCGCACTTAATTCCCGGC
Brb_F         CCGCCTCCGCCGCCTTCACTCCGGCGCATGGGTTGCCCGCCCCCGCACTTAATTCCCGGC
Brb_R         CCGCCTCCGCCGCCTTCACTCCGGCGCATGGGTTGCCCGCCCCCGCACTTAATTCCCGGC
              ************************************************************

Pallas_F      CGGCCTGCGCTTTCAGTTAAGGTACCTCCACGTACCGCAGTACGGGCCCGGGTCCATCGA
Pallas_R      CGGCCTGCGCTTTCAGTTAAGGTACCTCCACGTACCGCAGTACGGGCCCGGGTCCATCGA
Brb_F         CGGCCTGCGCTTTCAGTTAAGGTACCTCCACGTACCGCAGTACGGGCCCGGGTCCATCGA
Brb_R         CGGCCTGCGCTTTCAGTTAAGGTACCTCCACGTACCGCAGTACGGGCCCGGGTCCATCGA
              ************************************************************

Pallas_F      TCGCCATTAGCTTAGCTCCCCCGTCGCCATGATTAATGGCGCCCGGGCTAGCTTCCAAAA
Pallas_R      TCGCCATTAGCTTAGCTCCCCCGTCGCCATGATTAATGGCGCCCGGGCTAGCTTCCAAAA
Brb_F         TCGCCATTAGCTTAGCTCCCCCGTCGCCATGATTAATGGCGCCCGGGCTAGCTTCCAAAA
Brb_R         TCGCCATTAGCTTAGCTCCCCCGTCGCCATGATTAATGGCGCCCGGGCTAGCTTCCAAAA
              ************************************************************

Pallas_F      CCCGATCCATTGGCTCGATCCGAACGGAATGCTGCCGCATTTACTCGCCTGCTTTTTTGA
Pallas_R      CCCGATCCATTGGCTCGATCCGAACGGAATGCTGCCGCATTTACTCGCCTGCTTTTTTGA
Brb_F         CCCGATCCATTGGCTCGATCCGAACGGAATGCTGCCGCATTTACTCGCCTGCTTTTTTGA
Brb_R         CCCGATCCATTGGCTCGATCCGAACGGAATGCTGCCGCATTTACTCGCCTGCTTTTTTGA
              ************************************************************
```

```
Pallas_F      ATGCGTGCTGCGATTTCAGCTTGCCGCGCATGCAGCCGTGTCCAGCCGCCGGGTGTTCGG
Pallas_R      ATGCGTGCTGCGATTTCAGCTTGCCGCGCATGCAGCCGTGTCCAGCCGCCGGGTGTTCGG
Brb_F         ATGCGTGCTGCGATTTCAGCTTGCCGCGCATGCAGCCGTGTCCAGCCGCCGGGTGTTCGG
Brb_R         ATGCGTGCTGCGATTTCAGCTTGCCGCGCATGCAGCCGTGTCCAGCCGCCGGGTGTTCGG
              ************************************************************

Pallas_F      TGTTCGGATGGATGATGCTTCGCTCTGCTCCTAGTAGCAGCCTCCTATAAATACCGGGCG
Pallas_R      TGTTCGGATGGATGATGCTTCGCTCTGCTCCTAGTAGCAGCCTCCTATAAATACCGGGCG
Brb_F         TGTTCGGATGGATGATGCTTCGCTCTGCTCCTAGTAGCAGCCTCCTATAAATACCGGGCG
Brb_R         TGTTCGGATGGATGATGCTTCGCTCTGCTCCTAGTAGCAGCCTCCTATAAATACCGGGCG
              ************************************************************

Pallas_F      TCGTCGTGCATCGTGCATCGATCGATCGCCAACAATTCGAATTGTCCATCTCCCGCCACG
Pallas_R      TCGTCGTGCATCGTGCATCGATCGATCGCCAACAATTCGAATTGTCCATCTCCCGCCACG
Brb_F         TCGTCGTGCATCGTGCATCGATCGATCGCCAACAATTCGAATTGTCCATCTCCCGCCACG
Brb_R         TCGTCGTGCATCGTGCATCGATCGATCGCCAACAATTCGAATTGTCCATCTCCCGCCACG
              ************************************************************

Pallas_F      CGCACCCACCCACCTACGGGAATAAGCAAGCAAGCTGGTCACTGGGGTGACGAACTAACG
Pallas_R      CGCACCCACCCACCTACGGGAATAAGCAAGCAAGCTGGTCACTGGGGTGACGAACTAACG
Brb_F         CGCACCCACCCACCTACGGGAATAAGCAAGCAAGCTGGTCACTGGGGTGACGAACTAACG
Brb_R         CGCACCCACCCACCTACGGGAATAAGCAAGCAAGCTGGTCACTGGGGTGACGAACTAACG
              ************************************************************

Pallas_F      ATCGATGGCCCGTTTGGTTAGAGACGGCGGTCGTGGACGAGGGGTGGCGGCGGTGGAGGA
Pallas_R      ATCGATGGCCCGTTTGGTTAGAGACGGCGGTCGTGGACGAGGGGTGGCGGCGGTGGAGGA
Brb_F         ATCGATGGCCCGTTTGGTTAGAGACGGCGGTCGTGGACGAGGGGTGGCGGCGGTGGAGGA
Brb_R         ATCGATGGCCCGTTTGGTTAGAGACGGCGGTCGTGGACGAGGGGTGGCGGCGGTGGAGGA
              ************************************************************

Pallas_F      GATGGCGCCGCCACTGCCTCTGCCTCCGCCGCCGCGCCTCGCGTCGGCGGCGTTGTCTTC
Pallas_R      GATGGCGCCGCCACTGCCTCTGCCTCCGCCGCCGCGCCTCGCGTCGGCGGCGTTGTCTTC
Brb_F         GATGGCGCCGCCACTGCCTCTGCCTCCGCCGCCGCGCCTCGCGTCGGCGGCGTTGTCTTC
Brb_R         GATGGCGCCGCCACTGCCTCTGCCTCCGCCGCCGCGCCTCGCGTCGGCGGCGTTGTCTTC
              ************************************************************

Pallas_F      CTCGTCGCCGGCGTCCATCCGTGCGCTGCTGGCGAGGACCGGGGGCGGGGCGGACTGCCA
Pallas_R      CTCGTCGCCGGCGTCCATCCGTGCGCTGCTGGCGAGGACCGGGGGCGGGGCGGACTGCCA
Brb_F         CTCGTCGCCGGCGTCCATCCGTGCGCTGCTGGCGAGGACCGGGGGCGGGGCGGACTGCCA
Brb_R         CTCGTCGCCGGCGTCCATCCGTGCGCTGCTGGCGAGGACCGGGGGCGGGGCGGACTGCCA
              ************************************************************

Pallas_F      GCAGTCGCCGCGGTCGCTGCTGTCCCGCATCCTGCTGCGCGGCGGCGGCGATCATCACGG
Pallas_R      GCAGTCGCCGCGGTCGCTGCTGTCCCGCATCCTGCTGCGCGGCGGCGGCGATCATCACGG
Brb_F         GCAGTCGCCGCGGTCGCTGCTGTCCCGCATCCTGCTGCGCGGCGGCGGCGATCATCACGG
Brb_R         GCAGTCGCCGCGGTCGCTGCTGTCCCGCATCCTGCTGCGCGGCGGCGGCGATCATCACGG
              ************************************************************

Pallas_F      CGGGAACGGGGGAGGGTCGTTCGGGTGCCGGGTCAGGCTTCCGCGGCGGTACGGTAGTAG
Pallas_R      CGGGAACGGGGGAGGGTCGTTCGGGTGCCGGGTCAGGCTTCCGCGGCGGTACGGTAGTAG
Brb_F         CGGGAACGGGGGAGGGTCGTTCGGGTGCCGGGTCAGGCTTCCGCGGCGGTACGGTAGTAG
Brb_R         CGGGAACGGGGGAGGGTCGTTCGGGTGCCGGGTCAGGCTTCCGCGGCGGTACGGTAGTAG
              ************************************************************

Pallas_F      CAGCTCGTCCGTCGGCGACAGCATCAGGGAGGAGAGGAAGGACGACGGCGCTGCCTCCGA
Pallas_R      CAGCTCGTCCGTCGGCGACAGCATCAGGGAGGAGAGGAAGGACGACGGCGCTGCCTCCGA
Brb_F         CAGCTCGTCCGTCGGCGACAGCATCAGGGAGGAGAGGAAGGACGACGGCGCTGCCTCCGA
Brb_R         CAGCTCGTCCGTCGGCGACAGCATCAGGGAGGAGAGGAAGGACGACGGCGCTGCCTCCGA
              ************************************************************

Pallas_F      GCAGTCGGCGGACGACGTCGGCTCCGCCAGGGTCAAGGTTGTCGAGCGCTCGCCGGAGCT
Pallas_R      GCAGTCGGCGGACGACGTCGGCTCCGCCAGGGTCAAGGTTGTCGAGCGCTCGCCGGAGCT
Brb_F         GCAGTCGGCGGACGACGTCGGCTCCGCCAGGGTCAAGGTTGTCGAGCGCTCGCCGGAGCT
Brb_R         GCAGTCGGCGGACGACGTCGGCTCCGCCAGGGTCAAGGTTGTCGAGCGCTCGCCGGAGCT
              ************************************************************

Pallas_F      GCCCGTCGACACGCCCCGGAGCTCCCTAGGTAATTGCCATATAGATCGTCTACGTACAGT
Pallas_R      GCCCGTCGACACGCCCCGGAGCTCCCTAGGTAATTGCCATATAGATCGTCTACGTACAGT
Brb_F         GCCCGTCGACACGCCCCGGAGCTCCCTAGGTAATTGCCATATAGATCGTCTACGTACAGT
```

```
Brb_R           GCCCGTCGACACGCCCCGGAGCTCCCTAGGTAATTGCCATATAGATCGTCTACGTACAGT
                ************************************************************

Pallas_F        TCGTAGCGATTAGTAGATTAGATGATGTTGATCGGAACTTGTGGATGATCGATGCAGGCA
Pallas_R        TCGTAGCGATTAGTAGATTAGATGATGTTGATCGGAACTTGTGGATGATCGATGCAGGCA
Brb_F           TCGTAGCGATTAGTAGATTAGATGATGTTGATCGGAACTTGTGGATGATCGATGCAGGCA
Brb_R           TCGTAGCGATTAGTAGATTAGATGATGTTGATCGGAACTTGTGGATGATCGATGCAGGCA
                ************************************************************

Pallas_F        AGAAGAAGCCGGAGGAGGAGGTCATGTCGATGAGCCTTCGGCTGGGCGCGAGCCTGGTGC
Pallas_R        AGAAGAAGCCGGAGGAGGAGGTCATGTCGATGAGCCTTCGGCTGGGCGCGAGCCTGGTGC
Brb_F           AGAAGAAGCCGGAGGAGGAGGTCATGTCGATGAGCCTTCGGCTGGGCGCGAGCCTGGTGC
Brb_R           AGAAGAAGCCGGAGGAGGAGGTCATGTCGATGAGCCTTCGGCTGGGCGCGAGCCTGGTGC
                ************************************************************

Pallas_F        TGCTGCTCTCCAAGAGCGCGGTGGAGCTGAACAAGATGGTGGAGCTCCGCGCGCAGATGG
Pallas_R        TGCTGCTCTCCAAGAGCGCGGTGGAGCTGAACAAGATGGTGGAGCTCCGCGCGCAGATGG
Brb_F           TGCTGCTCTCCAAGAGCGCGGTGGAGCTGAACAAGATGGTGGAGCTCCGCGCGCAGATGG
Brb_R           TGCTGCTCTCCAAGAGCGCGGTGGAGCTGAACAAGATGGTGGAGCTCCGCGCGCAGATGG
                ************************************************************

Pallas_F        AGGCGCTCGTGTCGGAGATCAGGCACGAGACCATCGGGAAGGAGAAGCACGGCGGCTCTG
Pallas_R        AGGCGCTCGTGTCGGAGATCAGGCACGAGACCATCGGGAAGGAGAAGCACGGCGGCTCTG
Brb_F           AGGCGCTCGTGTCGGAGATCAGGCACGAGACCATCGGGAAGGAGAAGCACGGCGGCTCTG
Brb_R           AGGCGCTCGTGTCGGAGATCAGGCACGAGACCATCGGGAAGGAGAAGCACGGCGGCTCTG
                ************************************************************

Pallas_F        CTCCGGCCGCCTCGTCCTCCTCCTCCCAGGAGTCCACCGTGATCAAGGACCCCATCGCCC
Pallas_R        CTCCGGCCGCCTCGTCCTCCTCCTCCCAGGAGTCCACCGTGATCAAGGACCCCATCGCCC
Brb_F           CTCCGGCCGCCTCGTCCTCCTCCTCCCAGGAGTCCACCGTGATCAAGGACCCCATCGCCC
Brb_R           CTCCGGCCGCCTCGTCCTCCTCCTCCCAGGAGTCCACCGTGATCAAGGACCCCATCGCCC
                ************************************************************

Pallas_F        GCGCCGAGGACGCGCTGTCCGGCAACTGCTCCGGCGCCCGGACCGCTGATCGCCGCCAGC
Pallas_R        GCGCCGAGGACGCGCTGTCCGGCAACTGCTCCGGCGCCCGGACCGCTGATCGCCGCCAGC
Brb_F           GCGCCGAGGACGCGCTGTCCGGCAACTGCTCCGGCGCCCGGACCGCTGATCGCCGCCAGC
Brb_R           GCGCCGAGGACGCGCTGTCCGGCAACTGCTCCGGCGCCCGGACCGCTGATCGCCGCCAGC
                ************************************************************

Pallas_F        TTTCCGCCGCCGTCGTCGCGATGGACCATAACAAGATGGAGGCCGAGCTCCAGCTCGAGC
Pallas_R        TTTCCGCCGCCGTCGTCGCGATGGACCATAACAAGATGGAGGCCGAGCTCCAGCTCGAGC
Brb_F           TTTCCGCCGCCGTCGTCGCGATGGACCATAACAAGATGGAGGCCGAGCTCCAGCTCGAGC
Brb_R           TTTCCGCCGCCGTCGTCGCGATGGACCATAACAAGATGGAGGCCGAGCTCCAGCTCGAGC
                ************************************************************

Pallas_F        TGAGCCGCATGCAGGCGCAGCATCGCGCCATGCACGCGCCCATCAGAGGGCTCGAGGTAC
Pallas_R        TGAGCCGCATGCAGGCGCAGCATCGCGCCATGCACGCGCCCATCAGAGGGCTCGAGGTAC
Brb_F           TGAGCCGCATGCAGGCGCAGCATCGCGCCATGCACGCGCCCATCAGAGGGCTCGAGGTAC
Brb_R           TGAGCCGCATGCAGGCGCAGCATCGCGCCATGCACGCGCCCATCAGAGGGCTCGAGGTAC
                ************************************************************

Pallas_F        GTAGCGTACGTACGATCGAGTGTACCGCACGGCATATCTTGGTATCTTGAGTAGCACGTA
Pallas_R        GTAGCGTACGTACGATCGAGTGTACCGCACGGCATATCTTGGTATCTTGAGTAGCACGTA
Brb_F           GTAGCGTACGTACGATCGAGTGTACCGCACGGCATATCTTGGTATCTTGAGTAGCACGTA
Brb_R           GTAGCGTACGTACGATCGAGTGTACCGCACGGCATATCTTGGTATCTTGAGTAGCACGTA
                ************************************************************

Pallas_F        CTCCATGCATGCATGCATGCACAGCGAATTGCGTCCCGTTCCCGCGCTTGCTTGCTGATG
Pallas_R        CTCCATGCATGCATGCATGCACAGCGAATTGCGTCCCGTTCCCGCGCTTGCTTGCTGATG
Brb_F           CTCCATGCATGCATGCATGCACAGCGAATTGCGTCCCGTTCCCGCGCTTGCTTGCTGATG
Brb_R           CTCCATGCATGCATGCATGCACAGCGAATTGCGTCCCGTTCCCGCGCTTGCTTGCTGATG
                ************************************************************

Pallas_F        ATGCACTTACGCGTCTTCTCTTTTTGGCCAGCTGCCGCCGCTGCAGGTGAAGACGGCGAG
Pallas_R        ATGCACTTACGCGTCTTCTCTTTTTGGCCAGCTGCCGCCGCTGCAGGTGAAGACGGCGAG
Brb_F           ATGCACTTACGCGTCTTCTCTTTTTGGCCAGCTGCCGCCGCTGCAGGTGAAGACGGCGAG
Brb_R           ATGCACTTACGCGTCTTCTCTTTTTGGCCAGCTGCCGCCGCTGCAGGTGAAGACGGCGAG
                ************************************************************
```

```
Pallas_F     GAGCGCGCACGTGTCCGTCGACACGACGTCGAGGAGCTGCGTCGTCGACCACGCGACGCA
Pallas_R     GAGCGCGCACGTGTCCGTCGACACGACGTCGAGGAGCTGCGTCGTCGACCACGCGACGCA
Brb_F        GAGCGCGCACGTGTCCGTCGACACGACGTCGAGGAGCTGCGTCGTCGACCACGCGACGCA
Brb_R        GAGCGCGCACGTGTCCGTCGACACGACGTCGAGGAGCTGCGTCGTCGACCACGCGACGCA
             ************************************************************

Pallas_F     GGTGAACGCCGACGAGGAGGACGGCGAGGACGAAGAGGATCAGCGCGAGGAGGACTACGA
Pallas_R     GGTGAACGCCGACGAGGAGGACGGCGAGGACGAAGAGGATCAGCGCGAGGAGGACTACGA
Brb_F        GGTGAACGCCGACGAGGAGGACGGCGAGGACGAAGAGGATCAGCGCGAGGAGGACTACGA
Brb_R        GGTGAACGCCGACGAGGAGGACGGCGAGGACGAAGAGGATCAGCGCGAGGAGGACTACGA
             ************************************************************

Pallas_F     CGAGGAGGAGGAGGGCGACGACGACGACGACGGTGGCGAGGTGGTGGACCGCGACAGGAG
Pallas_R     CGAGGAGGAGGAGGGCGACGACGACGACGACGGTGGCGAGGTGGTGGACCGCGACAGGAG
Brb_F        CGAGGAGGAGGAGGGCGACGACGACGACGACGGTGGCGAGGTGGTGGACCGCGACAGGAG
Brb_R        CGAGGAGGAGGAGGGCGACGACGACGACGACGGTGGCGAGGTGGTGGACCGCGACAGGAG
             ************************************************************

Pallas_F     CCCCCCGCACGGCGGCGTGTCGGCGCGCGCGCTGGAACGGCGGCTGCACGAGCTGCTGCA
Pallas_R     CCCCCCGCACGGCGGCGTGTCGGCGCGCGCGCTGGAACGGCGGCTGCACGAGCTGCTGCA
Brb_F        CCCCCCGCACGGCGGCGTGTCGGCGCGCGCGCTGGAACGGCGGCTGCACGAGCTGCTGCA
Brb_R        CCCCCCGCACGGCGGCGTGTCGGCGCGCGCGCTGGAACGGCGGCTGCACGAGCTGCTGCA
             ************************************************************

Pallas_F     GCGGCGGCAGCAGGACCGCATCGTGGAGCTGGAGGCGGCGCTGGACGGCGCCCAGCGGCG
Pallas_R     GCGGCGGCAGCAGGACCGCATCGTGGAGCTGGAGGCGGCGCTGGACGGCGCCCAGCGGCG
Brb_F        GCGGCGGCAGCAGGACCGCATCGTGGAGCTGGAGGCGGCGCTGGACGGCGCCCAGCGGCG
Brb_R        GCGGCGGCAGCAGGACCGCATCGTGGAGCTGGAGGCGGCGCTGGACGGCGCCCAGCGGCG
             ************************************************************

Pallas_F     GCTCCAGGAGCGGGAGCGCGAGGTGGTGTGGTGGCGCGACGCCGCAAAGCTCGTGTCCCA
Pallas_R     GCTCCAGGAGCGGGAGCGCGAGGTGGTGTGGTGGCGCGACGCCGCAAAGCTCGTGTCCCA
Brb_F        GCTCCAGGAGCGGGAGCGCGAGGTGGTGTGGTGGCGCGACGCCGCAAAGCTCGTGTCCCA
Brb_R        GCTCCAGGAGCGGGAGCGCGAGGTGGTGTGGTGGCGCGACGCCGCAAAGCTCGTGTCCCA
             ************************************************************

Pallas_F     CCGCCGCGACGAGTCCCGCCGCCTCAGGTGCACCGCCCCCGAGCCGGTCCGATAAGCACA
Pallas_R     CCGCCGCGACGAGTCCCGCCGCCTCAGGTGCACCGCCCCCGAGCCGGTCCGATAAGCACA
Brb_F        CCGCCGCGACGAGTCCCGCCGCCTCAGGTGCACCGCCCCCGAGCCGGTCCGATAAGCACA
Brb_R        CCGCCGCGACGAGTCCCGCCGCCTCAGGTGCACCGCCCCCGAGCCGGTCCGATAAGCACA
             ************************************************************

Pallas_F     CCCACACGCACACGCACGCGCACACGCGCGTGTCAGCTCTCGGCTCACGTGTGCGTGGCA
Pallas_R     CCCACACGCACACGCACGCGCACACGCGCGTGTCAGCTCTCGGCTCACGTGTGCGTGGCA
Brb_F        CCCACACGCACACGCACGCGCACACGCGCGTGTCAGCTCTCGGCTCACGTGTGCGTGGCA
Brb_R        CCCACACGCACACGCACGCGCACACGCGCGTGTCAGCTCTCGGCTCACGTGTGCGTGGCA
             ************************************************************

Pallas_F     TGGCAGCTCTAGCTAGCGAGTAATGGCGGGGCAGCCGCCATGTCAGGGTGTGTGCAGTCG
Pallas_R     TGGCAGCTCTAGCTAGCGAGTAATGGCGGGGCAGCCGCCATGTCAGGGTGTGTGCAGTCG
Brb_F        TGGCAGCTCTAGCTAGCGAGTAATGGCGGGGCAGCCGCCATGTCAGGGTGTGTGCAGTCG
Brb_R        TGGCAGCTCTAGCTAGCGAGTAATGGCGGGGCAGCCGCCATGTCAGGGTGTGTGCAGTCG
             ************************************************************

Pallas_F     GGCTTGTGGAGGGAGAGGAGTGGAGGTCTCCTGGTCACGCTGCGCGGGTGAGGCGGCGTT
Pallas_R     GGCTTGTGGAGGGAGAGGAGTGGAGGTCTCCTGGTCACGCTGCGCGGGTGAGGCGGCGTT
Brb_F        GGCTTGTGGAGGGAGAGGAGTGGAGGTCTCCTGGTCACGCTGCGCGGGTGAGGCGGCGTT
Brb_R        GGCTTGTGGAGGGAGAGGAGTGGAGGTCTCCTGGTCACGCTGCGCGGGTGAGGCGGCGTT
             ************************************************************

Pallas_F     AATAACGGCCCCCGATCGGGCTCGGTTTGGCGCGCAACCGCGCGCGCGCAGGCGCTCGGA
Pallas_R     AATAACGGCCCCCGATCGGGCTCGGTTTGGCGCGCAACCGCGCGCGCGCAGGCGCTCGGA
Brb_F        AATAACGGCCCCCGATCGGGCTCGGTTTGGCGCGCAACCGCGCGCGCGCAGGCGCTCGGA
Brb_R        AATAACGGCCCCCGATCGGGCTCGGTTTGGCGCGCAACCGCGCGCGCGCAGGCGCTCGGA
             ************************************************************

Pallas_F     GATGGCGGGAAATGGGTGCGTGTGTGGGGTCTGCGCTGTAATAACAACCATTGTTCAGAG
Pallas_R     GATGGCGGGAAATGGGTGCGTGTGTGGGGTCTGCGCTGTAATAACAACCATTGTTCAGAG
Brb_F        GATGGCGGGAAATGGGTGCGTGTGTGGGGTCTGCGCTGTAATAACAACCATTGTTCAGAG
```

```
Brb_R           GATGGCGGGAAATGGGTGCGTGTGTGGGGTCTGCGCTGTAATAACAACCATTGTTCAGAG
                ************************************************************

Pallas_F        TTTTTGTTTCGGCTGTCCTGCTGCCCGATTGATTGCTTGCTAGCGTCGATGCGTCCTTCG
Pallas_R        TTTTTGTTTCGGCTGTCCTGCTGCCCGATTGATTGCTTGCTAGCGTCGATGCGTCCTTCG
Brb_F           TTTTTGTTTCGGCTGTCCTGCTGCCCGATTGATTGCTTGCTAGCGTCGATGCGTCCTTCG
Brb_R           TTTTTGTTTCGGCTGTCCTGCTGCCCGATTGATTGCTTGCTAGCGTCGATGCGTCCTTCG
                ************************************************************

Pallas_F        TTTTATTTATTATATTATATATGCTCCTACGTATGTTTCCAAATTCTGCGAGGGAAAGAC
Pallas_R        TTTTATTTATTATATTATATATGCTCCTACGTATGTTTCCAAATTCTGCGAGGGAAAGAC
Brb_F           TTTTATTTATTATATTATATATGCTCCTACGTATGTTTCCAAATTCTGCGAGGGAAAGAC
Brb_R           TTTTATTTATTATATTATATATGCTCCTACGTATGTTTCCAAATTCTGCGAGGGAAAGAC
                ************************************************************

Pallas_F        AGAAGAGCTGGGGCCTGGTTTCAGAAACCAGAATTTCGGTTTCTGAAGCTCTGCTCTGAT
Pallas_R        AGAAGAGCTGGGGCCTGGTTTCAGAAACCAGAATTTCGGTTTCTGAAGCTCTGCTCTGAT
Brb_F           AGAAGAGCTGGGGCCTGGTTTCAGAAACCAGAATTTCGGTTTCTGAAGCTCTGCTCTGAT
Brb_R           AGAAGAGCTGGGGCCTGGTTTCAGAAACCAGAATTTCGGTTTCTGAAGCTCTGCTCTGAT
                ************************************************************

Pallas_F        TTTTCCAAATTCTGCGAGGGGCAGCTTGTTTCCGAACCTGTTCTGAGCTCTGCTGCGTTC
Pallas_R        TTTTCCAAATTCTGCGAGGGGCAGCTTGTTTCCGAACCTGTTCTGAGCTCTGCTGCGTTC
Brb_F           TTTTCCAAATTCTGCGAGGGGCAGCTTGTTTCCGAACCTGTTCTGAGCTCTGCTGCGTTC
Brb_R           TTTTCCAAATTCTGCGAGGGGCAGCTTGTTTCCGAACCTGTTCTGAGCTCTGCTGCGTTC
                ************************************************************

Pallas_F        TGGTTCCAAATTCAGCGAGGGAAAAACGGAGCTGGGGCCTGGTTTCTGAATTTGGTTCTG
Pallas_R        TGGTTCCAAATTCAGCGAGGGAAAAACGGAGCTGGGGCCTGGTTTCTGAATTTGGTTCTG
Brb_F           TGGTTCCAAATTCAGCGAGGGAAAAACGGAGCTGGGGCCTGGTTTCTGAATTTGGTTCTG
Brb_R           TGGTTCCAAATTCAGCGAGGGAAAAACGGAGCTGGGGCCTGGTTTCTGAATTTGGTTCTG
                ************************************************************

Pallas_F        GGCTCTGCTCATGCTCCAAACTCTGGGAGGGAAAATGGGCAGGGACCAGGTATATACTAG
Pallas_R        GGCTCTGCTCATGCTCCAAACTCTGGGAGGGAAAATGGGCAGGGACCAGGTATATACTAG
Brb_F           GGCTCTGCTCATGCTCCAAACTCTGGGAGGGAAAATGGGCAGGGACCAGGTATATACTAG
Brb_R           GGCTCTGCTCATGCTCCAAACTCTGGGAGGGAAAATGGGCAGGGACCAGGTATATACTAG
                ************************************************************

Pallas_F        CGTAGTACATTATCTCGAGCAGGAACTGTCTGATTCTAGCCGAATTTACTCGGGTGCTGC
Pallas_R        CGTAGTACATTATCTCGAGCAGGAACTGTCTGATTCTAGCCGAATTTACTCGGGTGCTGC
Brb_F           CGTAGTACATTATCTCGAGCAGGAACTGTCTGATTCTAGCCGAATTTACTCGGGTGCTGC
Brb_R           CGTAGTACATTATCTCGAGCAGGAACTGTCTGATTCTAGCCGAATTTACTCGGGTGCTGC
                ************************************************************

Pallas_F        GGCTCAGCAGATTGGCTGCTGCTGCTGTTGCGGTGTGTCCTTGGGGCTGGCCGGCTGGGG
Pallas_R        GGCTCAGCAGATTGGCTGCTGCTGCTGTTGCGGTGTGTCCTTGGGGCTGGCCGGCTGGGG
Brb_F           GGCTCAGCAGATTGGCTGCTGCTGCTGTTGCGGTGTGTCCTTGGGGCTGGCCGGCTGGGG
Brb_R           GGCTCAGCAGATTGGCTGCTGCTGCTGTTGCGGTGTGTCCTTGGGGCTGGCCGGCTGGGG
                ************************************************************

Pallas_F        CATTCATTTGGGCGGCAAACGGGGCGTTAGTTAGGACAGGGAAACCGACAGATGCATGCG
Pallas_R        CATTCATTTGGGCGGCAAACGGGGCGTTAGTTAGGACAGGGAAACCGACAGATGCATGCG
Brb_F           CATTCATTTGGGCGGCAAACGGGGCGTTAGTTAGGACAGGGAAACCGACAGATGCATGCG
Brb_R           CATTCATTTGGGCGGCAAACGGGGCGTTAGTTAGGACAGGGAAACCGACAGATGCATGCG
                ************************************************************

Pallas_F        GCGCAGCAGCAGTAACAGTCTAACAGACAGTGGTAAGAAATGGAAGGAGGAGAAGCTGCG
Pallas_R        GCGCAGCAGCAGTAACAGTCTAACAGACAGTGGTAAGAAATGGAAGGAGGAGAAGCTGCG
Brb_F           GCGCAGCAGCAGTAACAGTCTAACAGACAGTGGTAAGAAATGGAAGGAGGAGAAGCTGCG
Brb_R           GCGCAGCAGCAGTAACAGTCTAACAGACAGTGGTAAGAAATGGAAGGAGGAGAAGCTGCG
                ************************************************************

Pallas_F        CGCTCTGCTCT
Pallas_R        CGCTCTGCTCT
Brb_F           CGCTCTGCTCT
Brb_R           CGCTCTGCTCT
                ***********
```

Alignment_HORVU7Hr1G030270

```
Pallas_F      CATTGTGGGTAGACCCAAATAAGTGTCTGACAATGCCTCAGTAACAATGTCAAGTTCTCT
Pallas_R      CATTGTGGGTAGACCCAAATAAGTGTCTGACAATGCCTCAGTAACAATGTCAAGTTCTCT
Brb_F         CATTGTGGGTAGACCCAAATAAGTGTCTGACAATGCCTCAGTAACAATGTCAAGTTCTCT
Brb_R         CATTGTGGGTAGACCCAAATAAGTGTCTGACAATGCCTCAGTAACAATGTCAAGTTCTCT
              ************************************************************

Pallas_F      GCAGATATTCTCTCTCACACAGACCCCGGTATTCGGGCTGAACAATATACTAGATTTTGC
Pallas_R      GCAGATATTCTCTCTCACACAGACCCCGGTATTCGGGCTGAACAATATACTAGATTTTGC
Brb_F         GCAGATATTCTCTCTCACACAGACCCCGGTATTCGGGCTGAACAATATACTAGATTTTGC
Brb_R         GCAGATATTCTCTCTCACACAGACCCCGGTATTCGGGCTGAACAATATACTAGATTTTGC
              ************************************************************

Pallas_F      AGTACTTACAACTTGCCCTGAGCTGGTACAATAAAGATCCAACACCTTCTTAAGATTGCT
Pallas_R      AGTACTTACAACTTGCCCTGAGCTGGTACAATAAAGATCCAACACCTTCTTAAGATTGCT
Brb_F         AGTACTTACAACTTGCCCTGAGCTGGTACAATAAAGATCCAACACCTTCTTAAGATTGCT
Brb_R         AGTACTTACAACTTGCCCTGAGCTGGTACAATAAAGATCCAACACCTTCTTAAGATTGCT
              ************************************************************

Pallas_F      TGCATTCTGGGCATTGGCTTTCATCAACACCAACGAGTCATCTGCAAATAAAAGATGTGA
Pallas_R      TGCATTCTGGGCATTGGCTTTCATCAACACCAACGAGTCATCTGCAAATAAAAGATGTGA
Brb_F         TGCATTCTGGGCATTGGCTTTCATCAACACCAACGAGTCATCTGCAAATAAAAGATGTGA
Brb_R         TGCATTCTGGGCATTGGCTTTCATCAACACCAACGAGTCATCTGCAAATAAAAGATGTGA
              ************************************************************

Pallas_F      TACAGCGGGTGCACTCCTACACACCTTAACACACTCCAATCCTCGAGATTCCTCCTCGTG
Pallas_R      TACAGCGGGTGCACTCCTACACACCTTAACACACTCCAATCCTCGAGATTCCTCCTCGTG
Brb_F         TACAGCGGGTGCACTCCTACACACCTTAACACACTCCAATCCTCGAGATTCCTCCTCGTG
Brb_R         TACAGCGGGTGCACTCCTACACACCTTAACACACTCCAATCCTCGAGATTCCTCCTCGTG
              ************************************************************

Pallas_F      AGCTAATAAGCTTTTTTTTAAGAAGTGAGCTAATAAGCTTGATAGACCTTCAGAGCATAT
Pallas_R      AGCTAATAAGCTTTTTTTTAAGAAGTGAGCTAATAAGCTTGATAGACCTTCAGAGCATAT
Brb_F         AGCTAATAAGCTTTTTTTTAAGAAGTGAGCTAATAAGCTTGATAGACCTTCAGAGCATAT
Brb_R         AGCTAATAAGCTTTTTTTTAAGAAGTGAGCTAATAAGCTTGATAGACCTTCAGAGCATAT
              ************************************************************

Pallas_F      TAAGAACAAATATGGGATAACGGATCCCCCTATCTCAGGCCTCTACCGGGCACAAATTCG
Pallas_R      TAAGAACAAATATGGGATAACGGATCCCCCTATCTCAGGCCTCTACCGGGCACAAATTCG
Brb_F         TAAGAACAAATATGGGATAACGGATCCCCCTATCTCAGGCCTCTACCGGGCACAAATTCG
Brb_R         TAAGAACAAATATGGGATAACGGATCCCCCTATCTCAGGCCTCTACCGGGCACAAATTCG
              ************************************************************

Pallas_F      TTCGTCTCCGTATTATTAGAAACGCACTCTGTACGTGACCGAGGAAACACACTCCATATT
Pallas_R      TTCGTCTCCGTATTATTAGAAACGCACTCTGTACGTGACCGAGGAAACACACTCCATATT
Brb_F         TTCGTCTCCGTATTATTAGAAACGCACTCTGTACGTGACCGAGGAAACACACTCCATATT
Brb_R         TTCGTCTCCGTATTATTAGAAACGCACTCTGTACGTGACCGAGGAAACACACTCCATATT
              ************************************************************

Pallas_F      CAACACCTCTTCAATTCTTCCTCTAGCCCAATAACCAACCCGTCACTACGTTCTCCCTCG
Pallas_R      CAACACCTCTTCAATTCTTCCTCTAGCCCAATAACCAACCCGTCACTACGTTCTCCCTCG
Brb_F         CAACACCTCTTCAATTCTTCCTCTAGCCCAATAACCAACCCGTCACTACGTTCTCCCTCG
Brb_R         CAACACCTCTTCAATTCTTCCTCTAGCCCAATAACCAACCCGTCACTACGTTCTCCCTCG
              ************************************************************

Pallas_F      AACAGAGTCGCCCCGGTTTGCAGCGCCGCACCACGGGACCTCTCCGGCCGGCGACGACAA
Pallas_R      AACAGAGTCGCCCCGGTTTGCAGCGCCGCACCACGGGACCTCTCCGGCCGGCGACGACAA
Brb_F         AACAGAGTCGCCCCGGTTTGCAGCGCCGCACCACGGGACCTCTCCGGCCGGCGACGACAA
Brb_R         AACAGAGTCGCCCCGGTTTGCAGCGCCGCACCACGGGACCTCTCCGGCCGGCGACGACAA
              ************************************************************

Pallas_F      CCGGCCTCCGCCGGCAAACGGCGCACGCAGCGCAGGTAAGCTTCTTTCCCTCCATCCCCG
Pallas_R      CCGGCCTCCGCCGGCAAACGGCGCACGCAGCGCAGGTAAGCTTCTTTCCCTCCATCCCCG
Brb_F         CCGGCCTCCGCCGGCAAACGGCGCACGCAGCGCAGGTAAGCTTCTTTCCCTCCATCCCCG
Brb_R         CCGGCCTCCGCCGGCAAACGGCGCACGCAGCGCAGGTAAGCTTCTTTCCCTCCATCCCCG
              ************************************************************
```

```
Pallas_F      CCCCCAACCCGCTAAATCCACTCGCCCGCTCGGAAAAACAAAATGAAACGTCTCGTCGGC
Pallas_R      CCCCCAACCCGCTAAATCCACTCGCCCGCTCGGAAAAACAAAATGAAACGTCTCGTCGGC
Brb_F         CCCCCAACCCGCTAAATCCACTCGCCCGCTCGGAAAAACAAAATGAAACGTCTCGTCGGC
Brb_R         CCCCCAACCCGCTAAATCCACTCGCCCGCTCGGAAAAACAAAATGAAACGTCTCGTCGGC
              ************************************************************

Pallas_F      GCCGCGTCCGCCGCCTCTCACGCGAGCGAGCCAGCGGAGGCGCTTTCTCAGGCGACCCGT
Pallas_R      GCCGCGTCCGCCGCCTCTCACGCGAGCGAGCCAGCGGAGGCGCTTTCTCAGGCGACCCGT
Brb_F         GCCGCGTCCGCCGCCTCTCACGCGAGCGAGCCAGCGGAGGCGCTTTCTCAGGCGACCCGT
Brb_R         GCCGCGTCCGCCGCCTCTCACGCGAGCGAGCCAGCGGAGGCGCTTTCTCAGGCGACCCGT
              ************************************************************

Pallas_F      CCGCCGCCTCCTCCTCCTGTGCGGGTCTTCGTCTCTGTGCGGGTCTTCGTCTCCGGCGAC
Pallas_R      CCGCCGCCTCCTCCTCCTGTGCGGGTCTTCGTCTCTGTGCGGGTCTTCGTCTCCGGCGAC
Brb_F         CCGCCGCCTCCTCCTCCTGTGCGGGTCTTCGTCTCTGTGCGGGTCTTCGTCTCCGGCGAC
Brb_R         CCGCCGCCTCCTCCTCCTGTGCGGGTCTTCGTCTCTGTGCGGGTCTTCGTCTCCGGCGAC
              ************************************************************

Pallas_F      GCCTCCTCCTGCGGCGTCGCCCGCGAGGCTAGCCTGCTTCCTCTTCCTTCTCGCACGACG
Pallas_R      GCCTCCTCCTGCGGCGTCGCCCGCGAGGCTAGCCTGCTTCCTCTTCCTTCTCGCACGACG
Brb_F         GCCTCCTCCTGCGGCGTCGCCCGCGAGGCTAGCCTGCTTCCTCTTCCTTCTCGCACGACG
Brb_R         GCCTCCTCCTGCGGCGTCGCCCGCGAGGCTAGCCTGCTTCCTCTTCCTTCTCGCACGACG
              ************************************************************

Pallas_F      ACATGTTTTTTTTTTTAGGTTTAGGCGATTTCGGCCATGGCAGAGGGATAATTTTTTGTT
Pallas_R      ACATGTTTTTTTTTTTAGGTTTAGGCGATTTCGGCCATGGCAGAGGGATAATTTTTTGTT
Brb_F         ACATGTTTTTTTTTTTAGGTTTAGGCGATTTCGGCCATGGCAGAGGGATAATTTTTTGTT
Brb_R         ACATGTTTTTTTTTTTAGGTTTAGGCGATTTCGGCCATGGCAGAGGGATAATTTTTTGTT
              ************************************************************

Pallas_F      CGGCGAACTCAAGGAGAAATGAAATTAACCTCAGAGTGTGCTGTGTGGAAGTCCTATGCT
Pallas_R      CGGCGAACTCAAGGAGAAATGAAATTAACCTCAGAGTGTGCTGTGTGGAAGTCCTATGCT
Brb_F         CGGCGAACTCAAGGAGAAATGAAATTAACCTCAGAGTGTGCTGTGTGGAAGTCCTATGCT
Brb_R         CGGCGAACTCAAGGAGAAATGAAATTAACCTCAGAGTGTGCTGTGTGGAAGTCCTATGCT
              ************************************************************

Pallas_F      ATGCAGTCACTAACATTAGGGGGAAGTTGTCAGGCATGCCCAACTGCCCATCATTATTTT
Pallas_R      ATGCAGTCACTAACATTAGGGGGAAGTTGTCAGGCATGCCCAACTGCCCATCATTATTTT
Brb_F         ATGCAGTCACTAACATTAGGGGGAAGTTGTCAGGCATGCCCAACTGCCCATCATTATTTT
Brb_R         ATGCAGTCACTAACATTAGGGGGAAGTTGTCAGGCATGCCCAACTGCCCATCATTATTTT
              ************************************************************

Pallas_F      CTGGATCAAGCATATCATCAGCCACATCACTGCCTTCAGATTCAGTTTGTTGAGGCCACT
Pallas_R      CTGGATCAAGCATATCATCAGCCACATCACTGCCTTCAGATTCAGTTTGTTGAGGCCACT
Brb_F         CTGGATCAAGCATATCATCAGCCACATCACTGCCTTCAGATTCAGTTTGTTGAGGCCACT
Brb_R         CTGGATCAAGCATATCATCAGCCACATCACTGCCTTCAGATTCAGTTTGTTGAGGCCACT
              ************************************************************

Pallas_F      AGCTGCTTGCTGCTTCTGGTACTTTTTTTTATTGAGAACAAATAACAGTCCTTCTGTTCA
Pallas_R      AGCTGCTTGCTGCTTCTGGTACTTTTTTTTATTGAGAACAAATAACAGTCCTTCTGTTCA
Brb_F         AGCTGCTTGCTGCTTCTGGTACTTTTTTTTATTGAGAACAAATAACAGTCCTTCTGTTCA
Brb_R         AGCTGCTTGCTGCTTCTGGTACTTTTTTTTATTGAGAACAAATAACAGTCCTTCTGTTCA
              ************************************************************

Pallas_F      TGCATTCAGGAAGAAGGAATTGTCTTCAGTTTTTTCTGAAACTGAAGCTTTGTAGCCGCT
Pallas_R      TGCATTCAGGAAGAAGGAATTGTCTTCAGTTTTTTCTGAAACTGAAGCTTTGTAGCCGCT
Brb_F         TGCATTCAGGAAGAAGGAATTGTCTTCAGTTTTTTCTGAAACTGAAGCTTTGTAGCCGCT
Brb_R         TGCATTCAGGAAGAAGGAATTGTCTTCAGTTTTTTCTGAAACTGAAGCTTTGTAGCCGCT
              ************************************************************

Pallas_F      AGAATAGCAGTCCTGTAGTGCTGTTCTCGTAGAATAGCAGCCCTGTAGTGCTGTTCTTGT
Pallas_R      AGAATAGCAGTCCTGTAGTGCTGTTCTCGTAGAATAGCAGCCCTGTAGTGCTGTTCTTGT
Brb_F         AGAATAGCAGTCCTGTAGTGCTGTTCTCGTAGAATAGCAGCCCTGTAGTGCTGTTCTTGT
Brb_R         AGAATAGCAGTCCTGTAGTGCTGTTCTCGTAGAATAGCAGCCCTGTAGTGCTGTTCTTGT
              ************************************************************

Pallas_F      ATTTCCATTTTCACCTGTGCAGAGATTAAAGTTAAGTAGGTGAAAATTAATCTTTGTGAT
Pallas_R      ATTTCCATTTTCACCTGTGCAGAGATTAAAGTTAAGTAGGTGAAAATTAATCTTTGTGAT
Brb_F         ATTTCCATTTTCACCTGTGCAGAGATTAAAGTTAAGTAGGTGAAAATTAATCTTTGTGAT
```

```
Brb_R           ATTTCCATTTTCACCTGTGCAGAGATTAAAGTTAAGTAGGTGAAAATTAATCTTTGTGAT
                ************************************************************

Pallas_F        GATGATTACTTGATAAAACTACCAAGATTAAATTACTGAACTATGTCTGTTATTATCTAT
Pallas_R        GATGATTACTTGATAAAACTACCAAGATTAAATTACTGAACTATGTCTGTTATTATCTAT
Brb_F           GATGATTACTTGATAAAACTACCAAGATTAAATTACTGAACTATGTCTGTTATTATCTAT
Brb_R           GATGATTACTTGATAAAACTACCAAGATTAAATTACTGAACTATGTCTGTTATTATCTAT
                ************************************************************

Pallas_F        GTTGGGCGTCATCCGTCTATGTTGGGCGTCATCCGCCACAAGGACCATTTGATGAAAATC
Pallas_R        GTTGGGCGTCATCCGTCTATGTTGGGCGTCATCCGCCACAAGGACCATTTGATGAAAATC
Brb_F           GTTGGGCGTCATCCGTCTATGTTGGGCGTCATCCGCCACAAGGACCATTTGATGAAAATC
Brb_R           GTTGGGCGTCATCCGTCTATGTTGGGCGTCATCCGCCACAAGGACCATTTGATGAAAATC
                ************************************************************

Pallas_F        TTGCTTGTAGAGTTTAGAGAGTAGACTCTTTGCTTTTAGTTTCTCCATTCTGTTGCCATC
Pallas_R        TTGCTTGTAGAGTTTAGAGAGTAGACTCTTTGCTTTTAGTTTCTCCATTCTGTTGCCATC
Brb_F           TTGCTTGTAGAGTTTAGAGAGTAGACTCTTTGCTTTTAGTTTCTCCATTCTGTTGCCATC
Brb_R           TTGCTTGTAGAGTTTAGAGAGTAGACTCTTTGCTTTTAGTTTCTCCATTCTGTTGCCATC
                ************************************************************

Pallas_F        TGACCACAGGCTTAAAAATAAATGAGCAGGGAATTCTTGTTGTTGGTATGAACTCTAAAC
Pallas_R        TGACCACAGGCTTAAAAATAAATGAGCAGGGAATTCTTGTTGTTGGTATGAACTCTAAAC
Brb_F           TGACCACAGGCTTAAAAATAAATGAGCAGGGAATTCTTGTTGTTGGTATGAACTCTAAAC
Brb_R           TGACCACAGGCTTAAAAATAAATGAGCAGGGAATTCTTGTTGTTGGTATGAACTCTAAAC
                ************************************************************

Pallas_F        ATGTGAGTGTCCAGCATCATGACTCTTGATACTTGCCCTATATTTTATTTTCCTAAATCT
Pallas_R        ATGTGAGTGTCCAGCATCATGACTCTTGATACTTGCCCTATATTTTATTTTCCTAAATCT
Brb_F           ATGTGAGTGTCCAGCATCATGACTCTTGATACTTGCCCTATATTTTATTTTCCTAAATCT
Brb_R           ATGTGAGTGTCCAGCATCATGACTCTTGATACTTGCCCTATATTTTATTTTCCTAAATCT
                ************************************************************

Pallas_F        AACAGTATGTTTTGTATAATGTGCATAAGATTTGTAAGGACGAGAACTTGTTCACATTTT
Pallas_R        AACAGTATGTTTTGTATAATGTGCATAAGATTTGTAAGGACGAGAACTTGTTCACATTTT
Brb_F           AACAGTATGTTTTGTATAATGTGCATAAGATTTGTAAGGACGAGAACTTGTTCACATTTT
Brb_R           AACAGTATGTTTTGTATAATGTGCATAAGATTTGTAAGGACGAGAACTTGTTCACATTTT
                ************************************************************

Pallas_F        TTCCCTTCTGGAAAGAATCTGATTGGAGTGCAGCTGTGCATATTTAATTATTTATAGCAT
Pallas_R        TTCCCTTCTGGAAAGAATCTGATTGGAGTGCAGCTGTGCATATTTAATTATTTATAGCAT
Brb_F           TTCCCTTCTGGAAAGAATCTGATTGGAGTGCAGCTGTGCATATTTAATTATTTATAGCAT
Brb_R           TTCCCTTCTGGAAAGAATCTGATTGGAGTGCAGCTGTGCATATTTAATTATTTATAGCAT
                ************************************************************

Pallas_F        ATATTCAGTAAAACACAAGGTATCTGAGTAGACTAATGGTTCATTAATCCTTGGTAGGAT
Pallas_R        ATATTCAGTAAAACACAAGGTATCTGAGTAGACTAATGGTTCATTAATCCTTGGTAGGAT
Brb_F           ATATTCAGTAAAACACAAGGTATCTGAGTAGACTAATGGTTCATTAATCCTTGGTAGGAT
Brb_R           ATATTCAGTAAAACACAAGGTATCTGAGTAGACTAATGGTTCATTAATCCTTGGTAGGAT
                ************************************************************

Pallas_F        GCTACATGGATGAATCTAATCCTGTAACTCATTGGATAAGTTAATAAGATGGTGTGGCTG
Pallas_R        GCTACATGGATGAATCTAATCCTGTAACTCATTGGATAAGTTAATAAGATGGTGTGGCTG
Brb_F           GCTACATGGATGAATCTAATCCTGTAACTCATTGGATAAGTTAATAAGATGGTGTGGCTG
Brb_R           GCTACATGGATGAATCTAATCCTGTAACTCATTGGATAAGTTAATAAGATGGTGTGGCTG
                ************************************************************

Pallas_F        CTGTGTCCTGTGGAGTACTATGTTCTATGTTGCTTTCTGGATTTTCTTACCGCTCAAACT
Pallas_R        CTGTGTCCTGTGGAGTACTATGTTCTATGTTGCTTTCTGGATTTTCTTACCGCTCAAACT
Brb_F           CTGTGTCCTGTGGAGTACTATGTTCTATGTTGCTTTCTGGATTTTCTTACCGCTCAAACT
Brb_R           CTGTGTCCTGTGGAGTACTATGTTCTATGTTGCTTTCTGGATTTTCTTACCGCTCAAACT
                ************************************************************

Pallas_F        TTGCAATCCTTTTACCCTGCTACCAGTTTGCCTTATCCATAACTTGGACTGTGTTTTGAT
Pallas_R        TTGCAATCCTTTTACCCTGCTACCAGTTTGCCTTATCCATAACTTGGACTGTGTTTTGAT
Brb_F           TTGCAATCCTTTTACCCTGCTACCAGTTTGCCTTATCCATAACTTGGACTGTGTTTTGAT
Brb_R           TTGCAATCCTTTTACCCTGCTACCAGTTTGCCTTATCCATAACTTGGACTGTGTTTTGAT
                ************************************************************
```

Pallas_F GTTCTTGTTGTTGTATCCATGTGAGAACATTCGTGCTTTTTCATAACTTGTGAAGCTGCT
Pallas_R GTTCTTGTTGTTGTATCCATGTGAGAACATTCGTGCTTTTTCATAACTTGTGAAGCTGCT
Brb_F GTTCTTGTTGTTGTATCCATGTGAGAACATTCGTGCTTTTTCATAACTTGTGAAGCTGCT
Brb_R GTTCTTGTTGTTGTATCCATGTGAGAACATTCGTGCTTTTTCATAACTTGTGAAGCTGCT
****

Pallas_F ACTATTCAAATTTTGCATTATGCAGTAATGGTGAAGCTCTGTTTGCTGTAAAGTTACCAA
Pallas_R ACTATTCAAATTTTGCATTATGCAGTAATGGTGAAGCTCTGTTTGCTGTAAAGTTACCAA
Brb_F ACTATTCAAATTTTGCATTATGCAGTAATGGTGAAGCTCTGTTTGCTGTAAAGTTACCAA
Brb_R ACTATTCAAATTTTGCATTATGCAGTAATGGTGAAGCTCTGTTTGCTGTAAAGTTACCAA
****

Pallas_F TCCTATTTCTAAGGGATCAGAAACCATTTTTTCTGTCGCATATATGGGACAATTCACTTA
Pallas_R TCCTATTTCTAAGGGATCAGAAACCATTTTTTCTGTCGCATATATGGGACAATTCACTTA
Brb_F TCCTATTTCTAAGGGATCAGAAACCATTTTTTCTGTCGCATATATGGGACAATTCACTTA
Brb_R TCCTATTTCTAAGGGATCAGAAACCATTTTTTCTGTCGCATATATGGGACAATTCACTTA
****

Pallas_F TCTGGGTCATAGAAATGGATGGGAGCAATCGATTTTAGCTTGAAACGATTCTGTGAAAAA
Pallas_R TCTGGGTCATAGAAATGGATGGGAGCAATCGATTTTAGCTTGAAACGATTCTGTGAAAAA
Brb_F TCTGGGTCATAGAAATGGATGGGAGCAATCGATTTTAGCTTGAAACGATTCTGTGAAAAA
Brb_R TCTGGGTCATAGAAATGGATGGGAGCAATCGATTTTAGCTTGAAACGATTCTGTGAAAAA
****

Pallas_F TAACCGAGATATTTTCTTTCCAAGGGAGTATGGGACTTTAATTGGGGCATACTAACAATT
Pallas_R TAACCGAGATATTTTCTTTCCAAGGGAGTATGGGACTTTAATTGGGGCATACTAACAATT
Brb_F TAACCGAGATATTTTCTTTCCAAGGGAGTATGGGACTTTAATTGGGGCATACTAACAATT
Brb_R TAACCGAGATATTTTCTTTCCAAGGGAGTATGGGACTTTAATTGGGGCATACTAACAATT
****

Pallas_F CATCCAAGACCTAAAGTAATGTTAGTTCATTCTTTAGTACACTGTGATGATTTTGTTGTA
Pallas_R CATCCAAGACCTAAAGTAATGTTAGTTCATTCTTTAGTACACTGTGATGATTTTGTTGTA
Brb_F CATCCAAGACCTAAAGTAATGTTAGTTCATTCTTTAGTACACTGTGATGATTTTGTTGTA
Brb_R CATCCAAGACCTAAAGTAATGTTAGTTCATTCTTTAGTACACTGTGATGATTTTGTTGTA
****

Pallas_F TCTGTATGTCCAGTGCAACTGGCAATGGCTAATCCAACTCTAACCTGCCACTGTTCCAAC
Pallas_R TCTGTATGTCCAGTGCAACTGGCAATGGCTAATCCAACTCTAACCTGCCACTGTTCCAAC
Brb_F TCTGTATGTCCAGTGCAACTGGCAATGGCTAATCCAACTCTAACCTGCCACTGTTCCAAC
Brb_R TCTGTATGTCCAGTGCAACTGGCAATGGCTAATCCAACTCTAACCTGCCACTGTTCCAAC
****

Pallas_F TCTAACCTGCCACTGTACTTGTGTAAGCTGACAGAGCTTTTGCCCTATTGTGTATATTCT
Pallas_R TCTAACCTGCCACTGTACTTGTGTAAGCTGACAGAGCTTTTGCCCTATTGTGTATATTCT
Brb_F TCTAACCTGCCACTGTACTTGTGTAAGCTGACAGAGCTTTTGCCCTATTGTGTATATTCT
Brb_R TCTAACCTGCCACTGTACTTGTGTAAGCTGACAGAGCTTTTGCCCTATTGTGTATATTCT
****

Pallas_F TCACAAATCTAAATGTAGCAGCAAGGTATTTGAATTATAATTGCTATTATTTGAGCATCT
Pallas_R TCACAAATCTAAATGTAGCAGCAAGGTATTTGAATTATAATTGCTATTATTTGAGCATCT
Brb_F TCACAAATCTAAATGTAGCAGCAAGGTATTTGAATTATAATTGCTATTATTTGAGCATCT
Brb_R TCACAAATCTAAATGTAGCAGCAAGGTATTTGAATTATAATTGCTATTATTTGAGCATCT
****

Pallas_F TTCATATGCTGCATGGAGACCGATTCTGATTAACATTTACGTATAGGCAGTTTCAGGATG
Pallas_R TTCATATGCTGCATGGAGACCGATTCTGATTAACATTTACGTATAGGCAGTTTCAGGATG
Brb_F TTCATATGCTGCATGGAGACCGATTCTGATTAACATTTACGTATAGGCAGTTTCAGGATG
Brb_R TTCATATGCTGCATGGAGACCGATTCTGATTAACATTTACGTATAGGCAGTTTCAGGATG
****

Pallas_F GGTACTGAAGACACTAAAGATATGCTGAAGAATGCGGACTGGAAGACAGTGAGTGGTCCC
Pallas_R GGTACTGAAGACACTAAAGATATGCTGAAGAATGCGGACTGGAAGACAGTGAGTGGTCCC
Brb_F GGTACTGAAGACACTAAAGATATGCTGAAGAATGCGGACTGGAAGACAGTGAGTGGTCCC
Brb_R GGTACTGAAGACACTAAAGATATGCTGAAGAATGCGGACTGGAAGACAGTGAGTGGTCCC
****

Pallas_F GTTATTACCGAGTCAAGCCAGCCGGTTGTCAAGAAGCGTCTTCCGAAGAAAATCAGACAA
Pallas_R GTTATTACCGAGTCAAGCCAGCCGGTTGTCAAGAAGCGTCTTCCGAAGAAAATCAGACAA
Brb_F GTTATTACCGAGTCAAGCCAGCCGGTTGTCAAGAAGCGTCTTCCGAAGAAAATCAGACAA

```
Brb_R        GTTATTACCGAGTCAAGCCAGCCGGTTGTCAAGAAGCGTCTTCCGAAGAAAATCAGACAA
             ************************************************************

Pallas_F     GTCCCTGAGTGTTACTTTCTGCCTCGACGATCTTTGCCGTCTGCATTGGCAATCTACGGT
Pallas_R     GTCCCTGAGTGTTACTTTCTGCCTCGACGATCTTTGCCGTCTGCATTGGCAATCTACGGT
Brb_F        GTCCCTGAGTGTTACTTTCTGCCTCGACGATCTTTGCCGTCTGCATTGGCAATCTACGGT
Brb_R        GTCCCTGAGTGTTACTTTCTGCCTCGACGATCTTTGCCGTCTGCATTGGCAATCTACGGT
             ************************************************************

Pallas_F     GCTGTTTGTGCTGCTGGAGTTGGTACAGGGATGTTGCTTGAGGTTTGGATAAACAAAAAG
Pallas_R     GCTGTTTGTGCTGCTGGAGTTGGTACAGGGATGTTGCTTGAGGTTTGGATAAACAAAAAG
Brb_F        GCTGTTTGTGCTGCTGGAGTTGGTACAGGGATGTTGCTTGAGGTTTGGATAAACAAAAAG
Brb_R        GCTGTTTGTGCTGCTGGAGTTGGTACAGGGATGTTGCTTGAGGTTTGGATAAACAAAAAG
             ************************************************************

Pallas_F     ATCAAAGGTATGATCCTGCATTTCATGGCCGAACCCTCTGGATGAACCACTTATCATAAG
Pallas_R     ATCAAAGGTATGATCCTGCATTTCATGGCCGAACCCTCTGGATGAACCACTTATCATAAG
Brb_F        ATCAAAGGTATGATCCTGCATTTCATGGCCGAACCCTCTGGATGAACCACTTATCATAAG
Brb_R        ATCAAAGGTATGATCCTGCATTTCATGGCCGAACCCTCTGGATGAACCACTTATCATAAG
             ************************************************************

Pallas_F     AAAACTACCCGACCTGGAAATTTGTGATCTCGTACTGTACCATGGAAATTGTTGCATATC
Pallas_R     AAAACTACCCGACCTGGAAATTTGTGATCTCGTACTGTACCATGGAAATTGTTGCATATC
Brb_F        AAAACTACCCGACCTGGAAATTTGTGATCTCGTACTGTACCATGGAAATTGTTGCATATC
Brb_R        AAAACTACCCGACCTGGAAATTTGTGATCTCGTACTGTACCATGGAAATTGTTGCATATC
             ************************************************************

Pallas_F     TTTTTCATCCTCATCGATTGTGTTTTTCCACACCCTCTTACTTTTTGTCGATTGCTGCAT
Pallas_R     TTTTTCATCCTCATCGATTGTGTTTTTCCACACCCTCTTACTTTTTGTCGATTGCTGCAT
Brb_F        TTTTTCATCCTCATCGATTGTGTTTTTCCACACCCTCTTACTTTTTGTCGATTGCTGCAT
Brb_R        TTTTTCATCCTCATCGATTGTGTTTTTCCACACCCTCTTACTTTTTGTCGATTGCTGCAT
             ************************************************************

Pallas_F     GCAGAGGATGGCGGCGTTGTCTGGGAGATGGGCAAATGATGTTGATGCACTTTGAGTTGG
Pallas_R     GCAGAGGATGGCGGCGTTGTCTGGGAGATGGGCAAATGATGTTGATGCACTTTGAGTTGG
Brb_F        GCAGAGGATGGCGGCGTTGTCTGGGAGATGGGCAAATGATGTTGATGCACTTTGAGTTGG
Brb_R        GCAGAGGATGGCGGCGTTGTCTGGGAGATGGGCAAATGATGTTGATGCACTTTGAGTTGG
             ************************************************************

Pallas_F     TAACAATCTGGTTTGGCAGACTCACTTGGCTTGCAGACTCACTTGGCTTCACTTATGCTG
Pallas_R     TAACAATCTGGTTTGGCAGACTCACTTGGCTTGCAGACTCACTTGGCTTCACTTATGCTG
Brb_F        TAACAATCTGGTTTGGCAGACTCACTTGGCTTGCAGACTCACTTGGCTTCACTTATGCTG
Brb_R        TAACAATCTGGTTTGGCAGACTCACTTGGCTTGCAGACTCACTTGGCTTCACTTATGCTG
             ************************************************************

Pallas_F     CTTAAGCTGTATCACGGAAATAAGCGGAGATTTGTTGTTTCAGACTATCTGTTGAGACTG
Pallas_R     CTTAAGCTGTATCACGGAAATAAGCGGAGATTTGTTGTTTCAGACTATCTGTTGAGACTG
Brb_F        CTTAAGCTGTATCACGGAAATAAGCGGAGATTTGTTGTTTCAGACTATCTGTTGAGACTG
Brb_R        CTTAAGCTGTATCACGGAAATAAGCGGAGATTTGTTGTTTCAGACTATCTGTTGAGACTG
             ************************************************************

Pallas_F     ACAAACGCAGACAATGTTTTCCGGTGTAATTGACACAACCTTGTCCCTGTGTTTCTAGTT
Pallas_R     ACAAACGCAGACAATGTTTTCCGGTGTAATTGACACAACCTTGTCCCTGTGTTTCTAGTT
Brb_F        ACAAACGCAGACAATGTTTTCCGGTGTAATTGACACAACCTTGTCCCTGTGTTTCTAGTT
Brb_R        ACAAACGCAGACAATGTTTTCCGGTGTAATTGACACAACCTTGTCCCTGTGTTTCTAGTT
             ************************************************************

Pallas_F     TGGTTGTGCAAGCTGTGTGATGGTTCAAACTTTATATTCGGCAAGCAAGCTGAAAACACC
Pallas_R     TGGTTGTGCAAGCTGTGTGATGGTTCAAACTTTATATTCGGCAAGCAAGCTGAAAACACC
Brb_F        TGGTTGTGCAAGCTGTGTGATGGTTCAAACTTTATATTCGGCAAGCAAGCTGAAAACACC
Brb_R        TGGTTGTGCAAGCTGTGTGATGGTTCAAACTTTATATTCGGCAAGCAAGCTGAAAACACC
             ************************************************************

Pallas_F     AGTCATCAACGCATCTGTAGCTGTCATTTGTAGGCATCATTAGTACTGCACAGGTAAGAA
Pallas_R     AGTCATCAACGCATCTGTAGCTGTCATTTGTAGGCATCATTAGTACTGCACAGGTAAGAA
Brb_F        AGTCATCAACGCATCTGTAGCTGTCATTTGTAGGCATCATTAGTACTGCACAGGTAAGAA
Brb_R        AGTCATCAACGCATCTGTAGCTGTCATTTGTAGGCATCATTAGTACTGCACAGGTAAGAA
             ************************************************************
```

```
Pallas_F      CAATCTGAAACTTTGTCTGCAGGTAGTTGCACGTCGTGATCAAAATAGTGTACCATGATT
Pallas_R      CAATCTGAAACTTTGTCTGCAGGTAGTTGCACGTCGTGATCAAAATAGTGTACCATGATT
Brb_F         CAATCTGAAACTTTGTCTGCAGGTAGTTGCACGTCGTGATCAAAATAGTGTACCATGATT
Brb_R         CAATCTGAAACTTTGTCTGCAGGTAGTTGCACGTCGTGATCAAAATAGTGTACCATGATT
              ************************************************************

Pallas_F      GATCTATTTTGTTTTGTTTATTATACCATGAAGTCTGCAGAAATCGTTACCATGATTAAT
Pallas_R      GATCTATTTTGTTTTGTTTATTATACCATGAAGTCTGCAGAAATCGTTACCATGATTAAT
Brb_F         GATCTATTTTGTTTTGTTTATTATACCATGAAGTCTGCAGAAATCGTTACCATGATTAAT
Brb_R         GATCTATTTTGTTTTGTTTATTATACCATGAAGTCTGCAGAAATCGTTACCATGATTAAT
              ************************************************************

Pallas_F      CTATTTTGTTTTGTTTATTATACCATGAAGTCTGGCAGAAATCGTCGTGCCCTCTGTATA
Pallas_R      CTATTTTGTTTTGTTTATTATACCATGAAGTCTGGCAGAAATCGTCGTGCCCTCTGTATA
Brb_F         CTATTTTGTTTTGTTTATTATACCATGAAGTCTGGCAGAAATCGTCGTGCCCTCTGTATA
Brb_R         CTATTTTGTTTTGTTTATTATACCATGAAGTCTGGCAGAAATCGTCGTGCCCTCTGTATA
              ************************************************************

Pallas_F      ATATTGGCAGCAGAAGTTGTTTGGTGATCAATAAATGCAAAAATAATAAATATCGGAAAA
Pallas_R      ATATTGGCAGCAGAAGTTGTTTGGTGATCAATAAATGCAAAAATAATAAATATCGGAAAA
Brb_F         ATATTGGCAGCAGAAGTTGTTTGGTGATCAATAAATGCAAAAATAATAAATATCGGAAAA
Brb_R         ATATTGGCAGCAGAAGTTGTTTGGTGATCAATAAATGCAAAAATAATAAATATCGGAAAA
              ************************************************************

Pallas_F      ATGTTTGAAGTAGTGCTATGCCATATACAGTTGGTTGGCCTCTGCTGTACTGGAGACAGA
Pallas_R      ATGTTTGAAGTAGTGCTATGCCATATACAGTTGGTTGGCCTCTGCTGTACTGGAGACAGA
Brb_F         ATGTTTGAAGTAGTGCTATGCCATATACAGTTGGTTGGCCTCTGCTGTACTGGAGACAGA
Brb_R         ATGTTTGAAGTAGTGCTATGCCATATACAGTTGGTTGGCCTCTGCTGTACTGGAGACAGA
              ************************************************************

Pallas_F      TATGATAGAAATTTGCATGCCCAAGAAGACAGATGCTTACCATATGCCTCATTTGTCTCC
Pallas_R      TATGATAGAAATTTGCATGCCCAAGAAGACAGATGCTTACCATATGCCTCATTTGTCTCC
Brb_F         TATGATAGAAATTTGCATGCCCAAGAAGACAGATGCTTACCATATGCCTCATTTGTCTCC
Brb_R         TATGATAGAAATTTGCATGCCCAAGAAGACAGATGCTTACCATATGCCTCATTTGTCTCC
              ************************************************************

Pallas_F      TTCATCTGCTGTGTTGGCACTTCCATCTCCCCATTATGCAAATGCAACCTTTCTGGCAAA
Pallas_R      TTCATCTGCTGTGTTGGCACTTCCATCTCCCCATTATGCAAATGCAACCTTTCTGGCAAA
Brb_F         TTCATCTGCTGTGTTGGCACTTCCATCTCCCCATTATGCAAATGCAACCTTTCTGGCAAA
Brb_R         TTCATCTGCTGTGTTGGCACTTCCATCTCCCCATTATGCAAATGCAACCTTTCTGGCAAA
              ************************************************************

Pallas_F      CGGAGCGTCTGAATTCATTATCATCGCTTGGAAAGCATTCGAGAAACTTGTGACCAGTTT
Pallas_R      CGGAGCGTCTGAATTCATTATCATCGCTTGGAAAGCATTCGAGAAACTTGTGACCAGTTT
Brb_F         CGGAGCGTCTGAATTCATTATCATCGCTTGGAAAGCATTCGAGAAACTTGTGACCAGTTT
Brb_R         CGGAGCGTCTGAATTCATTATCATCGCTTGGAAAGCATTCGAGAAACTTGTGACCAGTTT
              ************************************************************

Pallas_F      GTTTCATTATATTATTTGTTTCCCGGACTTCATTTCTCTGAAGCGCCTGATTTATTTCTG
Pallas_R      GTTTCATTATATTATTTGTTTCCCGGACTTCATTTCTCTGAAGCGCCTGATTTATTTCTG
Brb_F         GTTTCATTATATTATTTGTTTCCCGGACTTCATTTCTCTGAAGCGCCTGATTTATTTCTG
Brb_R         GTTTCATTATATTATTTGTTTCCCGGACTTCATTTCTCTGAAGCGCCTGATTTATTTCTG
              ************************************************************

Pallas_F      CTGAATGATGATGCGCCCGCTATCTTTTCTCAGACAAAGTCAAAGTCTGGCCTTTCTCTA
Pallas_R      CTGAATGATGATGCGCCCGCTATCTTTTCTCAGACAAAGTCAAAGTCTGGCCTTTCTCTA
Brb_F         CTGAATGATGATGCGCCCGCTATCTTTTCTCAGACAAAGTCAAAGTCTGGCCTTTCTCTA
Brb_R         CTGAATGATGATGCGCCCGCTATCTTTTCTCAGACAAAGTCAAAGTCTGGCCTTTCTCTA
              ************************************************************

Pallas_F      TGCCATGGGCGCACTTGCAAAAGAATAGAGATCCAGCGTCCTAGCCTTTCTCATCCAAGA
Pallas_R      TGCCATGGGCGCACTTGCAAAAGAATAGAGATCCAGCGTCCTAGCCTTTCTCATCCAAGA
Brb_F         TGCCATGGGCGCACTTGCAAAAGAATAGAGATCCAGCGTCCTAGCCTTTCTCATCCAAGA
Brb_R         TGCCATGGGCGCACTTGCAAAAGAATAGAGATCCAGCGTCCTAGCCTTTCTCATCCAAGA
              ************************************************************

Pallas_F      AGGGACACCACATTT
Pallas_R      AGGGACACCACATTT
Brb_F         AGGGACACCACATTT
```

```
Brb_R           AGGGACACCACATTT
                ***************
```
